# Supplementary material for: Genetic drift and purifying selection shape within-host influenza A virus populations during natural swine infections
Source: PLoS Pathog. 2024 Apr 16;20(4):e1012131. doi: 10.1371/journal.ppat.1012131 (PMC11051653; doi:10.1371/journal.ppat.1012131)

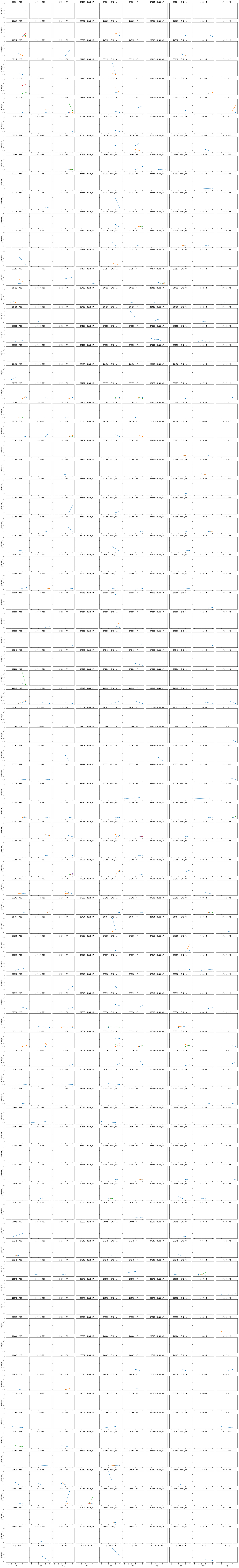

Supplement: S3 Fig — Each row shows iSNV dynamics for a single pig. Columns correspond to the six internal gene segments of IAV, and both lineage I and II HA and NA gene segments. Blank graphs indicate no iSNVs were detected in the reads mapping to each reference sequence. The x-axis specifies the sample day and the y-axis specifies iSNV frequency. (Figure above is representative only, see attached PDF for full length figure). (PDF) [file ppat.1012131.s006.pdf]
