# Supplementary material for: Genetic drift and purifying selection shape within-host influenza A virus populations during natural swine infections
Source: PLoS Pathog. 2024 Apr 16;20(4):e1012131. doi: 10.1371/journal.ppat.1012131 (PMC11051653; doi:10.1371/journal.ppat.1012131)
Supplement: S4 Fig — Each row is a separate sample. The pig ID, sample day, and sample classification are shown in the panel titles of the fifth column. The first four columns show mapping coverage against H1, N1, H3, and N2 references, respectively. The fifth column shows the number of dSNPs detected for each lineage in each of the six internal segments. The remaining six columns show coverage and dSNP frequency by location in the internal segments in the following order: PB2, PB1, PA, NP, M, NS. (Figure above is representative only, see attached PDF for full length figure). (PDF) [file ppat.1012131.s007.pdf]

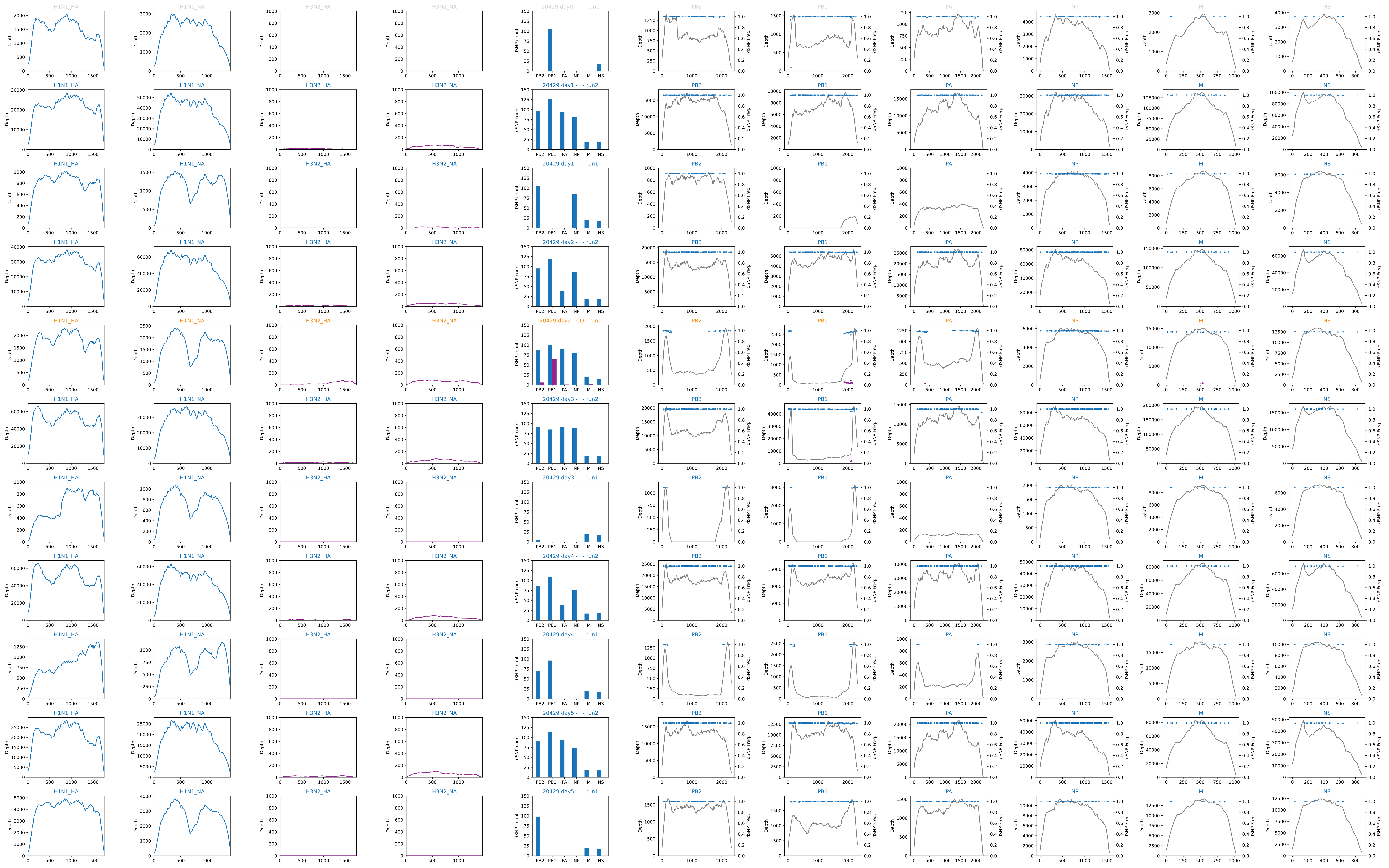
H1N1_HA
H1N1_NA
H3N2_HA
H3N2_NA
20429 day0 - -- - run1
PB2
PB1
PA
NP
M
NS
20429 day1 - I - run2
20429 day1 - I - run1
20429 day2 - I - run2
20429 day2 - CO - run1
20429 day3 - I - run2
20429 day3 - I - run1
20429 day4 - I - run2
20429 day4 - I - run1
20429 day5 - I - run2
20429 day5 - I - run1
Depth
dSNP count
dSNP Freq.

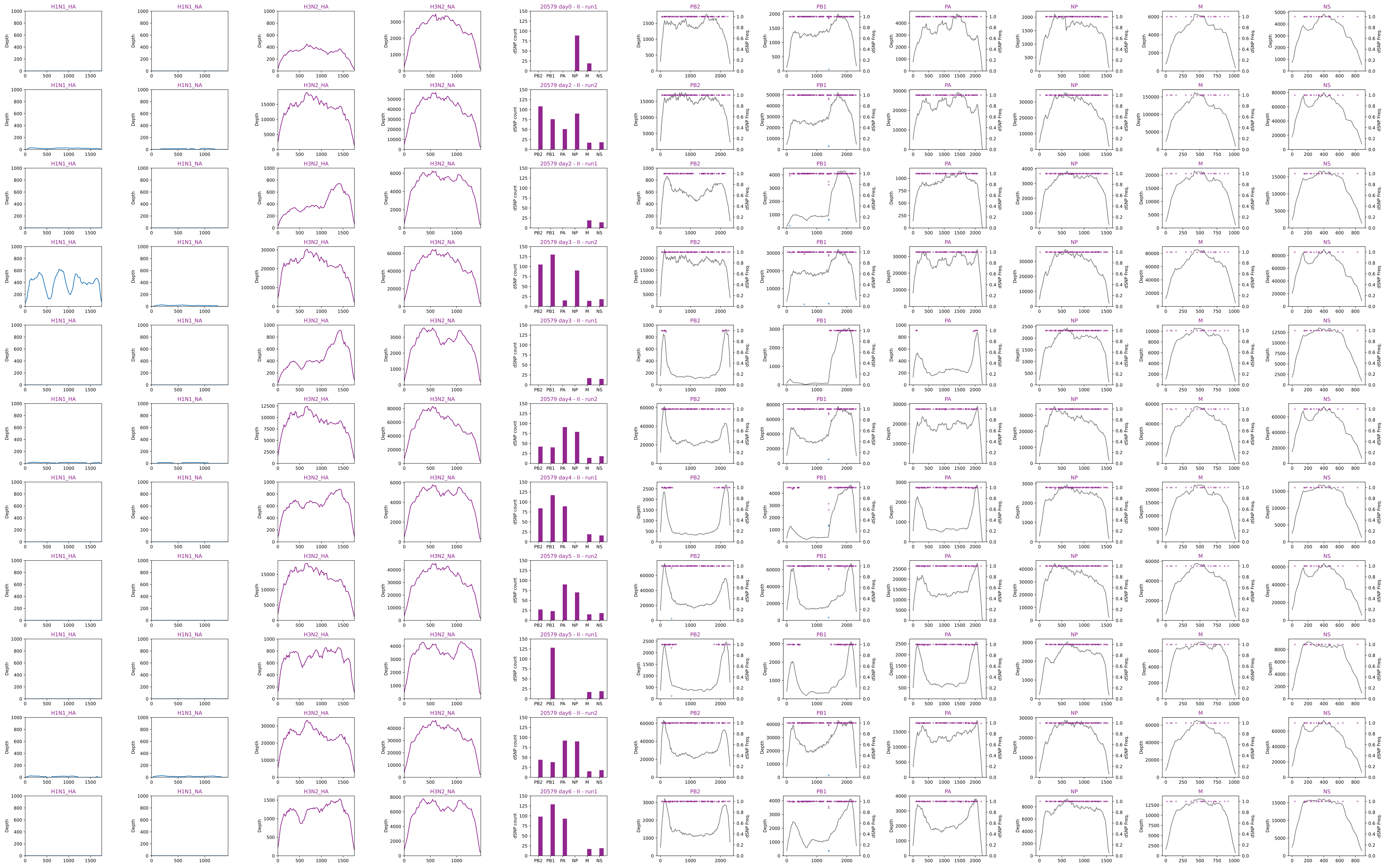
H1N1_HA
H1N1_NA
H3N2_HA
H3N2_NA
PB2
PB1
PA
NP
M
NS
Depth
dSNP count
dSNP Freq.
20579 day0 - II - run1
20579 day2 - II - run2
20579 day2 - II - run1
20579 day3 - II - run2
20579 day3 - II - run1
20579 day4 - II - run2
20579 day4 - II - run1
20579 day5 - II - run2
20579 day5 - II - run1
20579 day6 - II - run2
20579 day6 - II - run1

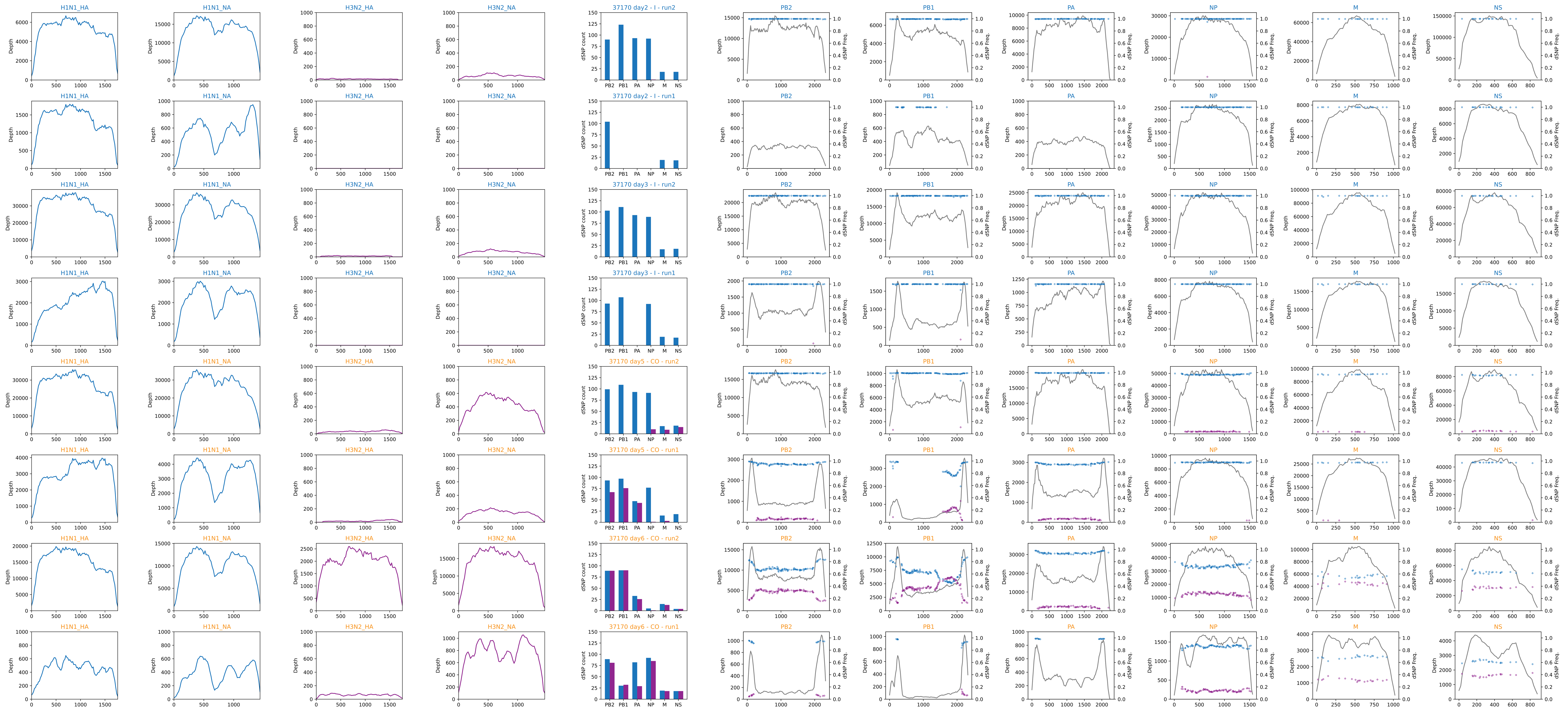
H1N1_HA
H1N1_NA
H3N2_HA
H3N2_NA
37170 day2 - I - run2
37170 day2 - I - run1
37170 day3 - I - run2
37170 day3 - I - run1
37170 day5 - CO - run2
37170 day5 - CO - run1
37170 day6 - CO - run2
37170 day6 - CO - run1
PB2
PB1
PA
NP
M
NS
Depth
dSNP count
dSNP Freq.

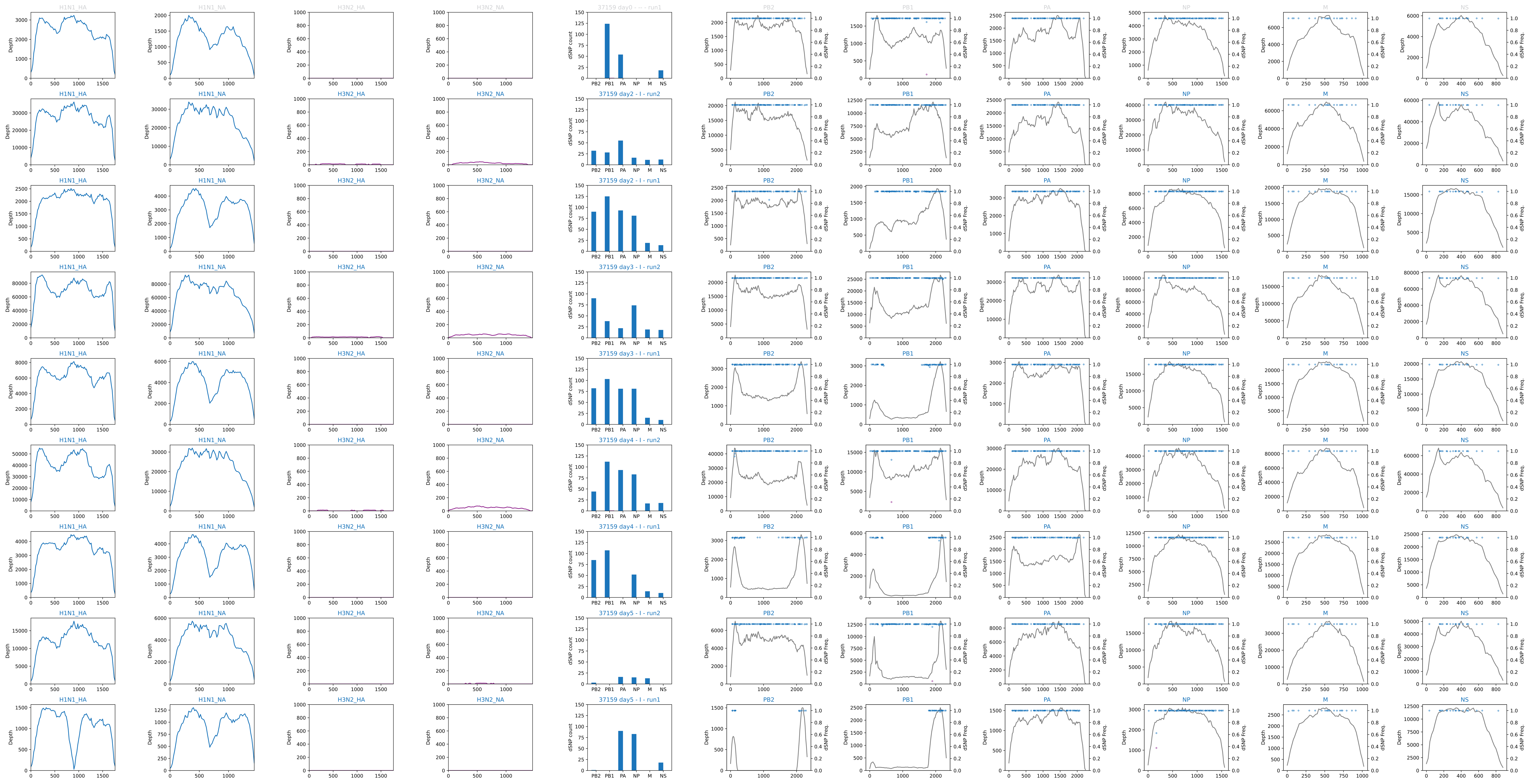

H1N1_HA
H1N1_NA
H3N2_HA
H3N2_NA
PB2
PB1
PA
NP
M
NS
37159 day2 - I - run2
37159 day2 - I - run1
37159 day3 - I - run2
37159 day3 - I - run1
37159 day4 - I - run2
37159 day4 - I - run1
37159 day5 - I - run2
37159 day5 - I - run1
Depth
dSNP count
dSNP Freq.

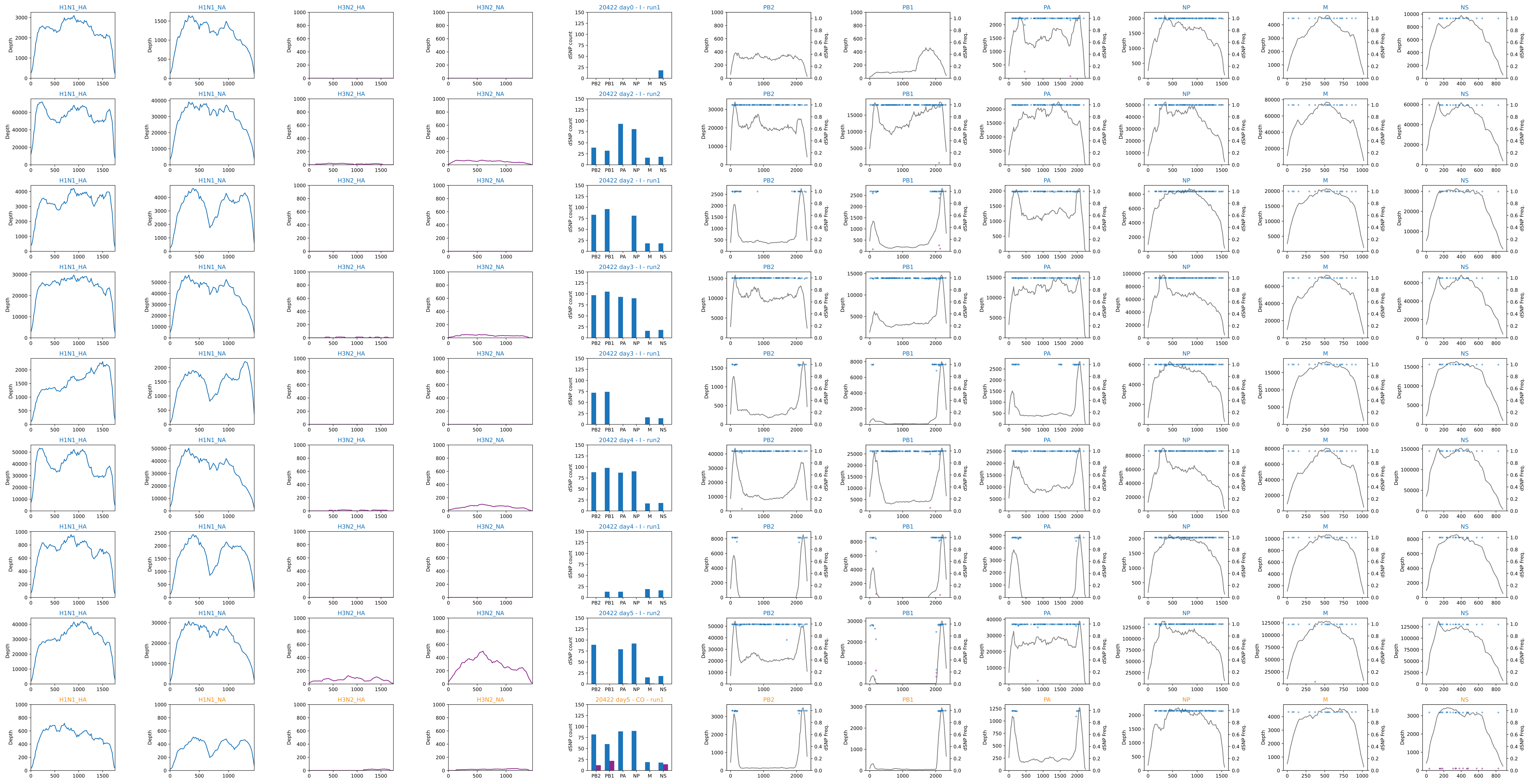
H1N1_HA
H1N1_NA
H3N2_HA
H3N2_NA
20422 day0 - I - run1
20422 day2 - I - run2
20422 day2 - I - run1
20422 day3 - I - run2
20422 day3 - I - run1
20422 day4 - I - run2
20422 day4 - I - run1
20422 day5 - I - run2
20422 day5 - CO - run1
PB2
PB1
PA
NP
M
NS
Depth
dSNP count
dSNP Freq.

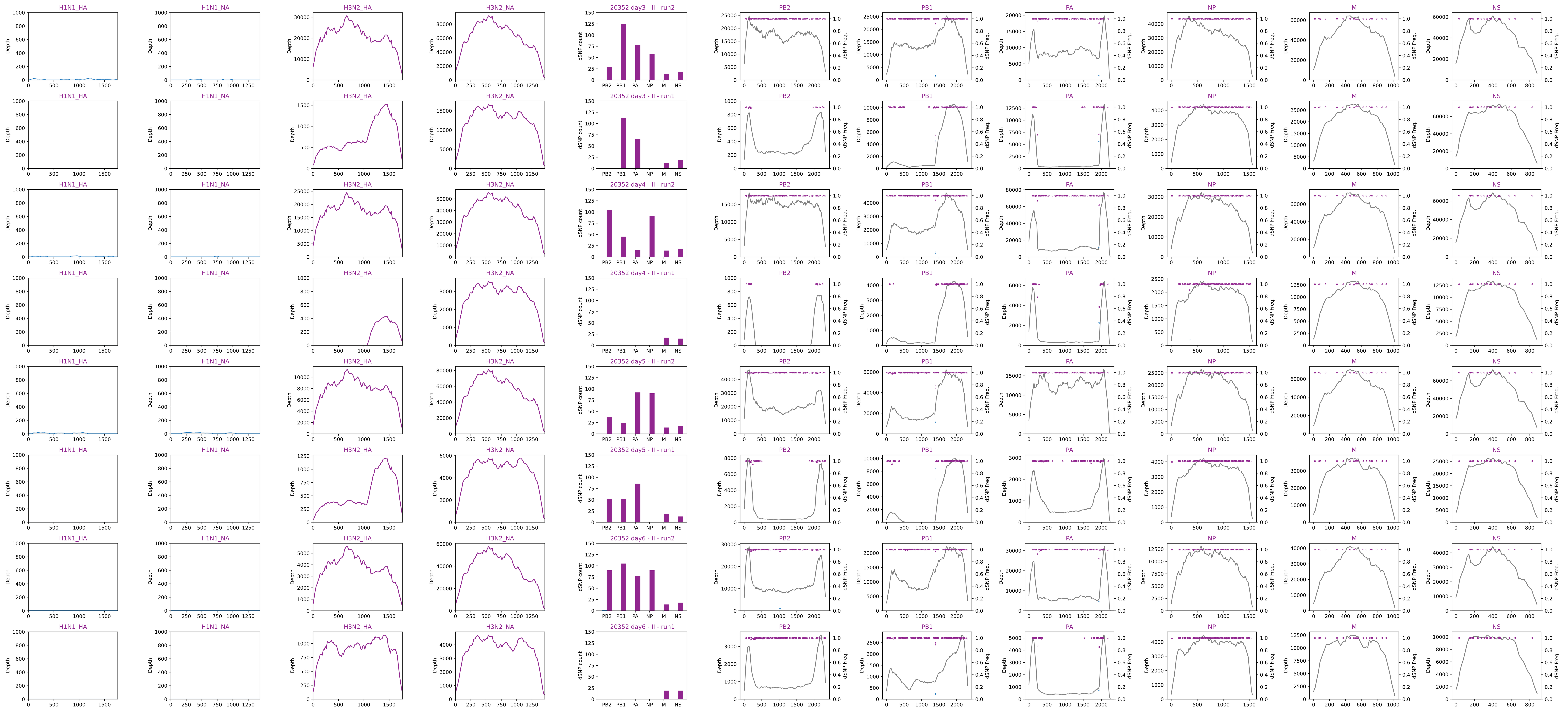
H1N1_HA
H1N1_NA
H3N2_HA
H3N2_NA
20352 day3 - II - run2
20352 day3 - II - run1
20352 day4 - II - run2
20352 day4 - II - run1
20352 day5 - II - run2
20352 day5 - II - run1
20352 day6 - II - run2
20352 day6 - II - run1
PB2
PB1
PA
NP
M
NS
Depth
dSNP count
dSNP Freq.

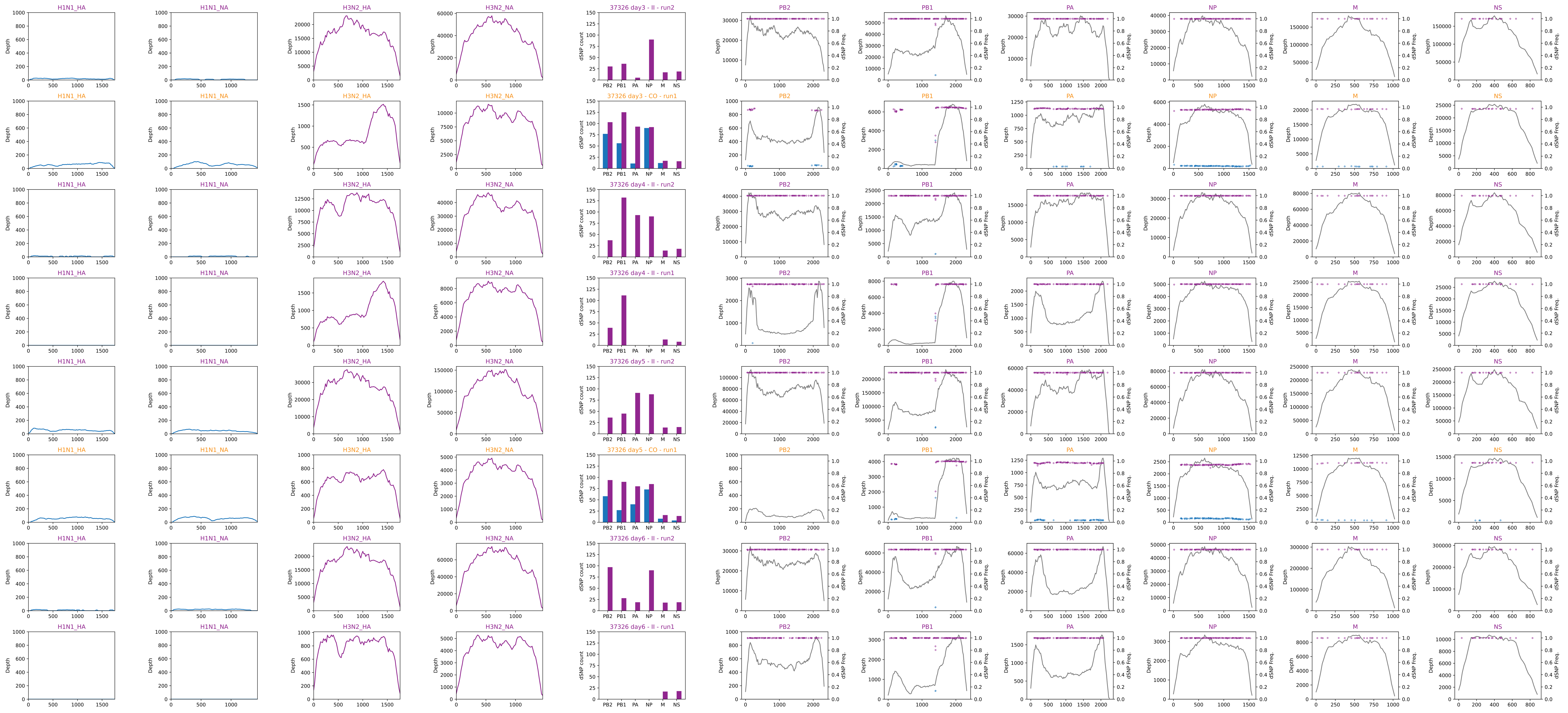

H1N1_HA
H1N1_NA
H3N2_HA
H3N2_NA
37326 day3 - II - run2
37326 day3 - CO - run1
37326 day4 - II - run2
37326 day4 - II - run1
37326 day5 - II - run2
37326 day5 - CO - run1
37326 day6 - II - run2
37326 day6 - II - run1
PB2
PB1
PA
NP
M
NS
Depth
dSNP count
dSNP Freq.

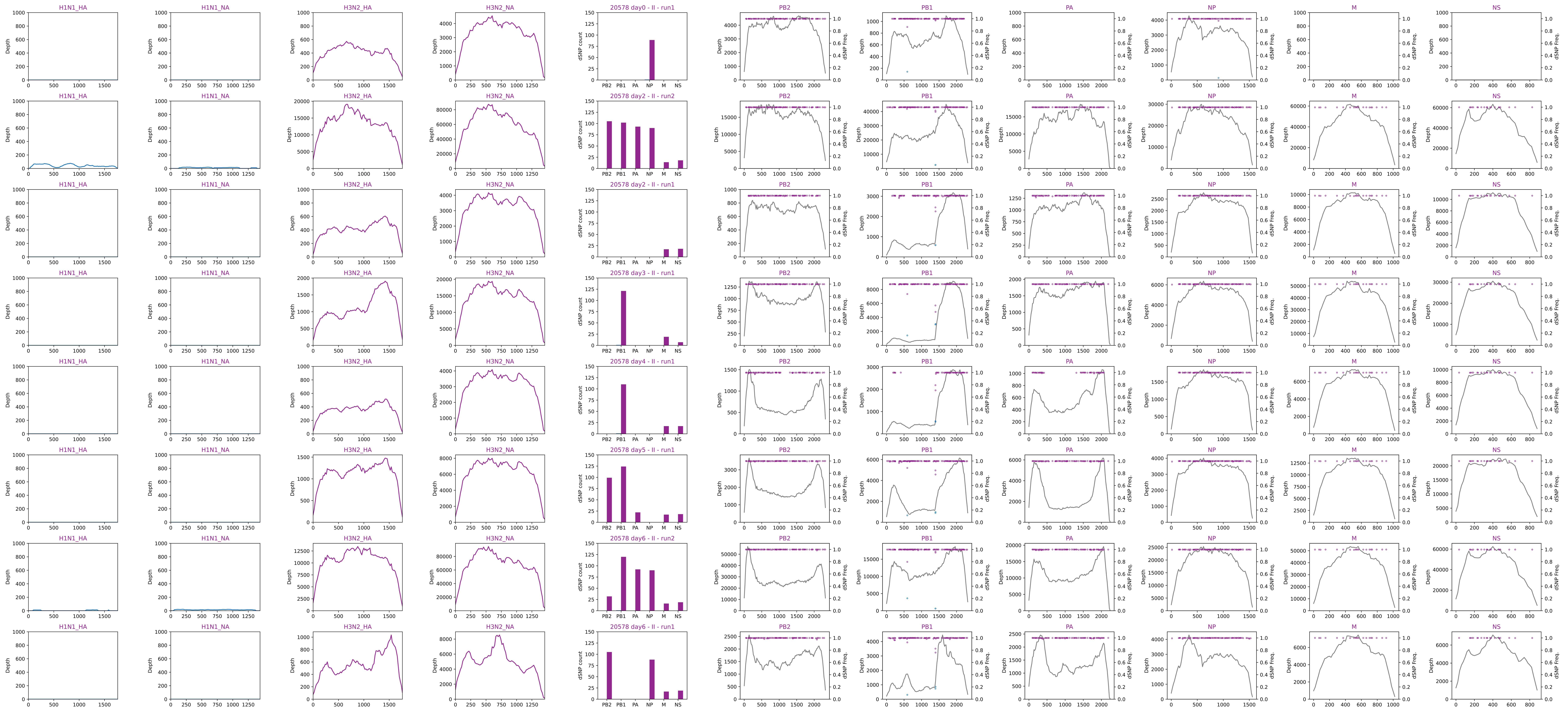
H1N1_HA
H1N1_NA
H3N2_HA
H3N2_NA
20578 day0 - II - run1
20578 day2 - II - run2
20578 day2 - II - run1
20578 day3 - II - run1
20578 day4 - II - run1
20578 day5 - II - run1
20578 day6 - II - run2
20578 day6 - II - run1
PB2
PB1
PA
NP
M
NS
Depth
dSNP count
dSNP Freq.

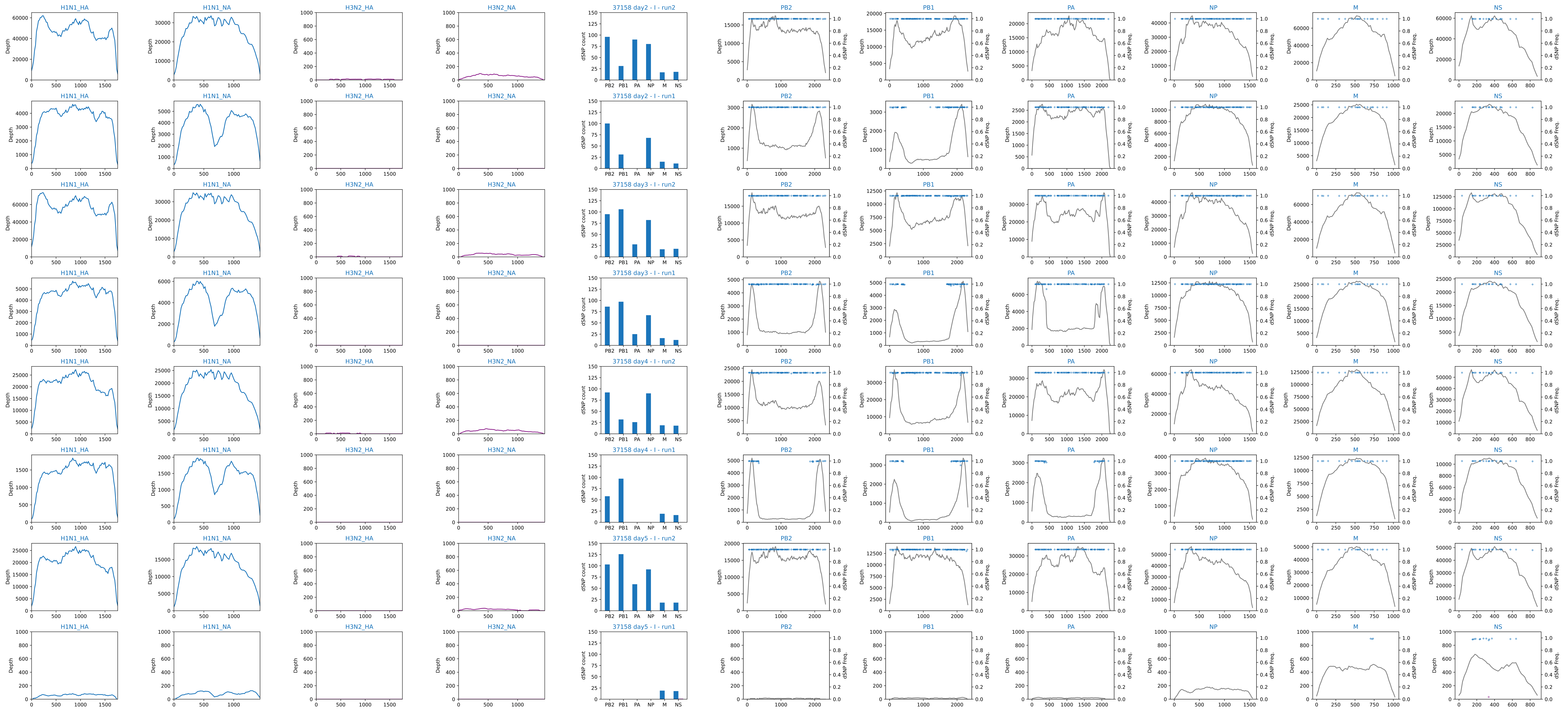

H1N1_HA
H1N1_NA
H3N2_HA
H3N2_NA
37158 day2 - I - run2
37158 day2 - I - run1
37158 day3 - I - run2
37158 day3 - I - run1
37158 day4 - I - run2
37158 day4 - I - run1
37158 day5 - I - run2
37158 day5 - I - run1
PB2
PB1
PA
NP
M
NS
Depth
dSNP count
dSNP Freq.

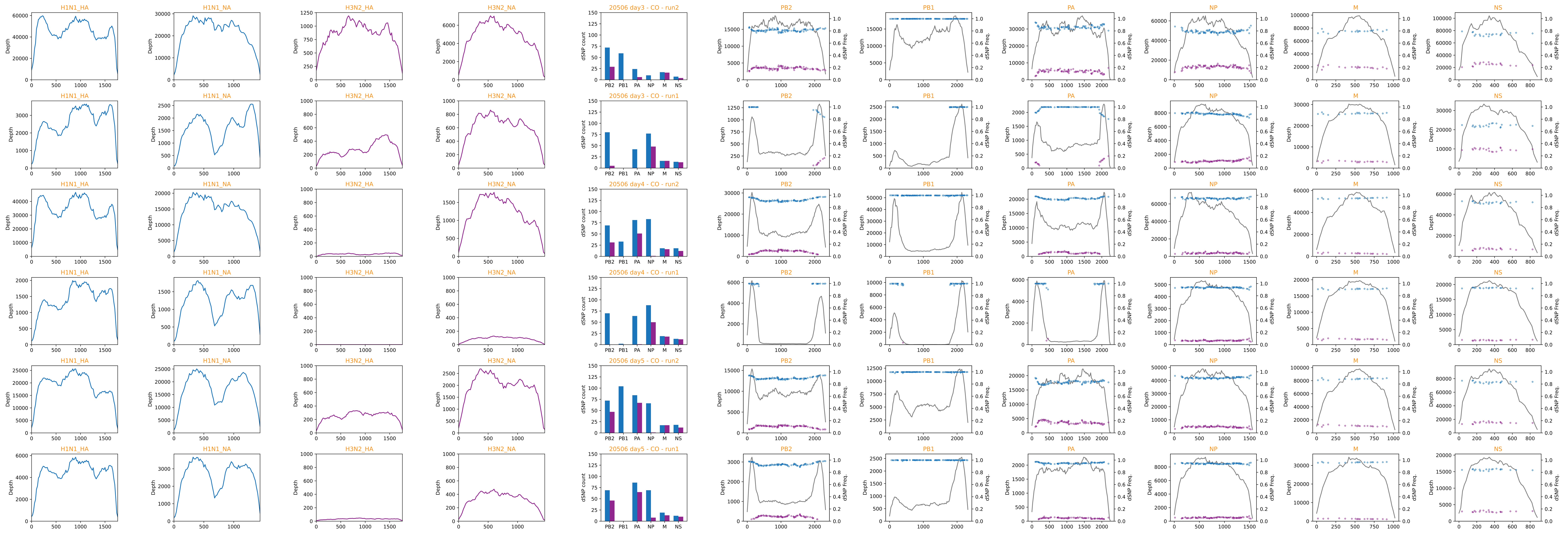

H1N1_HA
H1N1_NA
H3N2_HA
H3N2_NA
20506 day3 - CO - run2
20506 day3 - CO - run1
20506 day4 - CO - run2
20506 day4 - CO - run1
20506 day5 - CO - run2
20506 day5 - CO - run1
PB2
PB1
PA
NP
M
NS
Depth
dSNP count
dSNP Freq.

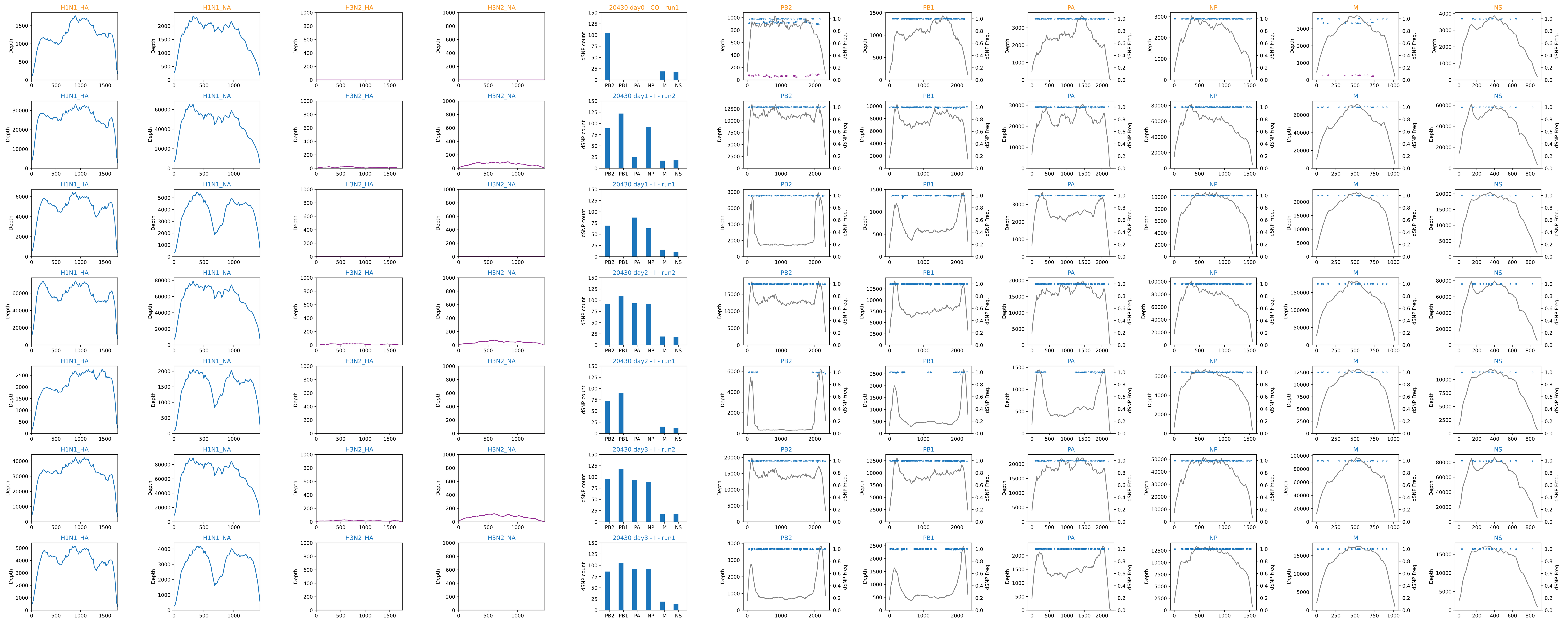
H1N1_HA
H1N1_NA
H3N2_HA
H3N2_NA
20430 day0 - CO - run1
20430 day1 - I - run2
20430 day1 - I - run1
20430 day2 - I - run2
20430 day2 - I - run1
20430 day3 - I - run2
20430 day3 - I - run1
PB2
PB1
PA
NP
M
NS
Depth
dSNP count
dSNP Freq.

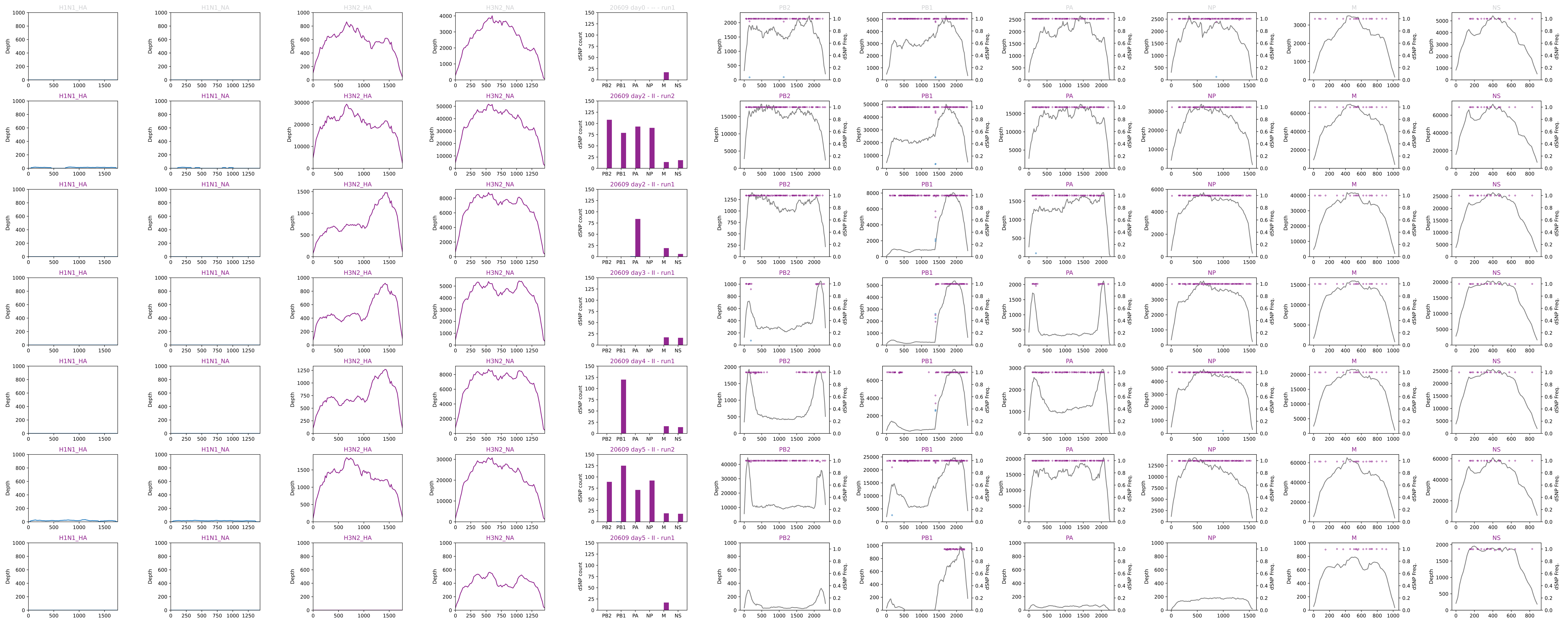
H1N1_HA
H1N1_NA
H3N2_HA
H3N2_NA
20609 day0 - -- - run1
20609 day2 - II - run2
20609 day2 - II - run1
20609 day3 - II - run1
20609 day4 - II - run1
20609 day5 - II - run2
20609 day5 - II - run1
PB2
PB1
PA
NP
M
NS
Depth
dSNP count
dSNP Freq.

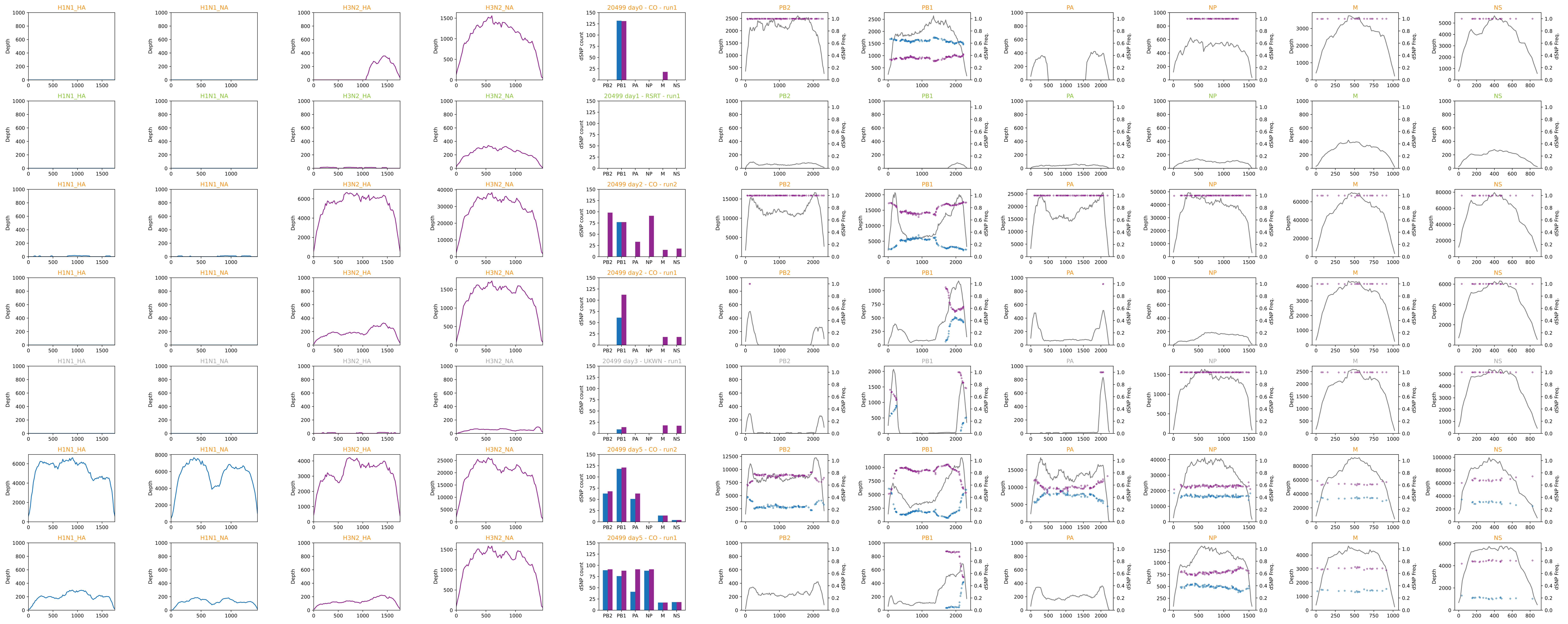
H1N1_HA
H1N1_NA
H3N2_HA
H3N2_NA
20499 day0 - CO - run1
20499 day1 - RSRT - run1
20499 day2 - CO - run2
20499 day2 - CO - run1
20499 day3 - UKWN - run1
20499 day5 - CO - run2
20499 day5 - CO - run1
PB2
PB1
PA
NP
M
NS
Depth
dSNP count
dSNP Freq.

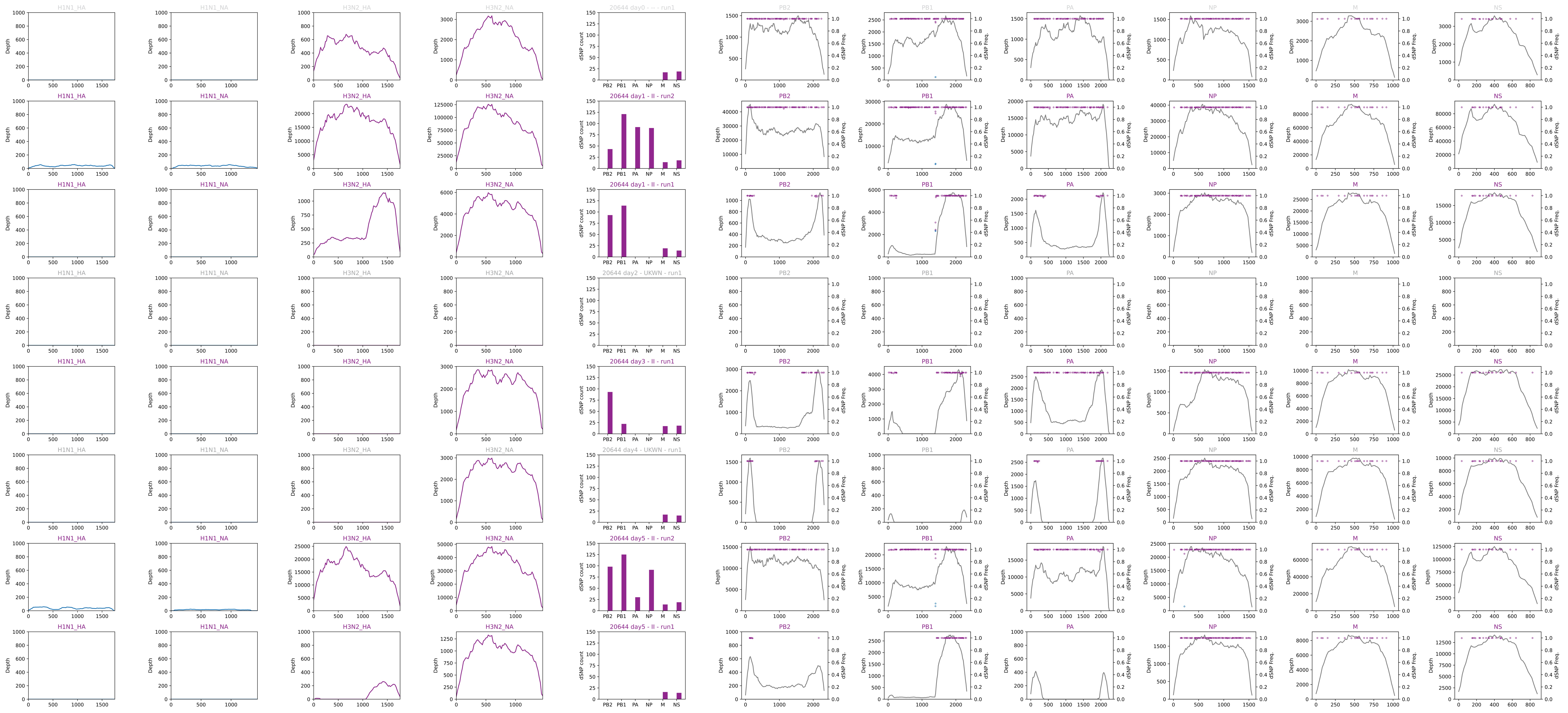
H1N1_HA
H1N1_NA
H3N2_HA
H3N2_NA
20644 day0 - -- - run1
20644 day1 - II - run2
20644 day1 - II - run1
20644 day2 - UKWN - run1
20644 day3 - II - run1
20644 day4 - UKWN - run1
20644 day5 - II - run2
20644 day5 - II - run1
PB2
PB1
PA
NP
M
NS
Depth
dSNP count
dSNP Freq.

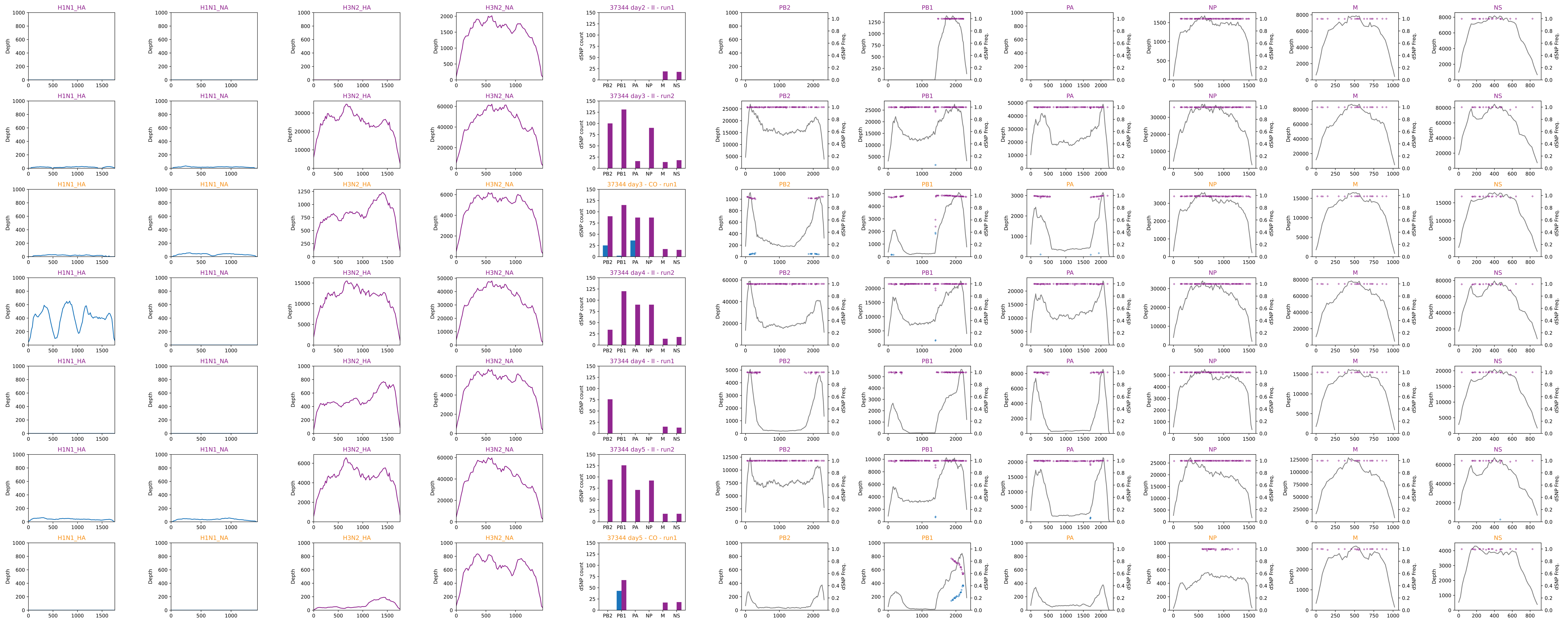
H1N1_HA
H1N1_NA
H3N2_HA
H3N2_NA
37344 day2 - II - run1
37344 day3 - II - run2
37344 day3 - CO - run1
37344 day4 - II - run2
37344 day4 - II - run1
37344 day5 - II - run2
37344 day5 - CO - run1
PB2
PB1
PA
NP
M
NS
Depth
dSNP count
dSNP Freq.

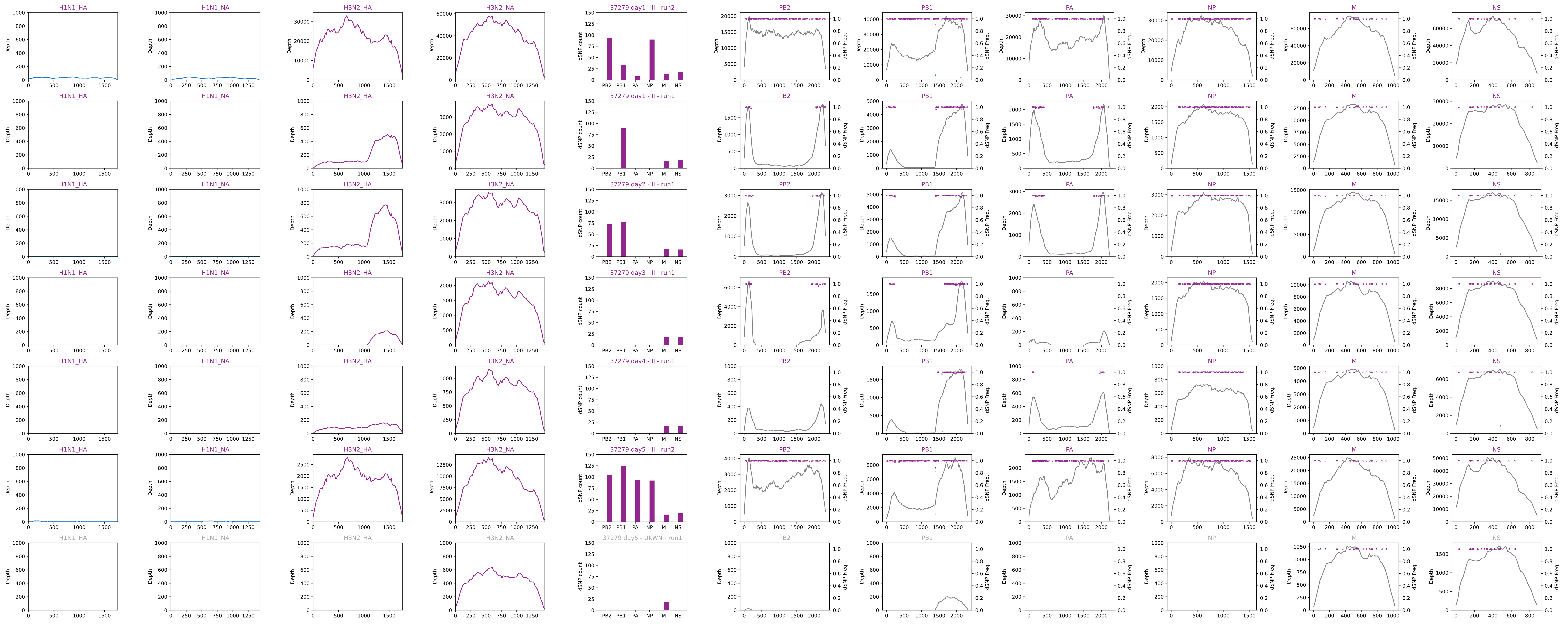
37279 day1 - II - run2
37279 day1 - II - run1
37279 day2 - II - run1
37279 day3 - II - run1
37279 day4 - II - run1
37279 day5 - II - run2
37279 day5 - UKWN - run1
H1N1_HA
H1N1_NA
H3N2_HA
H3N2_NA
PB2
PB1
PA
NP
M
NS
Depth
dSNP count
dSNP Freq.

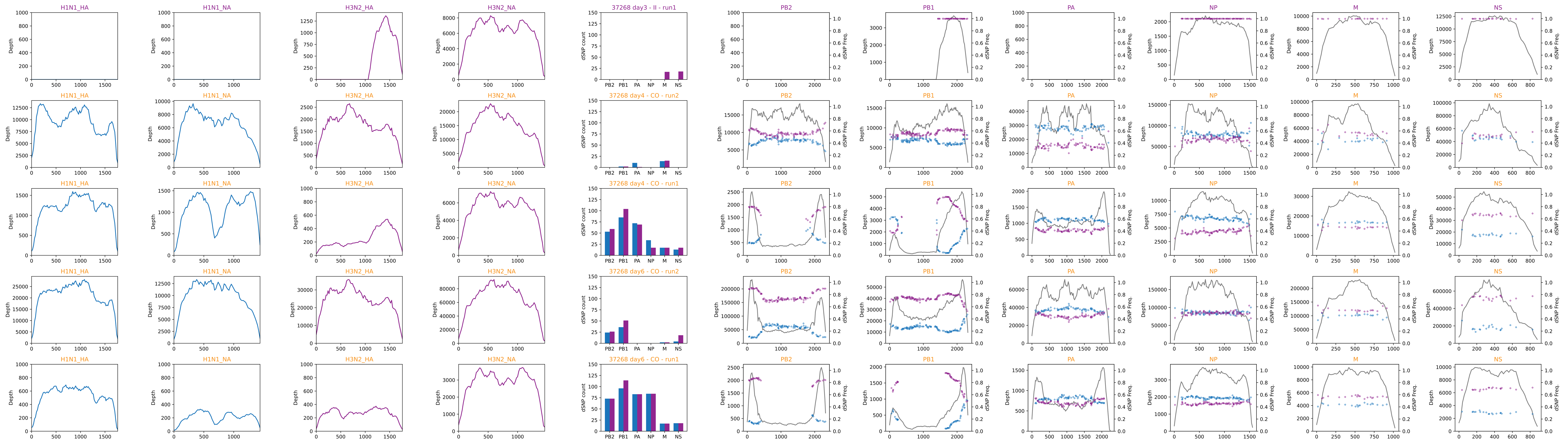

H1N1_HA
H1N1_NA
H3N2_HA
H3N2_NA
37268 day3 - II - run1
PB2
PB1
PA
NP
M
NS
37268 day4 - CO - run2
37268 day4 - CO - run1
37268 day6 - CO - run2
37268 day6 - CO - run1
Depth
dSNP count
dSNP Freq.

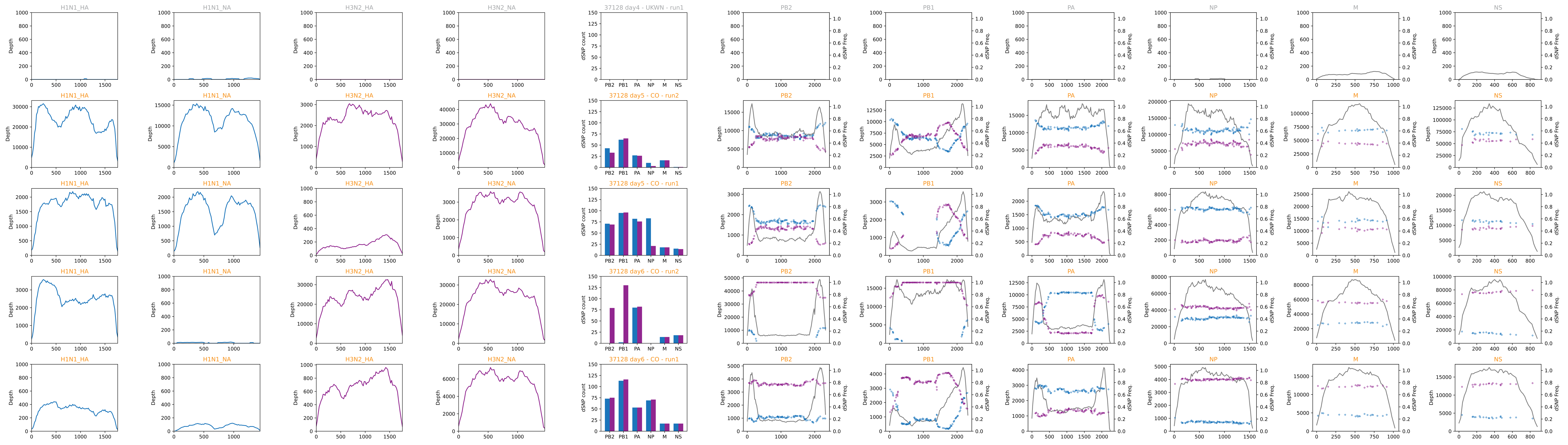

H1N1_HA
H1N1_NA
H3N2_HA
H3N2_NA
37128 day4 - UKWN - run1
PB2
PB1
PA
NP
M
NS
37128 day5 - CO - run2
37128 day5 - CO - run1
37128 day6 - CO - run2
37128 day6 - CO - run1
Depth
dSNP count
dSNP Freq.

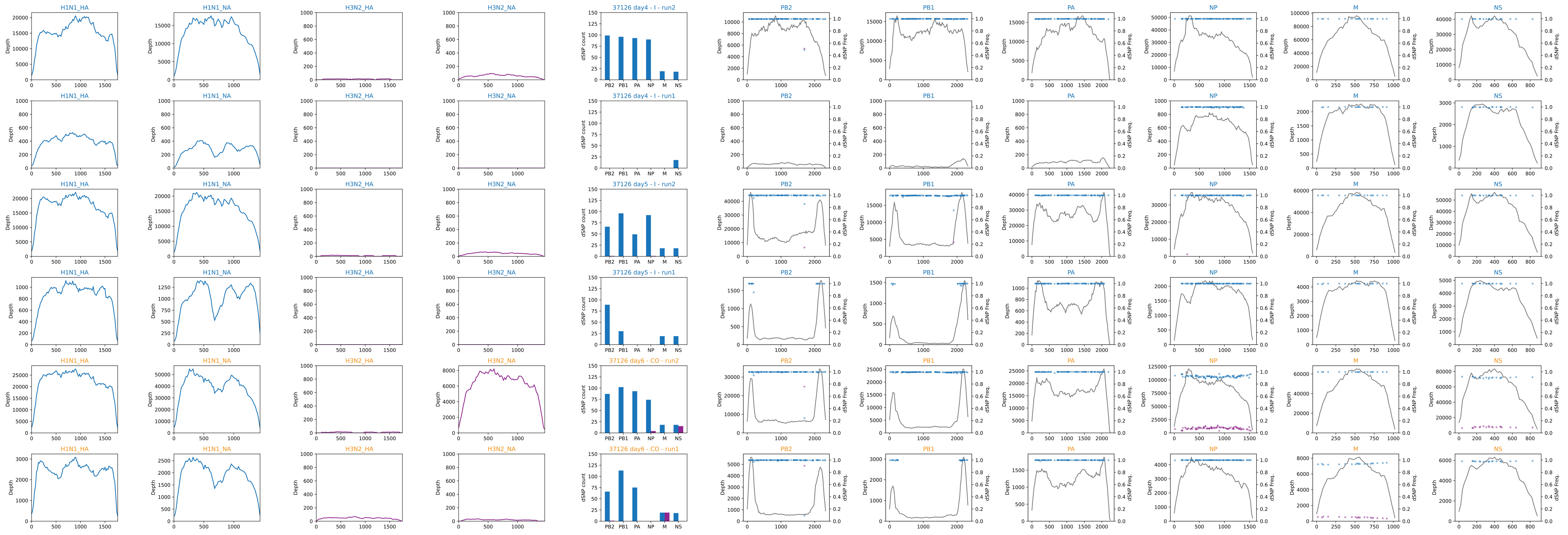

H1N1_HA
H1N1_NA
H3N2_HA
H3N2_NA
37126 day4 - I - run2
PB2
PB1
PA
NP
M
NS
37126 day4 - I - run1
37126 day5 - I - run2
37126 day5 - I - run1
37126 day6 - CO - run2
37126 day6 - CO - run1
Depth
dSNP count
dSNP Freq.

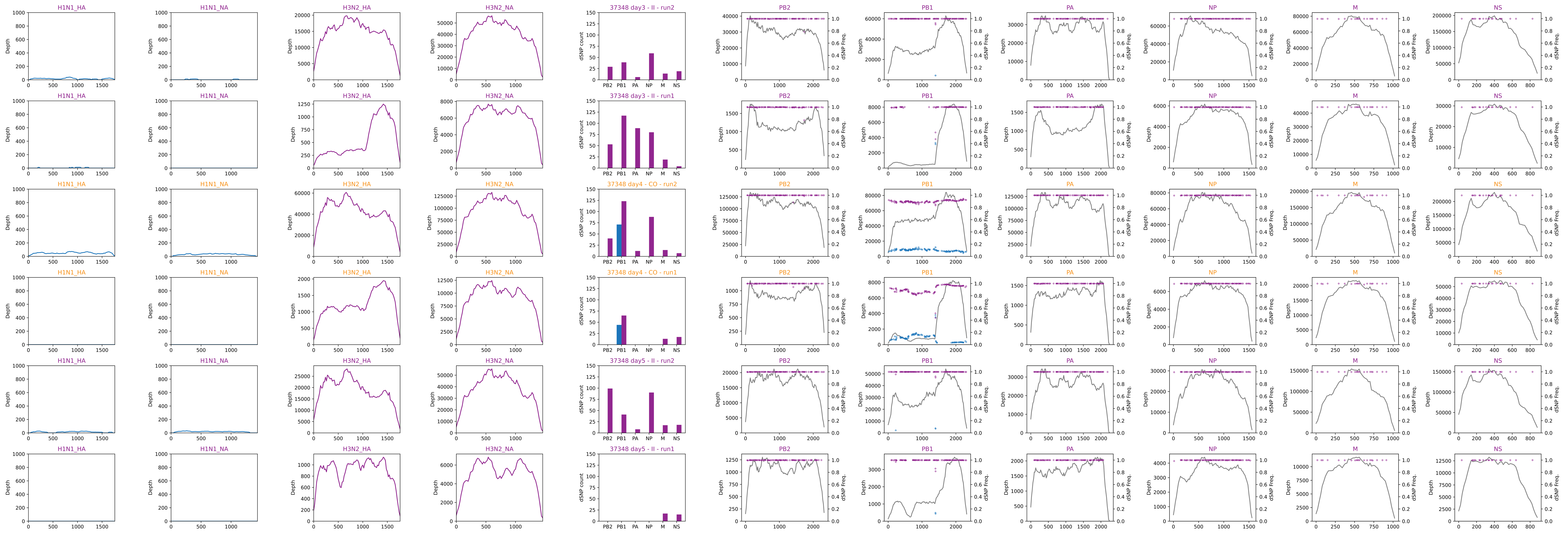
H1N1_HA
H1N1_NA
H3N2_HA
H3N2_NA
37348 day3 - II - run2
PB2
PB1
PA
NP
M
NS
37348 day3 - II - run1
37348 day4 - CO - run2
37348 day4 - CO - run1
37348 day5 - II - run2
37348 day5 - II - run1
Depth
dSNP count
dSNP Freq.

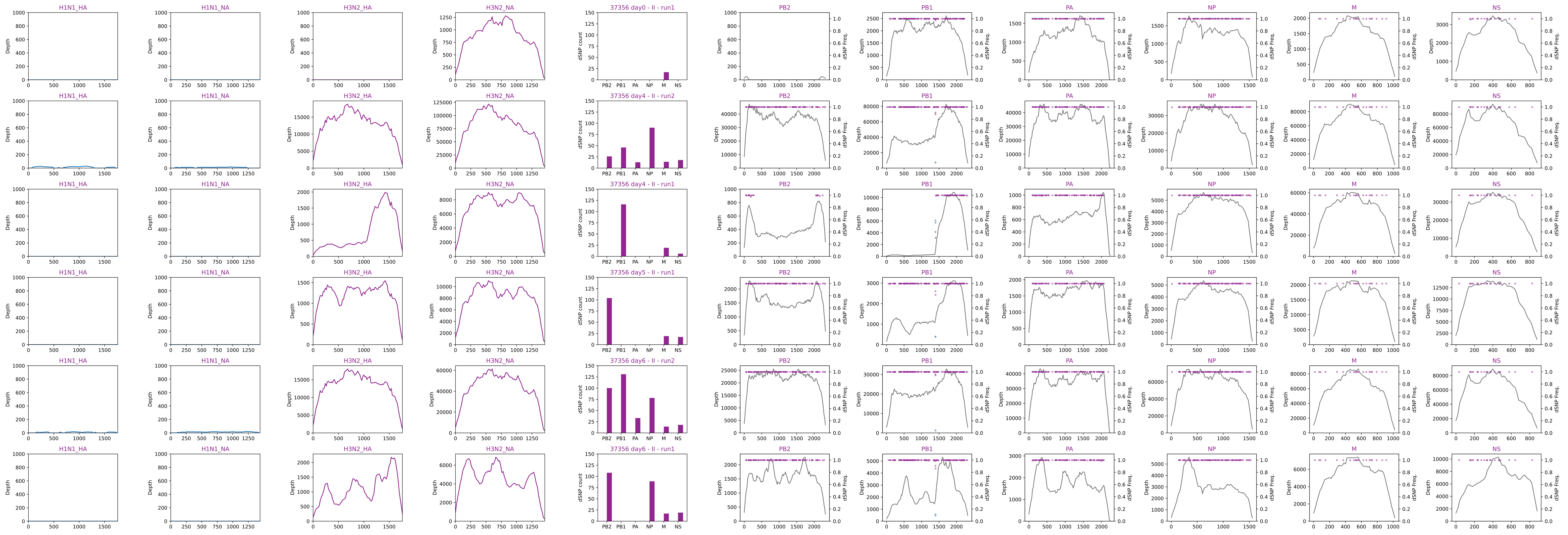

H1N1_HA
H1N1_NA
H3N2_HA
H3N2_NA
37356 day0 - II - run1
37356 day4 - II - run2
37356 day4 - II - run1
37356 day5 - II - run1
37356 day6 - II - run2
37356 day6 - II - run1
PB2
PB1
PA
NP
M
NS
Depth
dSNP count
dSNP Freq.

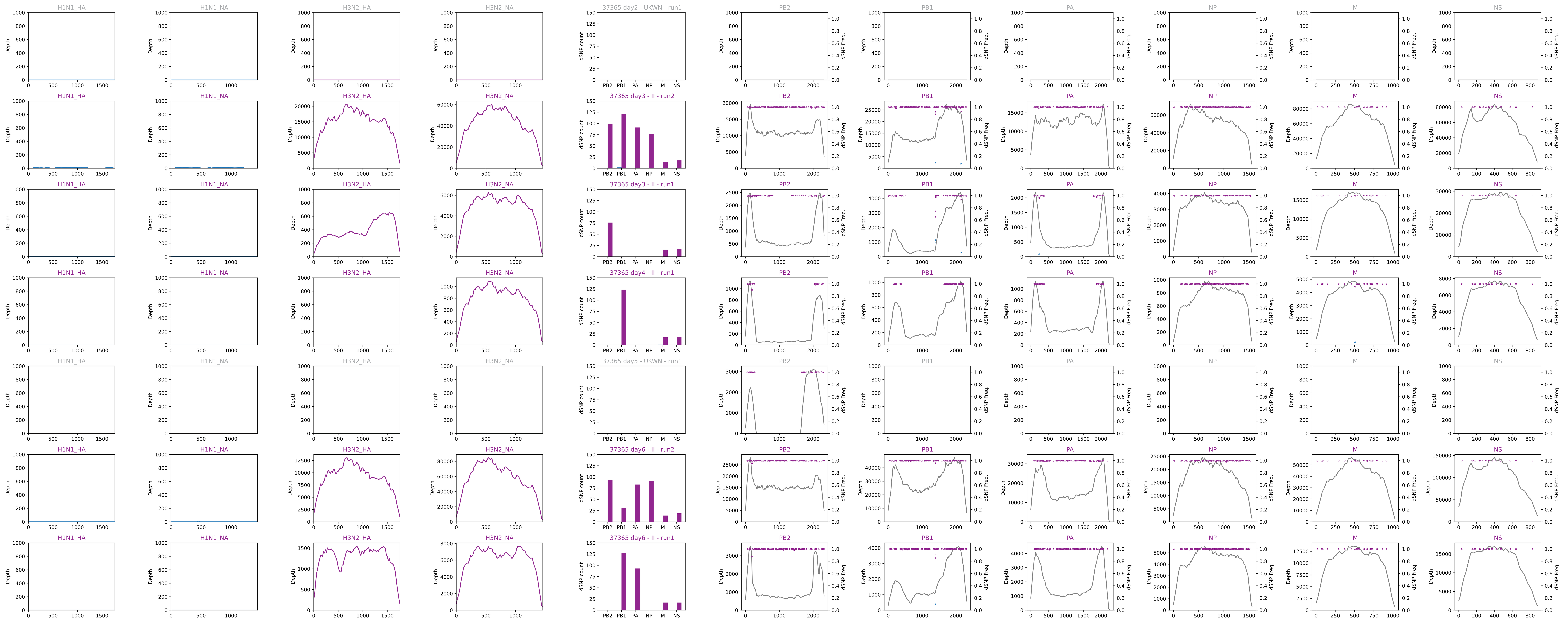

H1N1_HA
H1N1_NA
H3N2_HA
H3N2_NA
37365 day2 - UKWN - run1
PB2
PB1
PA
NP
M
NS
37365 day3 - II - run2
37365 day3 - II - run1
37365 day4 - II - run1
37365 day5 - UKWN - run1
37365 day6 - II - run2
37365 day6 - II - run1
Depth
dSNP count
dSNP Freq.

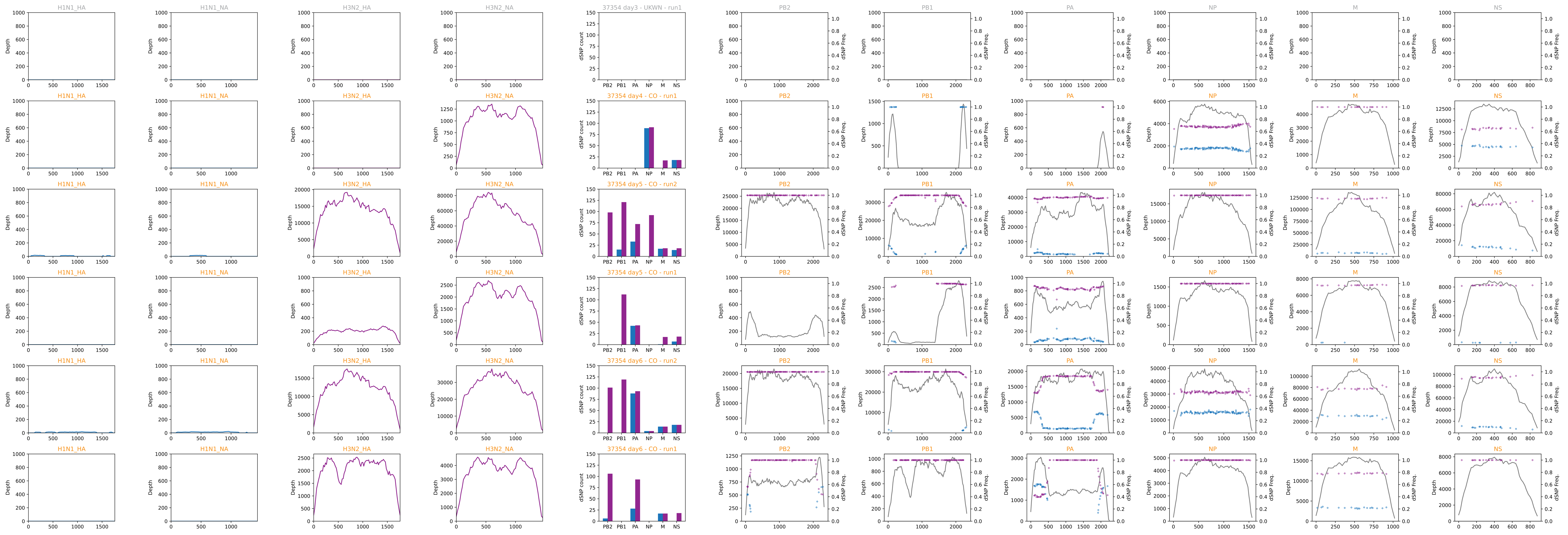
H1N1_HA
H1N1_NA
H3N2_HA
H3N2_NA
37354 day3 - UKWN - run1
37354 day4 - CO - run1
37354 day5 - CO - run2
37354 day5 - CO - run1
37354 day6 - CO - run2
37354 day6 - CO - run1
PB2
PB1
PA
NP
M
NS
Depth
dSNP count
dSNP Freq.

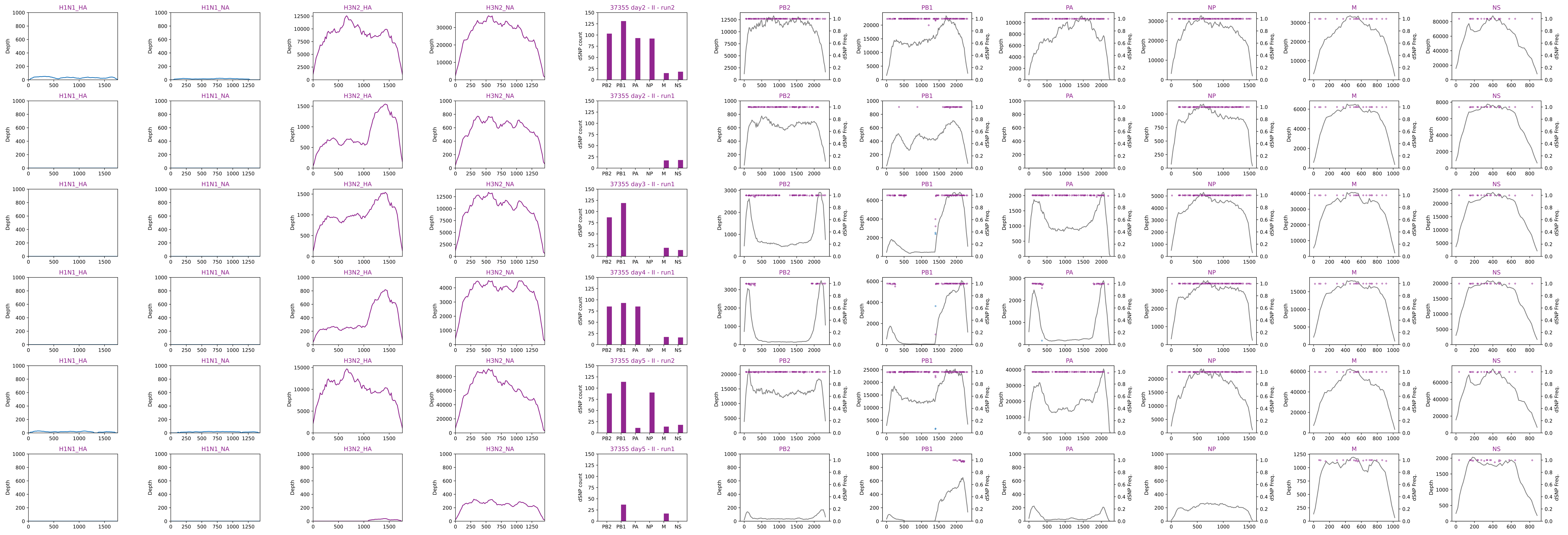
H1N1_HA
H1N1_NA
H3N2_HA
H3N2_NA
37355 day2 - II - run2
PB2
PB1
PA
NP
M
NS
37355 day2 - II - run1
37355 day3 - II - run1
37355 day4 - II - run1
37355 day5 - II - run2
37355 day5 - II - run1
Depth
dSNP count
dSNP Freq.

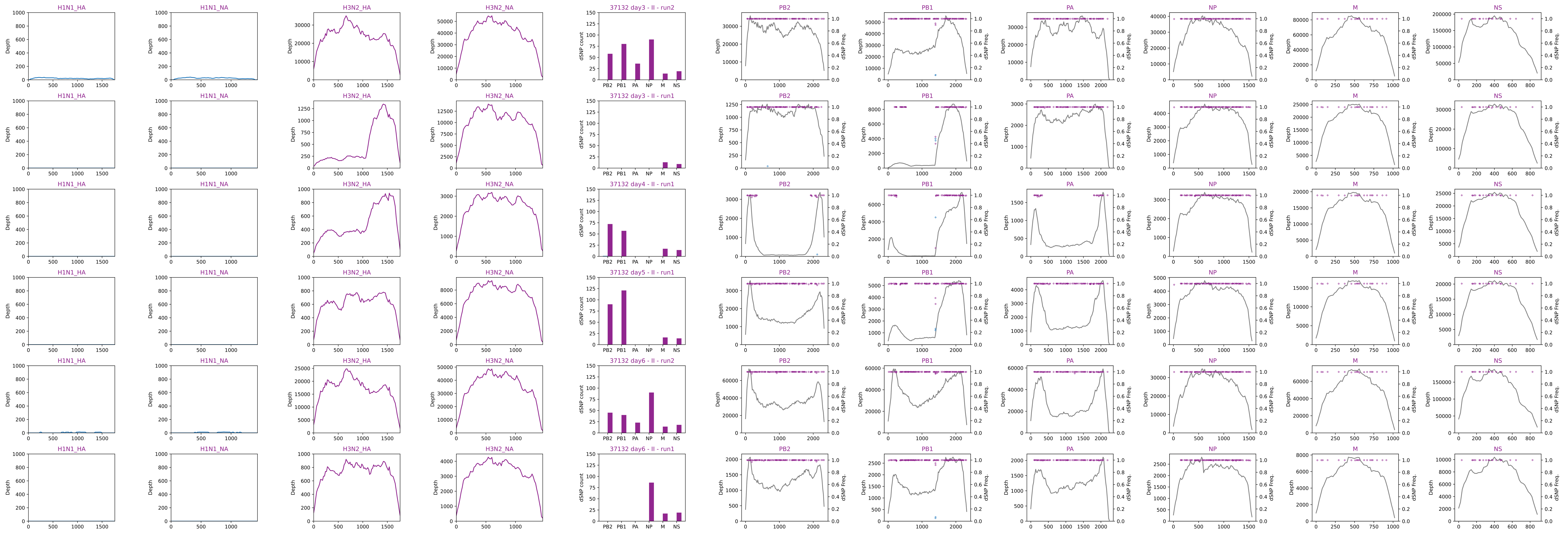
H1N1_HA
H1N1_NA
H3N2_HA
H3N2_NA
37132 day3 - II - run2
37132 day3 - II - run1
37132 day4 - II - run1
37132 day5 - II - run1
37132 day6 - II - run2
37132 day6 - II - run1
PB2
PB1
PA
NP
M
NS
Depth
dSNP count
dSNP Freq.

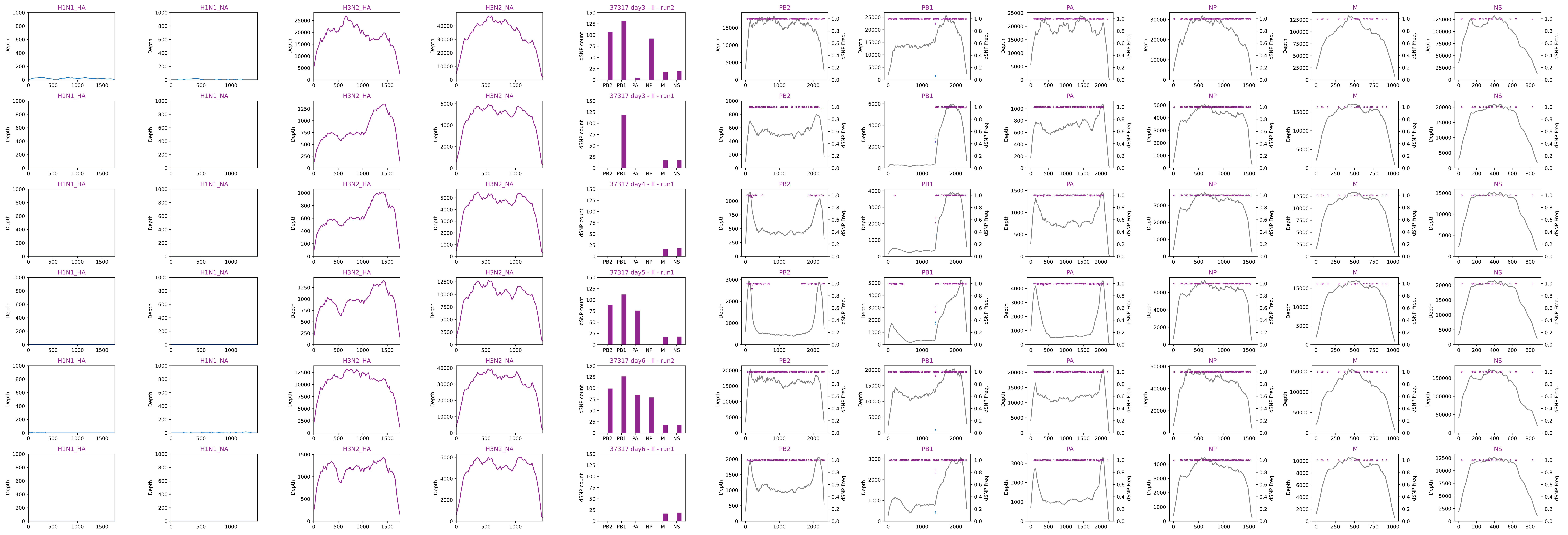

H1N1_HA
H1N1_NA
H3N2_HA
H3N2_NA
37317 day3 - II - run2
37317 day3 - II - run1
37317 day4 - II - run1
37317 day5 - II - run1
37317 day6 - II - run2
37317 day6 - II - run1
PB2
PB1
PA
NP
M
NS
Depth
dSNP count
dSNP Freq.

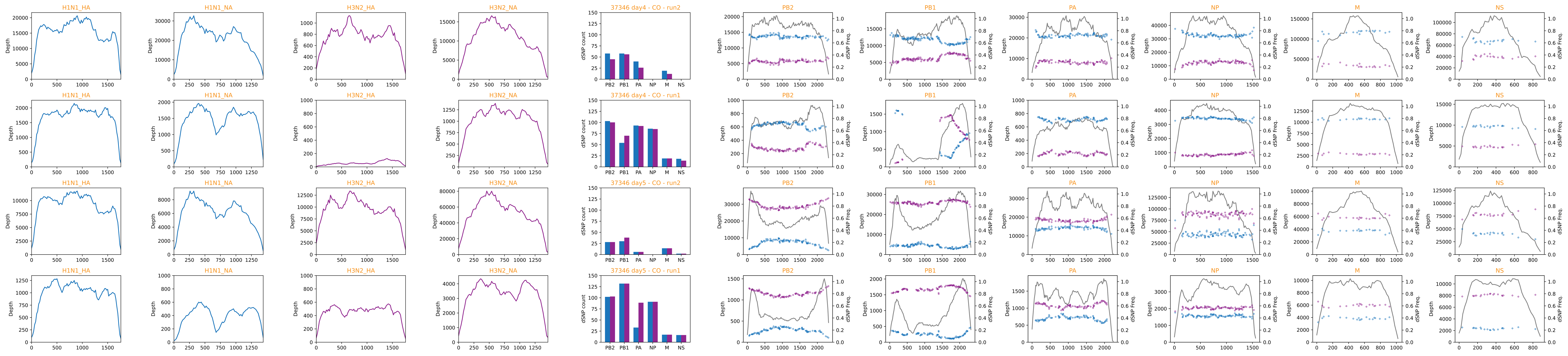
H1N1_HA
H1N1_NA
H3N2_HA
H3N2_NA
37346 day4 - CO - run2
37346 day4 - CO - run1
37346 day5 - CO - run2
37346 day5 - CO - run1
PB2
PB1
PA
NP
M
NS
Depth
dSNP count
dSNP Freq.

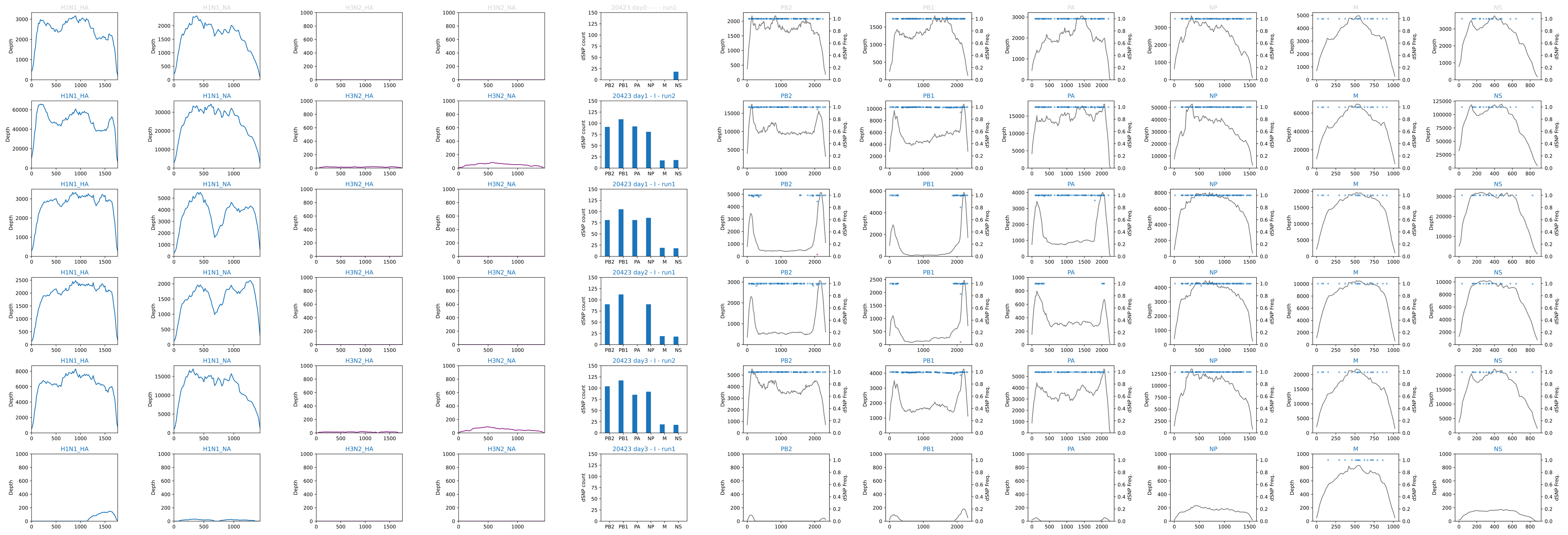
H1N1_HA
H1N1_NA
H3N2_HA
H3N2_NA
20423 day0 - - - run1
20423 day1 - I - run2
20423 day1 - I - run1
20423 day2 - I - run1
20423 day3 - I - run2
20423 day3 - I - run1
PB2
PB1
PA
NP
M
NS
Depth
dSNP count
dSNP Freq.

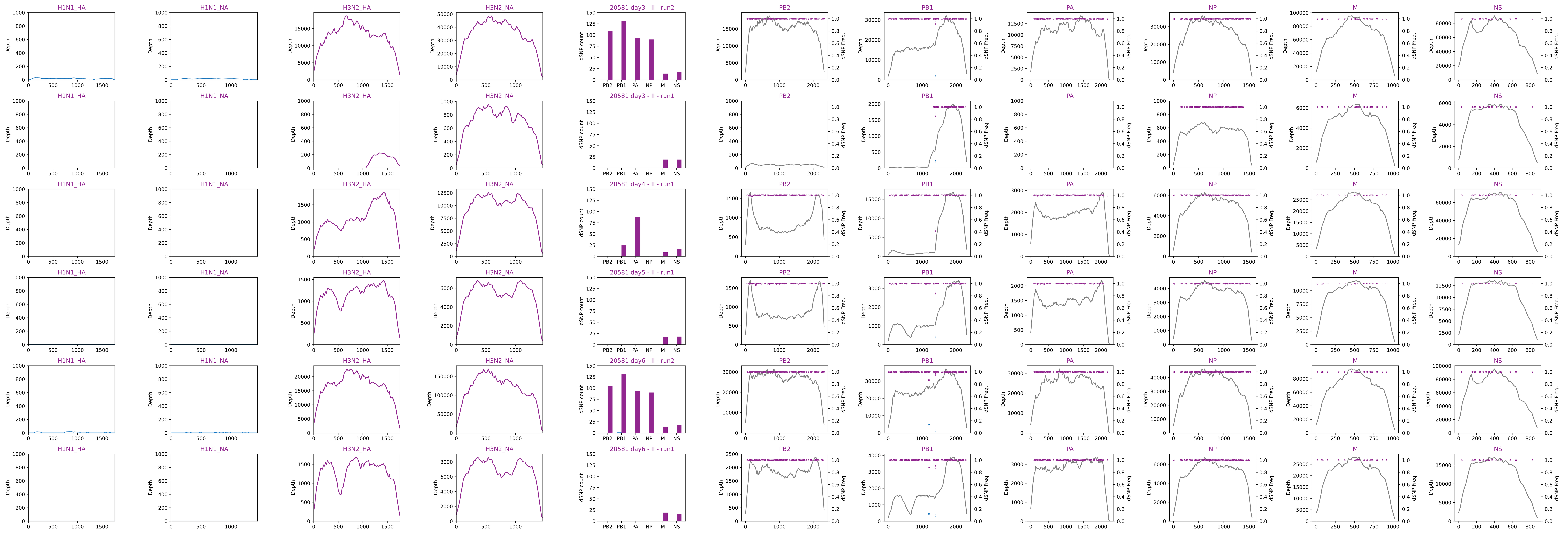
H1N1_HA
H1N1_NA
H3N2_HA
H3N2_NA
20581 day3 - II - run2
20581 day3 - II - run1
20581 day4 - II - run1
20581 day5 - II - run1
20581 day6 - II - run2
20581 day6 - II - run1
PB2
PB1
PA
NP
M
NS
Depth
dSNP count
dSNP Freq.

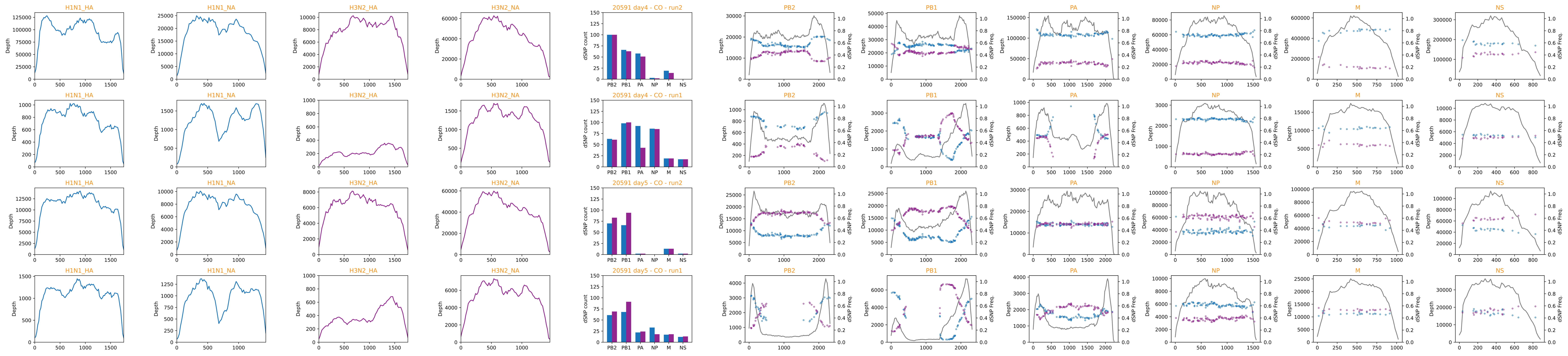
H1N1_HA
H1N1_NA
H3N2_HA
H3N2_NA
20591 day4 - CO - run2
20591 day4 - CO - run1
20591 day5 - CO - run2
20591 day5 - CO - run1
PB2
PB1
PA
NP
M
NS
Depth
dSNP count
dSNP Freq.

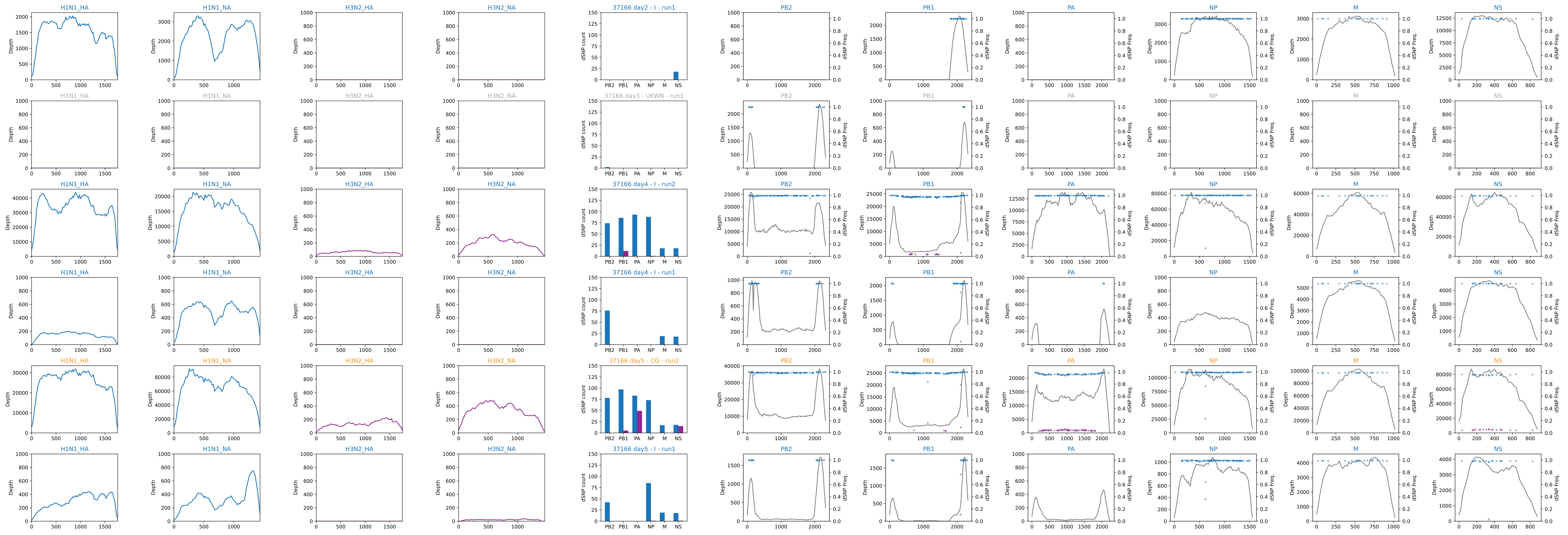

H1N1_HA
H1N1_NA
H3N2_HA
H3N2_NA
37166 day2 - I - run1
37166 day3 - UKWN - run1
37166 day4 - I - run2
37166 day4 - I - run1
37166 day5 - CO - run2
37166 day5 - I - run1
PB2
PB1
PA
NP
M
NS
Depth
dSNP count
dSNP Freq.

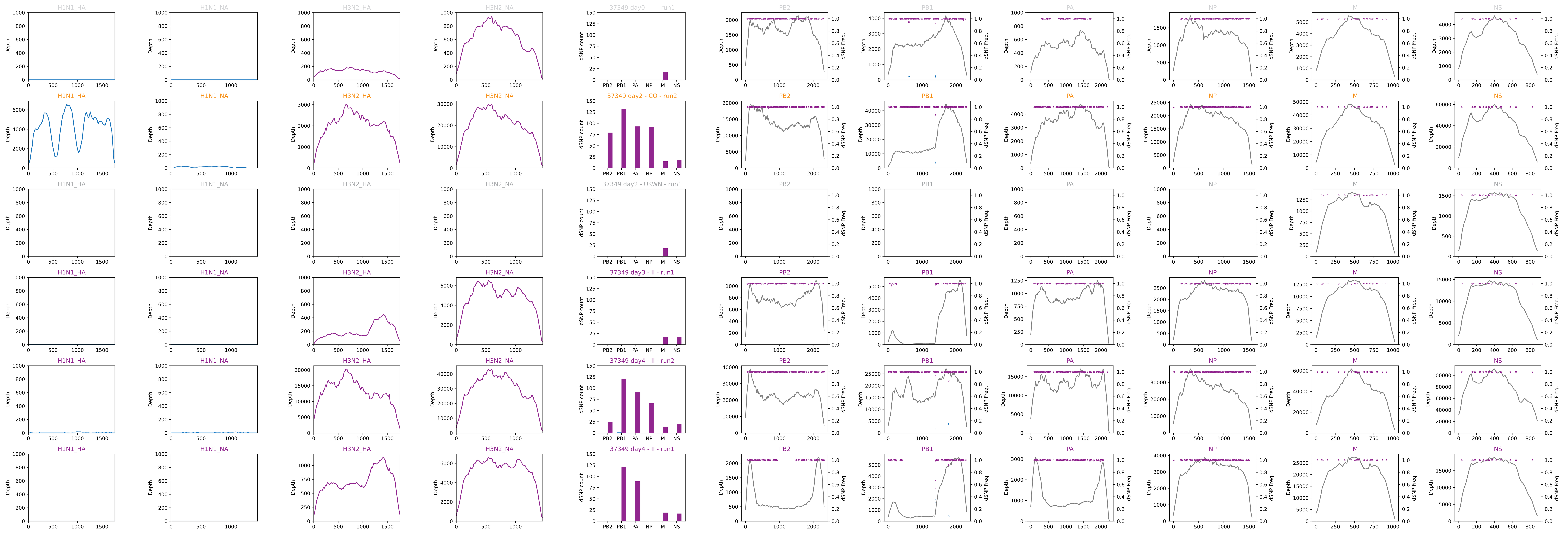

H1N1_HA
H1N1_NA
H3N2_HA
H3N2_NA
37349 day0 - -- - run1
37349 day2 - CO - run2
37349 day2 - UKWN - run1
37349 day3 - II - run1
37349 day4 - II - run2
37349 day4 - II - run1
PB2
PB1
PA
NP
M
NS
Depth
dSNP count
dSNP Freq.

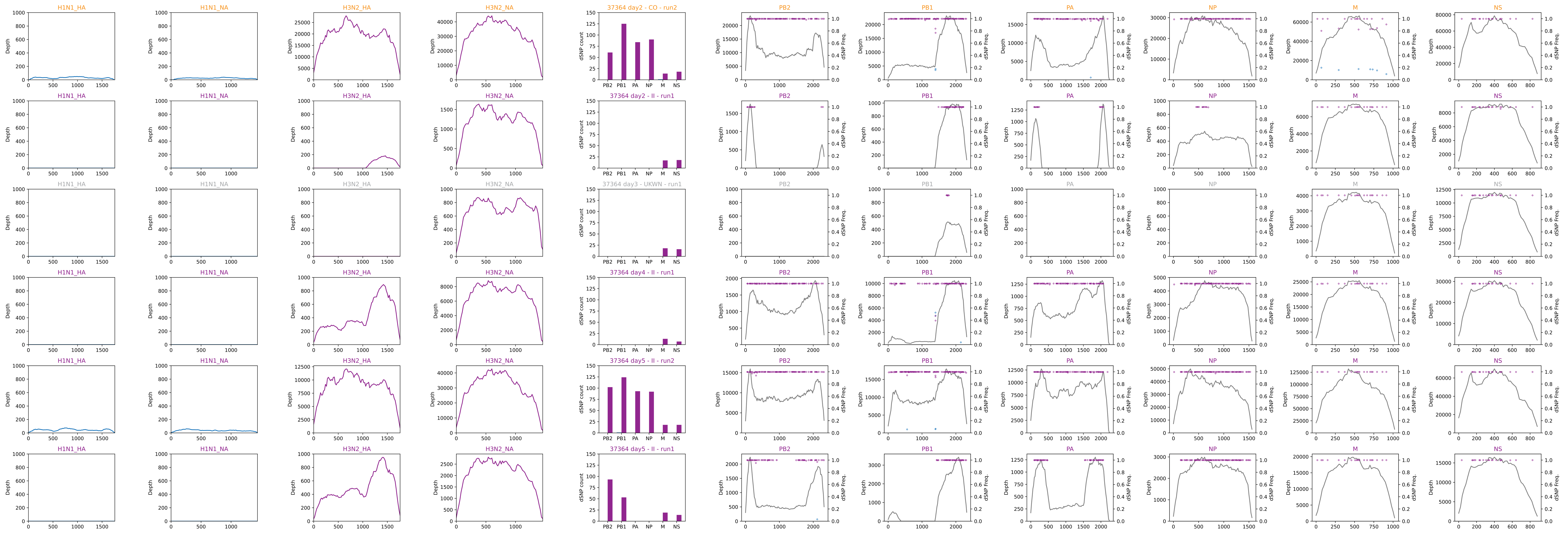
H1N1_HA
H1N1_NA
H3N2_HA
H3N2_NA
37364 day2 - CO - run2
PB2
PB1
PA
NP
M
NS
37364 day2 - II - run1
37364 day3 - UKWN - run1
37364 day4 - II - run1
37364 day5 - II - run2
37364 day5 - II - run1
Depth
dSNP count
dSNP Freq.

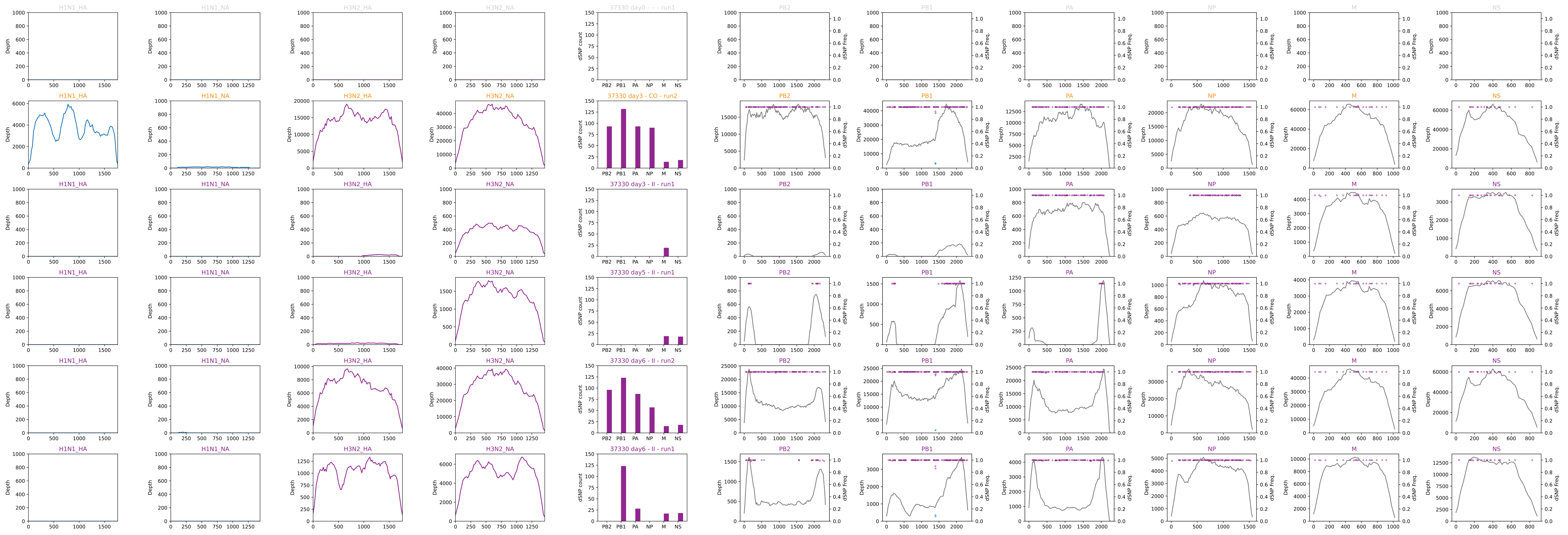

H1N1_HA
H1N1_NA
H3N2_HA
H3N2_NA
PB2
PB1
PA
NP
M
NS
37330 day0 - -- - run1
37330 day3 - CO - run2
37330 day3 - II - run1
37330 day5 - II - run1
37330 day6 - II - run2
37330 day6 - II - run1
Depth
dSNP count
dSNP Freq.

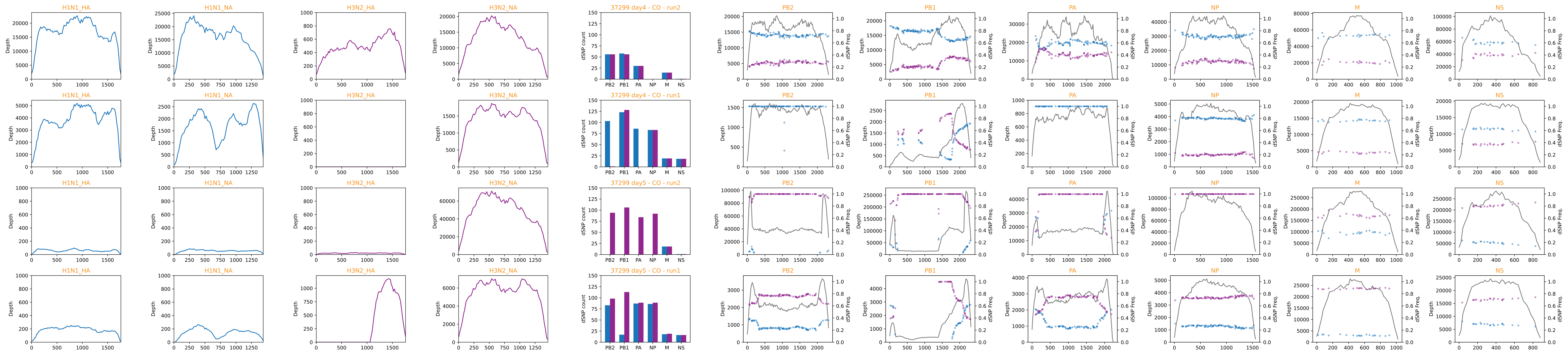
H1N1_HA
H1N1_NA
H3N2_HA
H3N2_NA
37299 day4 - CO - run2
PB2
PB1
PA
NP
M
NS
37299 day4 - CO - run1
37299 day5 - CO - run2
37299 day5 - CO - run1
Depth
dSNP count
dSNP Freq.

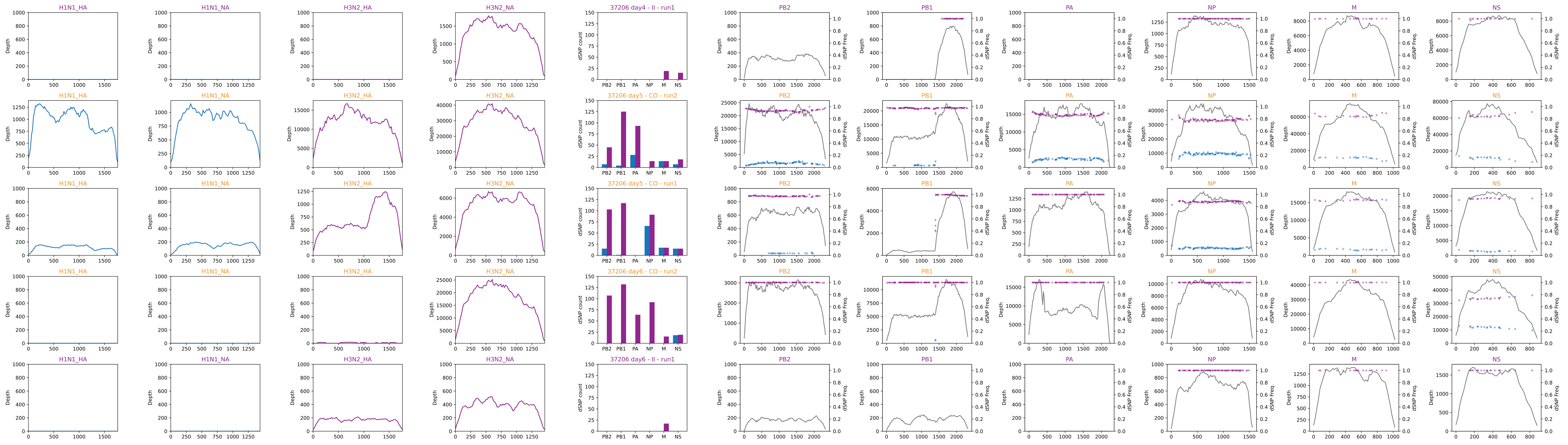
H1N1_HA
H1N1_NA
H3N2_HA
H3N2_NA
37206 day4 - II - run1
PB2
PB1
PA
NP
M
NS
37206 day5 - CO - run2
37206 day5 - CO - run1
37206 day6 - CO - run2
37206 day6 - II - run1
Depth
dSNP count
dSNP Freq.

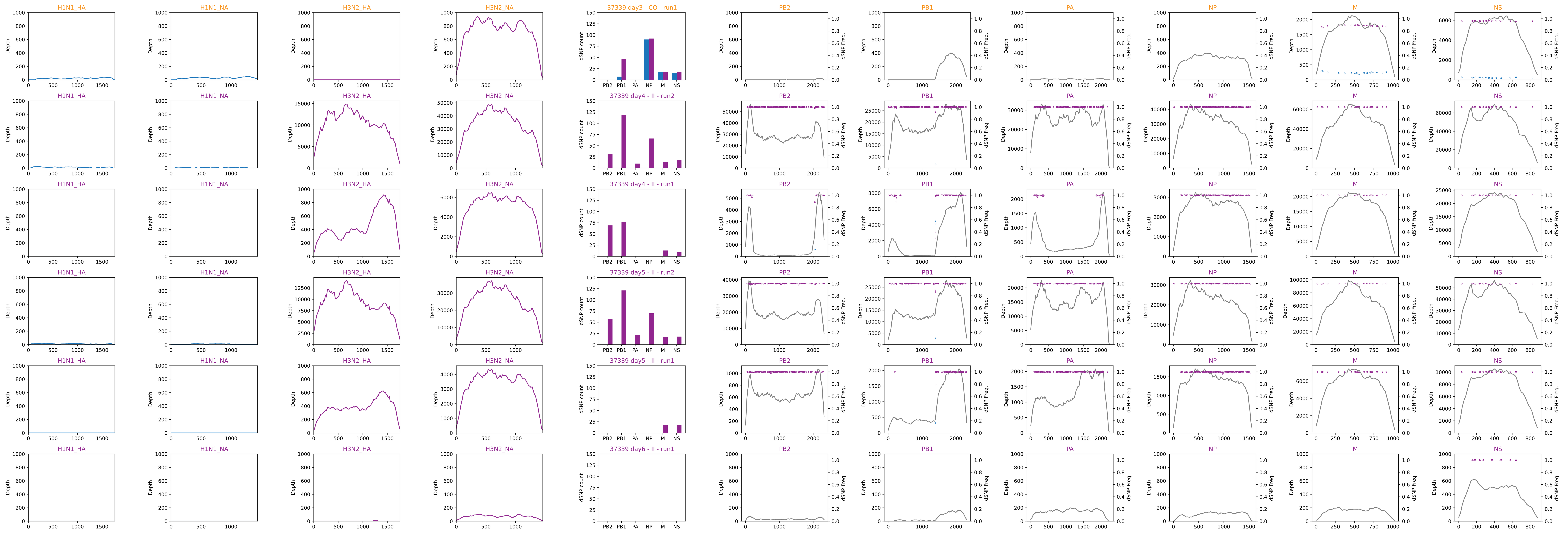
H1N1_HA
H1N1_NA
H3N2_HA
H3N2_NA
37339 day3 - CO - run1
PB2
PB1
PA
NP
M
NS
37339 day4 - II - run2
37339 day4 - II - run1
37339 day5 - II - run2
37339 day5 - II - run1
37339 day6 - II - run1
Depth
dSNP count
dSNP Freq.

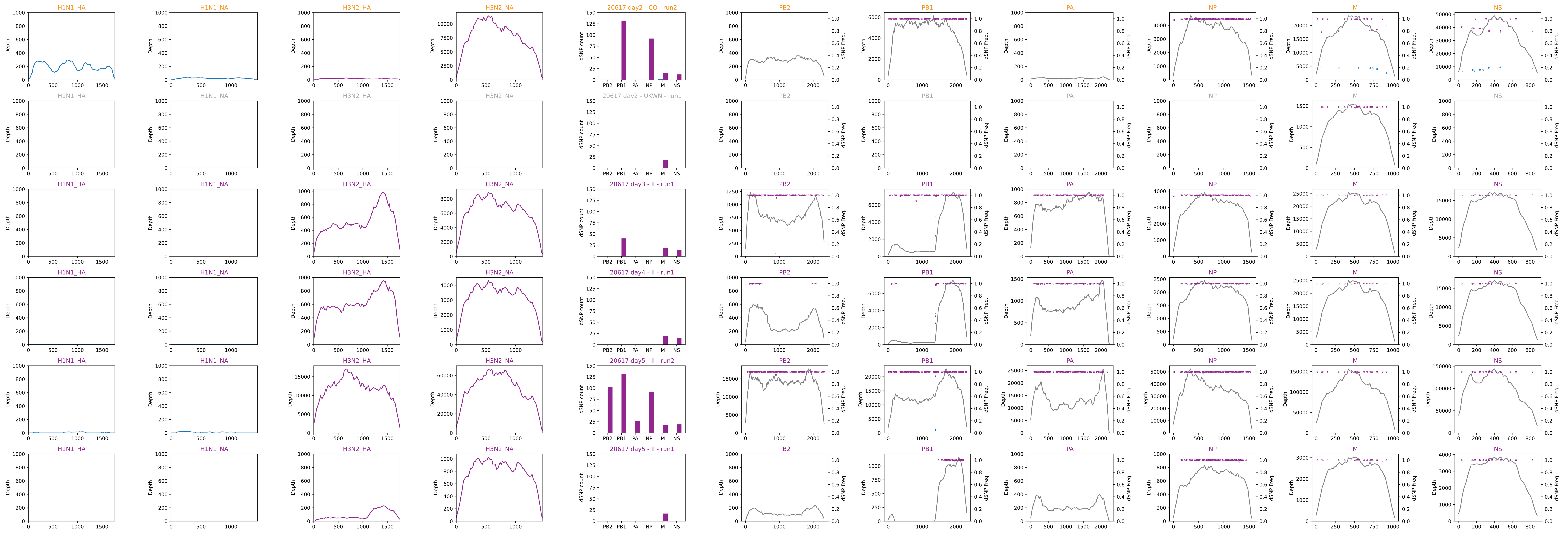
H1N1_HA
H1N1_NA
H3N2_HA
H3N2_NA
20617 day2 - CO - run2
20617 day2 - UKWN - run1
20617 day3 - II - run1
20617 day4 - II - run1
20617 day5 - II - run2
20617 day5 - II - run1
PB2
PB1
PA
NP
M
NS
Depth
dSNP count
dSNP Freq.

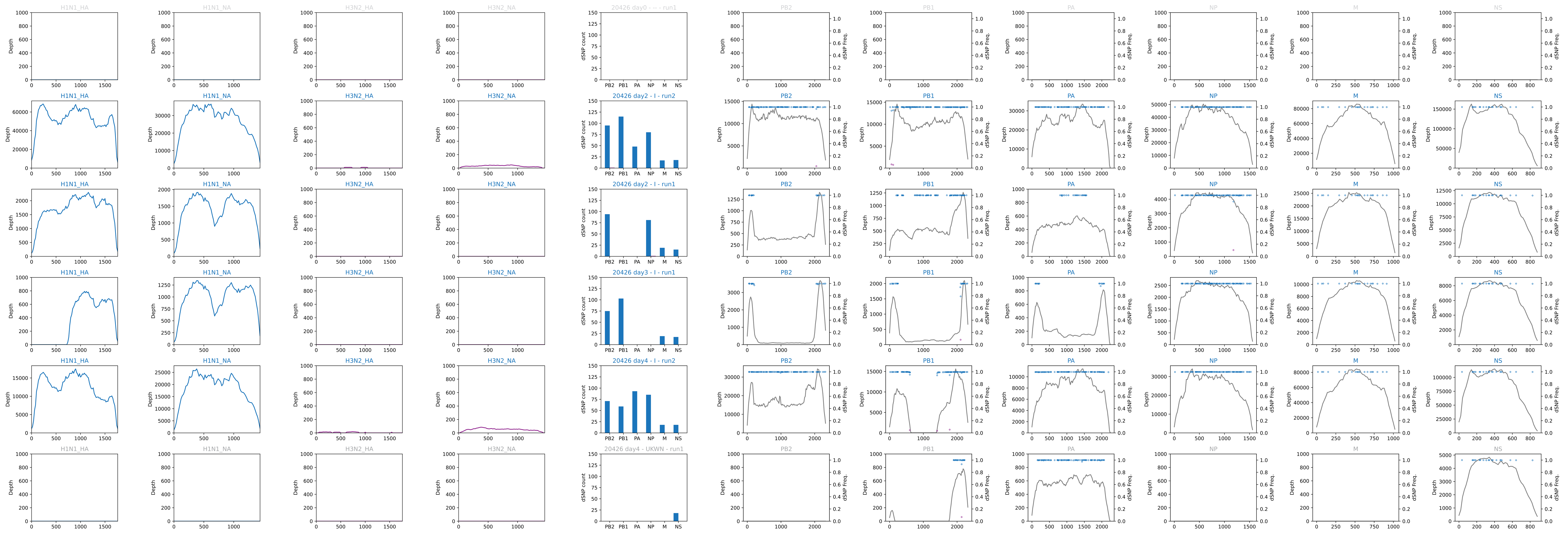
H1N1_HA
H1N1_NA
H3N2_HA
H3N2_NA
20426 day0 - -- - run1
PB2
PB1
PA
NP
M
NS
20426 day2 - I - run2
20426 day2 - I - run1
20426 day3 - I - run1
20426 day4 - I - run2
20426 day4 - UKWN - run1
Depth
dSNP count
dSNP Freq.

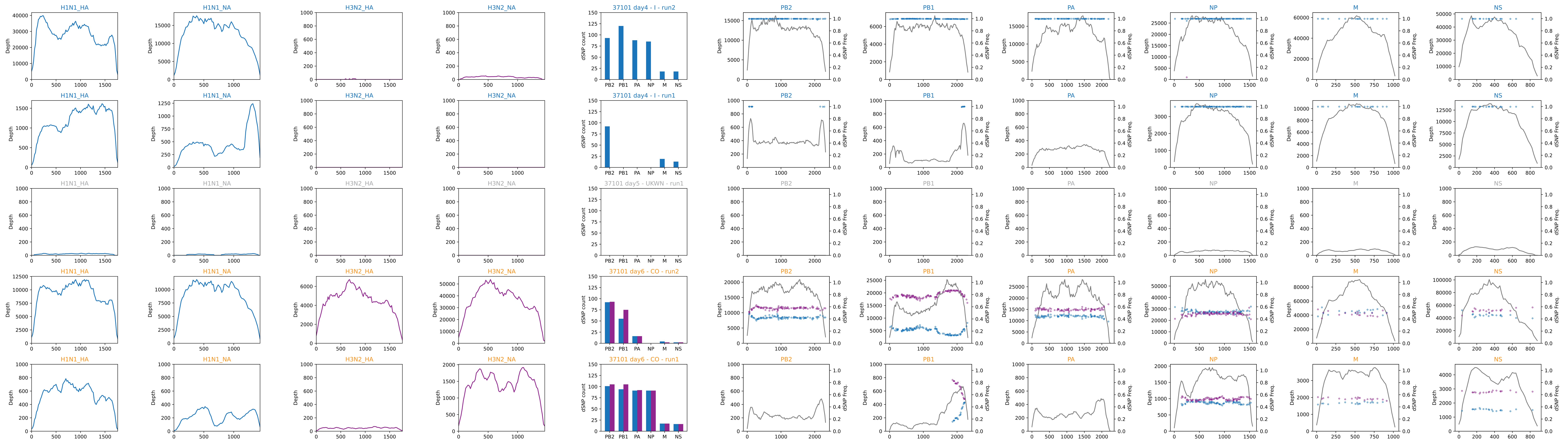

H1N1_HA
H1N1_NA
H3N2_HA
H3N2_NA
37101 day4 - I - run2
37101 day4 - I - run1
37101 day5 - UKWN - run1
37101 day6 - CO - run2
37101 day6 - CO - run1
PB2
PB1
PA
NP
M
NS
Depth
dSNP count
dSNP Freq.

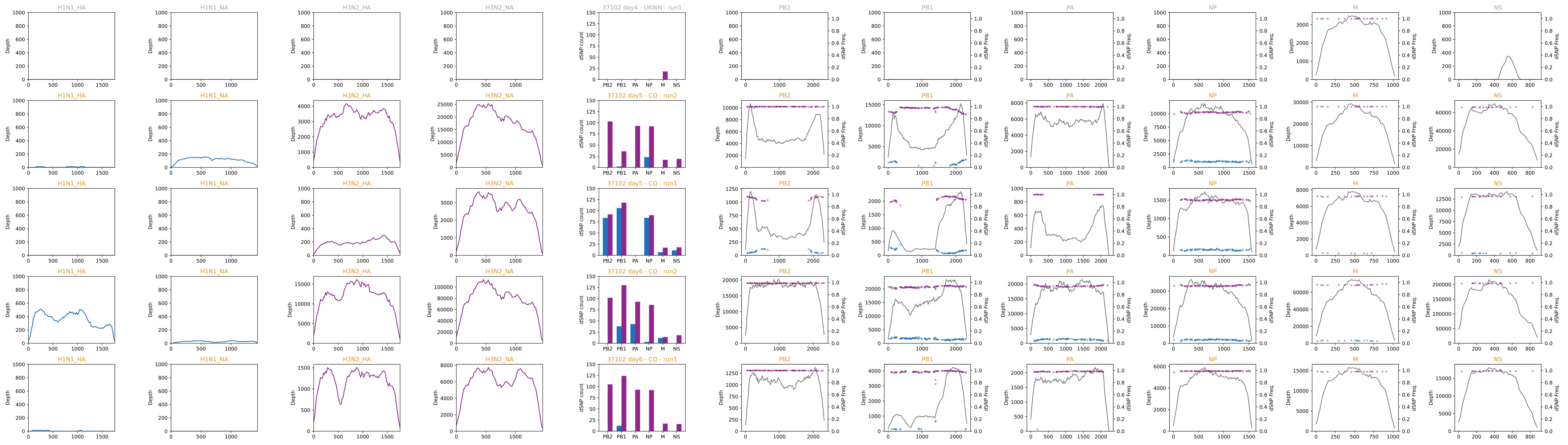

37102 day4 - UKWN - run1
37102 day5 - CO - run2
37102 day5 - CO - run1
37102 day6 - CO - run2
37102 day6 - CO - run1
H1N1_HA
H1N1_NA
H3N2_HA
H3N2_NA
PB2
PB1
PA
NP
M
NS
Depth
dSNP count
dSNP Freq.

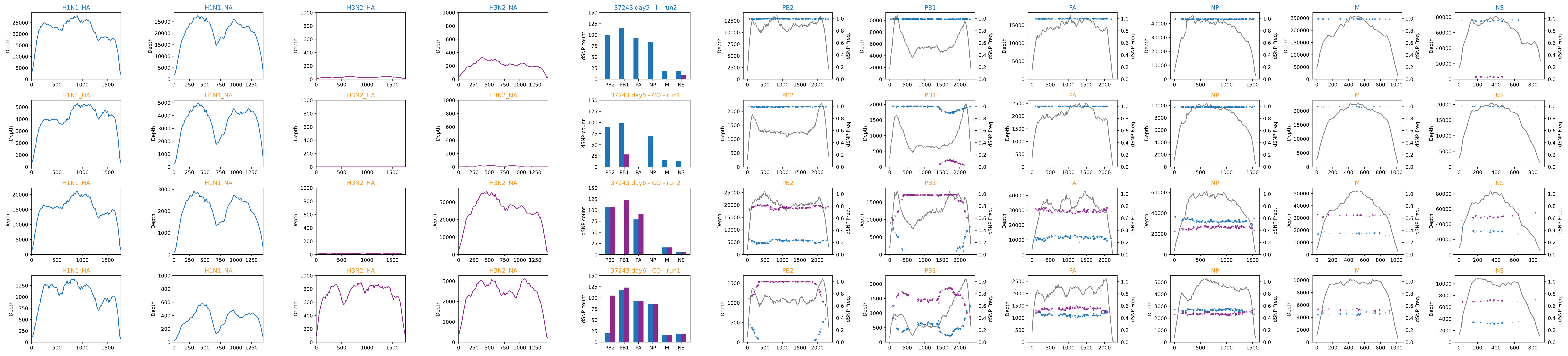

H1N1_HA
H1N1_NA
H3N2_HA
H3N2_NA
37243 day5 - I - run2
37243 day5 - CO - run1
37243 day6 - CO - run2
37243 day6 - CO - run1
PB2
PB1
PA
NP
M
NS
Depth
dSNP count
dSNP Freq.

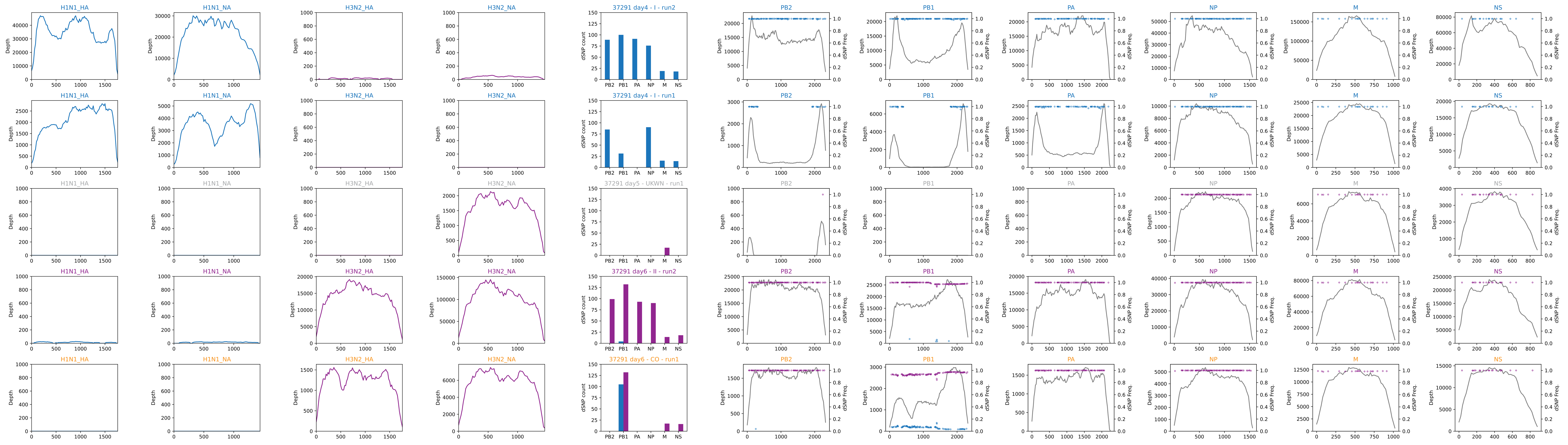
H1N1_HA
H1N1_NA
H3N2_HA
H3N2_NA
37291 day4 - I - run2
37291 day4 - I - run1
37291 day5 - UKWN - run1
37291 day6 - II - run2
37291 day6 - CO - run1
PB2
PB1
PA
NP
M
NS
Depth
dSNP count
dSNP Freq.

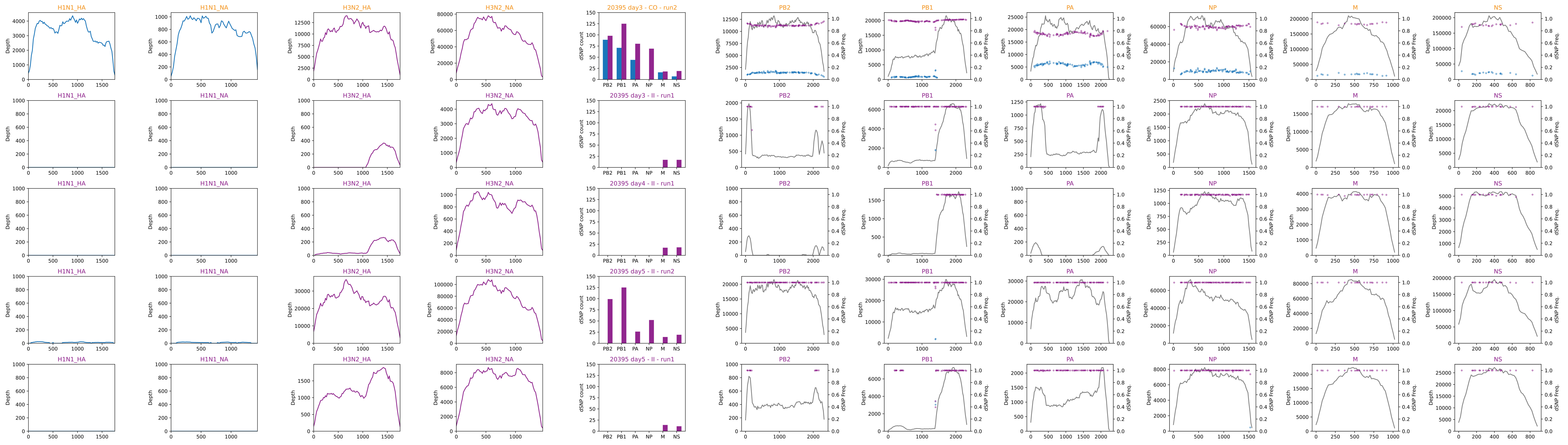

H1N1_HA
H1N1_NA
H3N2_HA
H3N2_NA
20395 day3 - CO - run2
PB2
PB1
PA
NP
M
NS
20395 day3 - II - run1
20395 day4 - II - run1
20395 day5 - II - run2
20395 day5 - II - run1
Depth
dSNP count
dSNP Freq.

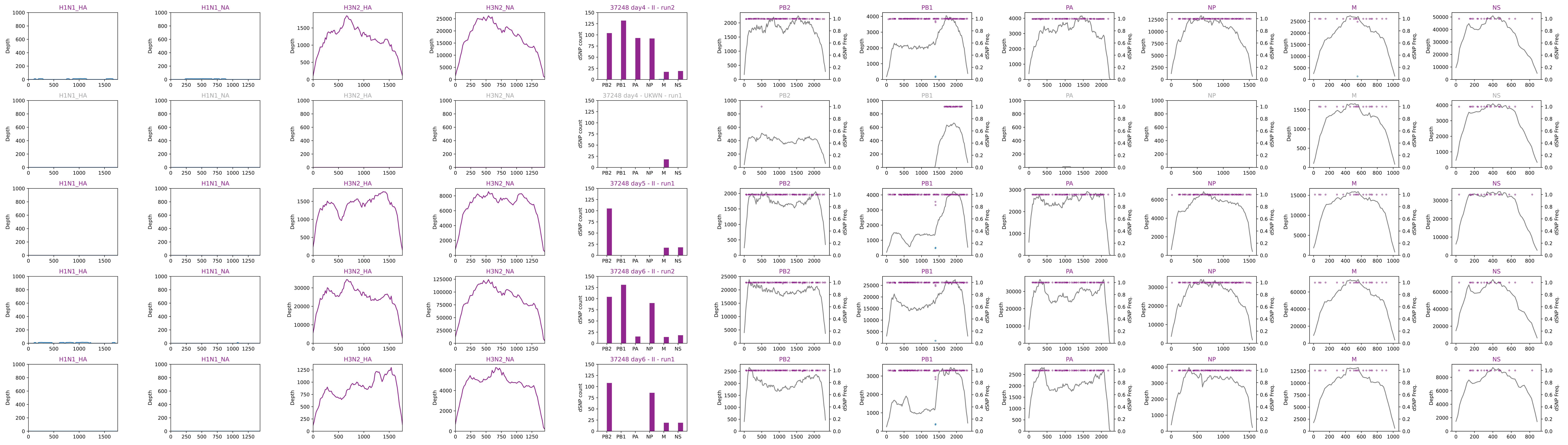

H1N1_HA
H1N1_NA
H3N2_HA
H3N2_NA
37248 day4 - II - run2
37248 day4 - UKWN - run1
37248 day5 - II - run1
37248 day6 - II - run2
37248 day6 - II - run1
PB2
PB1
PA
NP
M
NS
Depth
dSNP count
dSNP Freq.

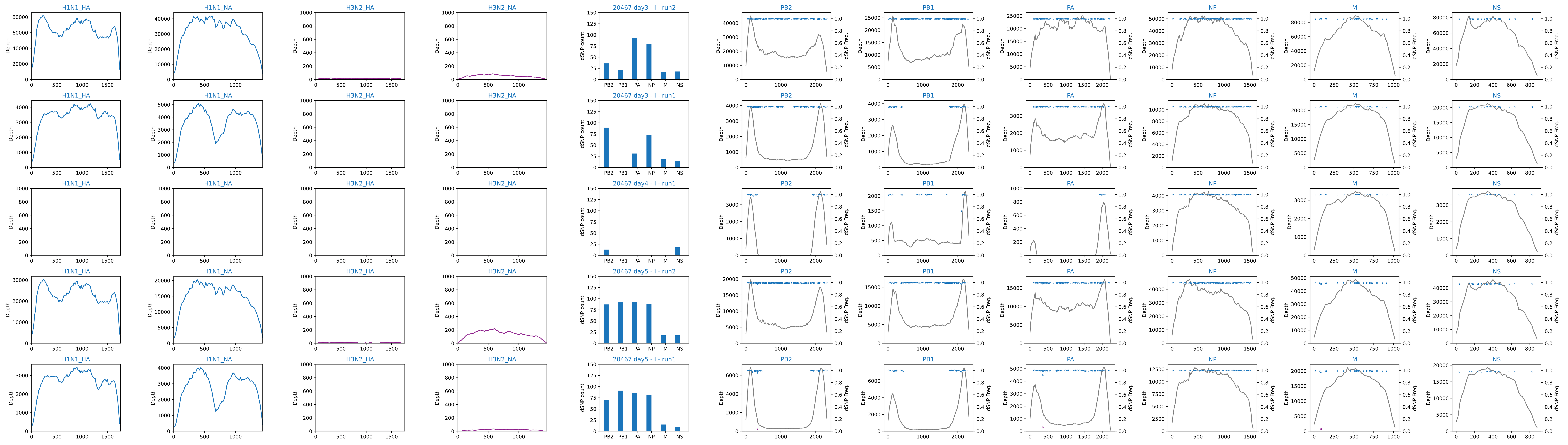

H1N1_HA
H1N1_NA
H3N2_HA
H3N2_NA
20467 day3 - I - run2
20467 day3 - I - run1
20467 day4 - I - run1
20467 day5 - I - run2
20467 day5 - I - run1
PB2
PB1
PA
NP
M
NS
Depth
dSNP count
dSNP Freq.

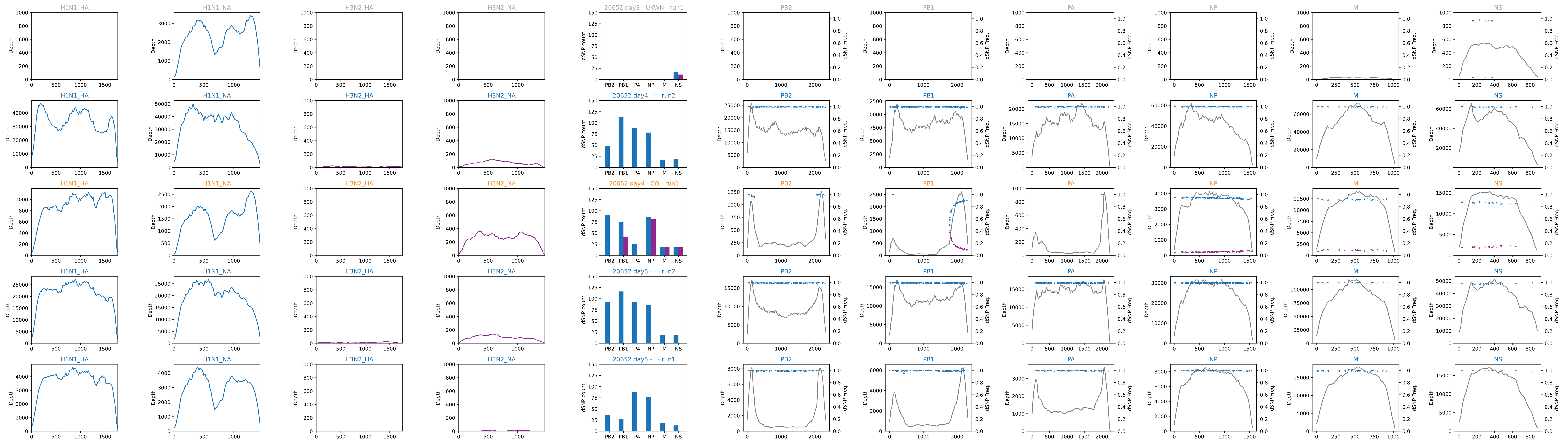
H1N1_HA
H1N1_NA
H3N2_HA
H3N2_NA
20652 day3 - UKWN - run1
PB2
PB1
PA
NP
M
NS
20652 day4 - I - run2
20652 day4 - CO - run1
20652 day5 - I - run2
20652 day5 - I - run1
Depth
dSNP count
dSNP Freq.

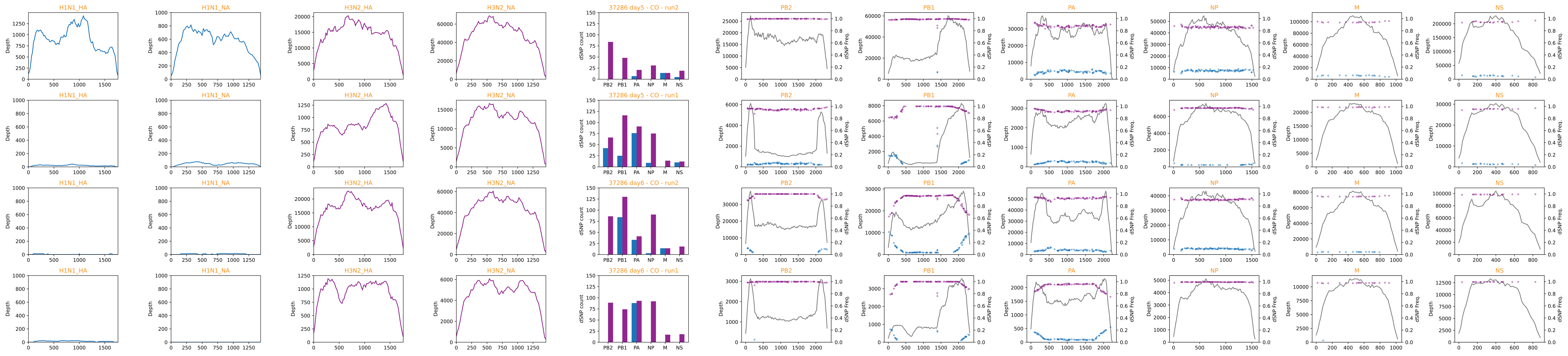
H1N1_HA
H1N1_NA
H3N2_HA
H3N2_NA
37286 day5 - CO - run2
PB2
PB1
PA
NP
M
NS
37286 day5 - CO - run1
37286 day6 - CO - run2
37286 day6 - CO - run1
Depth
dSNP count
dSNP Freq.

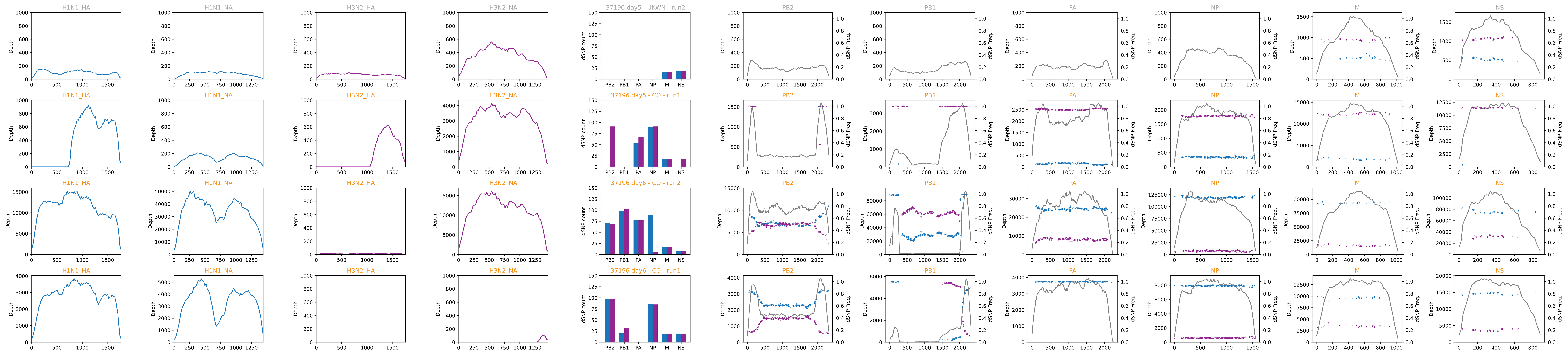

H1N1_HA
H1N1_NA
H3N2_HA
H3N2_NA
37196 day5 - UKWN - run2
37196 day5 - CO - run1
37196 day6 - CO - run2
37196 day6 - CO - run1
PB2
PB1
PA
NP
M
NS
Depth
dSNP count
dSNP Freq.

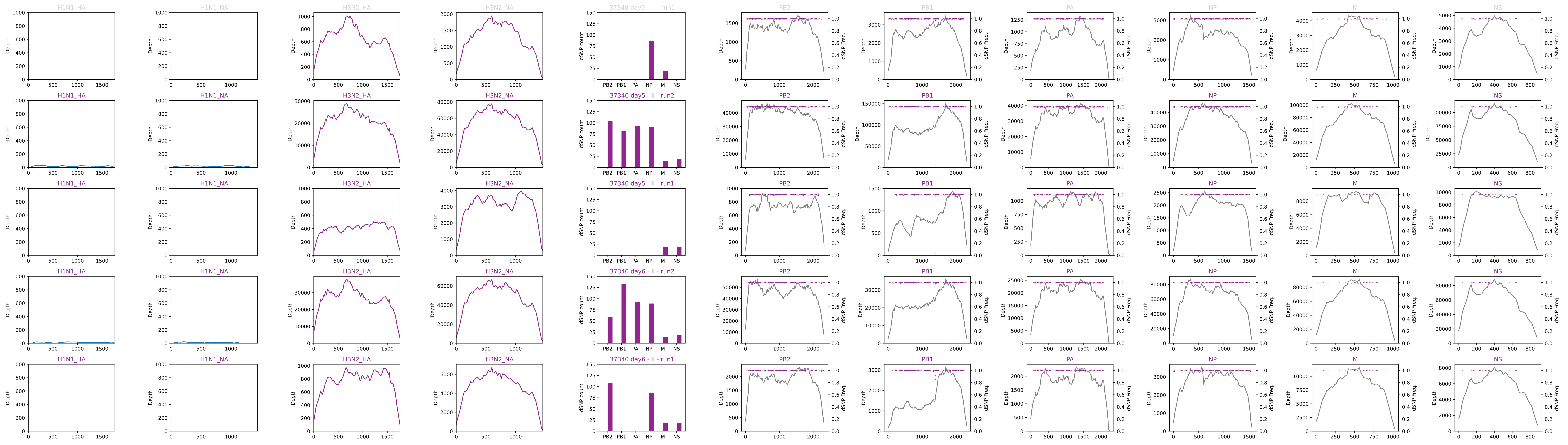
H1N1_HA
H1N1_NA
H3N2_HA
H3N2_NA
37340 day0 - -- - run1
37340 day5 - II - run2
37340 day5 - II - run1
37340 day6 - II - run2
37340 day6 - II - run1
PB2
PB1
PA
NP
M
NS
Depth
dSNP count
dSNP Freq.

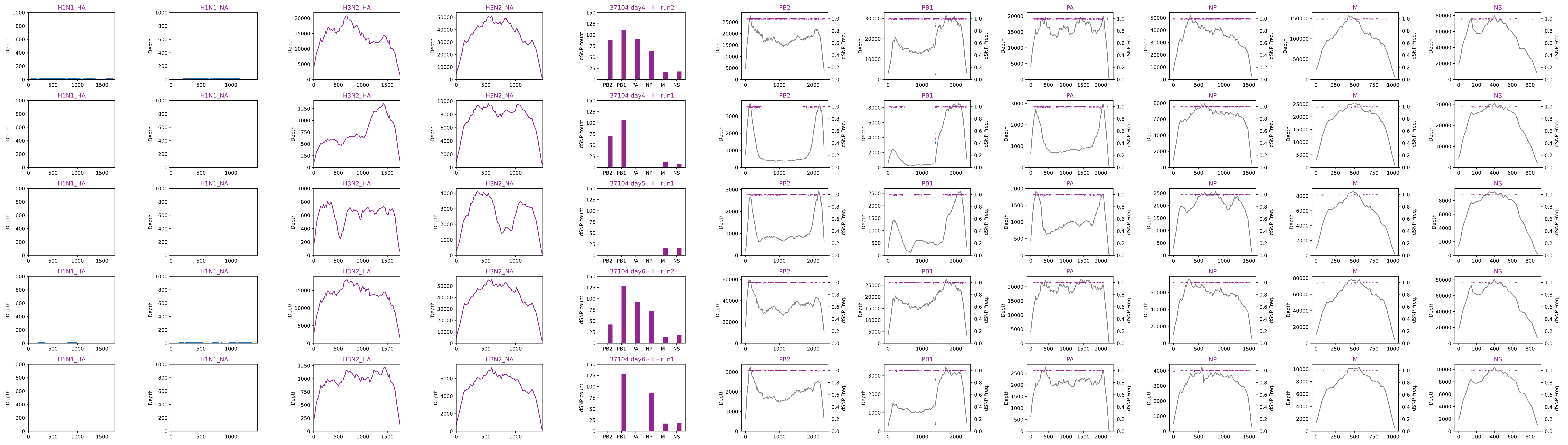
H1N1_HA
H1N1_NA
H3N2_HA
H3N2_NA
37104 day4 - II - run2
37104 day4 - II - run1
37104 day5 - II - run1
37104 day6 - II - run2
37104 day6 - II - run1
PB2
PB1
PA
NP
M
NS
Depth
dSNP count
dSNP Freq.

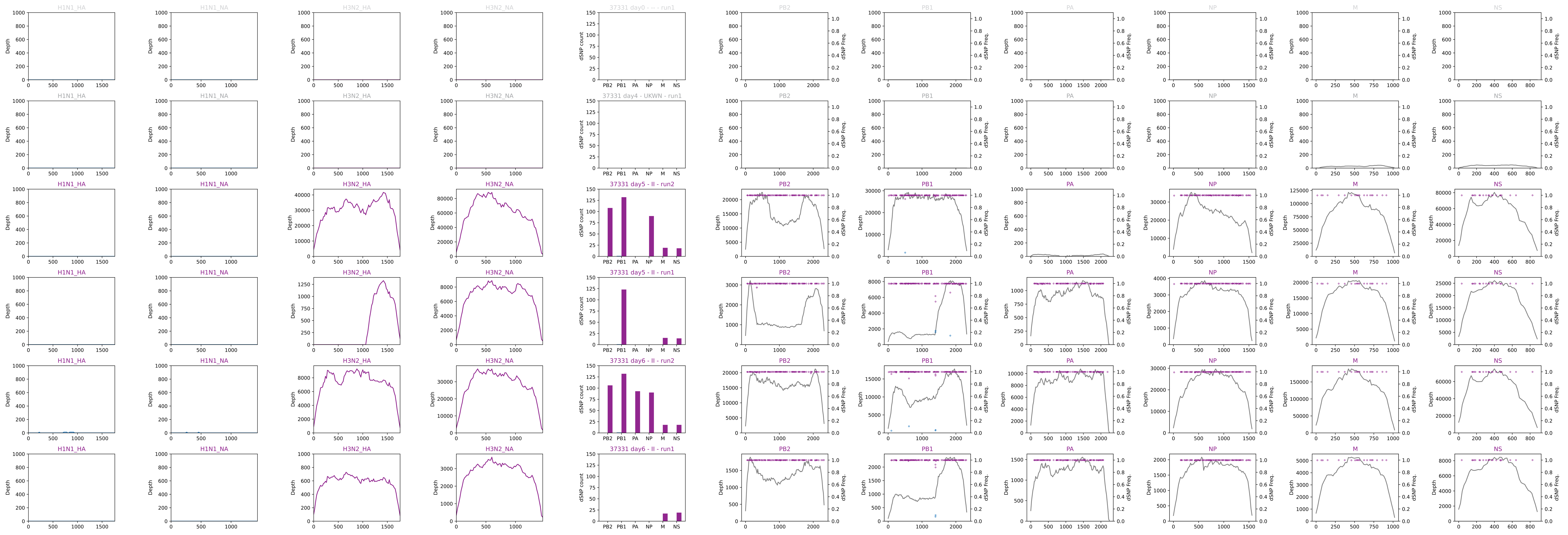

H1N1_HA
H1N1_NA
H3N2_HA
H3N2_NA
37331 day0 - -- - run1
PB2
PB1
PA
NP
M
NS
37331 day4 - UKWN - run1
37331 day5 - II - run2
37331 day5 - II - run1
37331 day6 - II - run2
37331 day6 - II - run1
Depth
dSNP count
dSNP Freq.

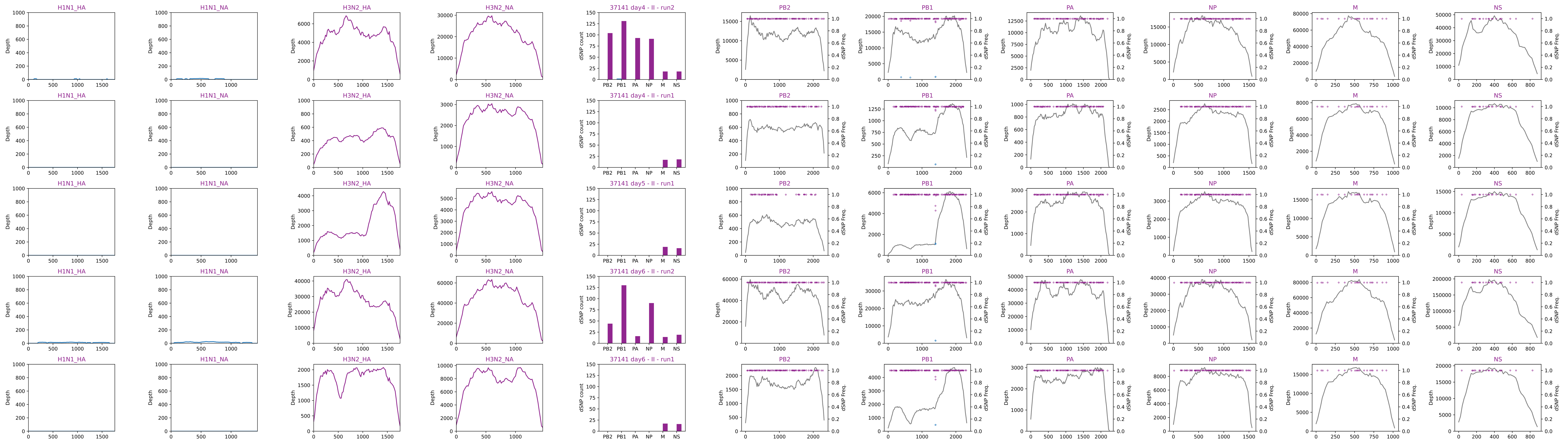
H1N1_HA
H1N1_NA
H3N2_HA
H3N2_NA
37141 day4 - II - run2
PB2
PB1
PA
NP
M
NS
37141 day4 - II - run1
37141 day5 - II - run1
37141 day6 - II - run2
37141 day6 - II - run1
Depth
dSNP count
dSNP Freq.

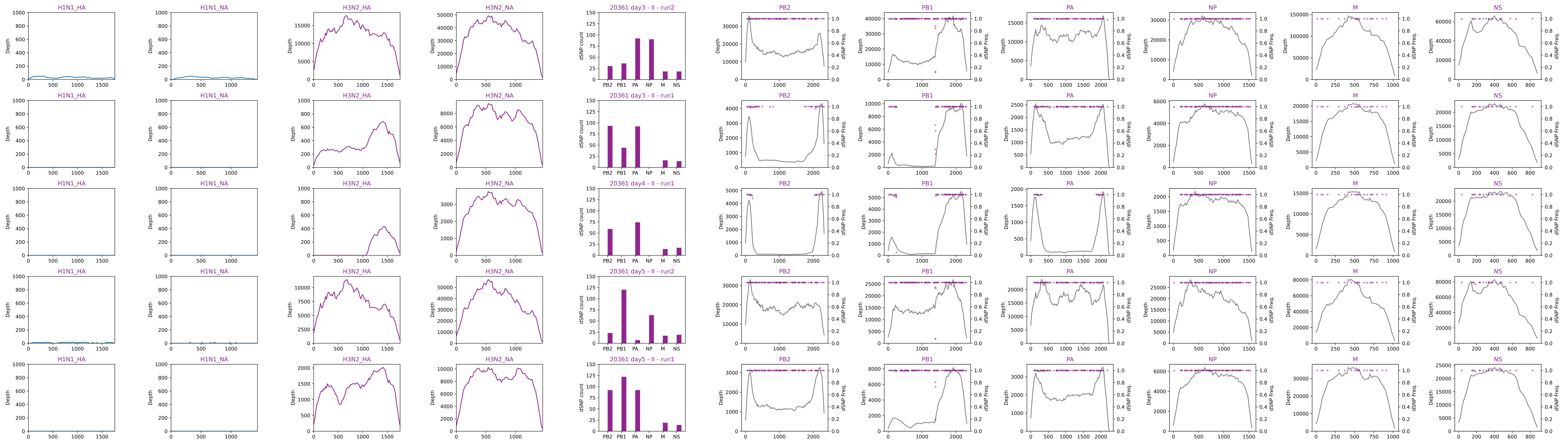

H1N1_HA
H1N1_NA
H3N2_HA
H3N2_NA
20361 day3 - II - run2
20361 day3 - II - run1
20361 day4 - II - run1
20361 day5 - II - run2
20361 day5 - II - run1
PB2
PB1
PA
NP
M
NS
Depth
dSNP count
dSNP Freq.

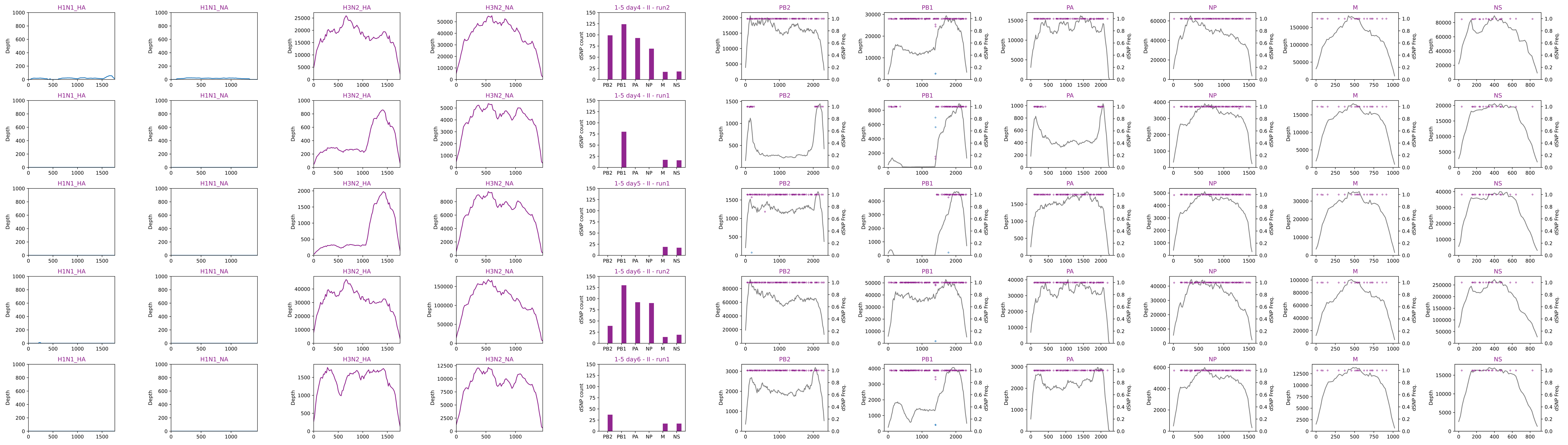
H1N1_HA
H1N1_NA
H3N2_HA
H3N2_NA
1-5 day4 - II - run2
1-5 day4 - II - run1
1-5 day5 - II - run1
1-5 day6 - II - run2
1-5 day6 - II - run1
PB2
PB1
PA
NP
M
NS
Depth
dSNP count
dSNP Freq.

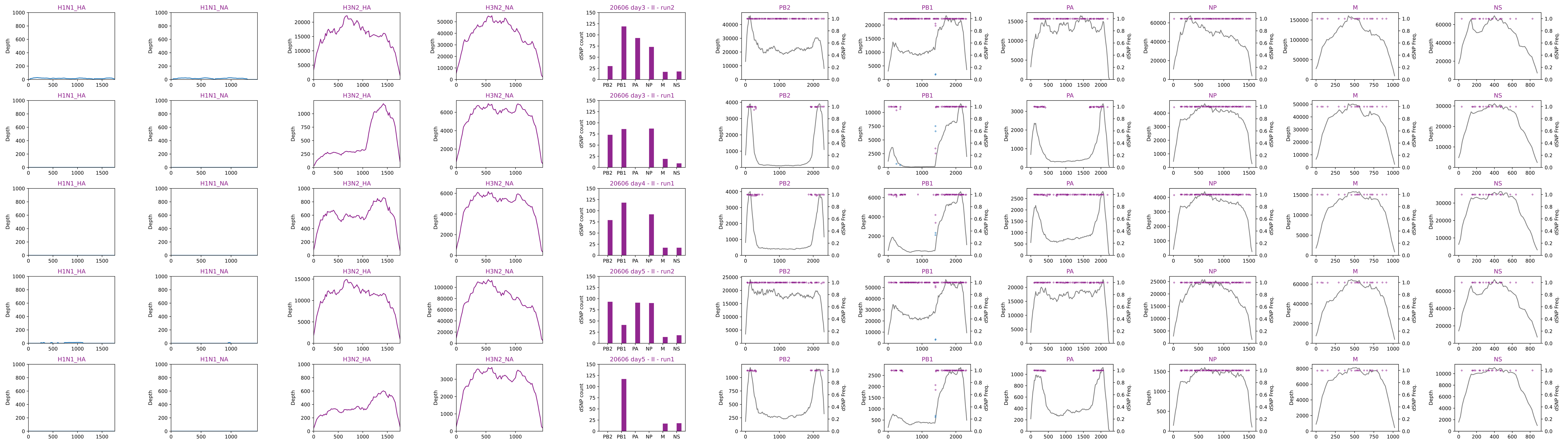
H1N1_HA
H1N1_NA
H3N2_HA
H3N2_NA
20606 day3 - II - run2
PB2
PB1
PA
NP
M
NS
20606 day3 - II - run1
20606 day4 - II - run1
20606 day5 - II - run2
20606 day5 - II - run1
Depth
dSNP count
dSNP Freq.

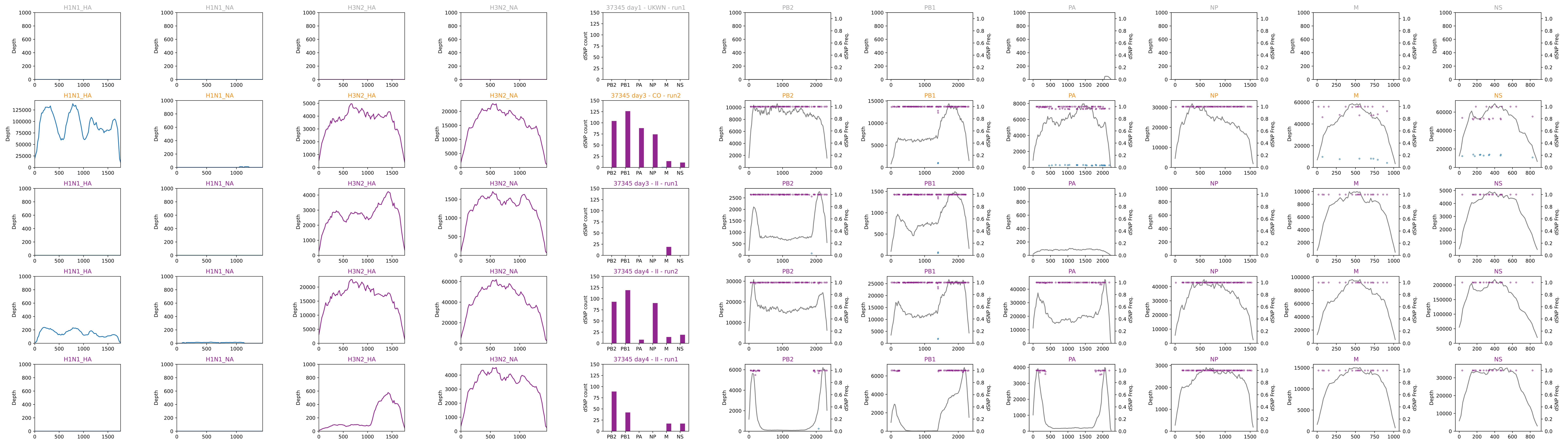

37345 day1 - UKWN - run1
37345 day3 - CO - run2
37345 day3 - II - run1
37345 day4 - II - run2
37345 day4 - II - run1
H1N1_HA
H1N1_NA
H3N2_HA
H3N2_NA
PB2
PB1
PA
NP
M
NS
Depth
dSNP count
dSNP Freq.

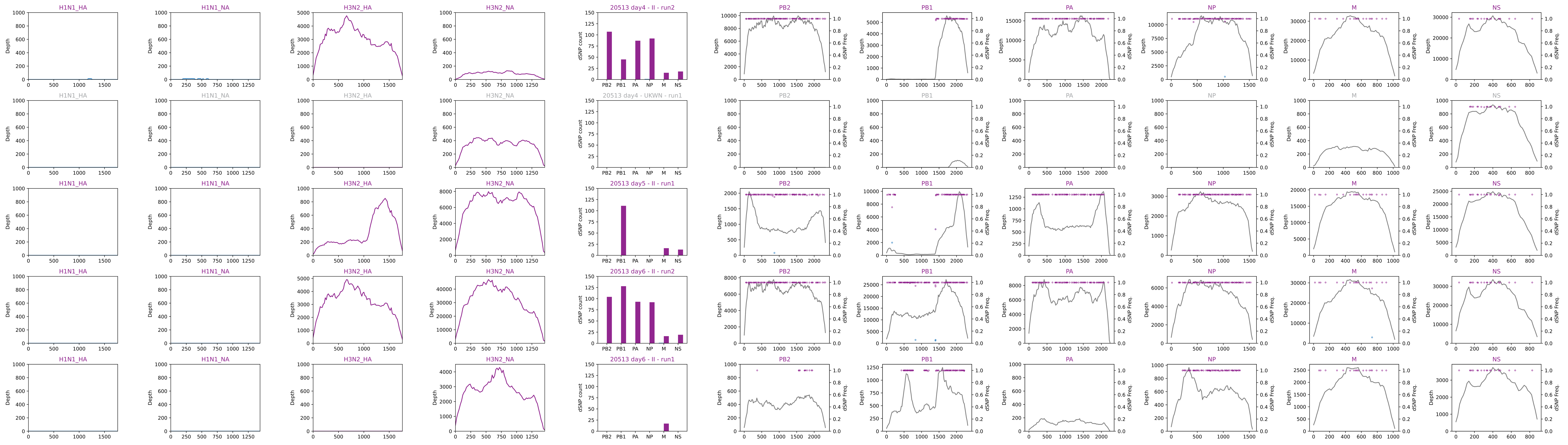
H1N1_HA
H1N1_NA
H3N2_HA
H3N2_NA
20513 day4 - II - run2
PB2
PB1
PA
NP
M
NS
20513 day4 - UKWN - run1
20513 day5 - II - run1
20513 day6 - II - run2
20513 day6 - II - run1
Depth
dSNP count
dSNP Freq.

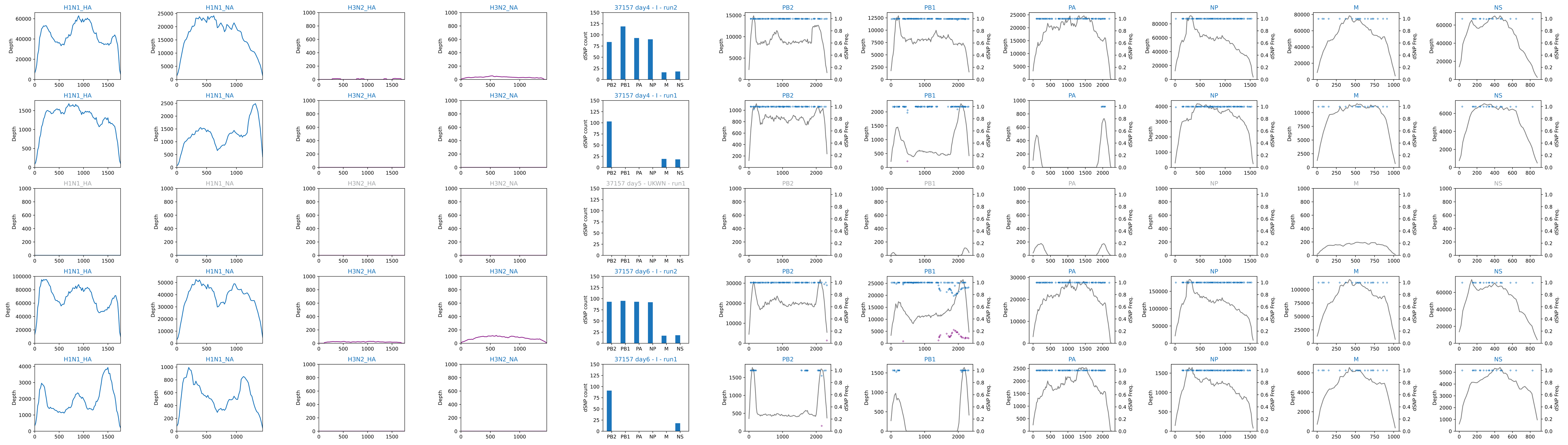

H1N1_HA
H1N1_NA
H3N2_HA
H3N2_NA
37157 day4 - I - run2
37157 day4 - I - run1
37157 day5 - UKWN - run1
37157 day6 - I - run2
37157 day6 - I - run1
PB2
PB1
PA
NP
M
NS
Depth
dSNP count
dSNP Freq.

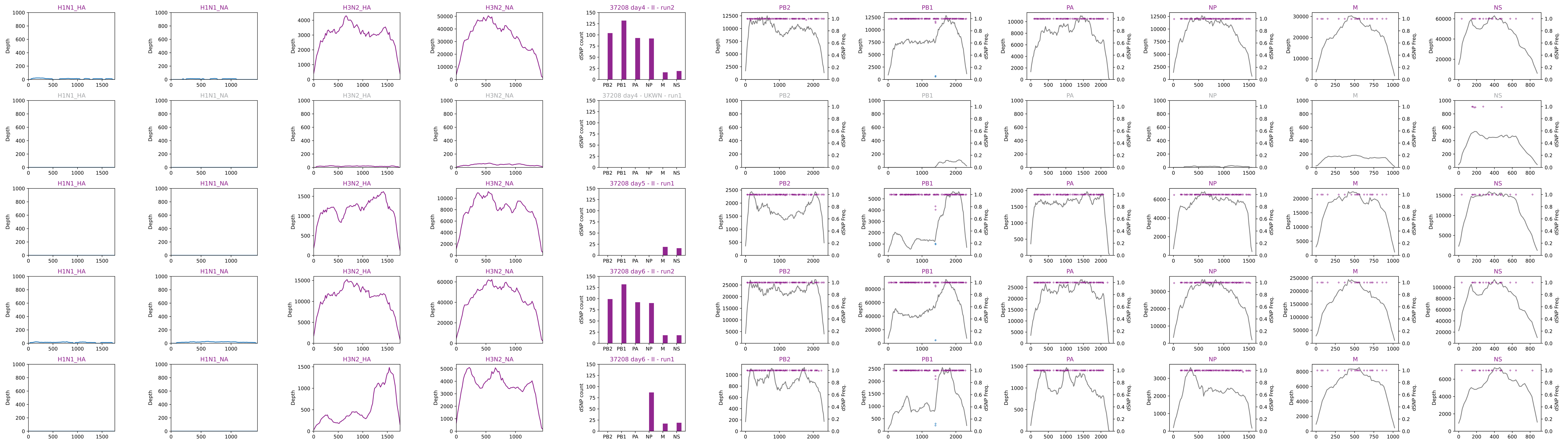

H1N1_HA
H1N1_NA
H3N2_HA
H3N2_NA
37208 day4 - II - run2
37208 day4 - UKWN - run1
37208 day5 - II - run1
37208 day6 - II - run2
37208 day6 - II - run1
PB2
PB1
PA
NP
M
NS
Depth
dSNP count
dSNP Freq.

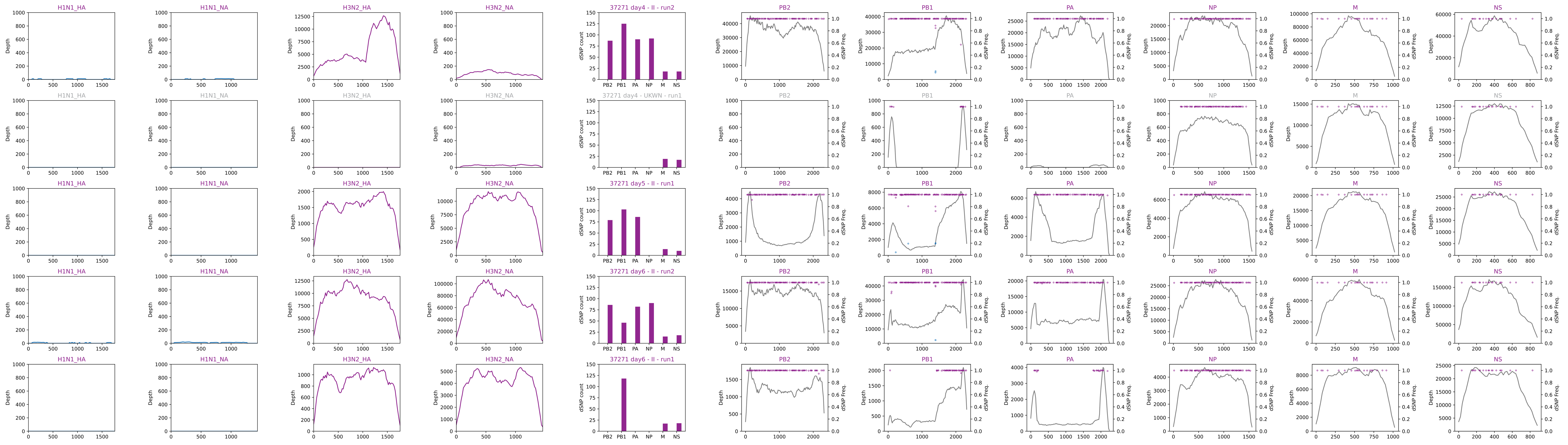
H1N1_HA
H1N1_NA
H3N2_HA
H3N2_NA
37271 day4 - II - run2
37271 day4 - UKWN - run1
37271 day5 - II - run1
37271 day6 - II - run2
37271 day6 - II - run1
PB2
PB1
PA
NP
M
NS
Depth
dSNP count
dSNP Freq.

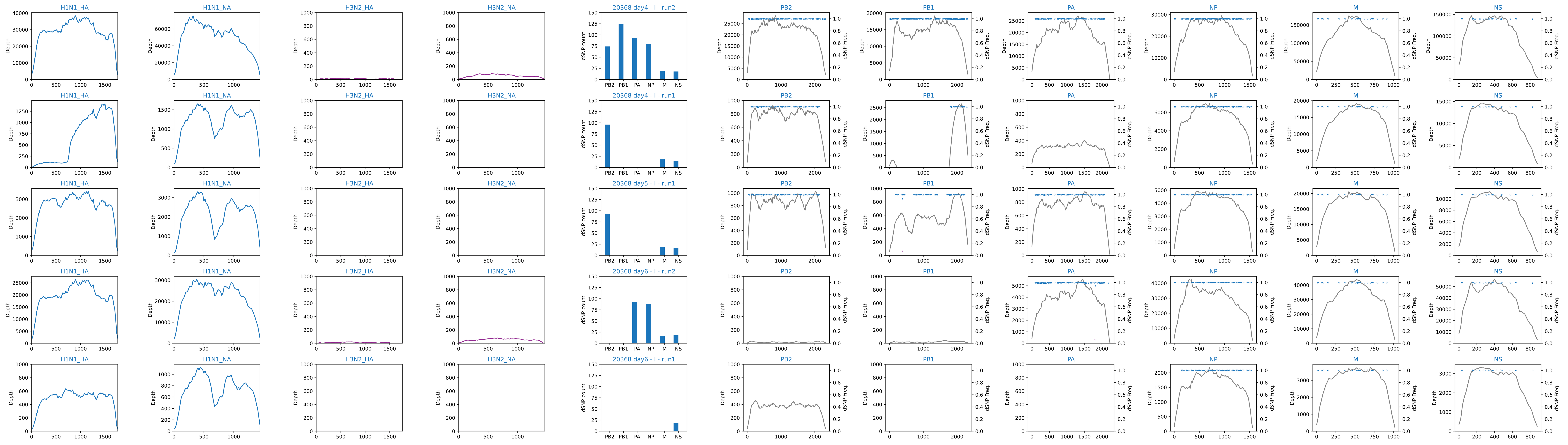

H1N1_HA
H1N1_NA
H3N2_HA
H3N2_NA
20368 day4 - I - run2
20368 day4 - I - run1
20368 day5 - I - run1
20368 day6 - I - run2
20368 day6 - I - run1
PB2
PB1
PA
NP
M
NS
Depth
dSNP count
dSNP Freq.

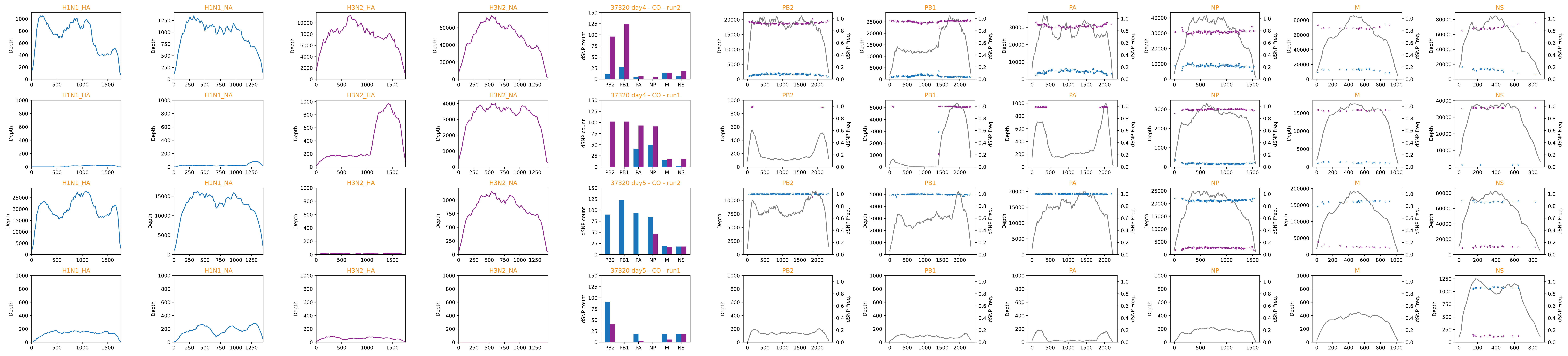
H1N1_HA
H1N1_NA
H3N2_HA
H3N2_NA
37320 day4 - CO - run2
37320 day4 - CO - run1
37320 day5 - CO - run2
37320 day5 - CO - run1
PB2
PB1
PA
NP
M
NS
Depth
dSNP count
dSNP Freq.

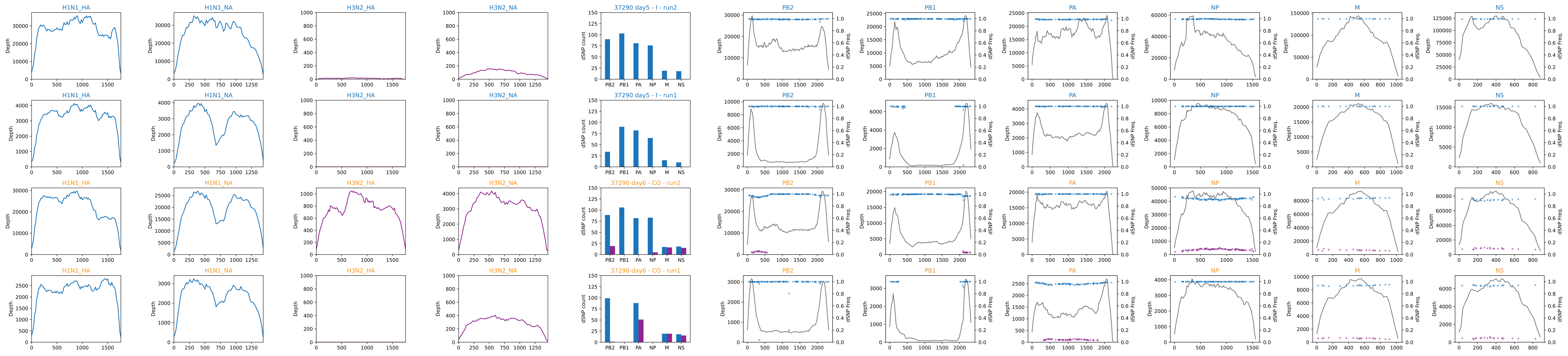
H1N1_HA
H1N1_NA
H3N2_HA
H3N2_NA
37290 day5 - I - run2
37290 day5 - I - run1
37290 day6 - CO - run2
37290 day6 - CO - run1
PB2
PB1
PA
NP
M
NS
Depth
dSNP count
dSNP Freq.

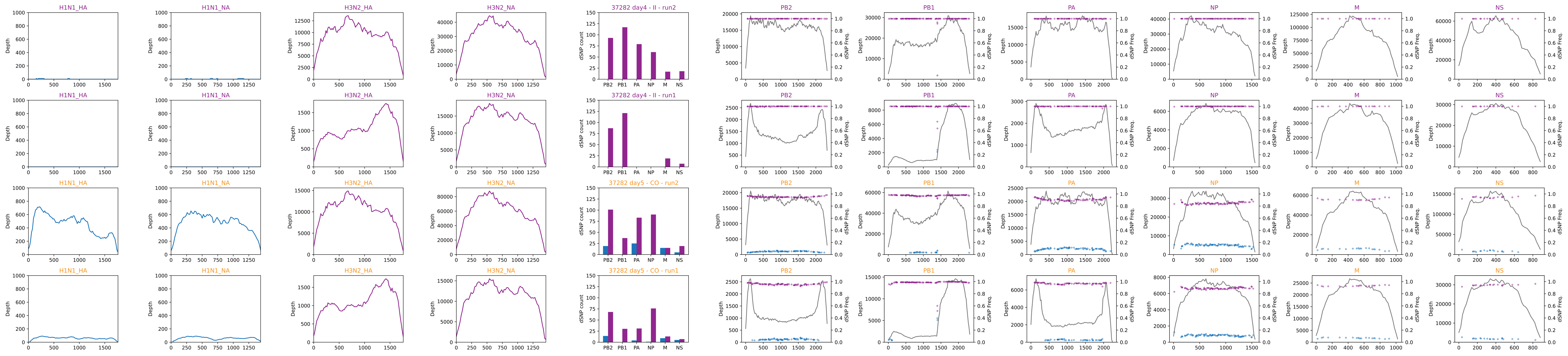
H1N1_HA
H1N1_NA
H3N2_HA
H3N2_NA
37282 day4 - II - run2
PB2
PB1
PA
NP
M
NS
37282 day4 - II - run1
37282 day5 - CO - run2
37282 day5 - CO - run1
Depth
dSNP count
dSNP Freq.

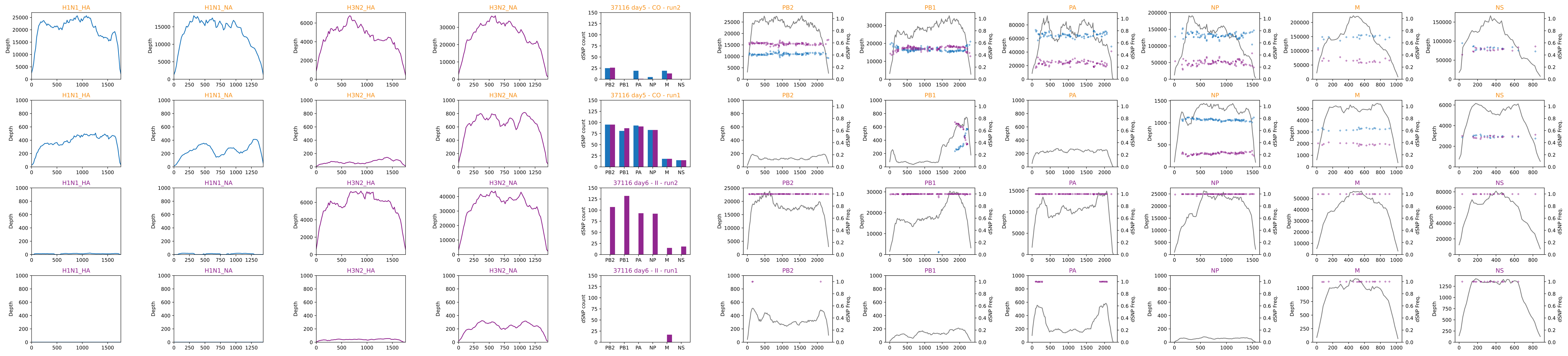

H1N1_HA
H1N1_NA
H3N2_HA
H3N2_NA
37116 day5 - CO - run2
37116 day5 - CO - run1
37116 day6 - II - run2
37116 day6 - II - run1
PB2
PB1
PA
NP
M
NS
Depth
dSNP count
dSNP Freq.

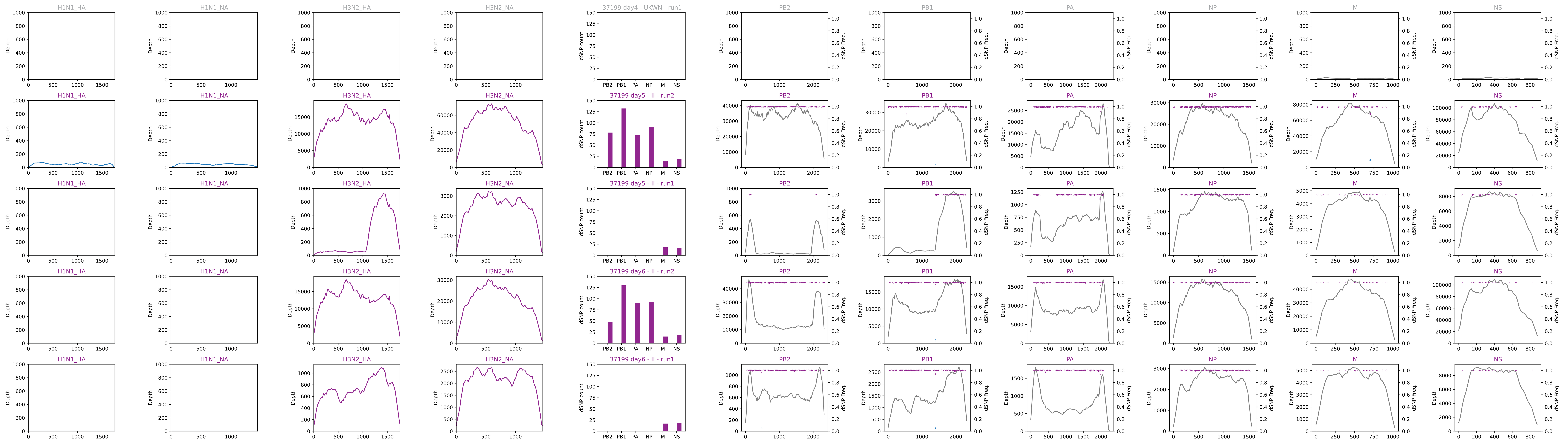

H1N1_HA
H1N1_NA
H3N2_HA
H3N2_NA
37199 day4 - UKWN - run1
37199 day5 - II - run2
37199 day5 - II - run1
37199 day6 - II - run2
37199 day6 - II - run1
PB2
PB1
PA
NP
M
NS
Depth
dSNP count
dSNP Freq.

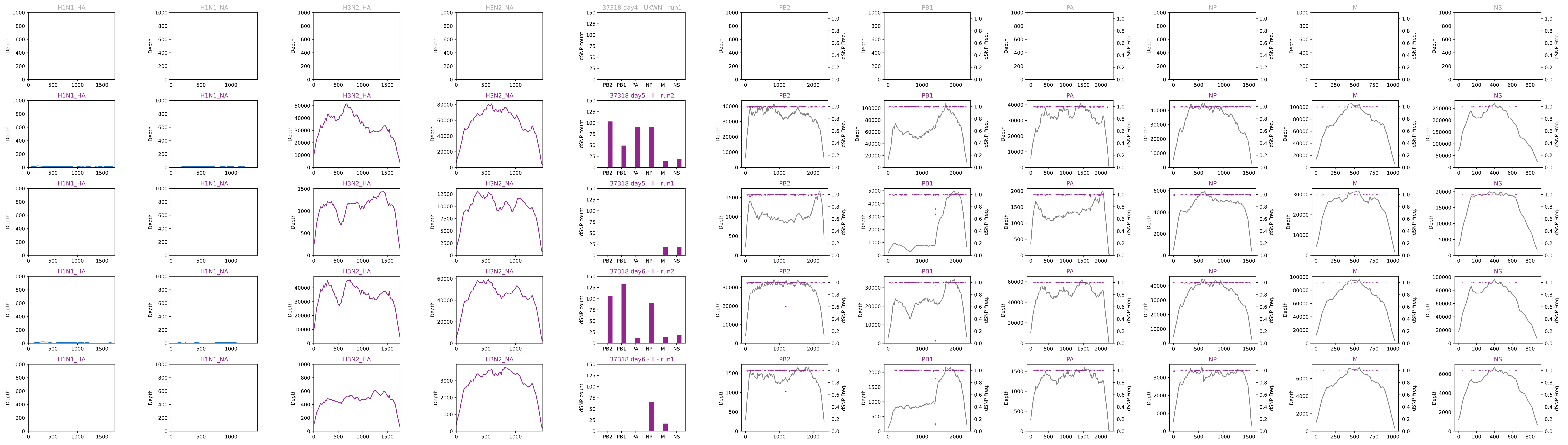

H1N1_HA
H1N1_NA
H3N2_HA
H3N2_NA
37318 day4 - UKWN - run1
37318 day5 - II - run2
37318 day5 - II - run1
37318 day6 - II - run2
37318 day6 - II - run1
PB2
PB1
PA
NP
M
NS
Depth
dSNP count
dSNP Freq.

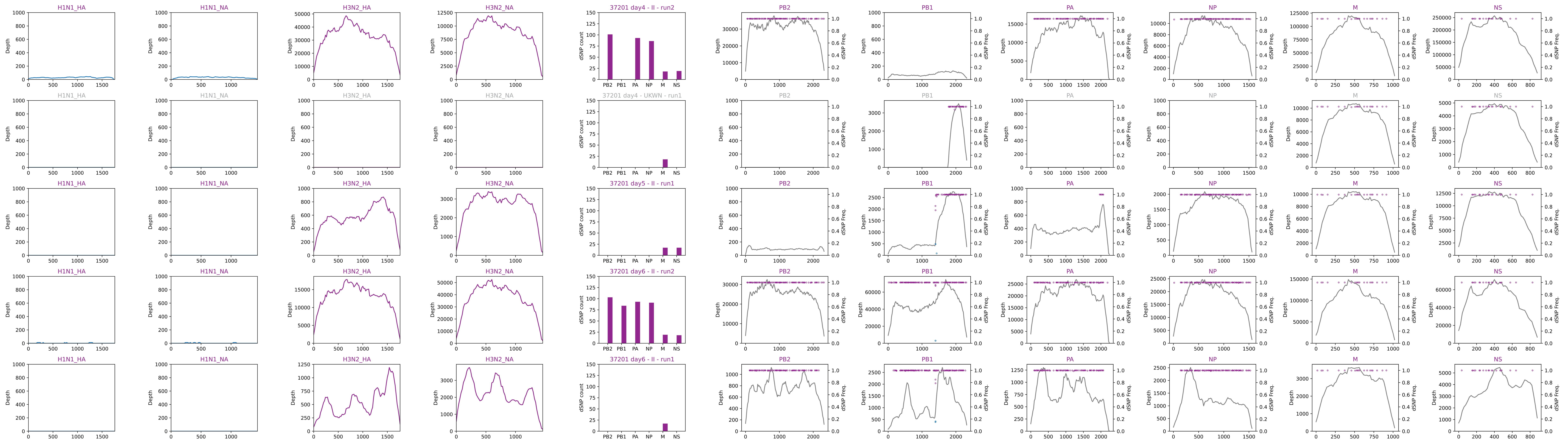
H1N1_HA
H1N1_NA
H3N2_HA
H3N2_NA
37201 day4 - II - run2
37201 day4 - UKWN - run1
37201 day5 - II - run1
37201 day6 - II - run2
37201 day6 - II - run1
PB2
PB1
PA
NP
M
NS
Depth
dSNP count
dSNP Freq.

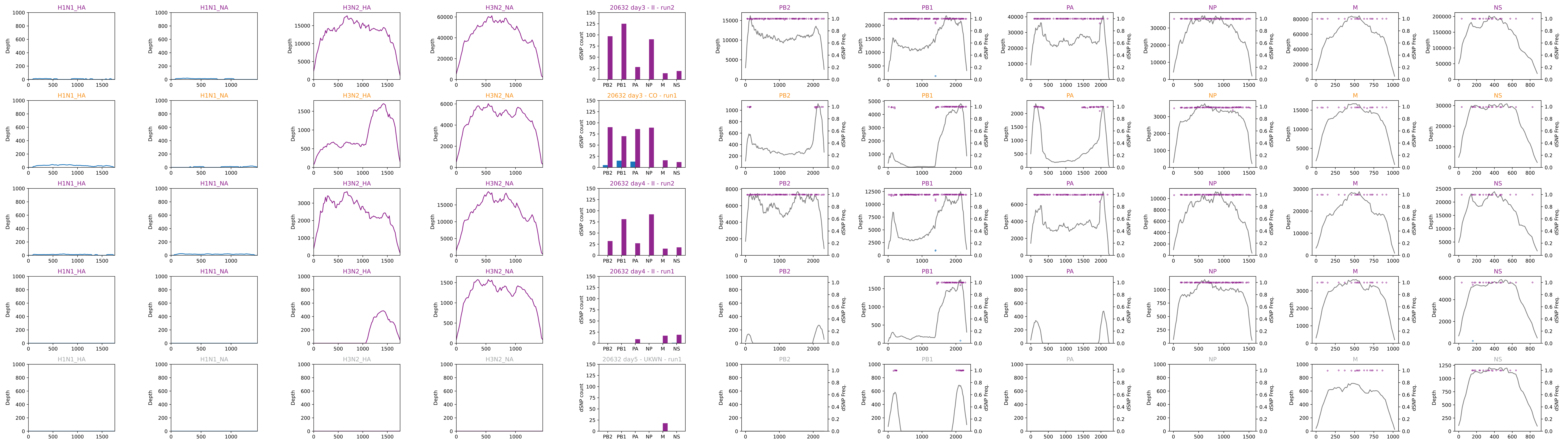

H1N1_HA
H1N1_NA
H3N2_HA
H3N2_NA
20632 day3 - II - run2
PB2
PB1
PA
NP
M
NS
20632 day3 - CO - run1
20632 day4 - II - run2
20632 day4 - II - run1
20632 day5 - UKWN - run1
Depth
dSNP count
dSNP Freq.

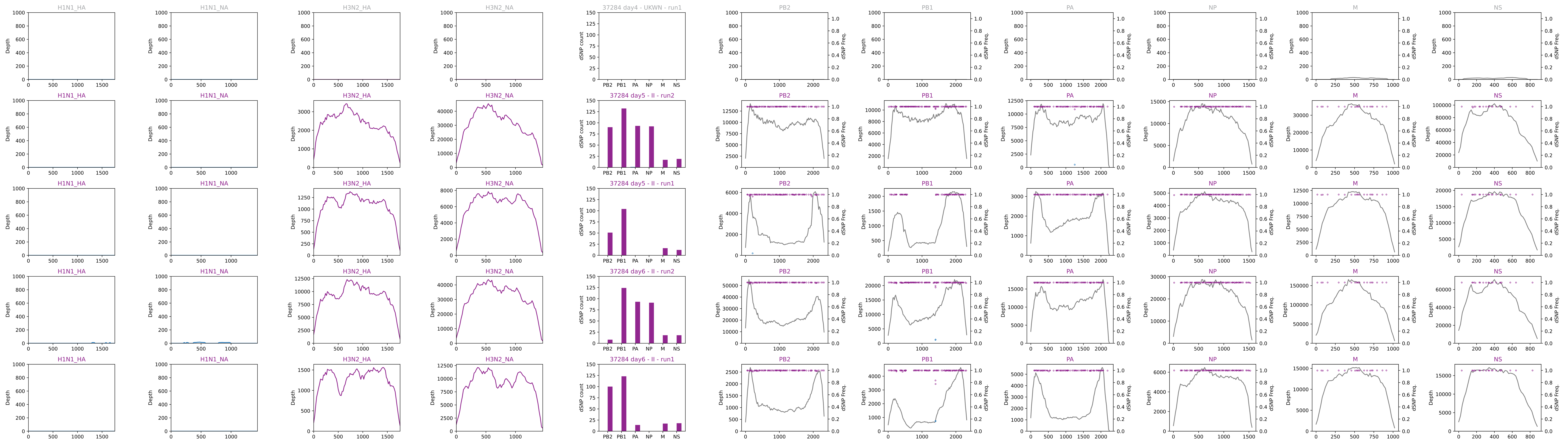

H1N1_HA
H1N1_NA
H3N2_HA
H3N2_NA
37284 day4 - UKWN - run1
PB2
PB1
PA
NP
M
NS
37284 day5 - II - run2
37284 day5 - II - run1
37284 day6 - II - run2
37284 day6 - II - run1
Depth
dSNP count
dSNP Freq.

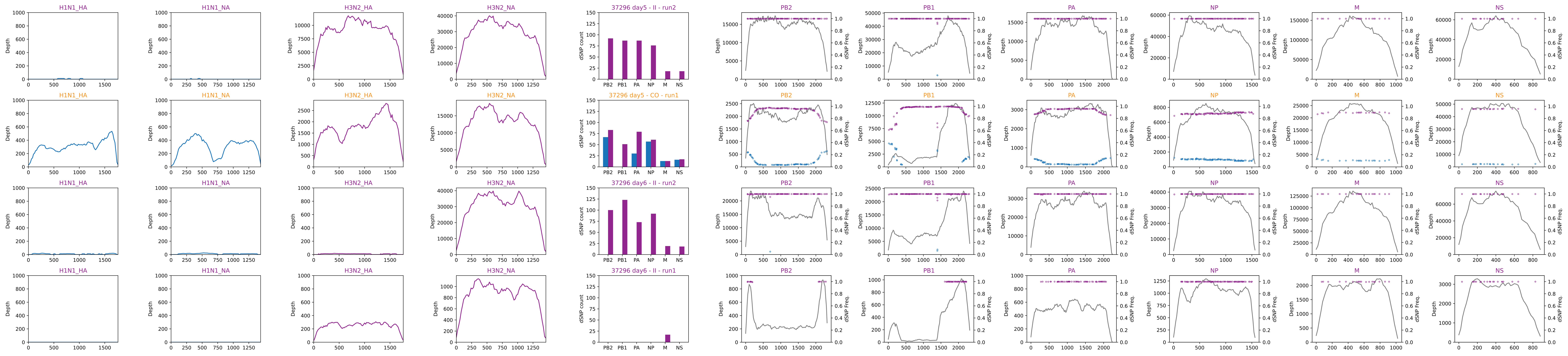
H1N1_HA
H1N1_NA
H3N2_HA
H3N2_NA
37296 day5 - II - run2
37296 day5 - CO - run1
37296 day6 - II - run2
37296 day6 - II - run1
PB2
PB1
PA
NP
M
NS
Depth
dSNP count
dSNP Freq.

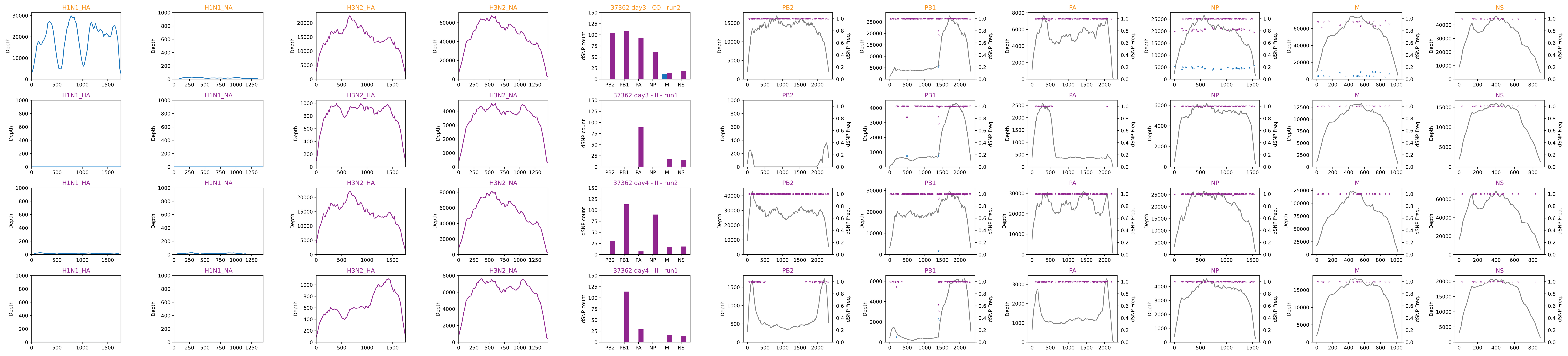
H1N1_HA
H1N1_NA
H3N2_HA
H3N2_NA
37362 day3 - CO - run2
PB2
PB1
PA
NP
M
NS
37362 day3 - II - run1
37362 day4 - II - run2
37362 day4 - II - run1
Depth
dSNP count
dSNP Freq.

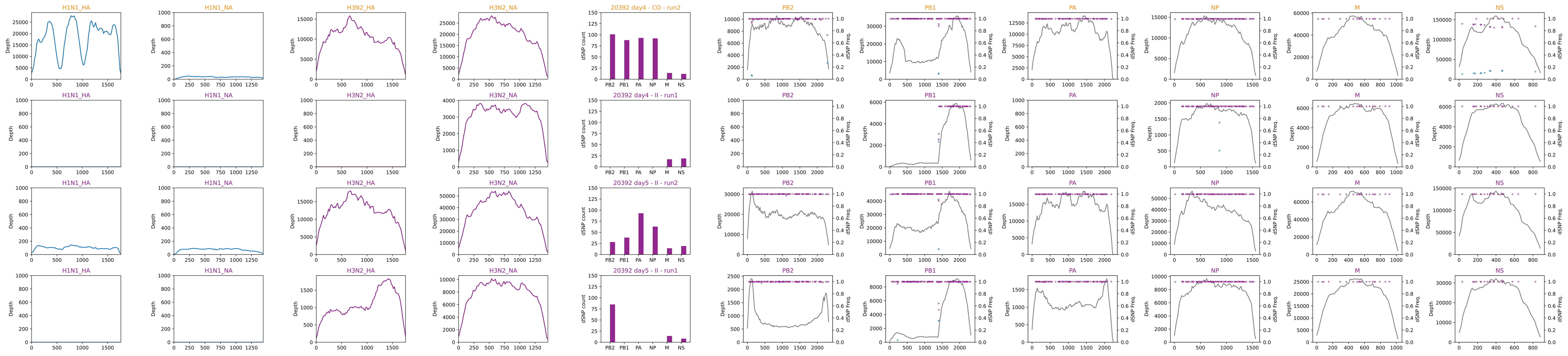
H1N1_HA
H1N1_NA
H3N2_HA
H3N2_NA
20392 day4 - CO - run2
PB2
PB1
PA
NP
M
NS
20392 day4 - II - run1
20392 day5 - II - run2
20392 day5 - II - run1
Depth
dSNP count
dSNP Freq.

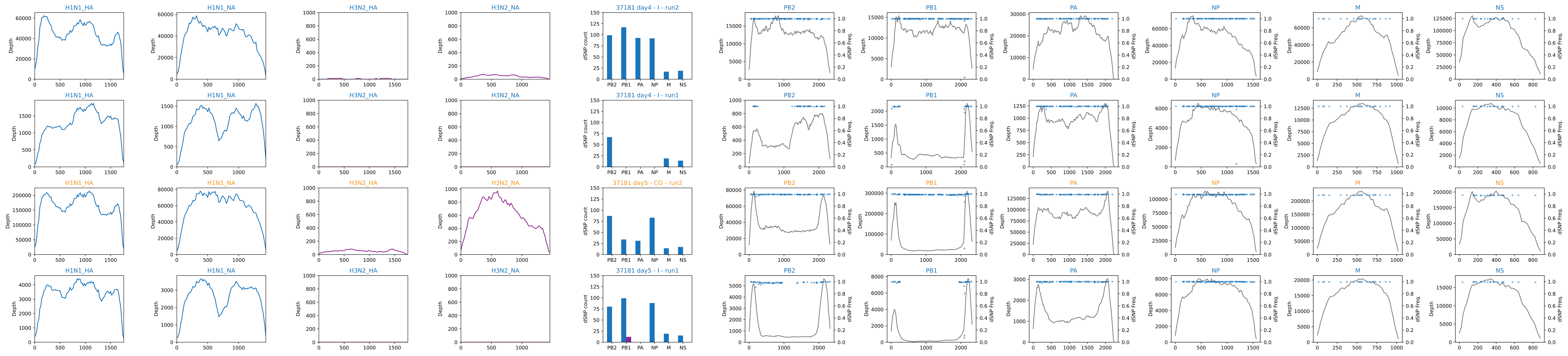
H1N1_HA
H1N1_NA
H3N2_HA
H3N2_NA
37181 day4 - I - run2
PB2
PB1
PA
NP
M
NS
37181 day4 - I - run1
37181 day5 - CO - run2
37181 day5 - I - run1
Depth
dSNP count
dSNP Freq.

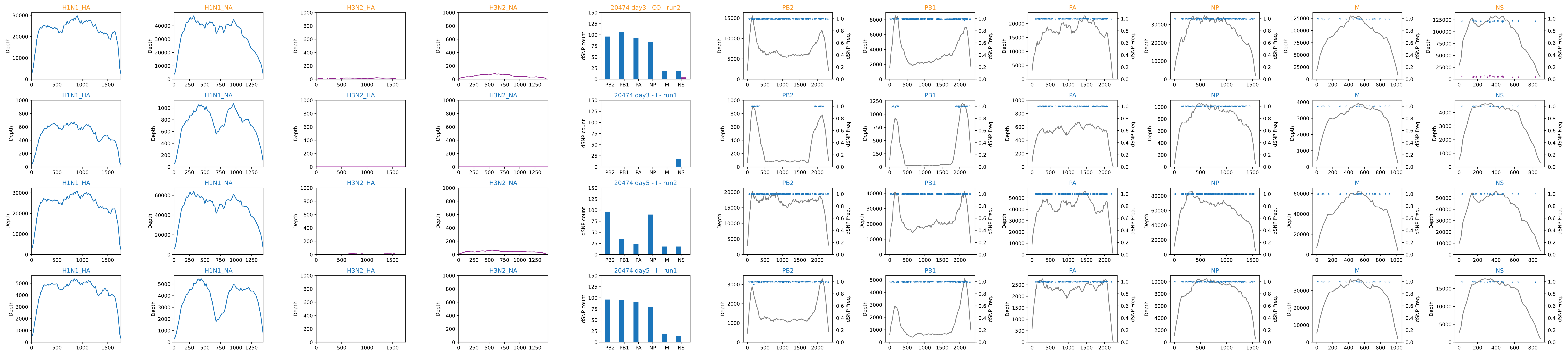
H1N1_HA
H1N1_NA
H3N2_HA
H3N2_NA
20474 day3 - CO - run2
PB2
PB1
PA
NP
M
NS
20474 day3 - I - run1
20474 day5 - I - run2
20474 day5 - I - run1
Depth
dSNP count
dSNP Freq.

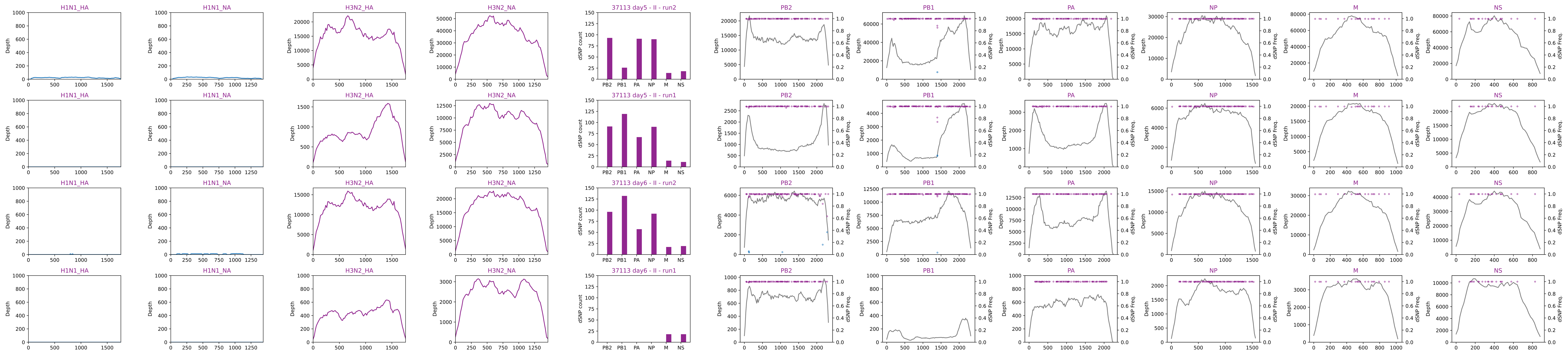
H1N1_HA
H1N1_NA
H3N2_HA
H3N2_NA
37113 day5 - II - run2
37113 day5 - II - run1
37113 day6 - II - run2
37113 day6 - II - run1
PB2
PB1
PA
NP
M
NS
Depth
dSNP count
dSNP Freq.

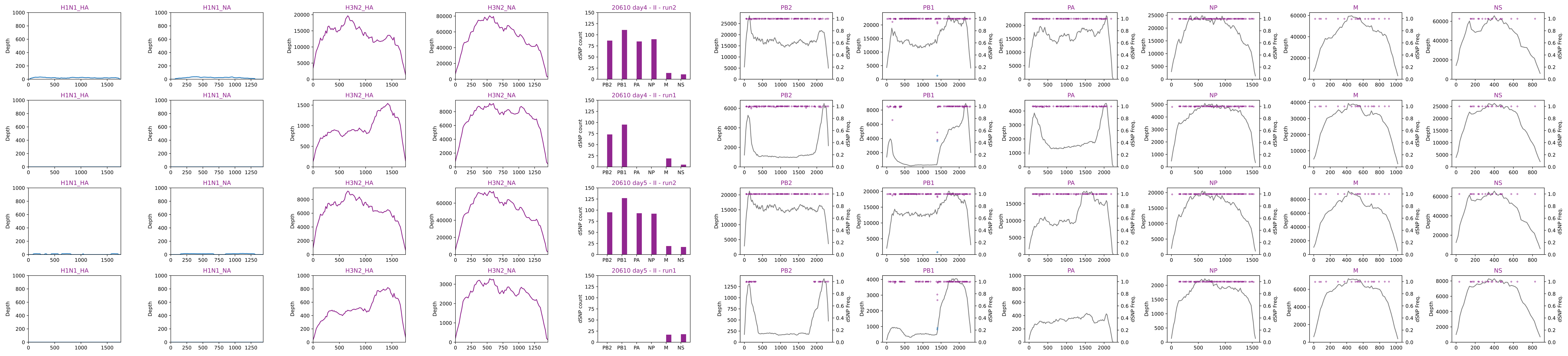
H1N1_HA
H1N1_NA
H3N2_HA
H3N2_NA
20610 day4 - II - run2
PB2
PB1
PA
NP
M
NS
20610 day4 - II - run1
20610 day5 - II - run2
20610 day5 - II - run1
Depth
dSNP count
dSNP Freq.

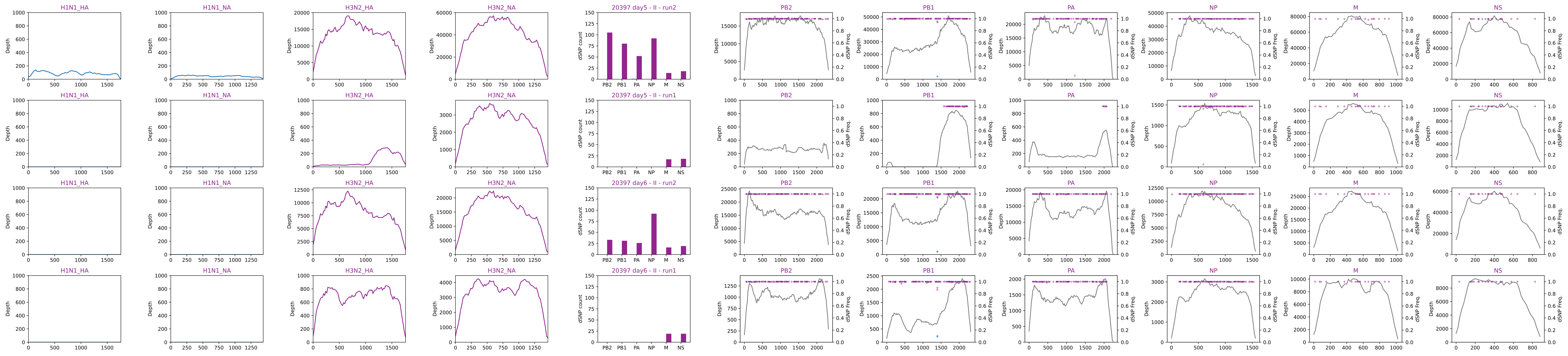
H1N1_HA
H1N1_NA
H3N2_HA
H3N2_NA
20397 day5 - II - run2
20397 day5 - II - run1
20397 day6 - II - run2
20397 day6 - II - run1
PB2
PB1
PA
NP
M
NS
Depth
dSNP count
dSNP Freq.

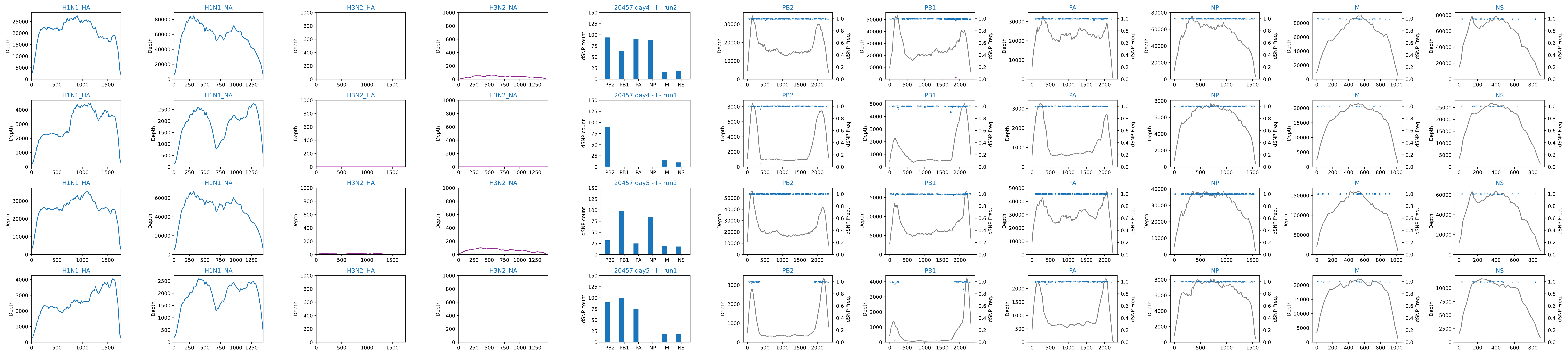
H1N1_HA
H1N1_NA
H3N2_HA
H3N2_NA
20457 day4 - I - run2
PB2
PB1
PA
NP
M
NS
20457 day4 - I - run1
20457 day5 - I - run2
20457 day5 - I - run1
Depth
dSNP count
dSNP Freq.

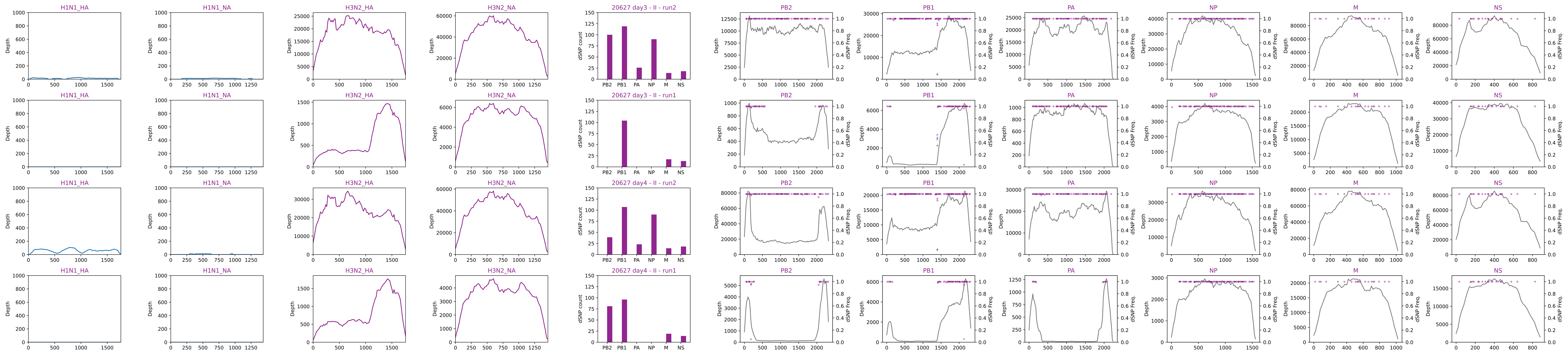
H1N1_HA
H1N1_NA
H3N2_HA
H3N2_NA
20627 day3 - II - run2
PB2
PB1
PA
NP
M
NS
H1N1_HA
H1N1_NA
H3N2_HA
H3N2_NA
20627 day3 - II - run1
PB2
PB1
PA
NP
M
NS
H1N1_HA
H1N1_NA
H3N2_HA
H3N2_NA
20627 day4 - II - run2
PB2
PB1
PA
NP
M
NS
H1N1_HA
H1N1_NA
H3N2_HA
H3N2_NA
20627 day4 - II - run1
PB2
PB1
PA
NP
M
NS
Depth
dSNP count
dSNP Freq.

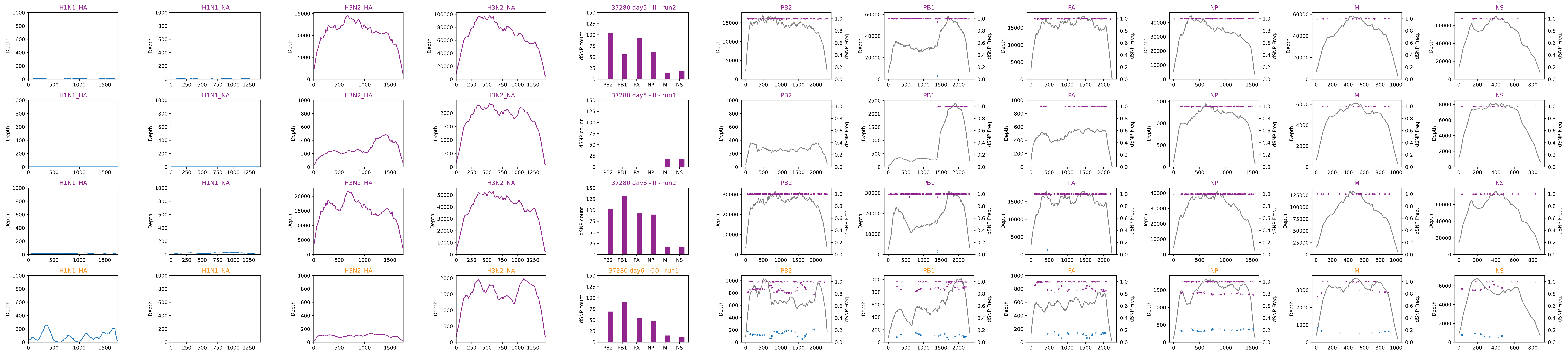

H1N1_HA
H1N1_NA
H3N2_HA
H3N2_NA
37280 day5 - II - run2
PB2
PB1
PA
NP
M
NS
H1N1_HA
H1N1_NA
H3N2_HA
H3N2_NA
37280 day5 - II - run1
PB2
PB1
PA
NP
M
NS
H1N1_HA
H1N1_NA
H3N2_HA
H3N2_NA
37280 day6 - II - run2
PB2
PB1
PA
NP
M
NS
H1N1_HA
H1N1_NA
H3N2_HA
H3N2_NA
37280 day6 - CO - run1
PB2
PB1
PA
NP
M
NS
Depth
dSNP count
dSNP Freq.

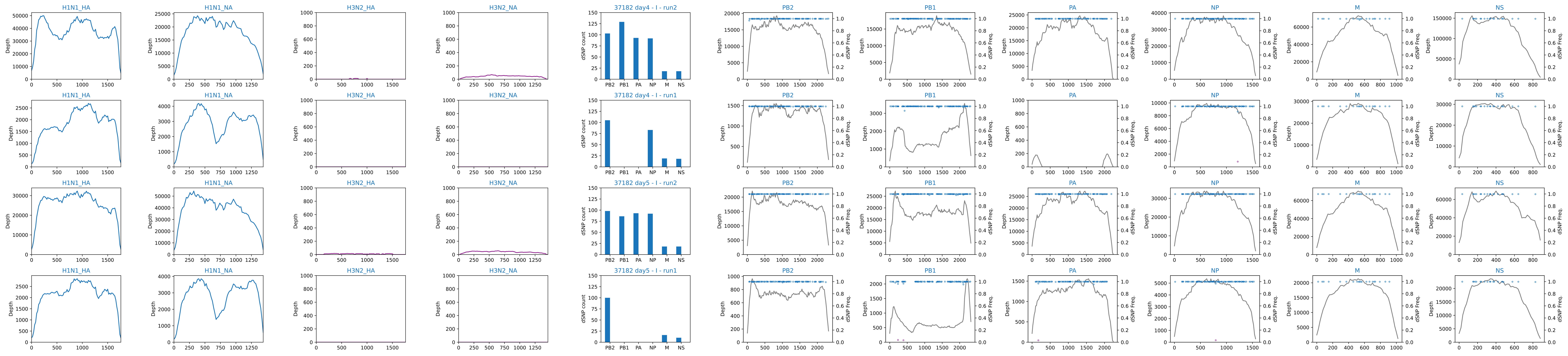
H1N1_HA
H1N1_NA
H3N2_HA
H3N2_NA
37182 day4 - I - run2
37182 day4 - I - run1
37182 day5 - I - run2
37182 day5 - I - run1
PB2
PB1
PA
NP
M
NS
Depth
dSNP count
dSNP Freq.

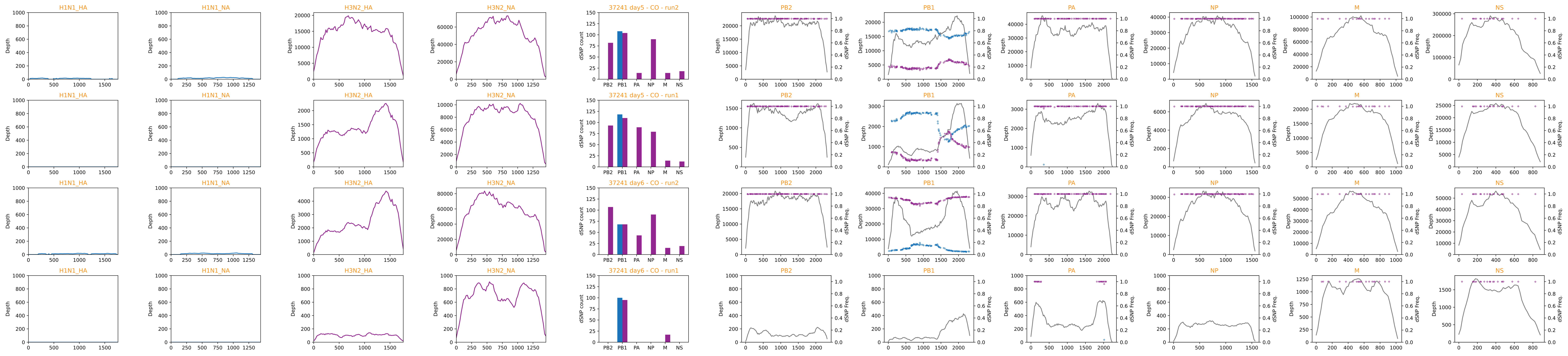
H1N1_HA
H1N1_NA
H3N2_HA
H3N2_NA
37241 day5 - CO - run2
PB2
PB1
PA
NP
M
NS
37241 day5 - CO - run1
37241 day6 - CO - run2
37241 day6 - CO - run1
Depth
dSNP count
dSNP Freq.
PB2 PB1 PA NP M NS

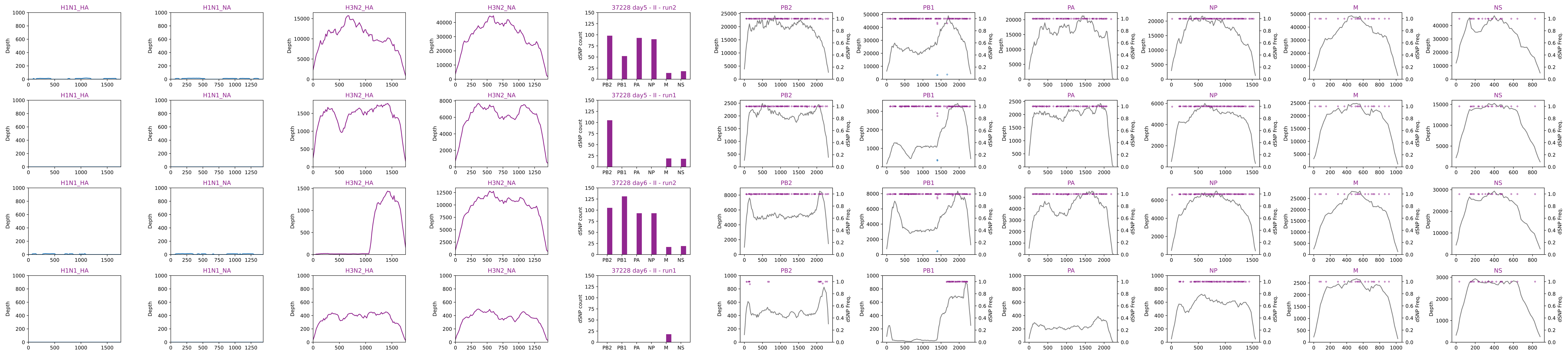

H1N1_HA
H1N1_NA
H3N2_HA
H3N2_NA
37228 day5 - II - run2
PB2
PB1
PA
NP
M
NS
37228 day5 - II - run1
37228 day6 - II - run2
37228 day6 - II - run1
Depth
dSNP count
dSNP Freq.

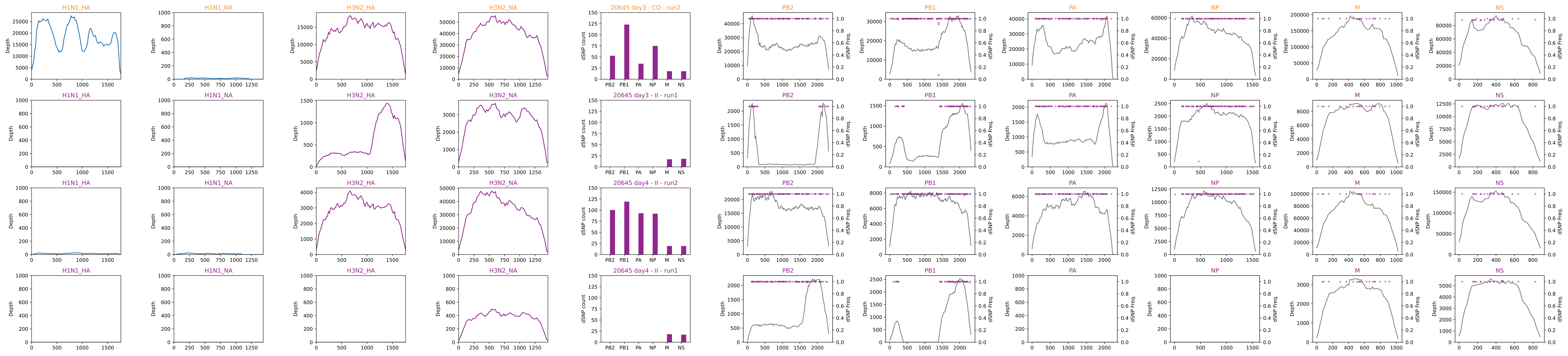
H1N1_HA
H1N1_NA
H3N2_HA
H3N2_NA
20645 day3 - CO - run2
20645 day3 - II - run1
20645 day4 - II - run2
20645 day4 - II - run1
PB2
PB1
PA
NP
M
NS
PB2 PB1 PA NP M NS
Depth
dSNP count
dSNP Freq.

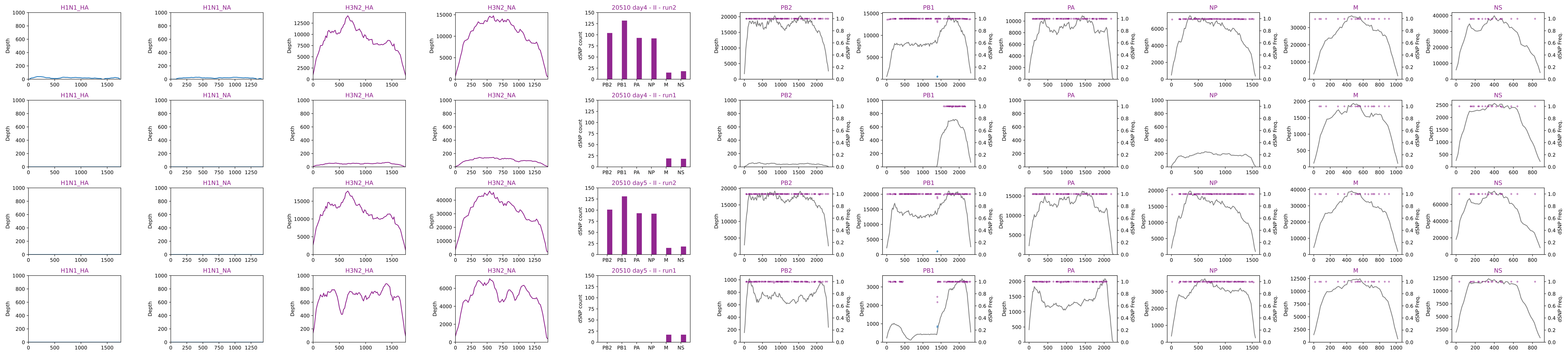
H1N1_HA
H1N1_NA
H3N2_HA
H3N2_NA
20510 day4 - II - run2
PB2
PB1
PA
NP
M
NS
20510 day4 - II - run1
20510 day5 - II - run2
20510 day5 - II - run1
Depth
dSNP count
dSNP Freq.
PB2 PB1 PA NP M NS

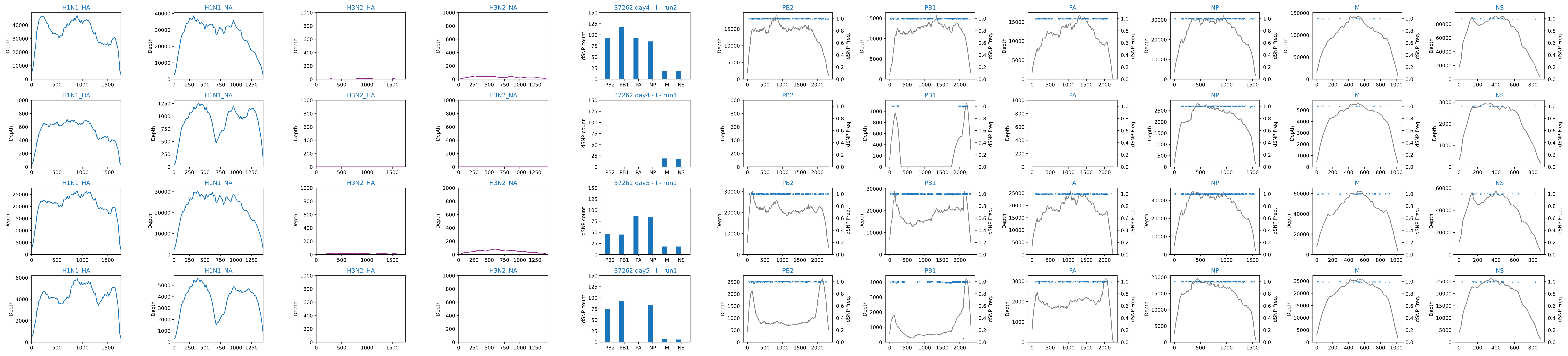

H1N1_HA
H1N1_NA
H3N2_HA
H3N2_NA
37262 day4 - I - run2
37262 day4 - I - run1
37262 day5 - I - run2
37262 day5 - I - run1
PB2
PB1
PA
NP
M
NS
Depth
dSNP count
dSNP Freq.

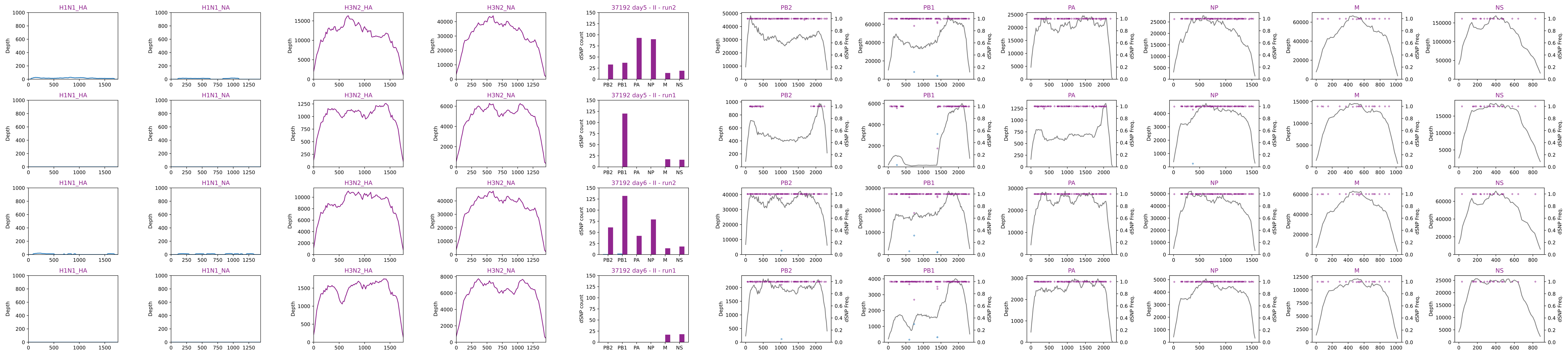
H1N1_HA
H1N1_NA
H3N2_HA
H3N2_NA
37192 day5 - II - run2
37192 day5 - II - run1
37192 day6 - II - run2
37192 day6 - II - run1
PB2
PB1
PA
NP
M
NS
Depth
dSNP count
dSNP Freq.

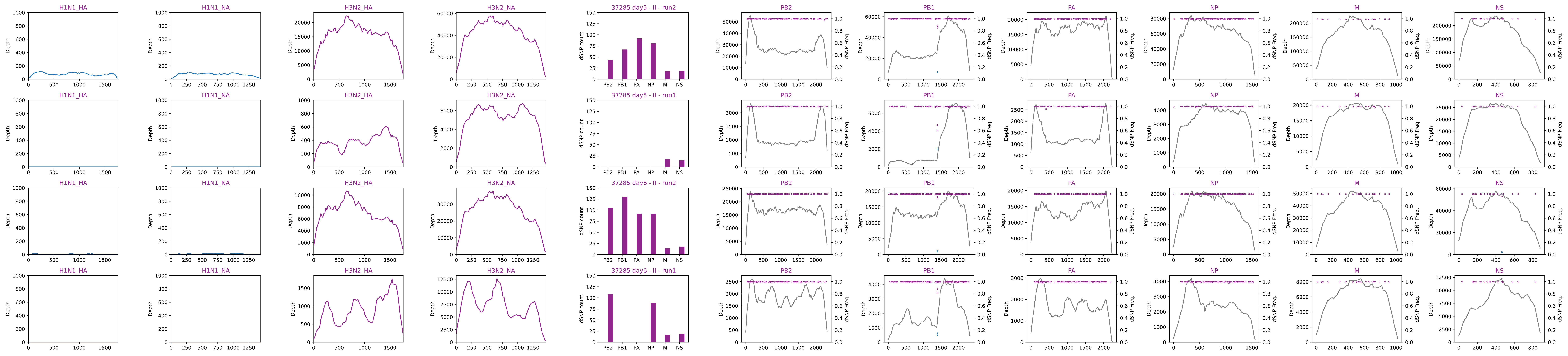

H1N1_HA
H1N1_NA
H3N2_HA
H3N2_NA
37285 day5 - II - run2
37285 day5 - II - run1
37285 day6 - II - run2
37285 day6 - II - run1
PB2
PB1
PA
NP
M
NS
Depth
dSNP count
dSNP Freq.

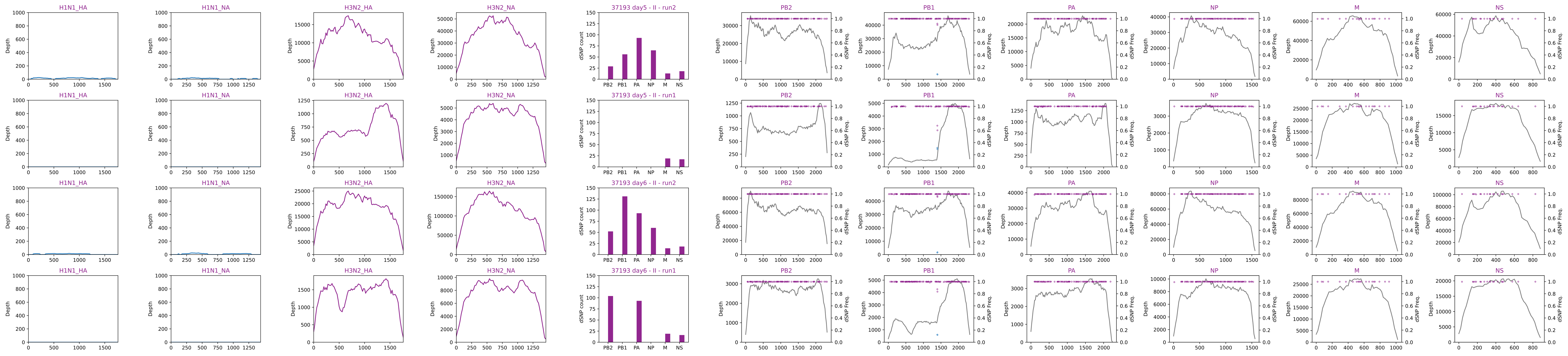
H1N1_HA
H1N1_NA
H3N2_HA
H3N2_NA
37193 day5 - II - run2
37193 day5 - II - run1
37193 day6 - II - run2
37193 day6 - II - run1
PB2
PB1
PA
NP
M
NS
Depth
dSNP count
dSNP Freq.

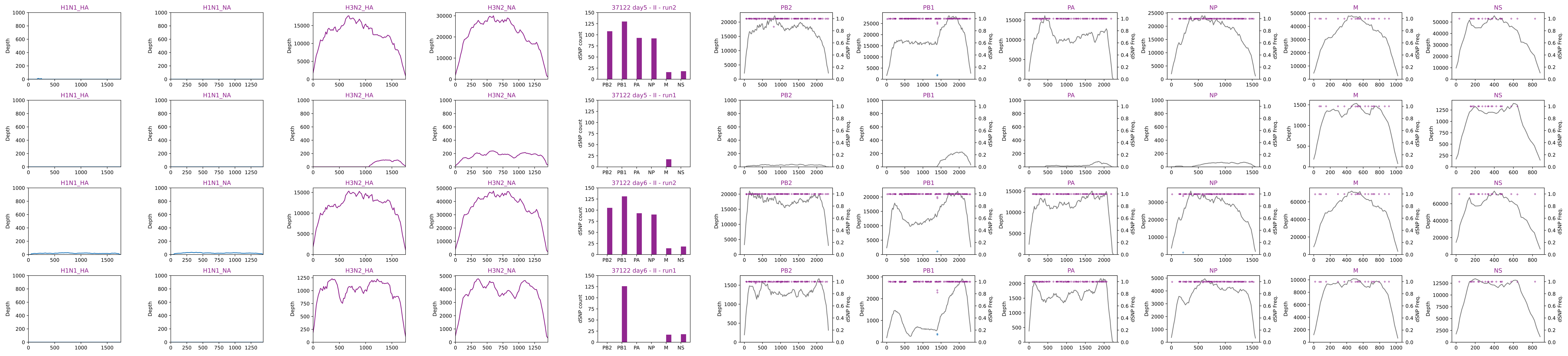

H1N1_HA
H1N1_NA
H3N2_HA
H3N2_NA
37122 day5 - II - run2
37122 day5 - II - run1
37122 day6 - II - run2
37122 day6 - II - run1
PB2
PB1
PA
NP
M
NS
Depth
dSNP count
dSNP Freq.

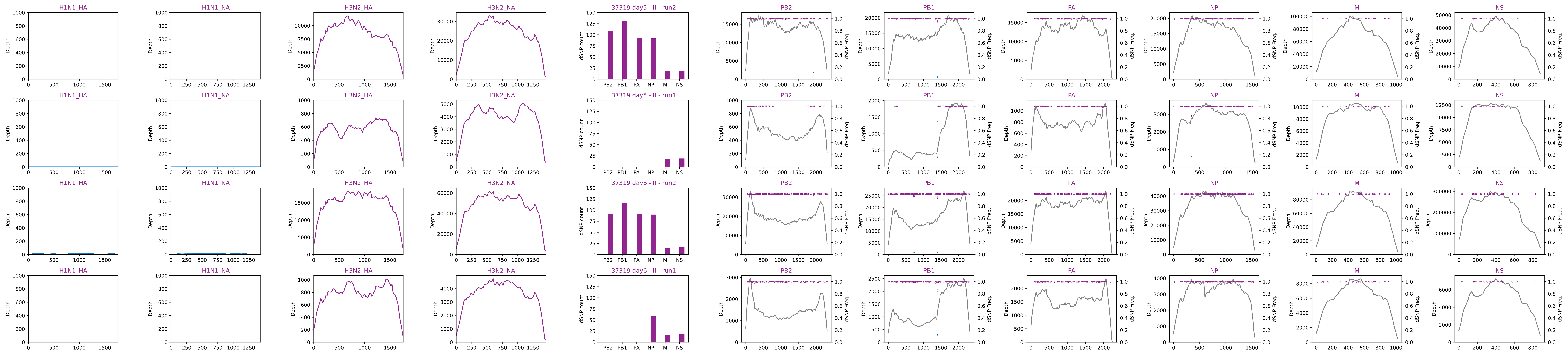

H1N1_HA
H1N1_NA
H3N2_HA
H3N2_NA
37319 day5 - II - run2
PB2
PB1
PA
NP
M
NS
37319 day5 - II - run1
37319 day6 - II - run2
37319 day6 - II - run1
Depth
dSNP count
dSNP Freq.

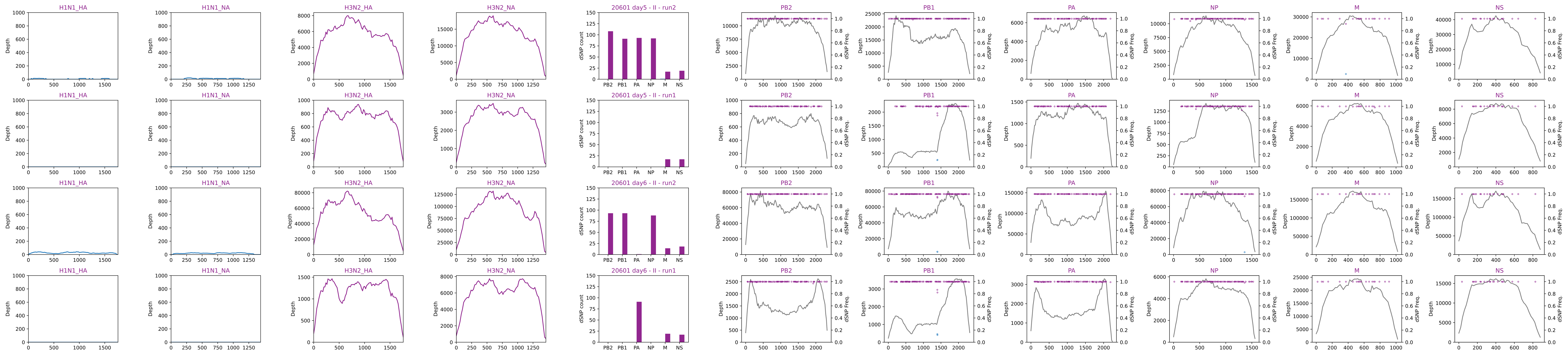

H1N1_HA
H1N1_NA
H3N2_HA
H3N2_NA
20601 day5 - II - run2
PB2
PB1
PA
NP
M
NS
H1N1_HA
H1N1_NA
H3N2_HA
H3N2_NA
20601 day5 - II - run1
PB2
PB1
PA
NP
M
NS
H1N1_HA
H1N1_NA
H3N2_HA
H3N2_NA
20601 day6 - II - run2
PB2
PB1
PA
NP
M
NS
H1N1_HA
H1N1_NA
H3N2_HA
H3N2_NA
20601 day6 - II - run1
PB2
PB1
PA
NP
M
NS
Depth
dSNP count
dSNP Freq.

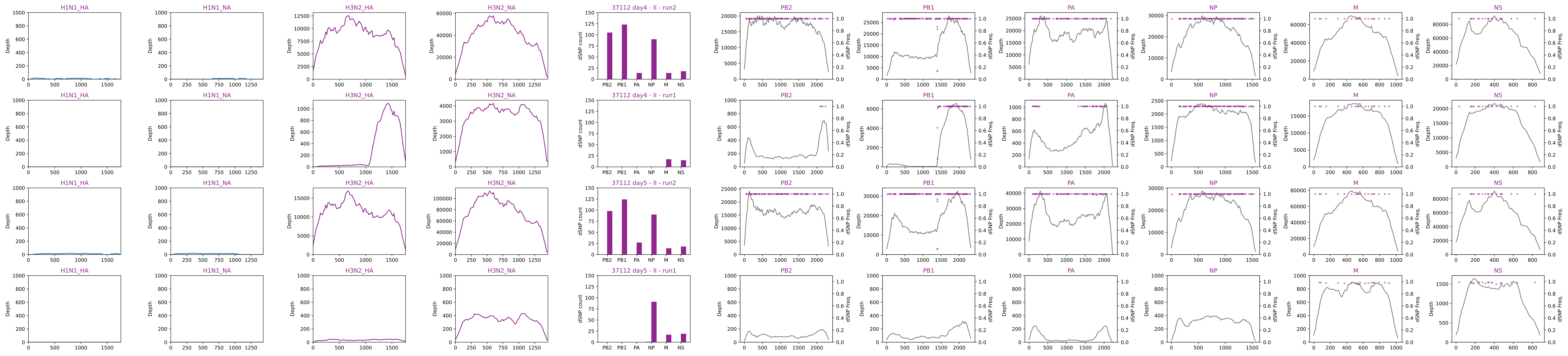

H1N1_HA
H1N1_NA
H3N2_HA
H3N2_NA
37112 day4 - II - run2
PB2
PB1
PA
NP
M
NS
37112 day4 - II - run1
37112 day5 - II - run2
37112 day5 - II - run1
Depth
dSNP count
dSNP Freq.

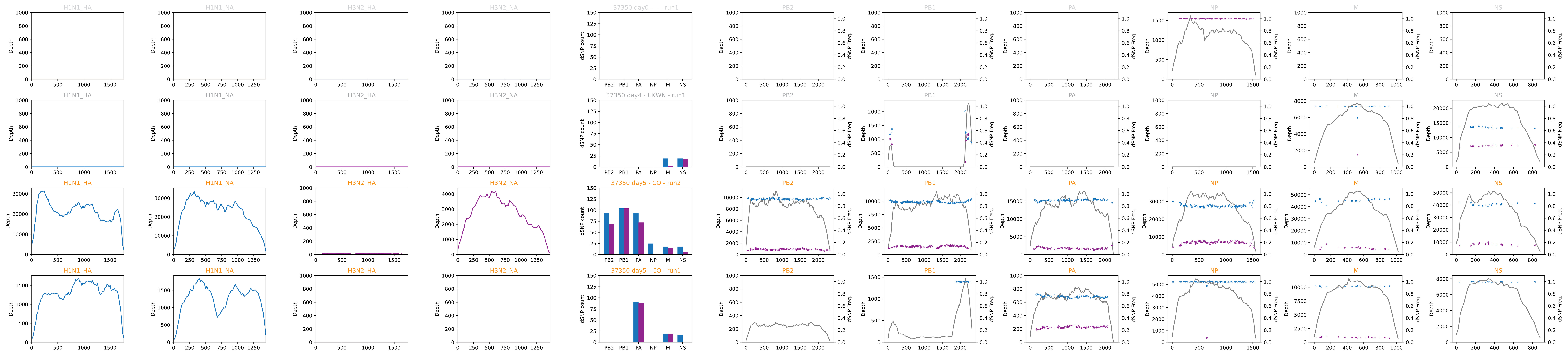
H1N1_HA
H1N1_NA
H3N2_HA
H3N2_NA
37350 day0 - - - - run1
PB2
PB1
PA
NP
M
NS
37350 day4 - UKWN - run1
37350 day5 - CO - run2
37350 day5 - CO - run1
Depth
dSNP count
dSNP Freq.

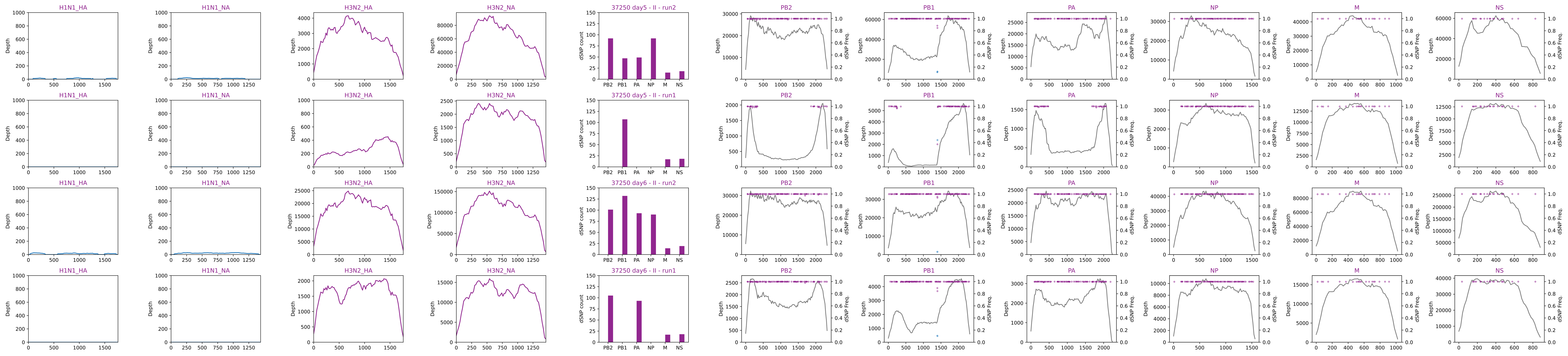

H1N1_HA
H1N1_NA
H3N2_HA
H3N2_NA
37250 day5 - II - run2
PB2
PB1
PA
NP
M
NS
37250 day5 - II - run1
37250 day6 - II - run2
37250 day6 - II - run1
Depth
dSNP count
dSNP Freq.

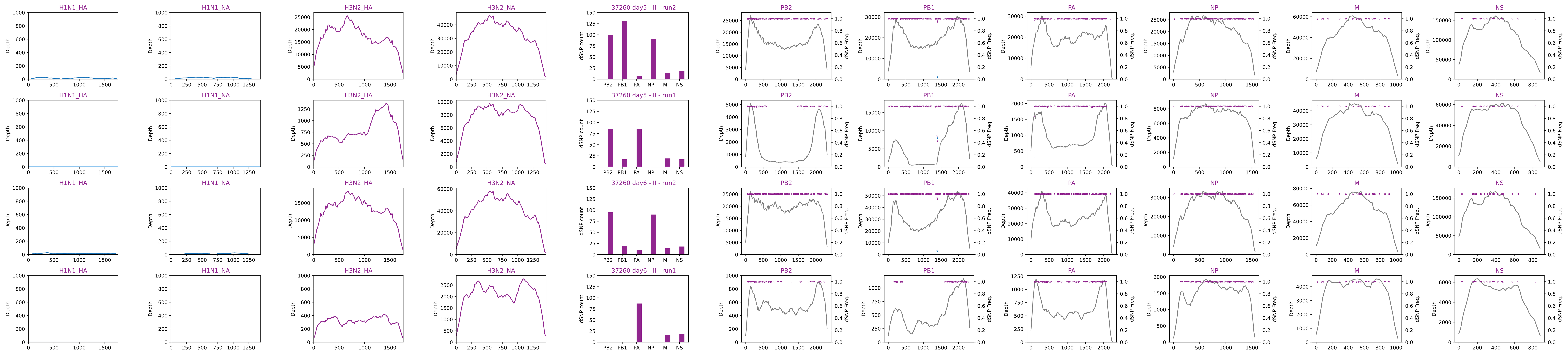

H1N1_HA
H1N1_NA
H3N2_HA
H3N2_NA
37260 day5 - II - run2
PB2
PB1
PA
NP
M
NS
37260 day5 - II - run1
37260 day6 - II - run2
37260 day6 - II - run1
Depth
dSNP count
dSNP Freq.

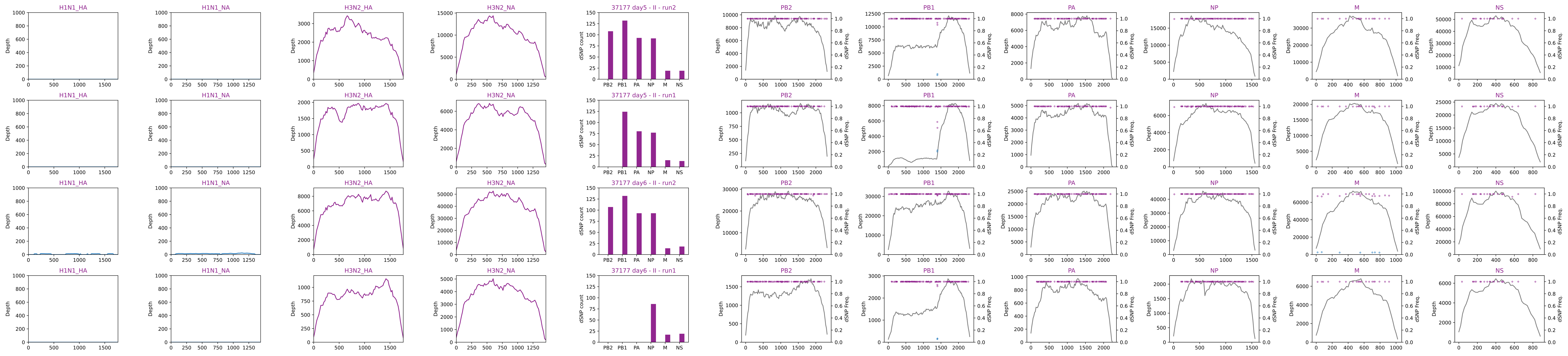

H1N1_HA
H1N1_NA
H3N2_HA
H3N2_NA
37177 day5 - II - run2
37177 day5 - II - run1
37177 day6 - II - run2
37177 day6 - II - run1
PB2
PB1
PA
NP
M
NS
Depth
dSNP count
dSNP Freq.

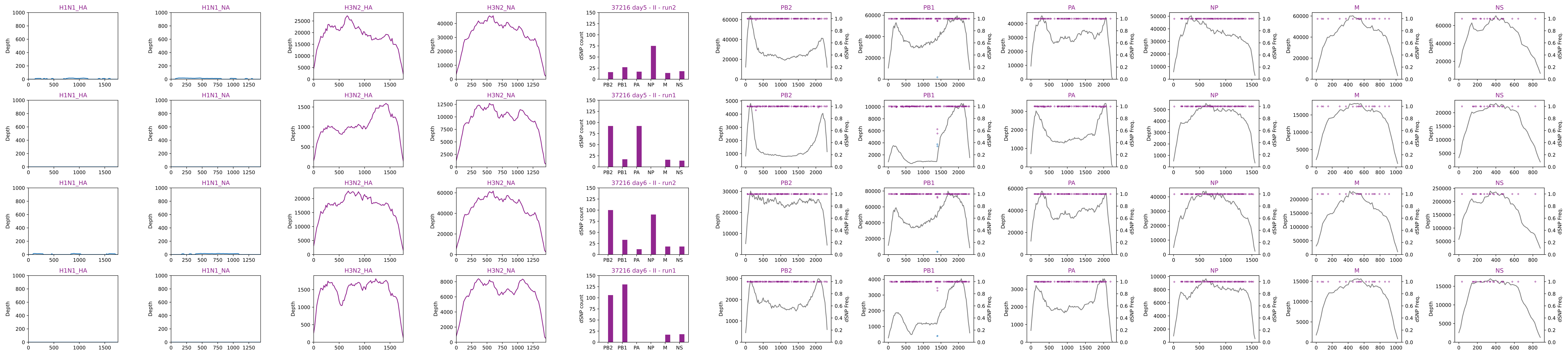
H1N1_HA
H1N1_NA
H3N2_HA
H3N2_NA
37216 day5 - II - run2
PB2
PB1
PA
NP
M
NS
H1N1_HA
H1N1_NA
H3N2_HA
H3N2_NA
37216 day5 - II - run1
PB2
PB1
PA
NP
M
NS
H1N1_HA
H1N1_NA
H3N2_HA
H3N2_NA
37216 day6 - II - run2
PB2
PB1
PA
NP
M
NS
H1N1_HA
H1N1_NA
H3N2_HA
H3N2_NA
37216 day6 - II - run1
PB2
PB1
PA
NP
M
NS
Depth
dSNP count
dSNP Freq.

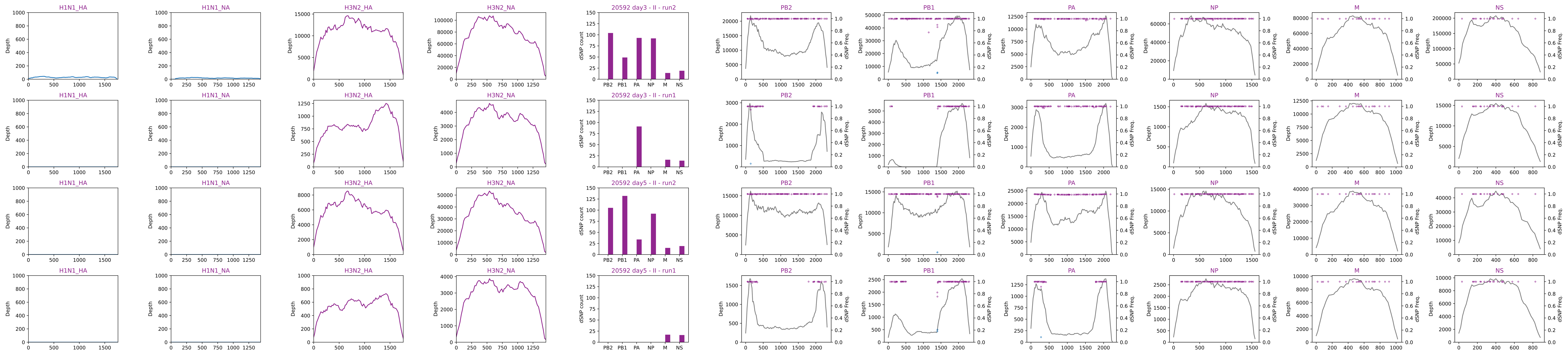

H1N1_HA
H1N1_NA
H3N2_HA
H3N2_NA
20592 day3 - II - run2
PB2
PB1
PA
NP
M
NS
20592 day3 - II - run1
20592 day5 - II - run2
20592 day5 - II - run1
Depth
dSNP count
dSNP Freq.

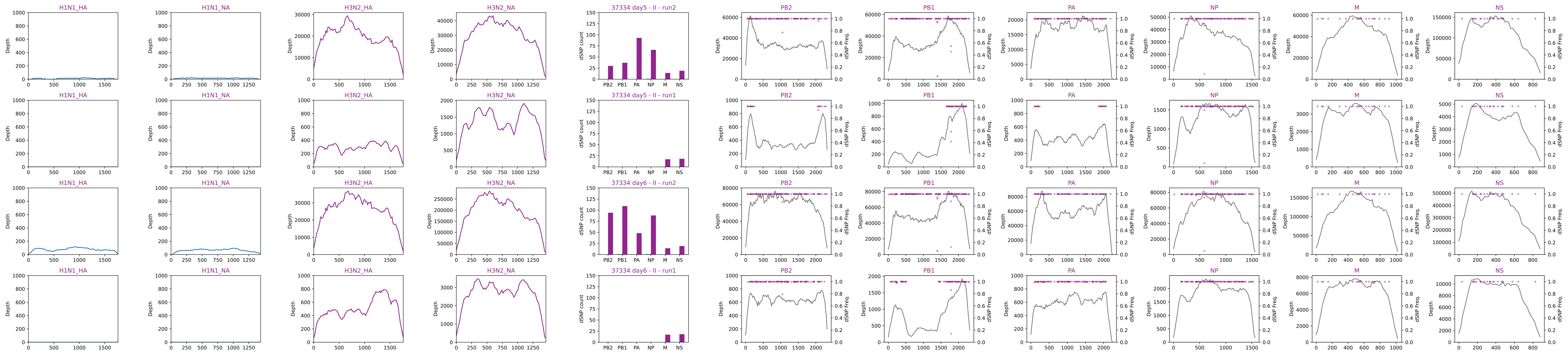
H1N1_HA
H1N1_NA
H3N2_HA
H3N2_NA
37334 day5 - II - run2
PB2
PB1
PA
NP
M
NS
H1N1_HA
H1N1_NA
H3N2_HA
H3N2_NA
37334 day5 - II - run1
PB2
PB1
PA
NP
M
NS
H1N1_HA
H1N1_NA
H3N2_HA
H3N2_NA
37334 day6 - II - run2
PB2
PB1
PA
NP
M
NS
H1N1_HA
H1N1_NA
H3N2_HA
H3N2_NA
37334 day6 - II - run1
PB2
PB1
PA
NP
M
NS
Depth
dSNP count
dSNP Freq.
PB2 PB1 PA NP M NS

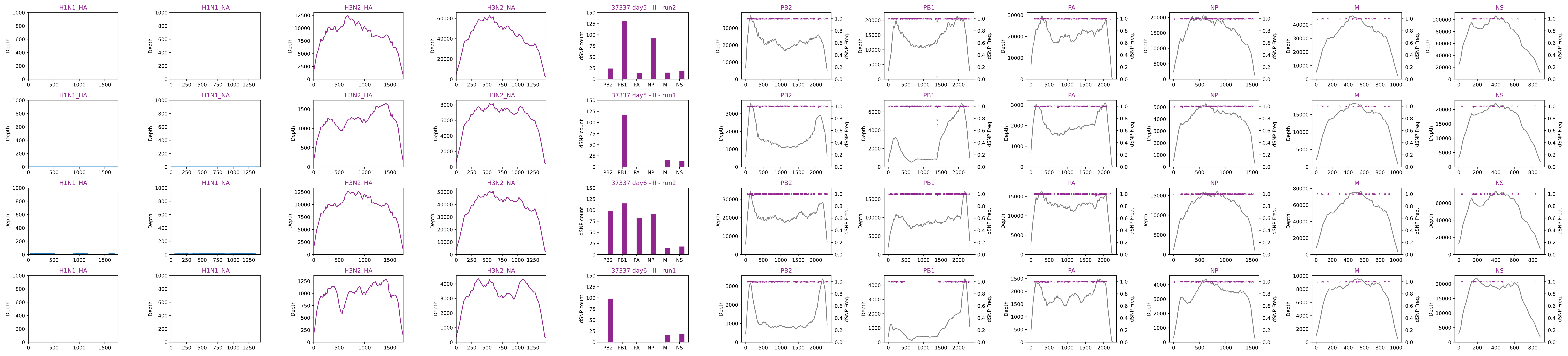
H1N1_HA
H1N1_NA
H3N2_HA
H3N2_NA
37337 day5 - II - run2
PB2
PB1
PA
NP
M
NS
37337 day5 - II - run1
37337 day6 - II - run2
37337 day6 - II - run1
Depth
dSNP count
dSNP Freq.

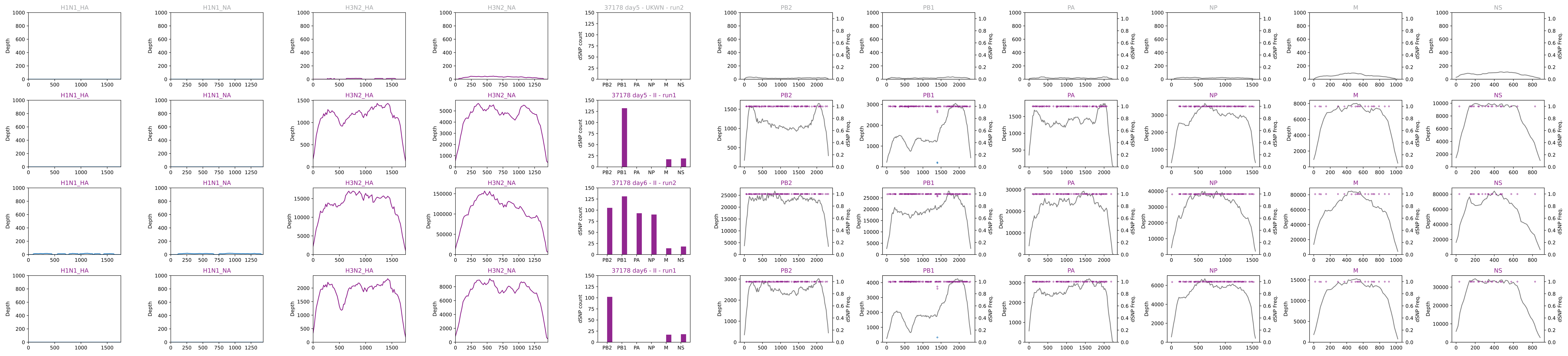

H1N1_HA
H1N1_NA
H3N2_HA
H3N2_NA
37178 day5 - UKWN - run2
37178 day5 - II - run1
37178 day6 - II - run2
37178 day6 - II - run1
PB2
PB1
PA
NP
M
NS
Depth
dSNP count
dSNP Freq.

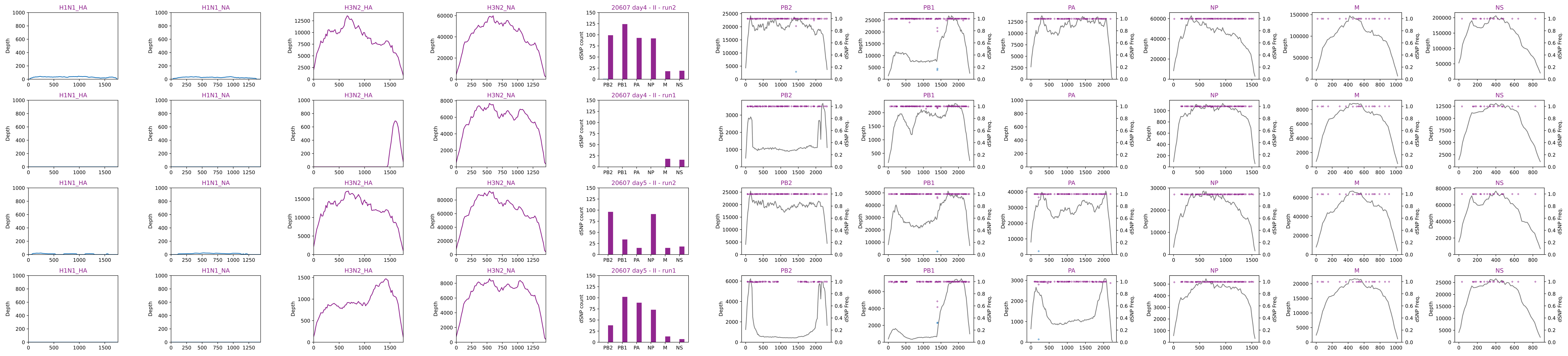
H1N1_HA
H1N1_NA
H3N2_HA
H3N2_NA
20607 day4 - II - run2
PB2
PB1
PA
NP
M
NS
H1N1_HA
H1N1_NA
H3N2_HA
H3N2_NA
20607 day4 - II - run1
PB2
PB1
PA
NP
M
NS
H1N1_HA
H1N1_NA
H3N2_HA
H3N2_NA
20607 day5 - II - run2
PB2
PB1
PA
NP
M
NS
H1N1_HA
H1N1_NA
H3N2_HA
H3N2_NA
20607 day5 - II - run1
PB2
PB1
PA
NP
M
NS
Depth
dSNP count
dSNP Freq.
PB2 PB1 PA NP M NS

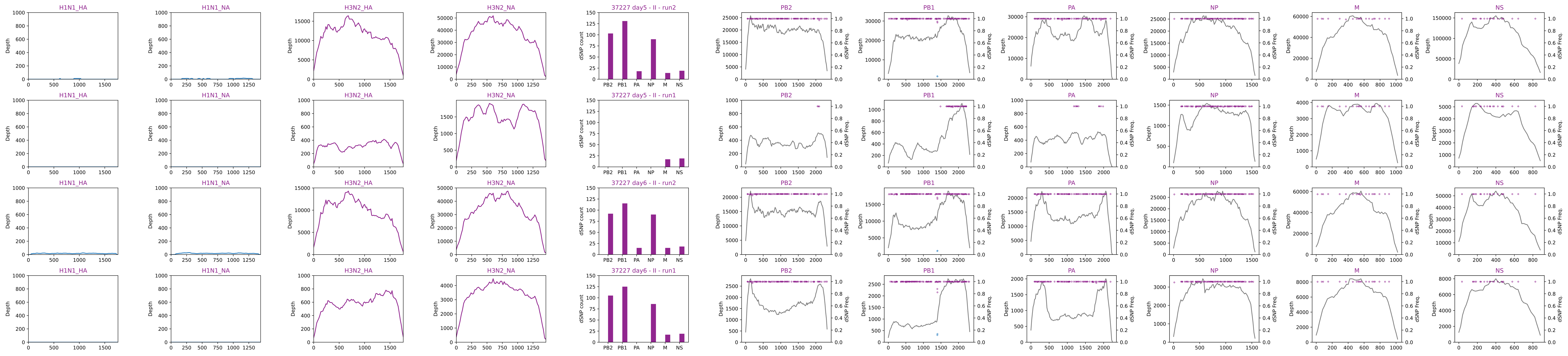

H1N1_HA
H1N1_NA
H3N2_HA
H3N2_NA
37227 day5 - II - run2
37227 day5 - II - run1
37227 day6 - II - run2
37227 day6 - II - run1
PB2
PB1
PA
NP
M
NS
Depth
dSNP count
dSNP Freq.

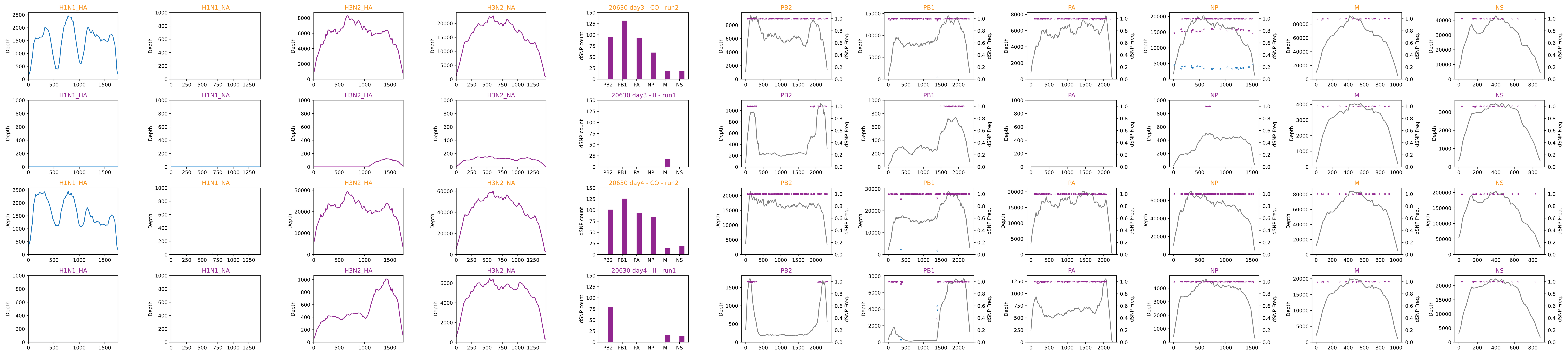

H1N1_HA
H1N1_NA
H3N2_HA
H3N2_NA
20630 day3 - CO - run2
PB2
PB1
PA
NP
M
NS
20630 day3 - II - run1
20630 day4 - CO - run2
20630 day4 - II - run1
Depth
dSNP count
dSNP Freq.

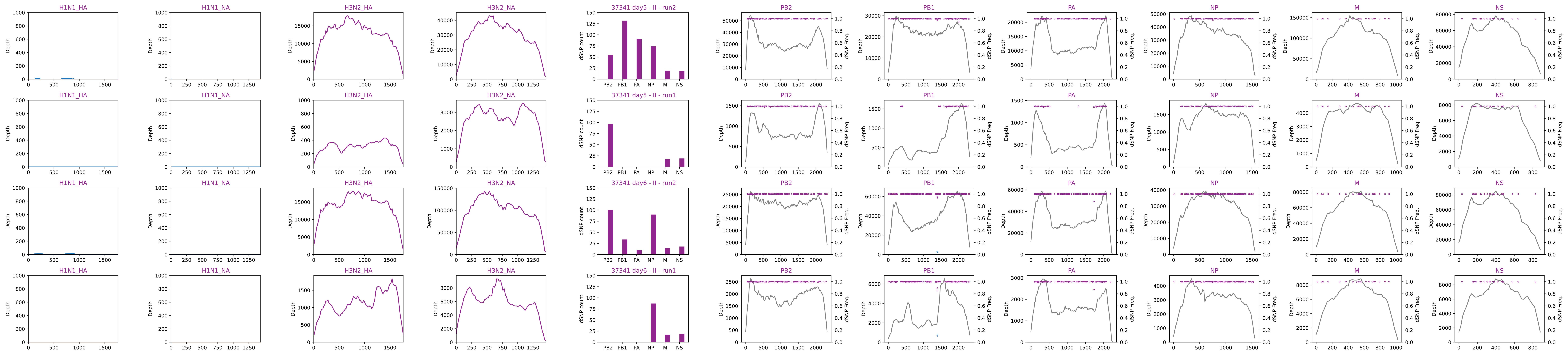

H1N1_HA
H1N1_NA
H3N2_HA
H3N2_NA
37341 day5 - II - run2
37341 day5 - II - run1
37341 day6 - II - run2
37341 day6 - II - run1
PB2
PB1
PA
NP
M
NS
Depth
dSNP count
dSNP Freq.

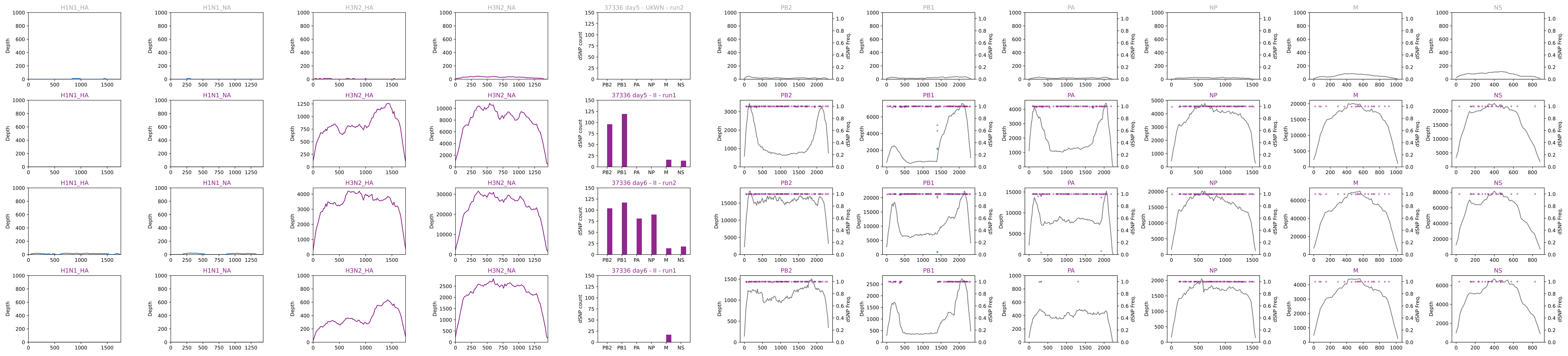

37336 day5 - UKWN - run2
37336 day5 - II - run1
37336 day6 - II - run2
37336 day6 - II - run1
H1N1_HA
H1N1_NA
H3N2_HA
H3N2_NA
PB2
PB1
PA
NP
M
NS
Depth
dSNP count
dSNP Freq.

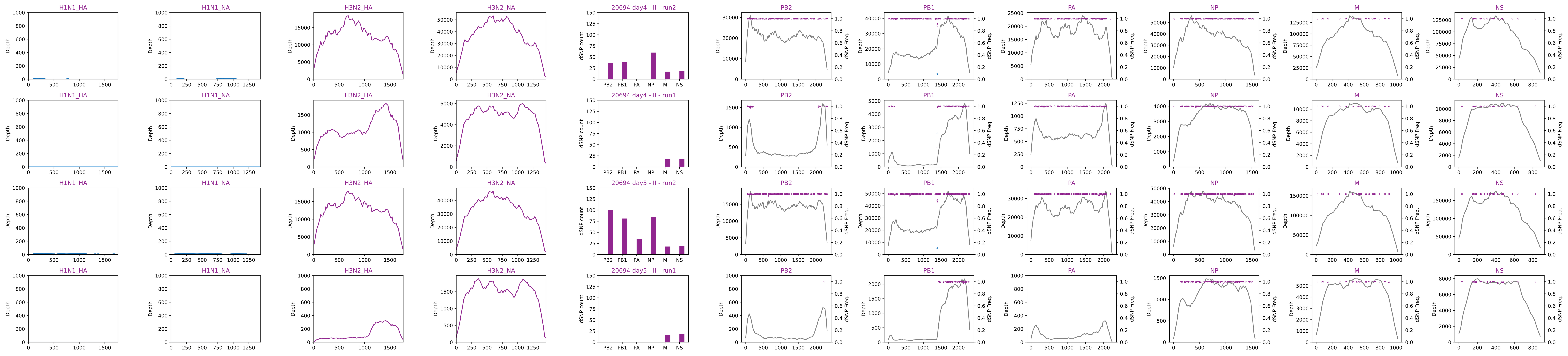
H1N1_HA
H1N1_NA
H3N2_HA
H3N2_NA
20694 day4 - II - run2
PB2
PB1
PA
NP
M
NS
H1N1_HA
H1N1_NA
H3N2_HA
H3N2_NA
20694 day4 - II - run1
PB2
PB1
PA
NP
M
NS
H1N1_HA
H1N1_NA
H3N2_HA
H3N2_NA
20694 day5 - II - run2
PB2
PB1
PA
NP
M
NS
H1N1_HA
H1N1_NA
H3N2_HA
H3N2_NA
20694 day5 - II - run1
PB2
PB1
PA
NP
M
NS
Depth
dSNP count
dSNP Freq.
PB2 PB1 PA NP M NS

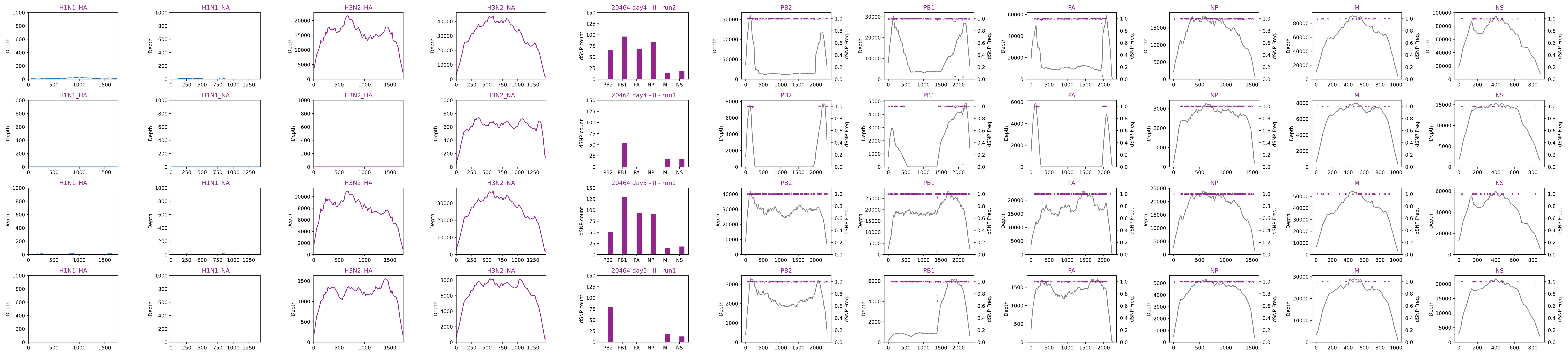

H1N1_HA
H1N1_NA
H3N2_HA
H3N2_NA
20464 day4 - II - run2
20464 day4 - II - run1
20464 day5 - II - run2
20464 day5 - II - run1
PB2
PB1
PA
NP
M
NS
Depth
dSNP count
dSNP Freq.

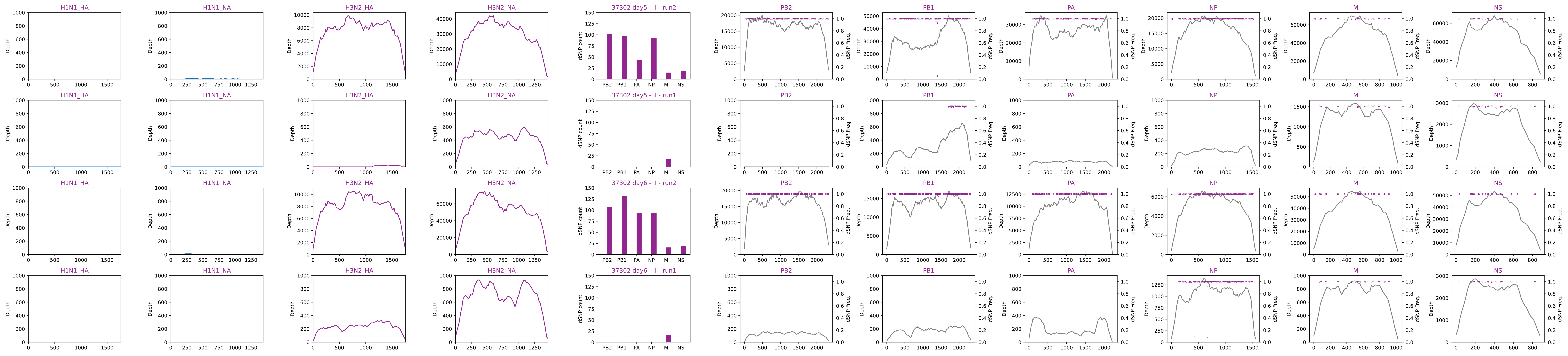

H1N1_HA
H1N1_NA
H3N2_HA
H3N2_NA
37302 day5 - II - run2
PB2
PB1
PA
NP
M
NS
37302 day5 - II - run1
37302 day6 - II - run2
37302 day6 - II - run1
Depth
dSNP count
dSNP Freq.

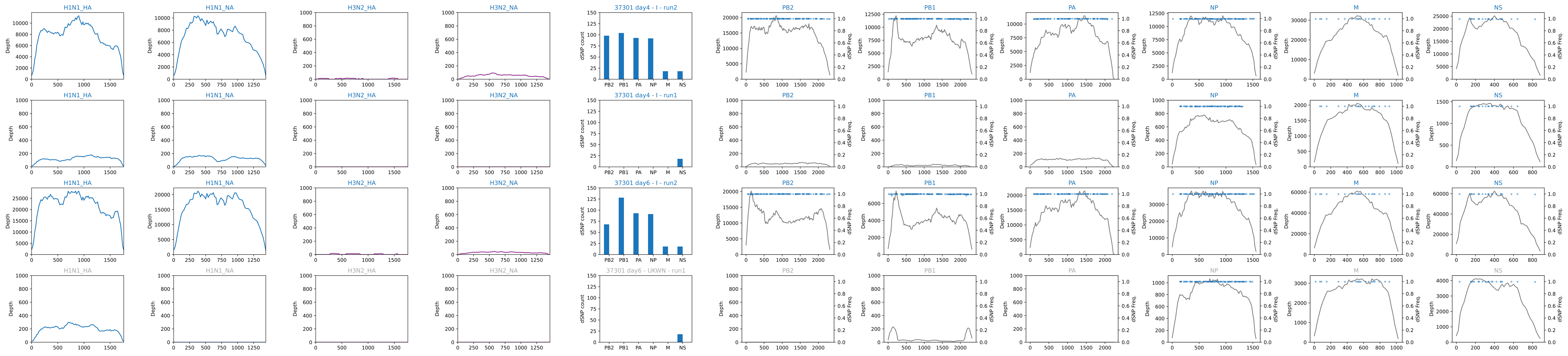

H1N1_HA
H1N1_NA
H3N2_HA
H3N2_NA
37301 day4 - I - run2
PB2
PB1
PA
NP
M
NS
H1N1_HA
H1N1_NA
H3N2_HA
H3N2_NA
37301 day4 - I - run1
PB2
PB1
PA
NP
M
NS
H1N1_HA
H1N1_NA
H3N2_HA
H3N2_NA
37301 day6 - I - run2
PB2
PB1
PA
NP
M
NS
H1N1_HA
H1N1_NA
H3N2_HA
H3N2_NA
37301 day6 - UKWN - run1
PB2
PB1
PA
NP
M
NS
Depth
dSNP count
dSNP Freq.

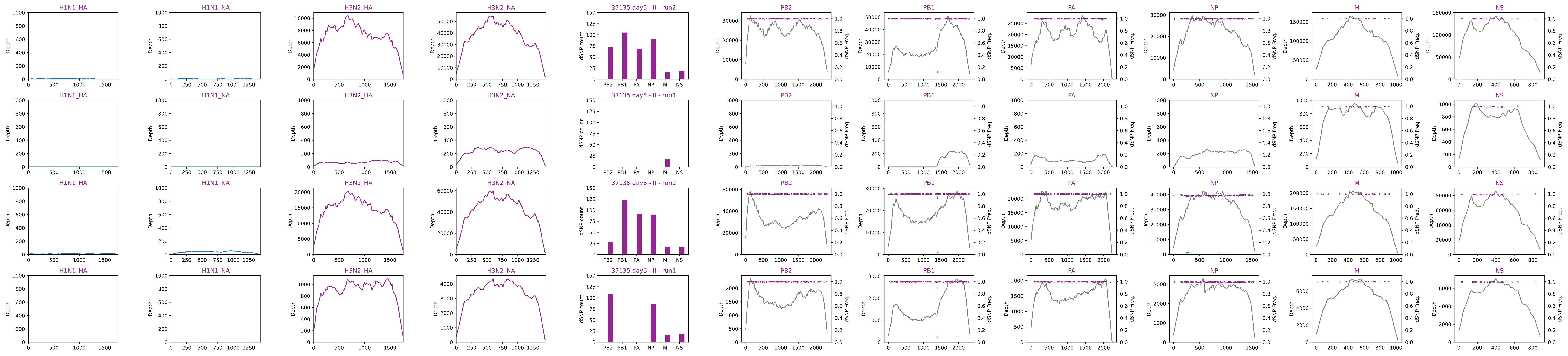
H1N1_HA
H1N1_NA
H3N2_HA
H3N2_NA
37135 day5 - II - run2
PB2
PB1
PA
NP
M
NS
H1N1_HA
H1N1_NA
H3N2_HA
H3N2_NA
37135 day5 - II - run1
PB2
PB1
PA
NP
M
NS
H1N1_HA
H1N1_NA
H3N2_HA
H3N2_NA
37135 day6 - II - run2
PB2
PB1
PA
NP
M
NS
H1N1_HA
H1N1_NA
H3N2_HA
H3N2_NA
37135 day6 - II - run1
PB2
PB1
PA
NP
M
NS
Depth
dSNP count
dSNP Freq.

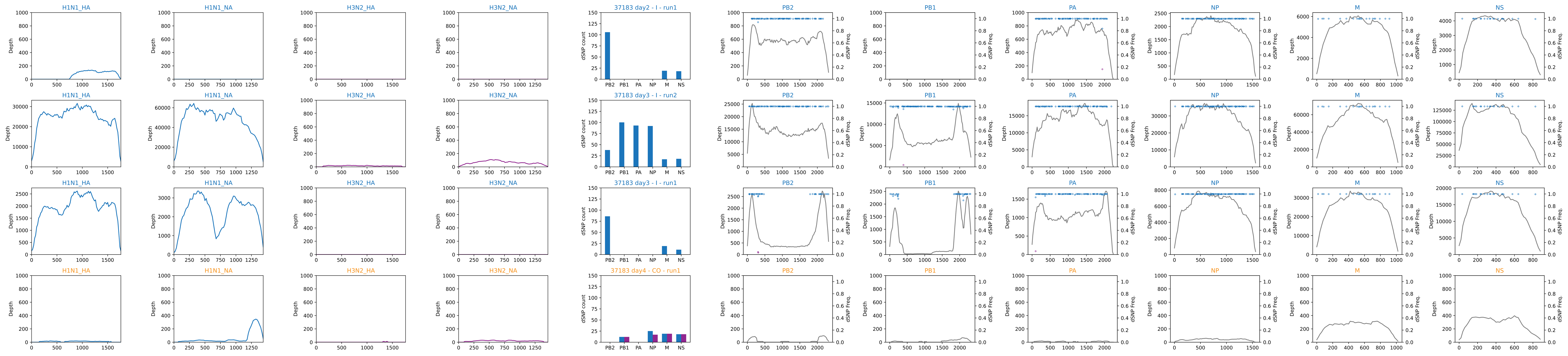
H1N1_HA
H1N1_NA
H3N2_HA
H3N2_NA
37183 day2 - I - run1
PB2
PB1
PA
NP
M
NS
37183 day3 - I - run2
37183 day3 - I - run1
37183 day4 - CO - run1
Depth
dSNP count
dSNP Freq.

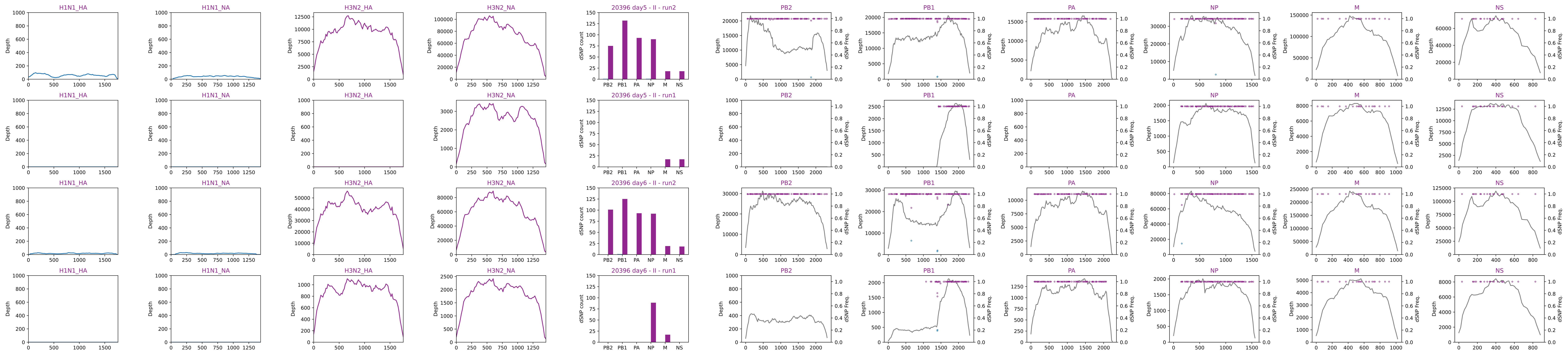
H1N1_HA
H1N1_NA
H3N2_HA
H3N2_NA
20396 day5 - II - run2
PB2
PB1
PA
NP
M
NS
20396 day5 - II - run1
20396 day6 - II - run2
20396 day6 - II - run1
Depth
dSNP count
dSNP Freq.

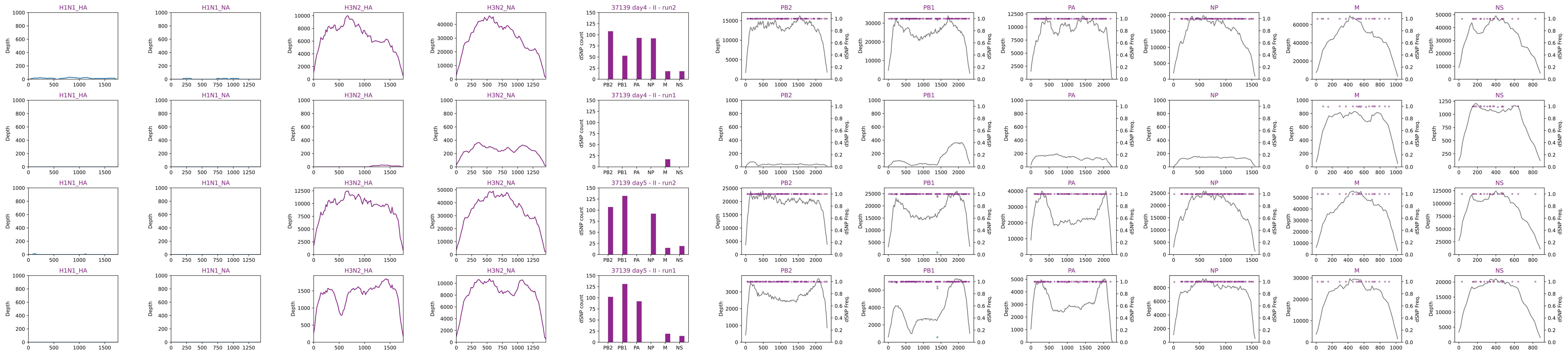
H1N1_HA
H1N1_NA
H3N2_HA
H3N2_NA
37139 day4 - II - run2
37139 day4 - II - run1
37139 day5 - II - run2
37139 day5 - II - run1
PB2
PB1
PA
NP
M
NS
Depth
dSNP count
dSNP Freq.

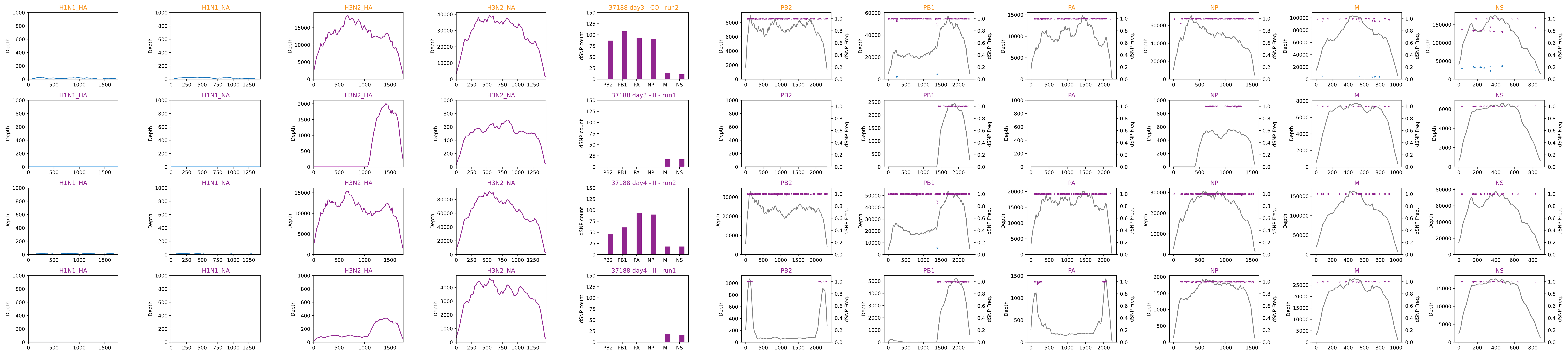
H1N1_HA
H1N1_NA
H3N2_HA
H3N2_NA
37188 day3 - CO - run2
PB2
PB1
PA
NP
M
NS
37188 day3 - II - run1
37188 day4 - II - run2
37188 day4 - II - run1
Depth
dSNP count
dSNP Freq.

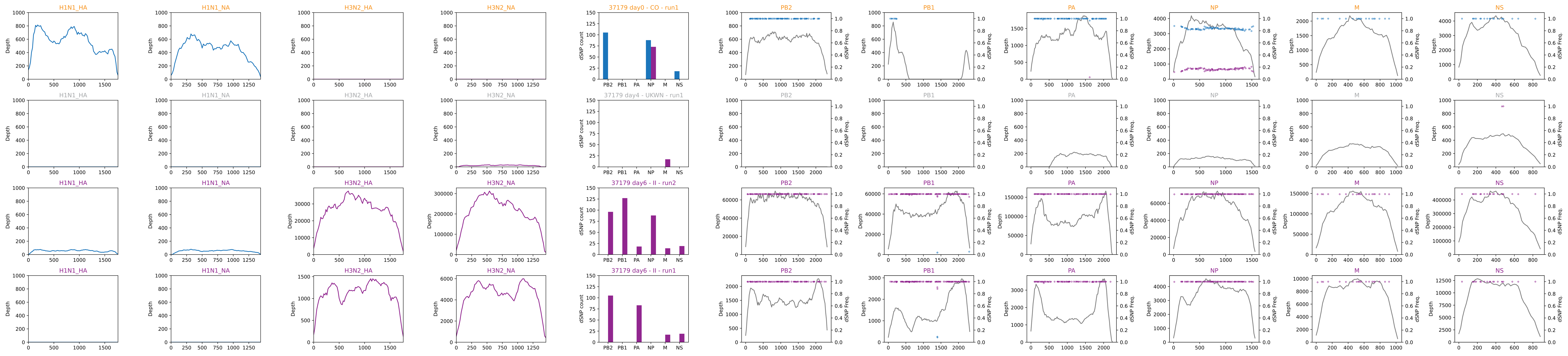
H1N1_HA
H1N1_NA
H3N2_HA
H3N2_NA
37179 day0 - CO - run1
37179 day4 - UKWN - run1
37179 day6 - II - run2
37179 day6 - II - run1
PB2
PB1
PA
NP
M
NS
Depth
dSNP count
dSNP Freq.

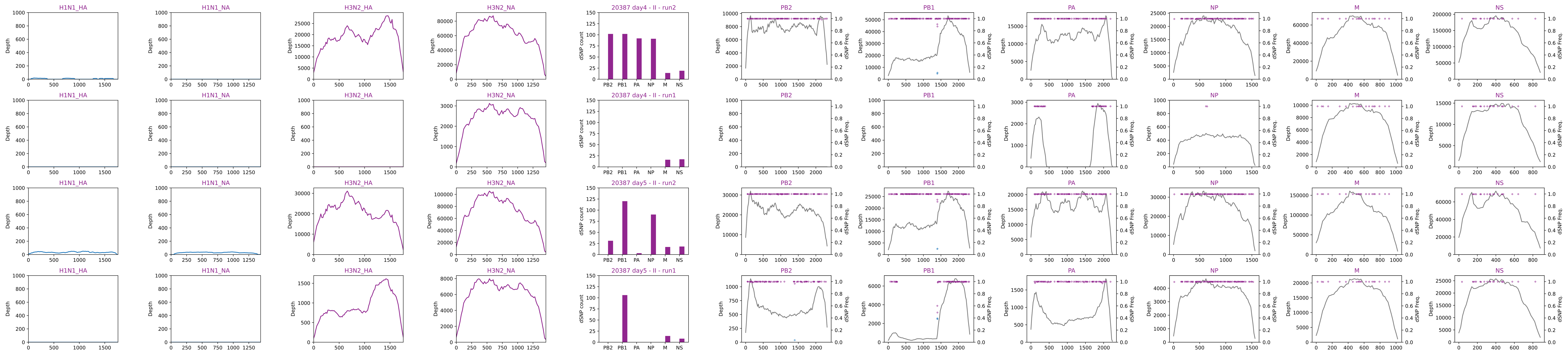

H1N1_HA
H1N1_NA
H3N2_HA
H3N2_NA
20387 day4 - II - run2
20387 day4 - II - run1
20387 day5 - II - run2
20387 day5 - II - run1
PB2
PB1
PA
NP
M
NS
Depth
dSNP count
dSNP Freq.

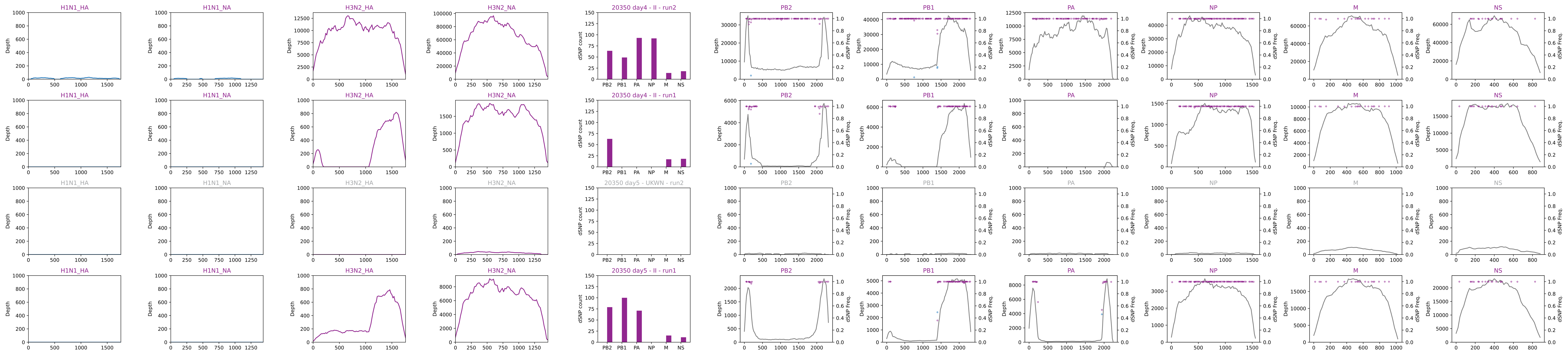

H1N1_HA
H1N1_NA
H3N2_HA
H3N2_NA
20350 day4 - II - run2
20350 day4 - II - run1
20350 day5 - UKWN - run2
20350 day5 - II - run1
PB2
PB1
PA
NP
M
NS
Depth
dSNP count
dSNP Freq.

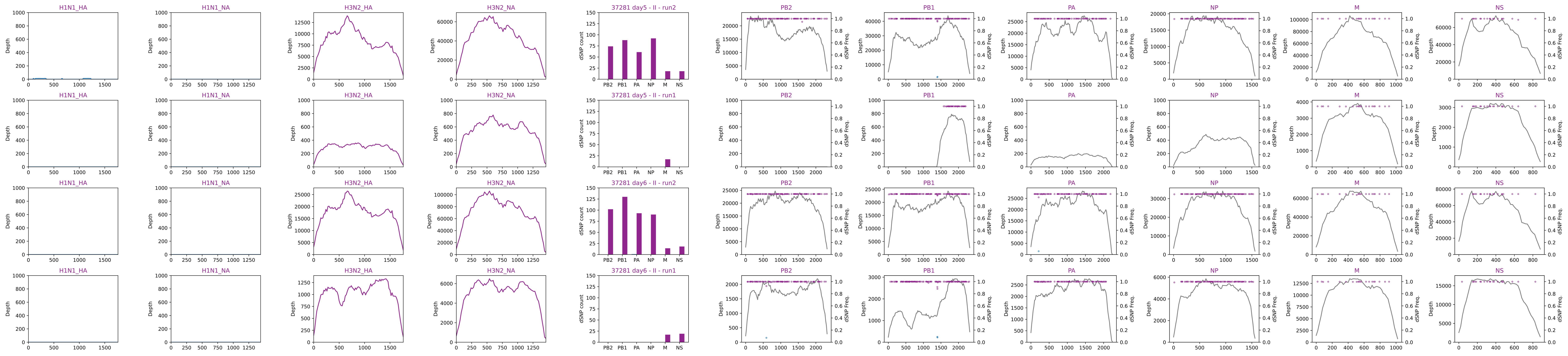
H1N1_HA
H1N1_NA
H3N2_HA
H3N2_NA
37281 day5 - II - run2
PB2
PB1
PA
NP
M
NS
H1N1_HA
H1N1_NA
H3N2_HA
H3N2_NA
37281 day5 - II - run1
PB2
PB1
PA
NP
M
NS
H1N1_HA
H1N1_NA
H3N2_HA
H3N2_NA
37281 day6 - II - run2
PB2
PB1
PA
NP
M
NS
H1N1_HA
H1N1_NA
H3N2_HA
H3N2_NA
37281 day6 - II - run1
PB2
PB1
PA
NP
M
NS
Depth
dSNP count
dSNP Freq.

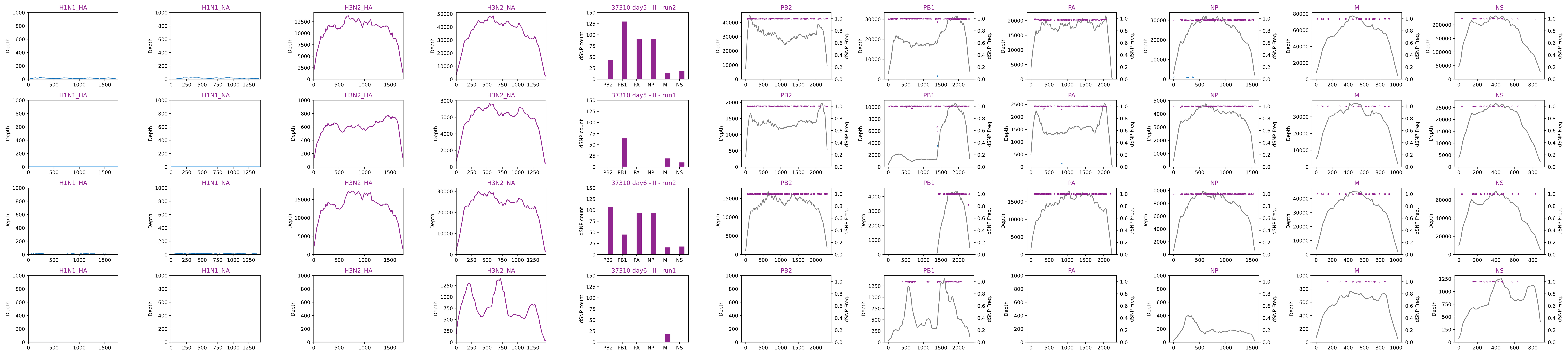

H1N1_HA
H1N1_NA
H3N2_HA
H3N2_NA
37310 day5 - II - run2
PB2
PB1
PA
NP
M
NS
H1N1_HA
H1N1_NA
H3N2_HA
H3N2_NA
37310 day5 - II - run1
PB2
PB1
PA
NP
M
NS
H1N1_HA
H1N1_NA
H3N2_HA
H3N2_NA
37310 day6 - II - run2
PB2
PB1
PA
NP
M
NS
H1N1_HA
H1N1_NA
H3N2_HA
H3N2_NA
37310 day6 - II - run1
PB2
PB1
PA
NP
M
NS
Depth
dSNP count
dSNP Freq.

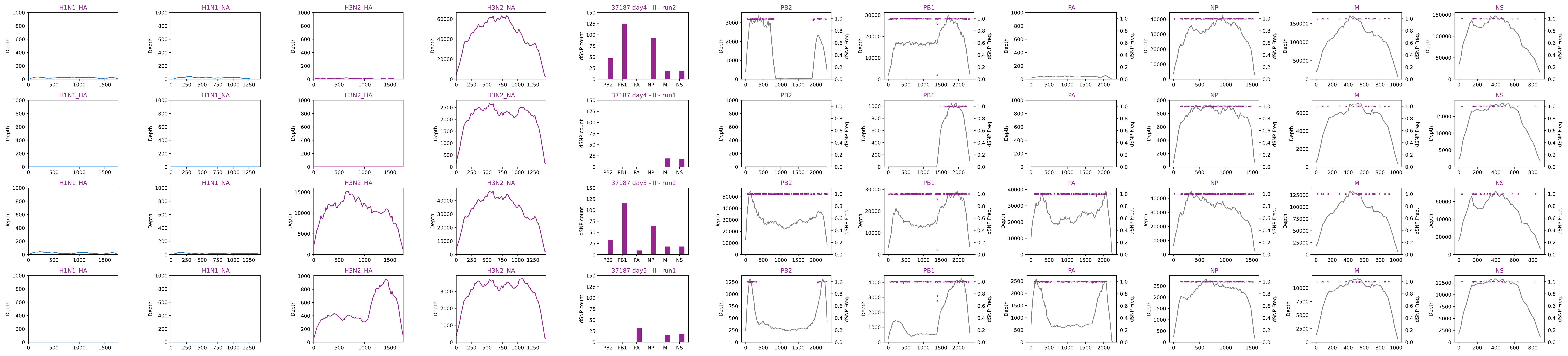
H1N1_HA
H1N1_NA
H3N2_HA
H3N2_NA
37187 day4 - II - run2
PB2
PB1
PA
NP
M
NS
37187 day4 - II - run1
37187 day5 - II - run2
37187 day5 - II - run1
Depth
dSNP count
dSNP Freq.

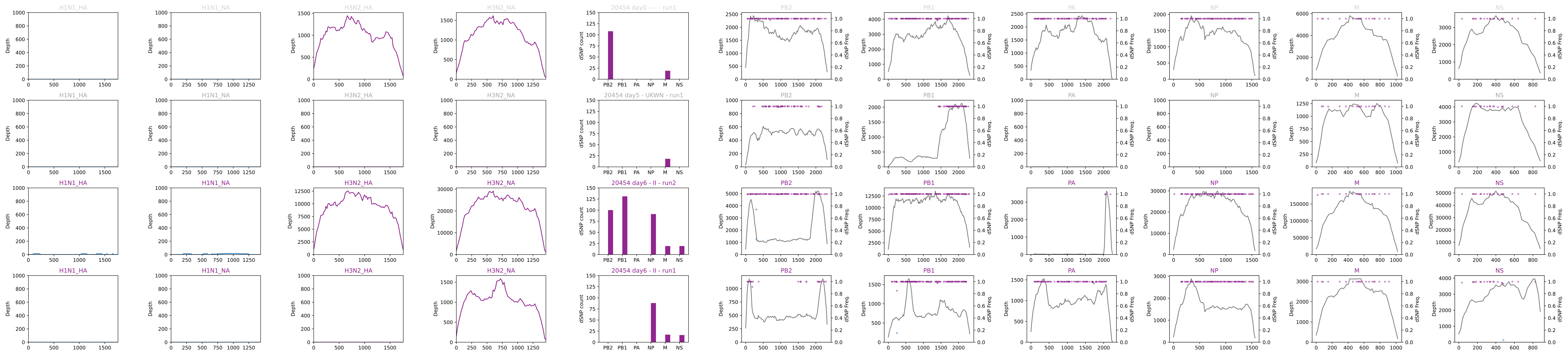
H1N1_HA
H1N1_NA
H3N2_HA
H3N2_NA
20454 day0 - -- - run1
PB2
PB1
PA
NP
M
NS
20454 day5 - UKWN - run1
20454 day6 - II - run2
20454 day6 - II - run1
Depth
dSNP count
dSNP Freq.

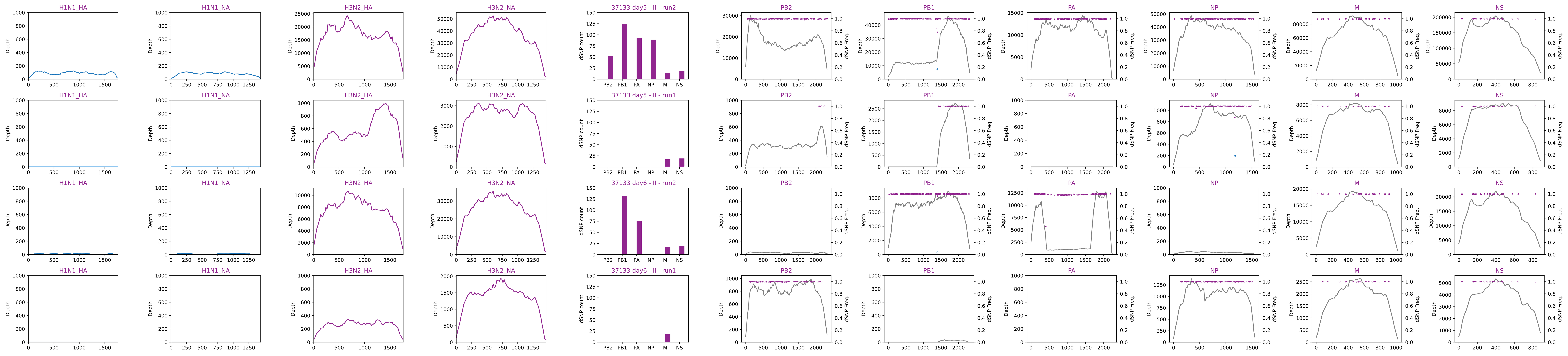
H1N1_HA
H1N1_NA
H3N2_HA
H3N2_NA
37133 day5 - II - run2
PB2
PB1
PA
NP
M
NS
37133 day5 - II - run1
37133 day6 - II - run2
37133 day6 - II - run1
Depth
dSNP count
dSNP Freq.
PB2 PB1 PA NP M NS

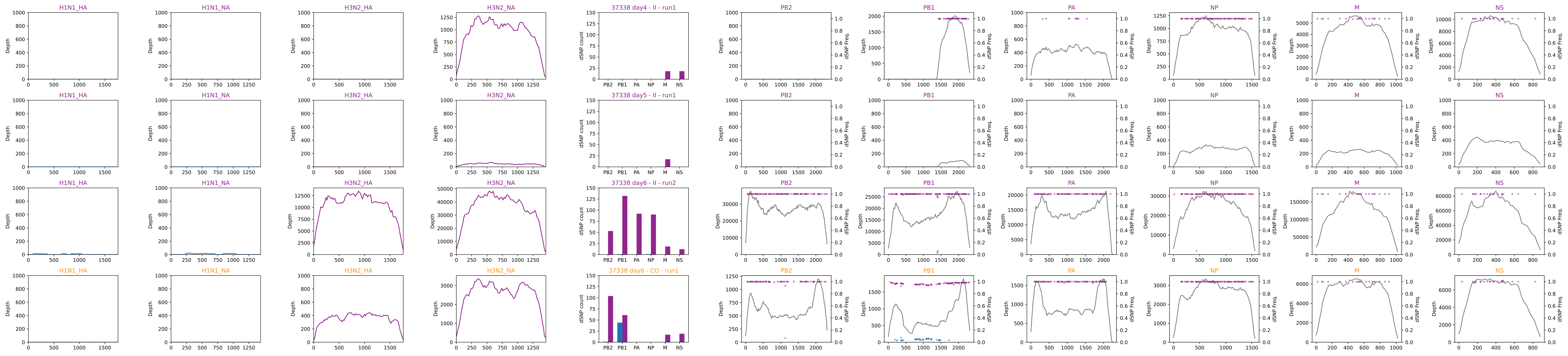
H1N1_HA
H1N1_NA
H3N2_HA
H3N2_NA
37338 day4 - II - run1
37338 day5 - II - run1
37338 day6 - II - run2
37338 day6 - CO - run1
PB2
PB1
PA
NP
M
NS
Depth
dSNP count
dSNP Freq.

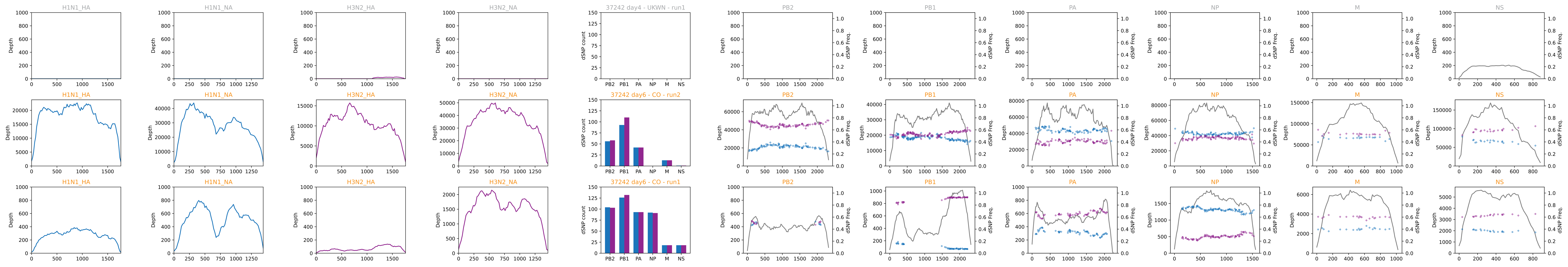

H1N1_HA
H1N1_NA
H3N2_HA
H3N2_NA
37242 day4 - UKWN - run1
PB2
PB1
PA
NP
M
NS
37242 day6 - CO - run2
37242 day6 - CO - run1
Depth
dSNP count
dSNP Freq.

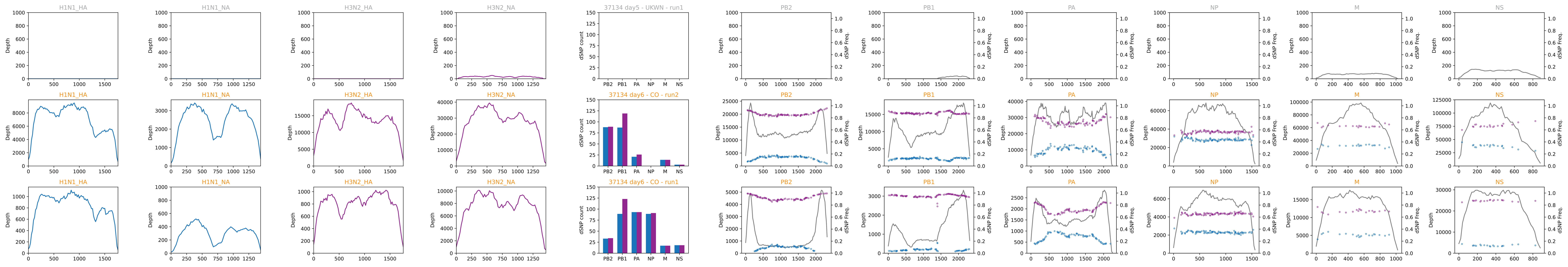
H1N1_HA
H1N1_NA
H3N2_HA
H3N2_NA
37134 day5 - UKWN - run1
PB2
PB1
PA
NP
M
NS
37134 day6 - CO - run2
37134 day6 - CO - run1
Depth
dSNP count
dSNP Freq.

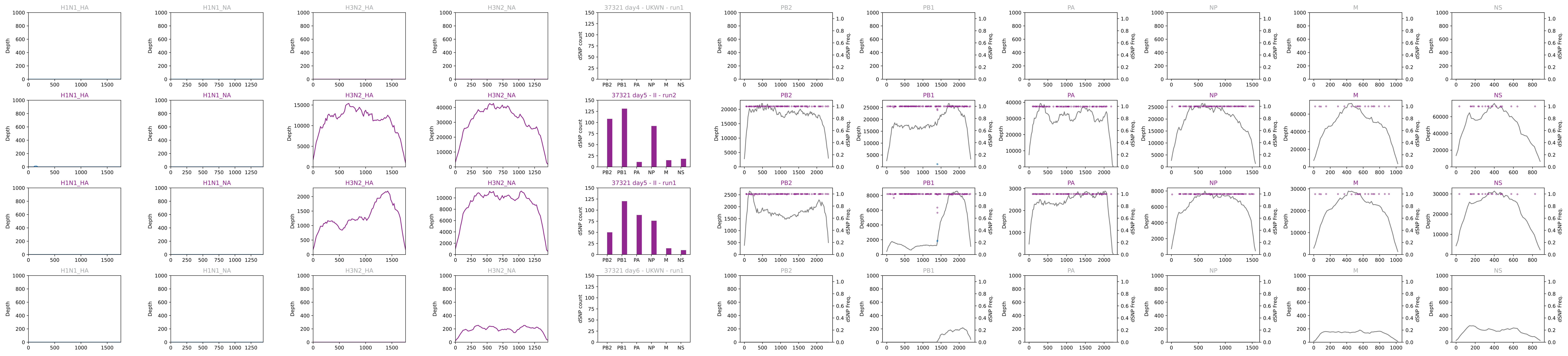
H1N1_HA
H1N1_NA
H3N2_HA
H3N2_NA
37321 day4 - UKWN - run1
PB2
PB1
PA
NP
M
NS
37321 day5 - II - run2
37321 day5 - II - run1
37321 day6 - UKWN - run1
Depth
dSNP count
dSNP Freq.

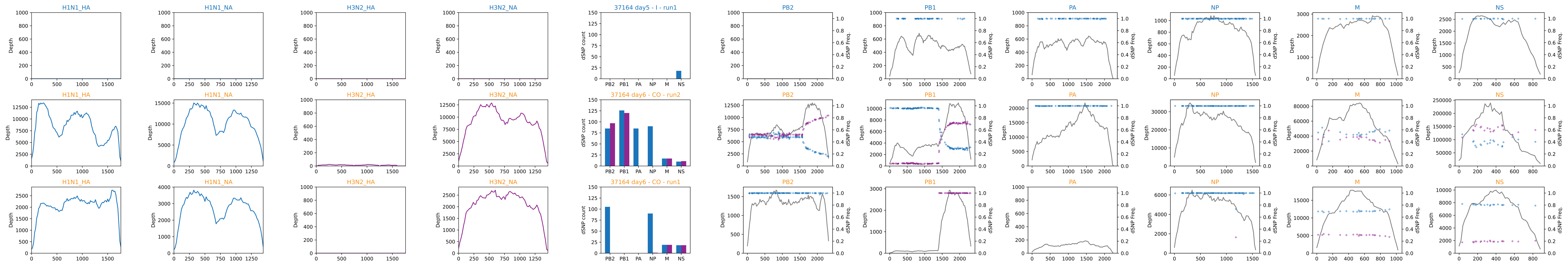
H1N1_HA
H1N1_NA
H3N2_HA
H3N2_NA
37164 day5 - I - run1
PB2
PB1
PA
NP
M
NS
37164 day6 - CO - run2
37164 day6 - CO - run1
Depth
dSNP count
dSNP Freq.

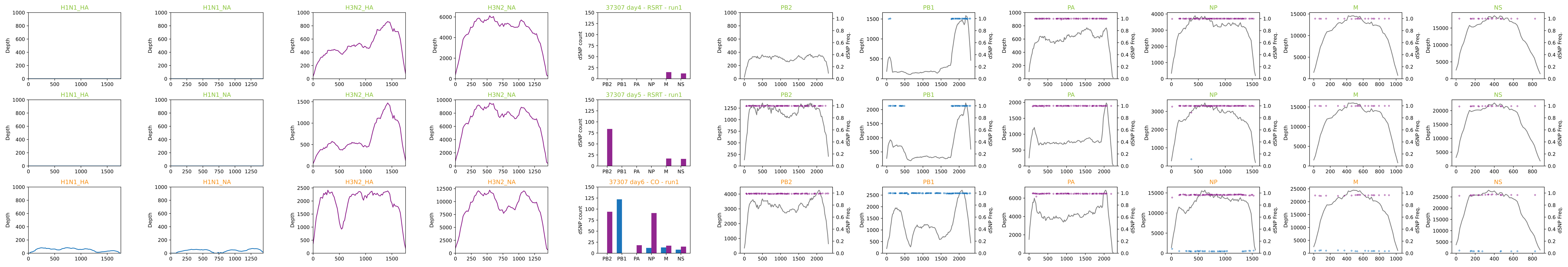

H1N1_HA
H1N1_NA
H3N2_HA
H3N2_NA
37307 day4 - RSRT - run1
PB2
PB1
PA
NP
M
NS
37307 day5 - RSRT - run1
37307 day6 - CO - run1
Depth
dSNP count
dSNP Freq.

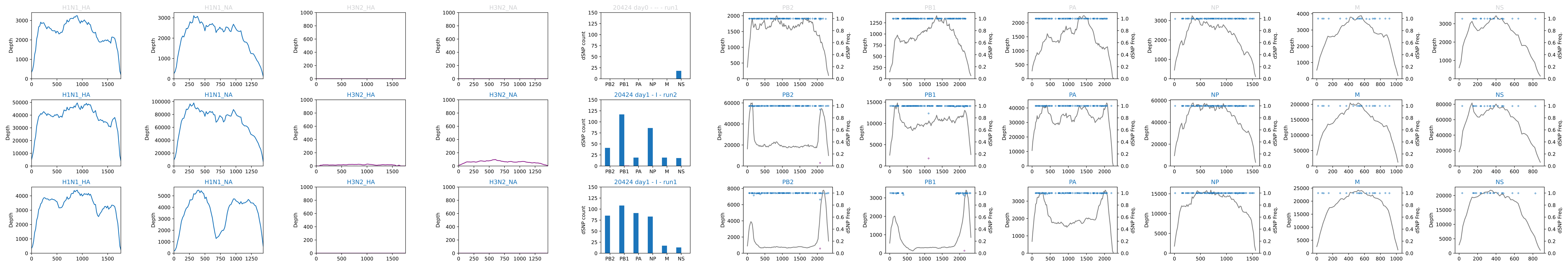

H1N1_HA
H1N1_NA
H3N2_HA
H3N2_NA
20424 day0 - - - run1
PB2
PB1
PA
NP
M
NS
20424 day1 - I - run2
20424 day1 - I - run1
Depth
dSNP count
dSNP Freq.

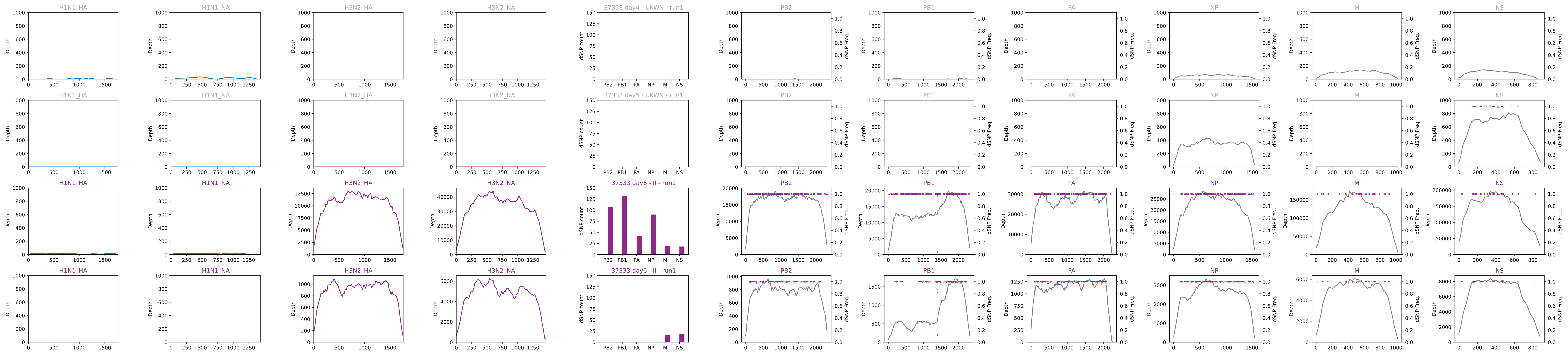
H1N1_HA
H1N1_NA
H3N2_HA
H3N2_NA
37333 day4 - UKWN - run1
37333 day5 - UKWN - run1
37333 day6 - II - run2
37333 day6 - II - run1
PB2
PB1
PA
NP
M
NS
Depth
dSNP count
dSNP Freq.

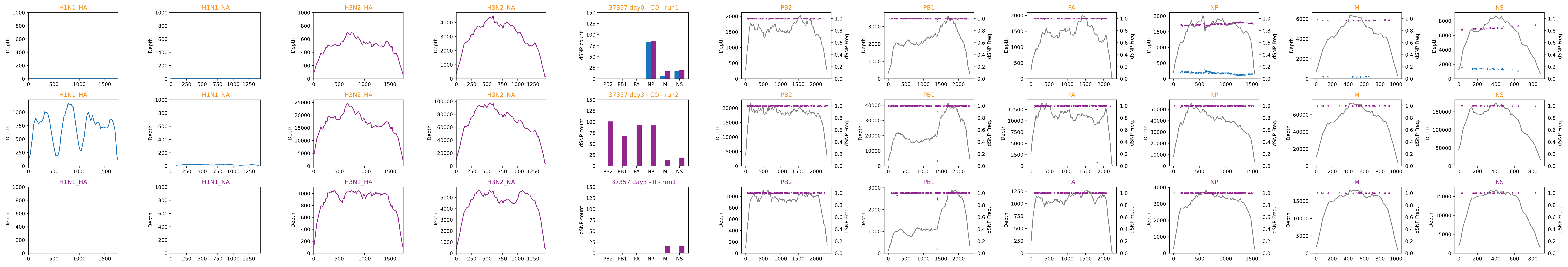

H1N1_HA
H1N1_NA
H3N2_HA
H3N2_NA
37357 day0 - CO - run1
37357 day3 - CO - run2
37357 day3 - II - run1
PB2
PB1
PA
NP
M
NS
Depth
dSNP count
dSNP Freq.

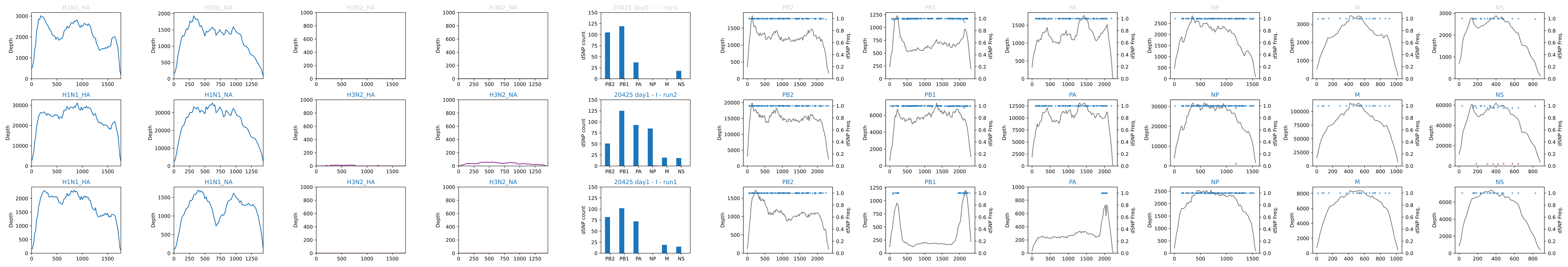
H1N1_HA
H1N1_NA
H3N2_HA
H3N2_NA
20425 day0 - -- - run1
PB2
PB1
PA
NP
M
NS
20425 day1 - I - run2
20425 day1 - I - run1
Depth
dSNP count
dSNP Freq.

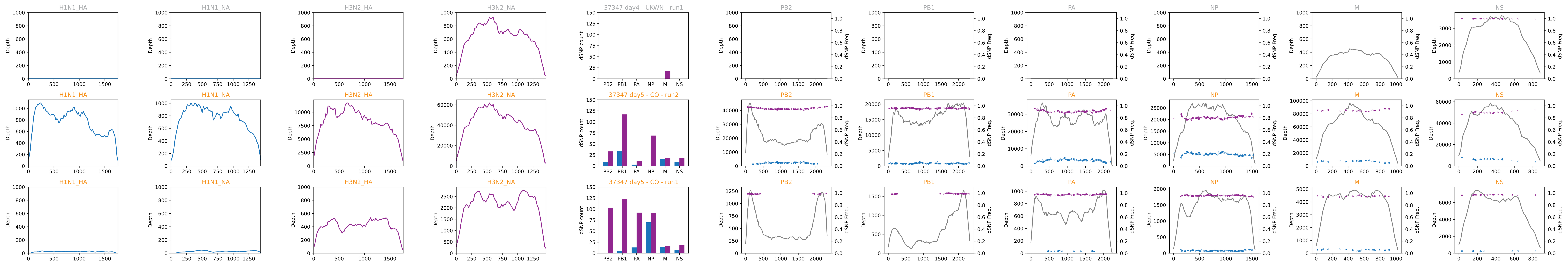

H1N1_HA
H1N1_NA
H3N2_HA
H3N2_NA
37347 day4 - UKWN - run1
37347 day5 - CO - run2
37347 day5 - CO - run1
PB2
PB1
PA
NP
M
NS
Depth
dSNP count
dSNP Freq.

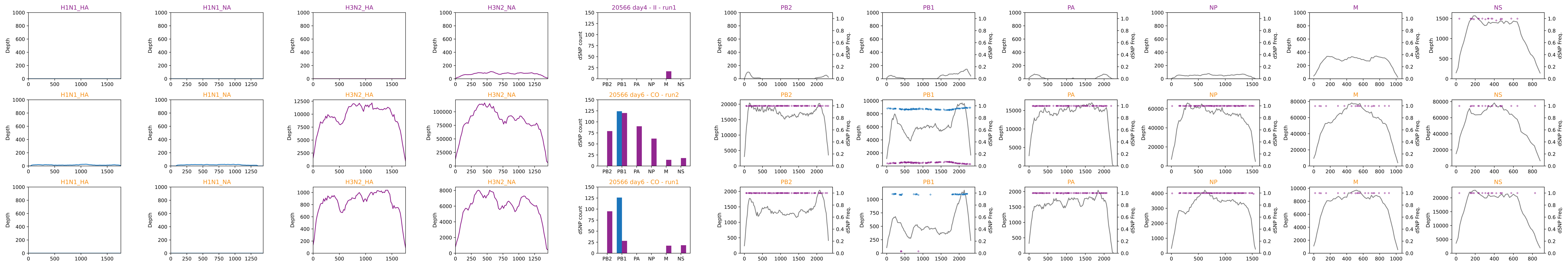

H1N1_HA
H1N1_NA
H3N2_HA
H3N2_NA
20566 day4 - II - run1
PB2
PB1
PA
NP
M
NS
20566 day6 - CO - run2
20566 day6 - CO - run1
Depth
dSNP count
dSNP Freq.

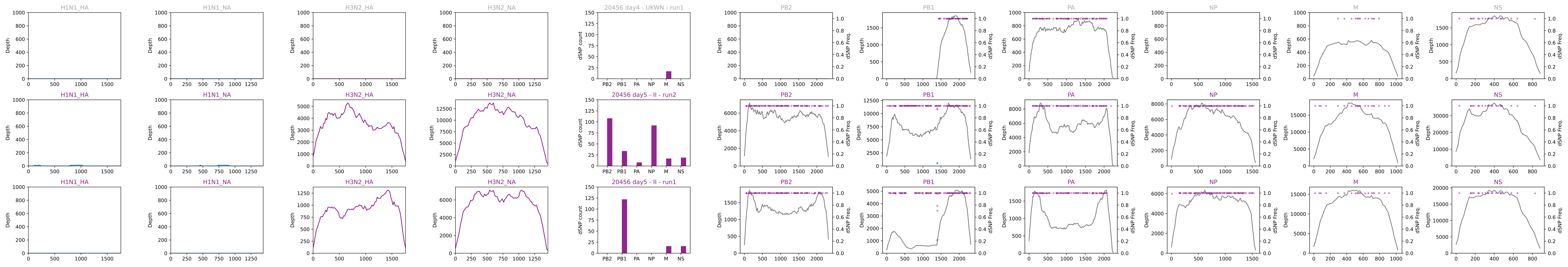
H1N1_HA
H1N1_NA
H3N2_HA
H3N2_NA
20456 day4 - UKWN - run1
PB2
PB1
PA
NP
M
NS
20456 day5 - II - run2
20456 day5 - II - run1
Depth
dSNP count
dSNP Freq.
PB2 PB1 PA NP M NS

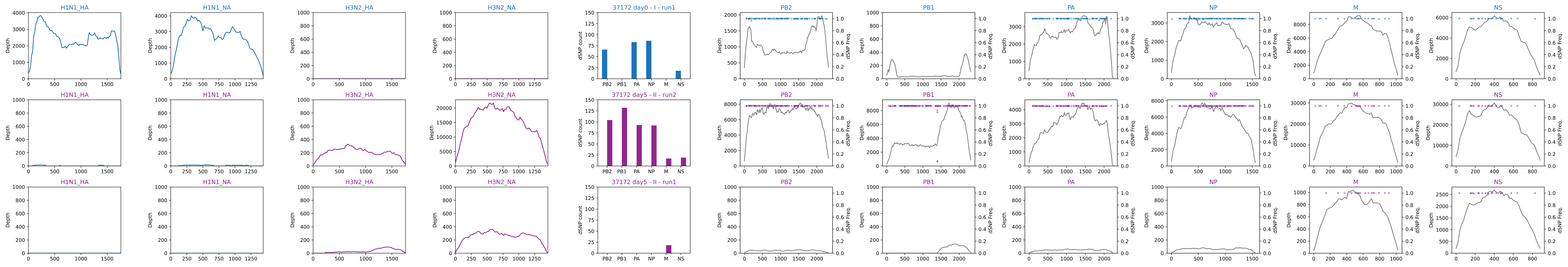

H1N1_HA
H1N1_NA
H3N2_HA
H3N2_NA
37172 day0 - I - run1
37172 day5 - II - run2
37172 day5 - II - run1
PB2
PB1
PA
NP
M
NS
Depth
dSNP count
dSNP Freq.

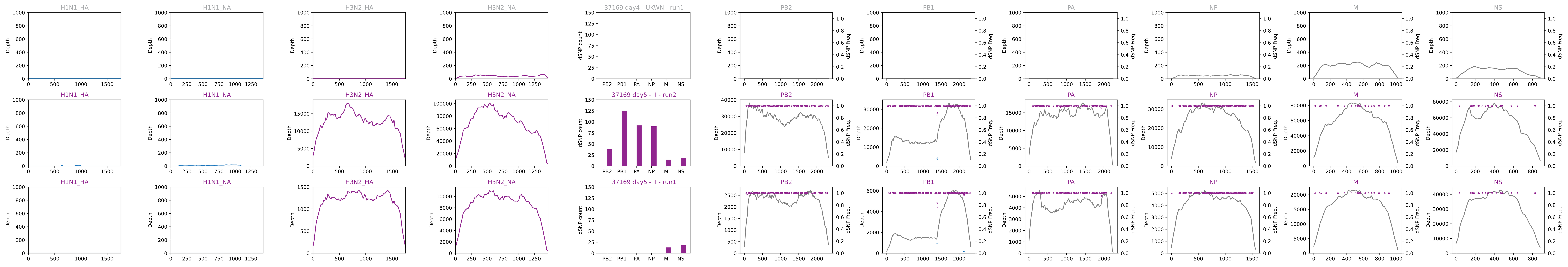
H1N1_HA
H1N1_NA
H3N2_HA
H3N2_NA
37169 day4 - UKWN - run1
PB2
PB1
PA
NP
M
NS
37169 day5 - II - run2
37169 day5 - II - run1
Depth
dSNP count
dSNP Freq.

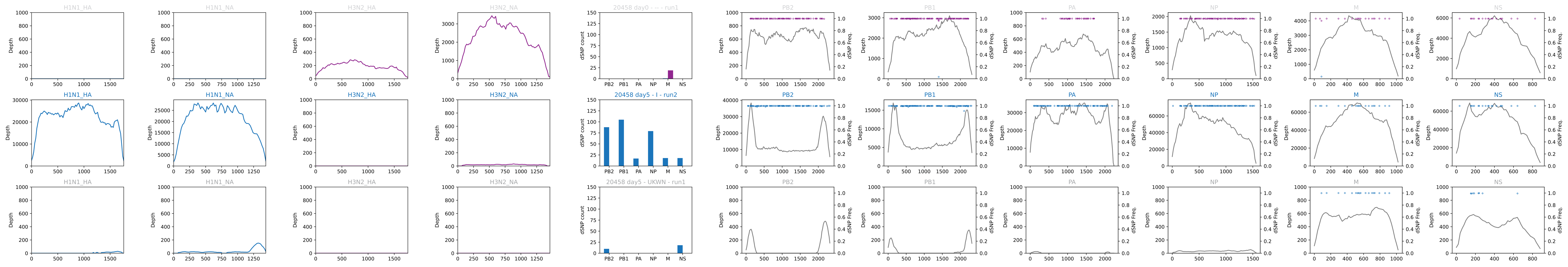
H1N1_HA
H1N1_NA
H3N2_HA
H3N2_NA
20458 day0 - --- - run1
PB2
PB1
PA
NP
M
NS
20458 day5 - I - run2
20458 day5 - UKWN - run1
Depth
dSNP count
dSNP Freq.

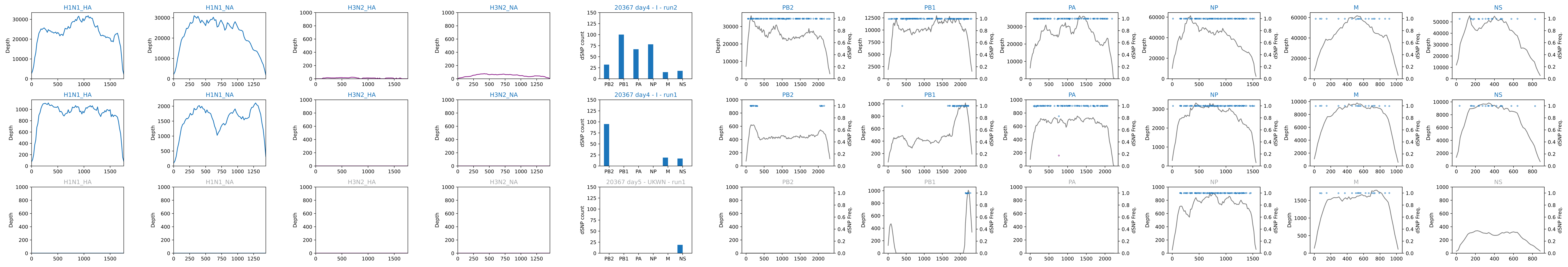

H1N1_HA
H1N1_NA
H3N2_HA
H3N2_NA
20367 day4 - I - run2
PB2
PB1
PA
NP
M
NS
20367 day4 - I - run1
20367 day5 - UKWN - run1
Depth
dSNP count
dSNP Freq.

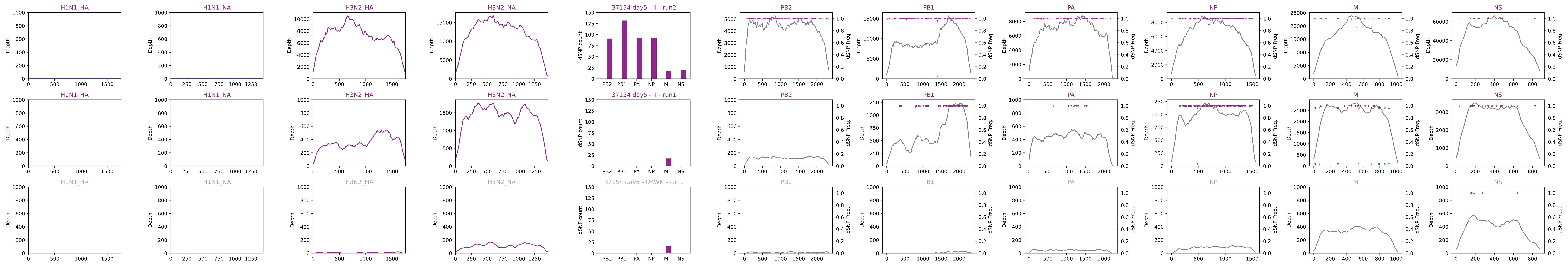
H1N1_HA
H1N1_NA
H3N2_HA
H3N2_NA
37154 day5 - II - run2
PB2
PB1
PA
NP
M
NS
37154 day5 - II - run1
37154 day6 - UKWN - run1
Depth
dSNP count
dSNP Freq.
PB2 PB1 PA NP M NS

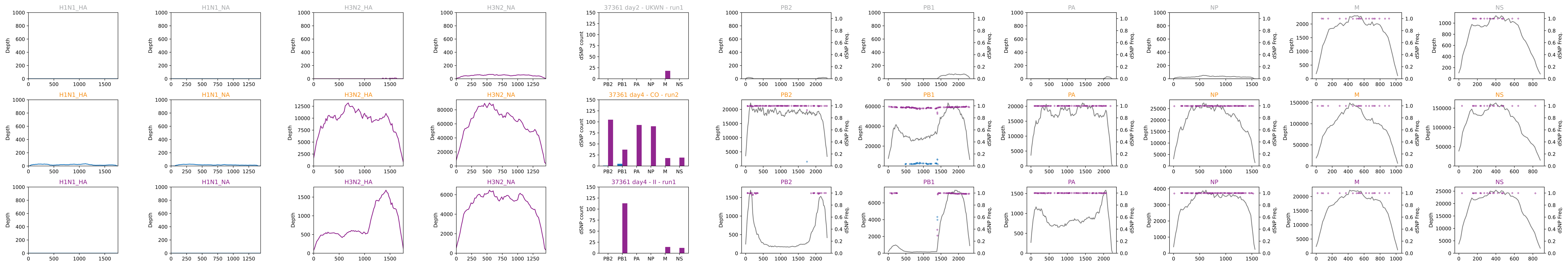
H1N1_HA
H1N1_NA
H3N2_HA
H3N2_NA
37361 day2 - UKWN - run1
PB2
PB1
PA
NP
M
NS
37361 day4 - CO - run2
37361 day4 - II - run1
Depth
dSNP count
dSNP Freq.

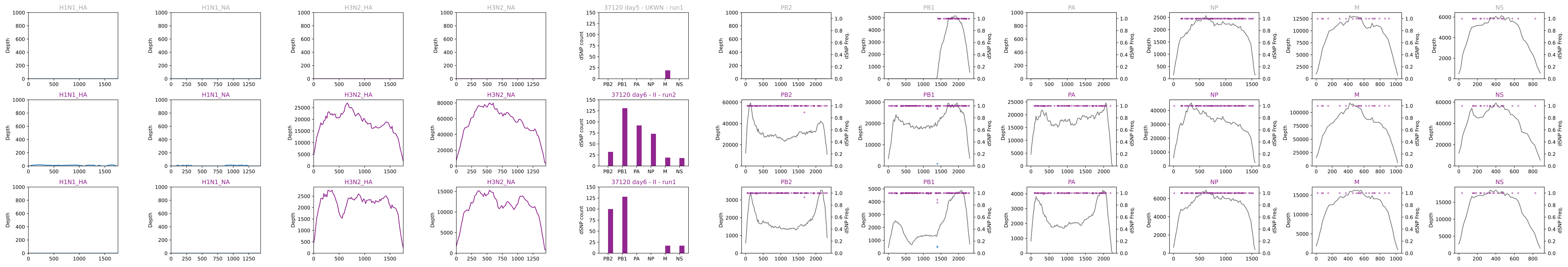

H1N1_HA
H1N1_NA
H3N2_HA
H3N2_NA
37120 day5 - UKWN - run1
PB2
PB1
PA
NP
M
NS
37120 day6 - II - run2
37120 day6 - II - run1
Depth
dSNP count
dSNP Freq.

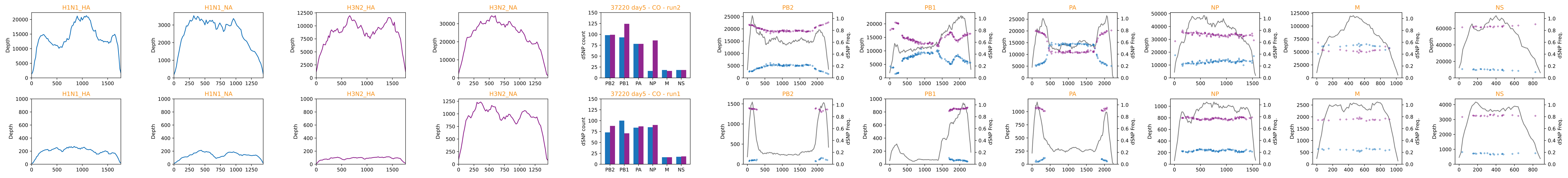

H1N1_HA
H1N1_NA
H3N2_HA
H3N2_NA
37220 day5 - CO - run2
37220 day5 - CO - run1
PB2
PB1
PA
NP
M
NS
Depth
dSNP count
dSNP Freq.

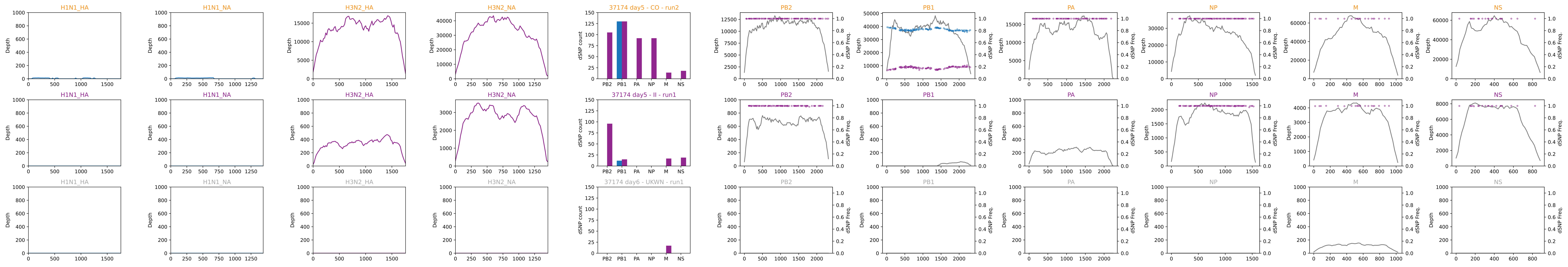
H1N1_HA
H1N1_NA
H3N2_HA
H3N2_NA
37174 day5 - CO - run2
PB2
PB1
PA
NP
M
NS
H1N1_HA
H1N1_NA
H3N2_HA
H3N2_NA
37174 day5 - II - run1
PB2
PB1
PA
NP
M
NS
H1N1_HA
H1N1_NA
H3N2_HA
H3N2_NA
37174 day6 - UKWN - run1
PB2
PB1
PA
NP
M
NS
Depth
dSNP count
dSNP Freq.

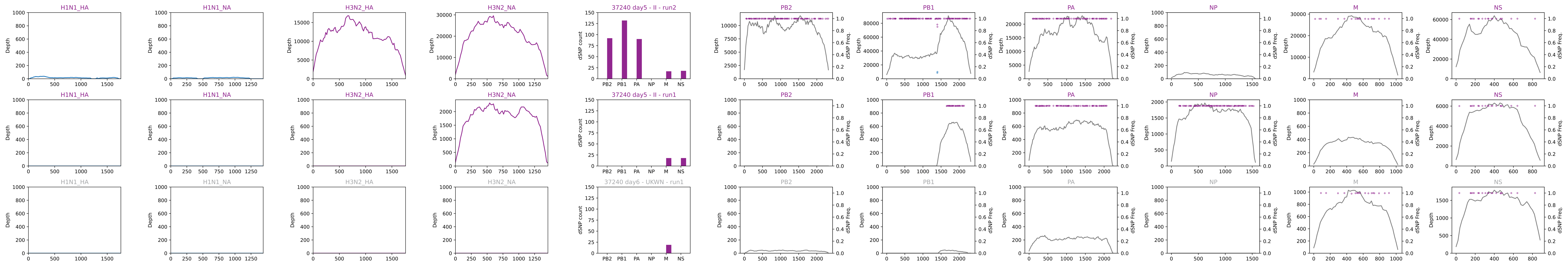
H1N1_HA
H1N1_NA
H3N2_HA
H3N2_NA
37240 day5 - II - run2
PB2
PB1
PA
NP
M
NS
37240 day5 - II - run1
37240 day6 - UKWN - run1
Depth
dSNP count
dSNP Freq.

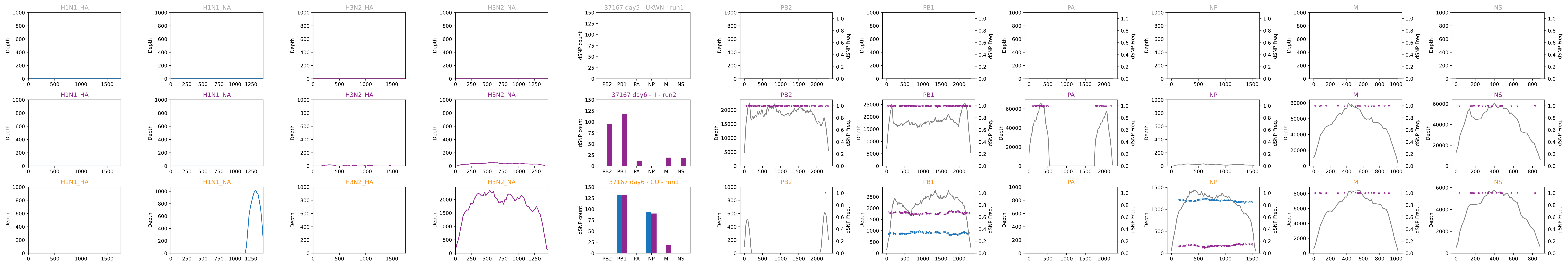

37167 day5 - UKWN - run1
37167 day6 - II - run2
37167 day6 - CO - run1
H1N1_HA
H1N1_NA
H3N2_HA
H3N2_NA
PB2
PB1
PA
NP
M
NS
Depth
dSNP count
dSNP Freq.

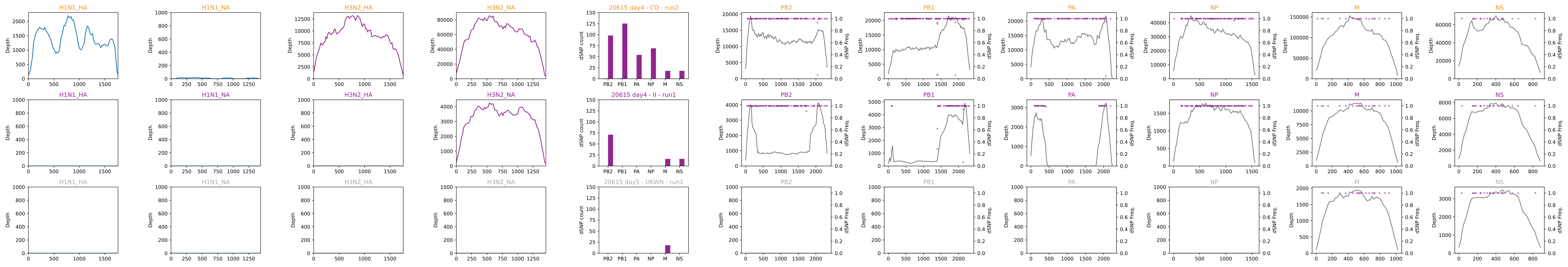
H1N1_HA
H1N1_NA
H3N2_HA
H3N2_NA
20615 day4 - CO - run2
PB2
PB1
PA
NP
M
NS
20615 day4 - II - run1
20615 day5 - UKWN - run1
Depth
dSNP count
dSNP Freq.

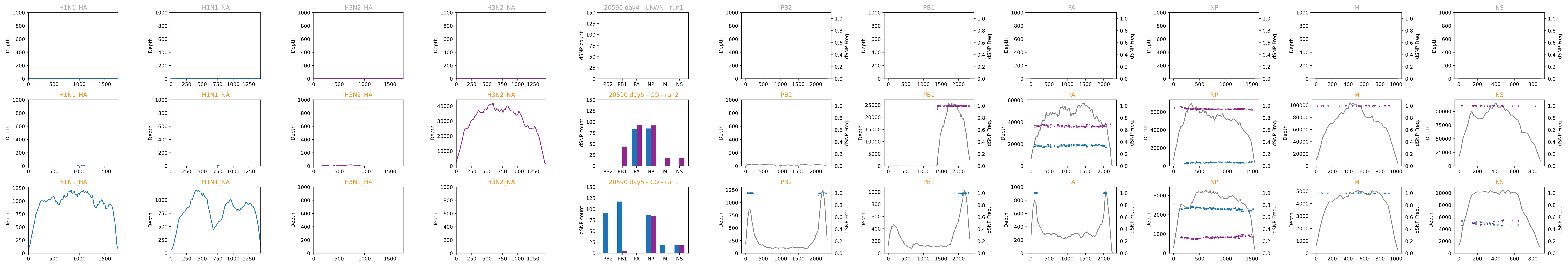
H1N1_HA
H1N1_NA
H3N2_HA
H3N2_NA
20590 day4 - UKWN - run1
20590 day5 - CO - run2
20590 day5 - CO - run1
PB2
PB1
PA
NP
M
NS
Depth
dSNP count
dSNP Freq.

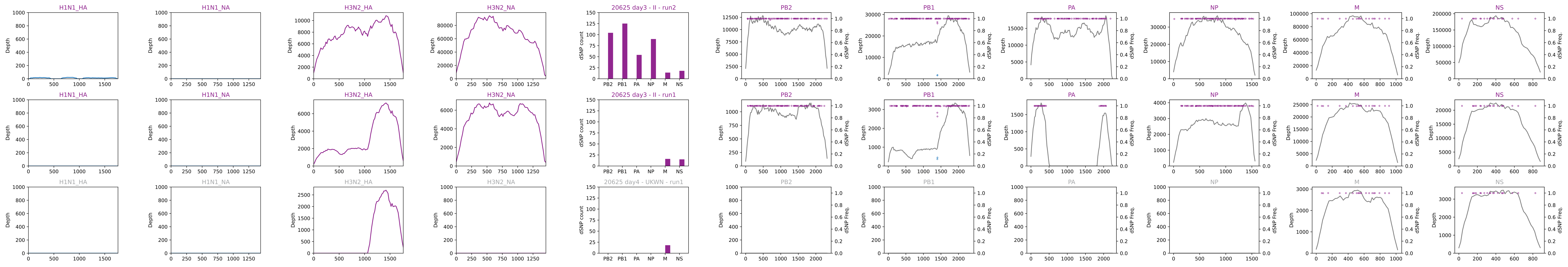

H1N1_HA
H1N1_NA
H3N2_HA
H3N2_NA
20625 day3 - II - run2
PB2
PB1
PA
NP
M
NS
H1N1_HA
H1N1_NA
H3N2_HA
H3N2_NA
20625 day3 - II - run1
PB2
PB1
PA
NP
M
NS
H1N1_HA
H1N1_NA
H3N2_HA
H3N2_NA
20625 day4 - UKWN - run1
PB2
PB1
PA
NP
M
NS
Depth
dSNP count
dSNP Freq.

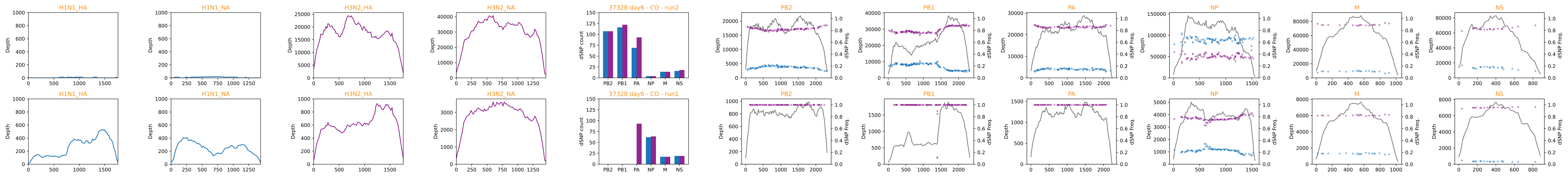
H1N1_HA
H1N1_NA
H3N2_HA
H3N2_NA
37328 day6 - CO - run2
PB2
PB1
PA
NP
M
NS
H1N1_HA
H1N1_NA
H3N2_HA
H3N2_NA
37328 day6 - CO - run1
PB2
PB1
PA
NP
M
NS
Depth
dSNP count
dSNP Freq.

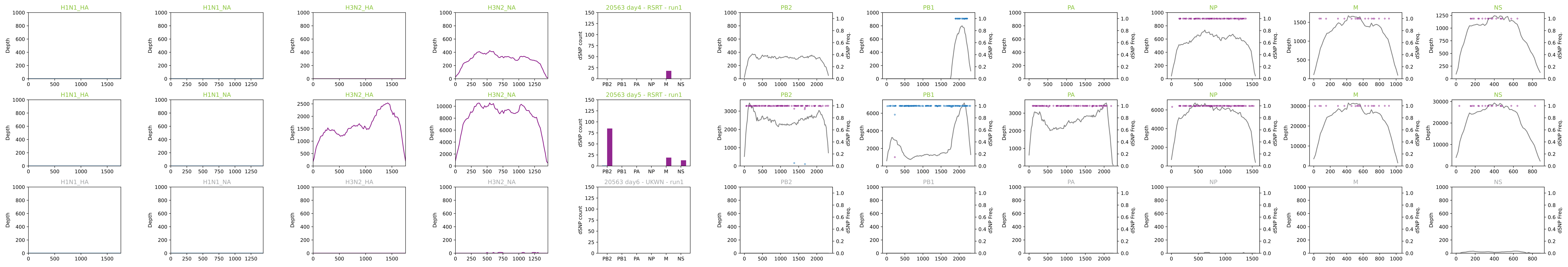

H1N1_HA
H1N1_NA
H3N2_HA
H3N2_NA
20563 day4 - RSRT - run1
20563 day5 - RSRT - run1
20563 day6 - UKWN - run1
PB2
PB1
PA
NP
M
NS
Depth
dSNP count
dSNP Freq.

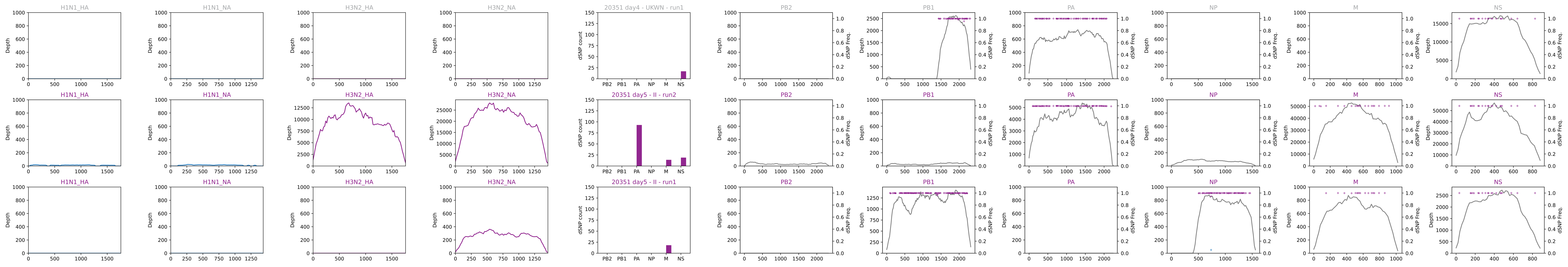

H1N1_HA
H1N1_NA
H3N2_HA
H3N2_NA
20351 day4 - UKWN - run1
PB2
PB1
PA
NP
M
NS
20351 day5 - II - run2
20351 day5 - II - run1
Depth
dSNP count
dSNP Freq.

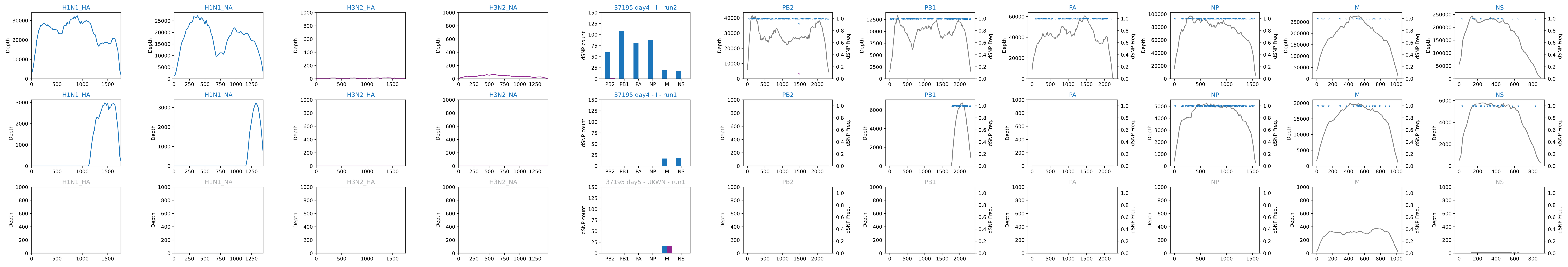
H1N1_HA
H1N1_NA
H3N2_HA
H3N2_NA
37195 day4 - I - run2
PB2
PB1
PA
NP
M
NS
37195 day4 - I - run1
37195 day5 - UKWN - run1
Depth
dSNP count
dSNP Freq.

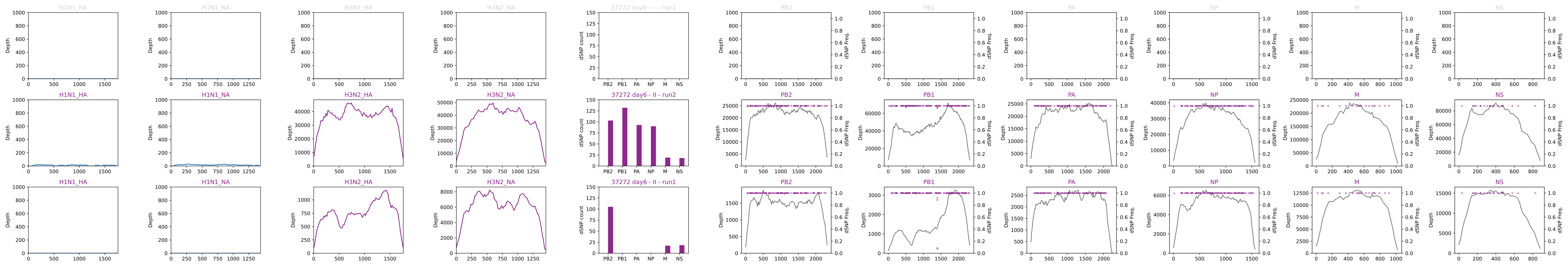
H1N1_HA
H1N1_NA
H3N2_HA
H3N2_NA
37272 day0 - -- - run1
PB2
PB1
PA
NP
M
NS
37272 day6 - II - run2
37272 day6 - II - run1
Depth
dSNP count
dSNP Freq.

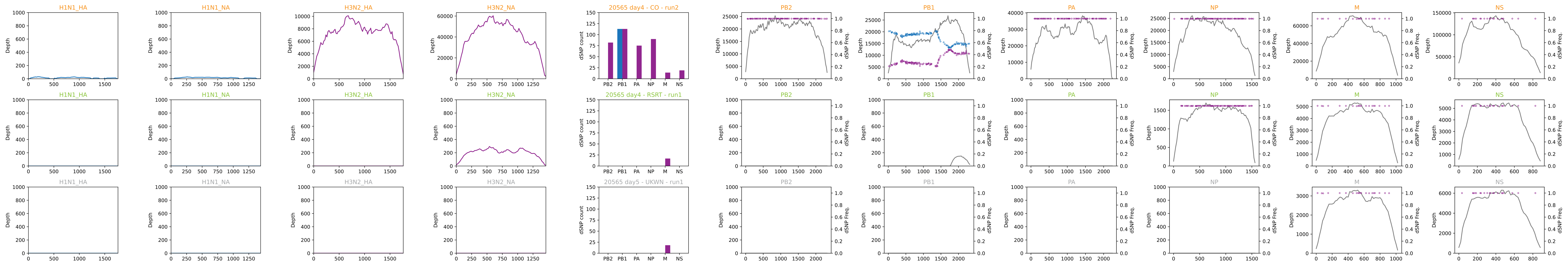

H1N1_HA
H1N1_NA
H3N2_HA
H3N2_NA
20565 day4 - CO - run2
PB2
PB1
PA
NP
M
NS
20565 day4 - RSRT - run1
20565 day5 - UKWN - run1
Depth
dSNP count
dSNP Freq.

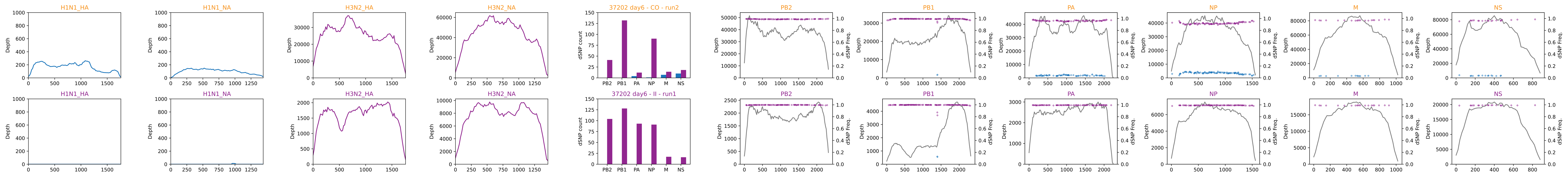
H1N1_HA
H1N1_NA
H3N2_HA
H3N2_NA
37202 day6 - CO - run2
PB2
PB1
PA
NP
M
NS
H1N1_HA
H1N1_NA
H3N2_HA
H3N2_NA
37202 day6 - II - run1
PB2
PB1
PA
NP
M
NS
Depth
dSNP count
dSNP Freq.

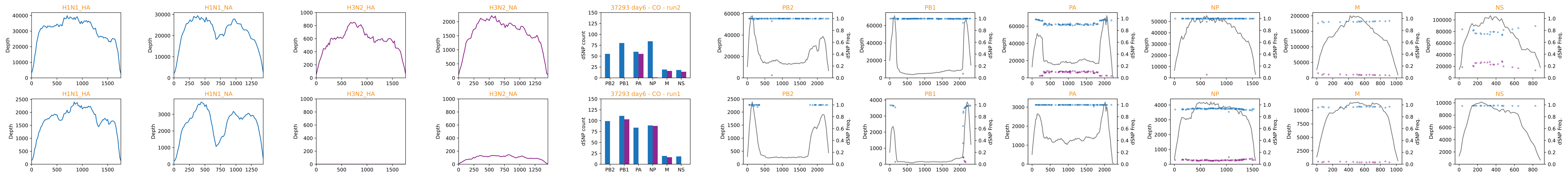
H1N1_HA
H1N1_NA
H3N2_HA
H3N2_NA
37293 day6 - CO - run2
PB2
PB1
PA
NP
M
NS
H1N1_HA
H1N1_NA
H3N2_HA
H3N2_NA
37293 day6 - CO - run1
PB2
PB1
PA
NP
M
NS
Depth
dSNP count
dSNP Freq.

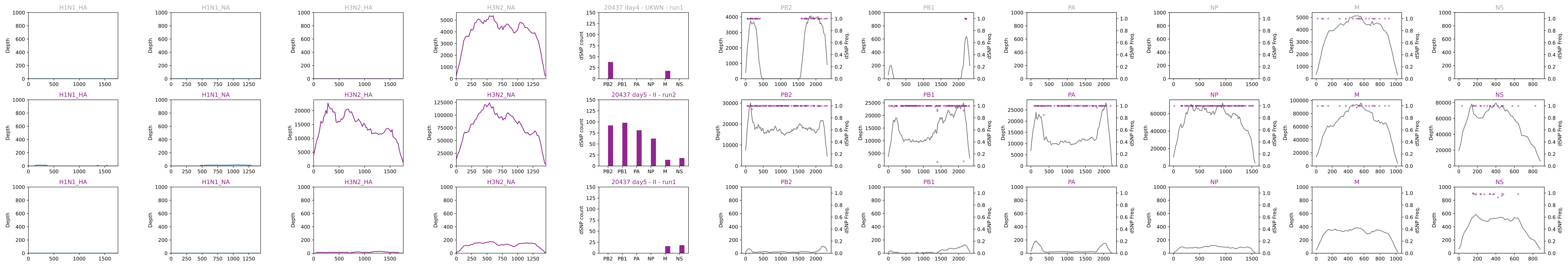
H1N1_HA
H1N1_NA
H3N2_HA
H3N2_NA
20437 day4 - UKWN - run1
PB2
PB1
PA
NP
M
NS
20437 day5 - II - run2
20437 day5 - II - run1
Depth
dSNP count
dSNP Freq.

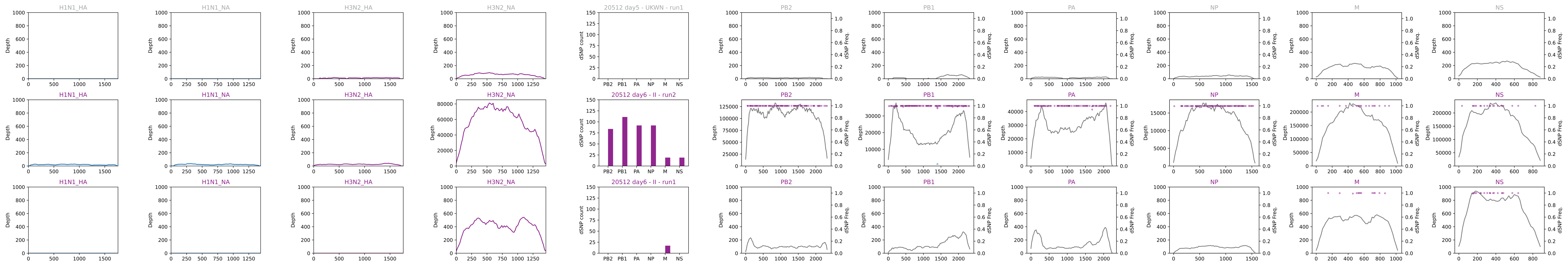

20512 day5 - UKWN - run1
20512 day6 - II - run2
20512 day6 - II - run1
H1N1_HA
H1N1_NA
H3N2_HA
H3N2_NA
PB2
PB1
PA
NP
M
NS
Depth
dSNP count
dSNP Freq.

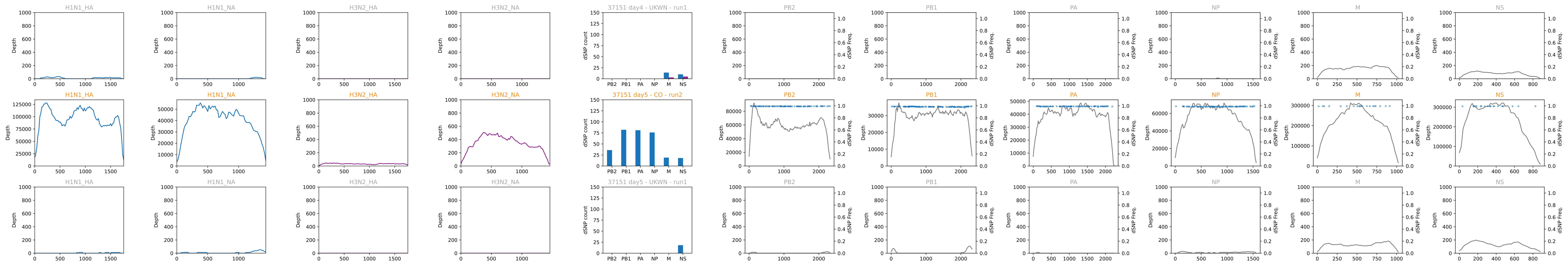
H1N1_HA
H1N1_NA
H3N2_HA
H3N2_NA
37151 day4 - UKWN - run1
PB2
PB1
PA
NP
M
NS
H1N1_HA
H1N1_NA
H3N2_HA
H3N2_NA
37151 day5 - CO - run2
PB2
PB1
PA
NP
M
NS
H1N1_HA
H1N1_NA
H3N2_HA
H3N2_NA
37151 day5 - UKWN - run1
PB2
PB1
PA
NP
M
NS
Depth
dSNP count
dSNP Freq.

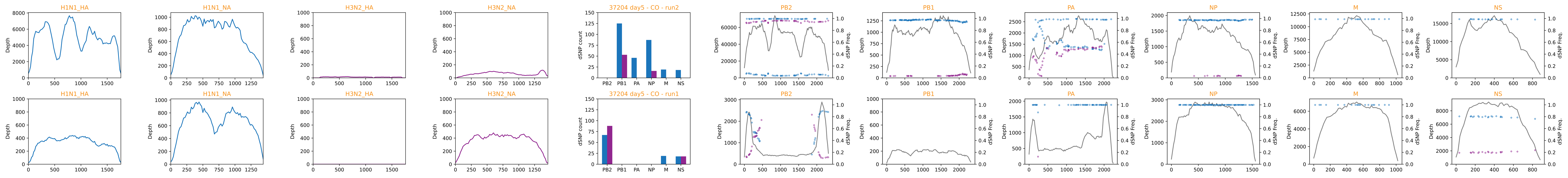
H1N1_HA
H1N1_NA
H3N2_HA
H3N2_NA
37204 day5 - CO - run2
PB2
PB1
PA
NP
M
NS
H1N1_HA
H1N1_NA
H3N2_HA
H3N2_NA
37204 day5 - CO - run1
PB2
PB1
PA
NP
M
NS
Depth
dSNP count
dSNP Freq.

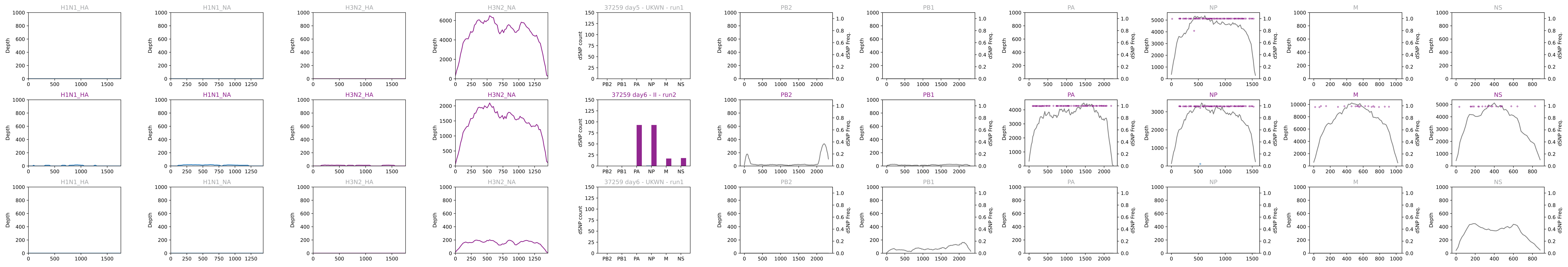
37259 day5 - UKWN - run1
37259 day6 - II - run2
37259 day6 - UKWN - run1
H1N1_HA
H1N1_NA
H3N2_HA
H3N2_NA
PB2
PB1
PA
NP
M
NS
Depth
dSNP count
dSNP Freq.

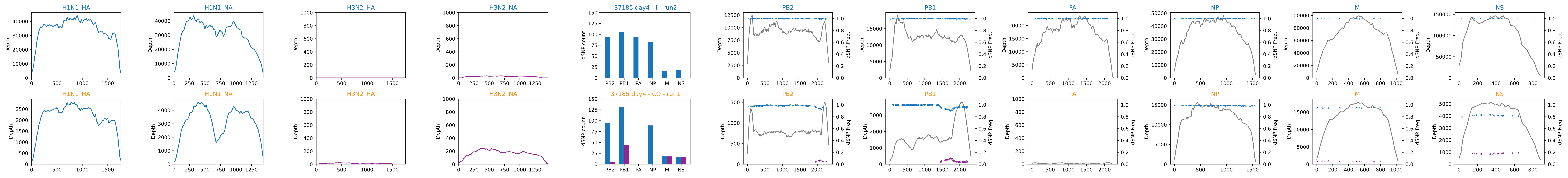
H1N1_HA
H1N1_NA
H3N2_HA
H3N2_NA
37185 day4 - I - run2
PB2
PB1
PA
NP
M
NS
H1N1_HA
H1N1_NA
H3N2_HA
H3N2_NA
37185 day4 - CO - run1
PB2
PB1
PA
NP
M
NS
Depth
dSNP count
dSNP Freq.

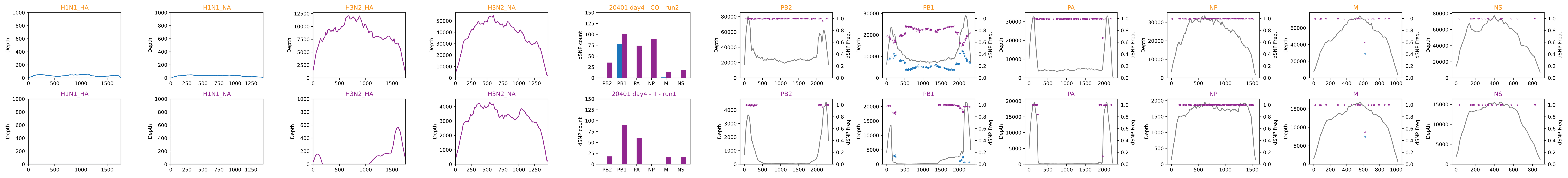
H1N1_HA
H1N1_NA
H3N2_HA
H3N2_NA
20401 day4 - CO - run2
PB2
PB1
PA
NP
M
NS
H1N1_HA
H1N1_NA
H3N2_HA
H3N2_NA
20401 day4 - II - run1
PB2
PB1
PA
NP
M
NS
Depth
dSNP count
dSNP Freq.

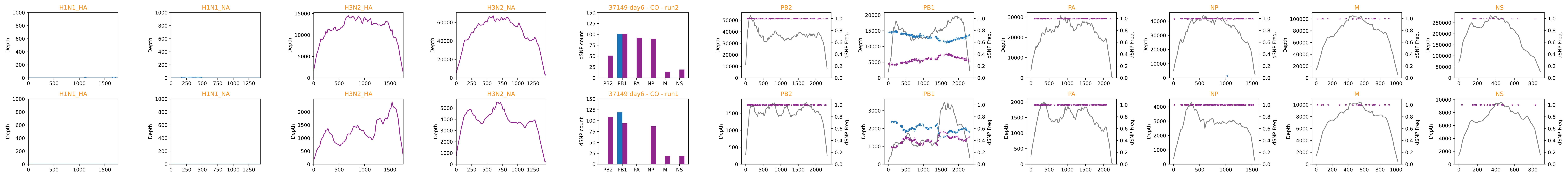
H1N1_HA
H1N1_NA
H3N2_HA
H3N2_NA
37149 day6 - CO - run2
PB2
PB1
PA
NP
M
NS
H1N1_HA
H1N1_NA
H3N2_HA
H3N2_NA
37149 day6 - CO - run1
PB2
PB1
PA
NP
M
NS
Depth
dSNP count
dSNP Freq.

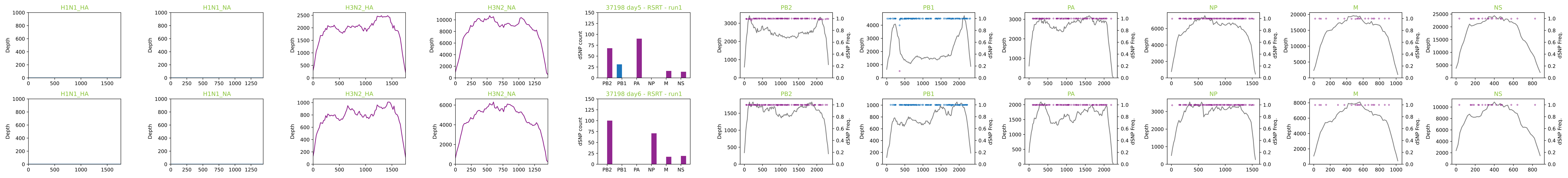
H1N1_HA
H1N1_NA
H3N2_HA
H3N2_NA
37198 day5 - RSRT - run1
PB2
PB1
PA
NP
M
NS
H1N1_HA
H1N1_NA
H3N2_HA
H3N2_NA
37198 day6 - RSRT - run1
PB2
PB1
PA
NP
M
NS
Depth
dSNP count
dSNP Freq.

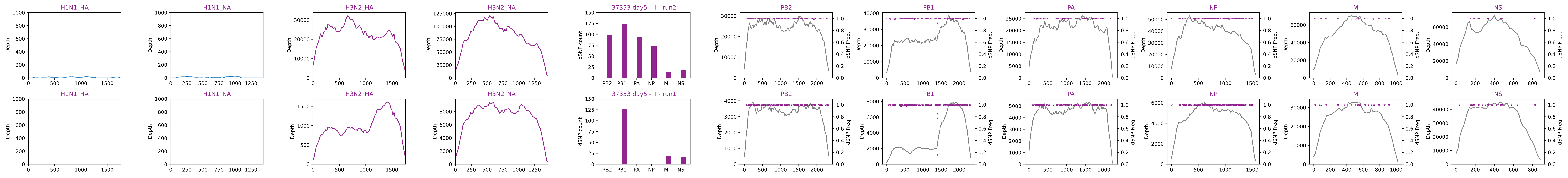
H1N1_HA
H1N1_NA
H3N2_HA
H3N2_NA
37353 day5 - II - run2
PB2
PB1
PA
NP
M
NS
H1N1_HA
H1N1_NA
H3N2_HA
H3N2_NA
37353 day5 - II - run1
PB2
PB1
PA
NP
M
NS
Depth
dSNP count
dSNP Freq.

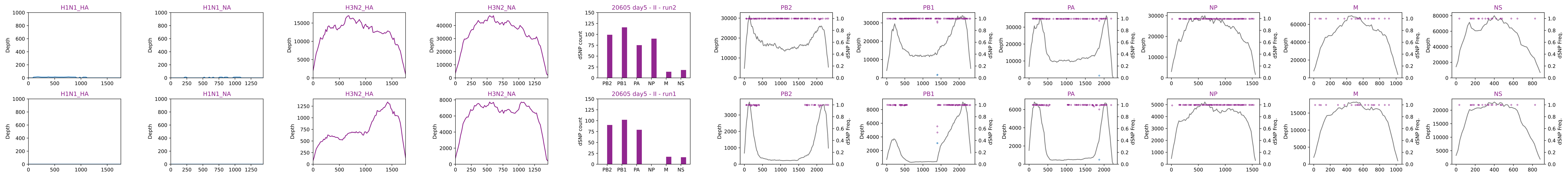
H1N1_HA
H1N1_NA
H3N2_HA
H3N2_NA
20605 day5 - II - run2
PB2
PB1
PA
NP
M
NS
H1N1_HA
H1N1_NA
H3N2_HA
H3N2_NA
20605 day5 - II - run1
PB2
PB1
PA
NP
M
NS
Depth
dSNP count
dSNP Freq.

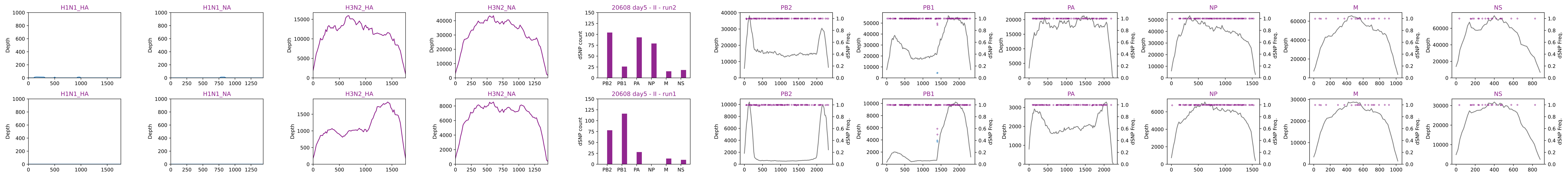
H1N1_HA
H1N1_NA
H3N2_HA
H3N2_NA
20608 day5 - II - run2
PB2
PB1
PA
NP
M
NS
20608 day5 - II - run1
Depth
dSNP count
dSNP Freq.

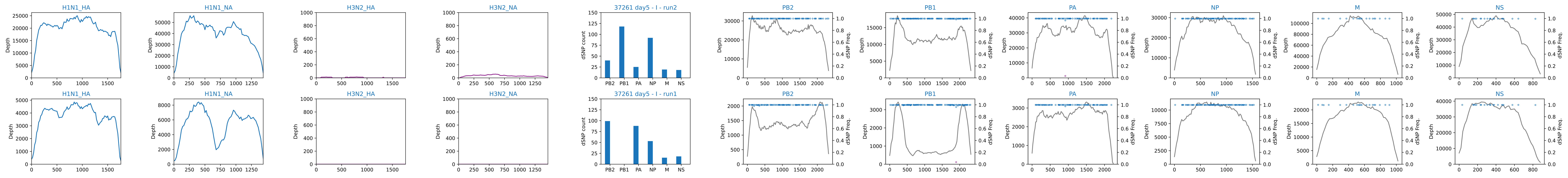
H1N1_HA
H1N1_NA
H3N2_HA
H3N2_NA
37261 day5 - I - run2
PB2
PB1
PA
NP
M
NS
H1N1_HA
H1N1_NA
H3N2_HA
H3N2_NA
37261 day5 - I - run1
PB2
PB1
PA
NP
M
NS
Depth
dSNP count
dSNP Freq.

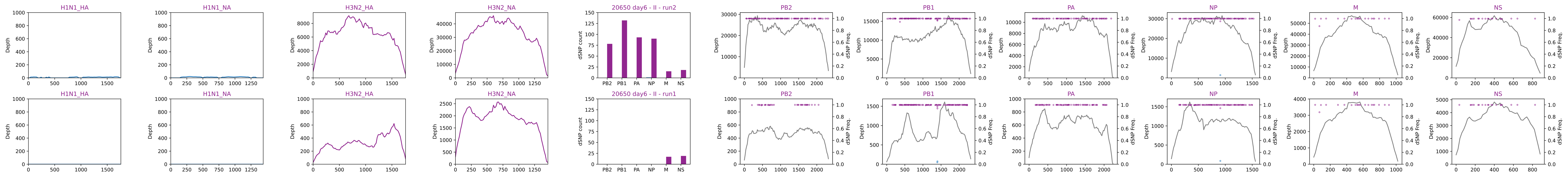
H1N1_HA
H1N1_NA
H3N2_HA
H3N2_NA
20650 day6 - II - run2
PB2
PB1
PA
NP
M
NS
20650 day6 - II - run1
Depth
dSNP count
dSNP Freq.

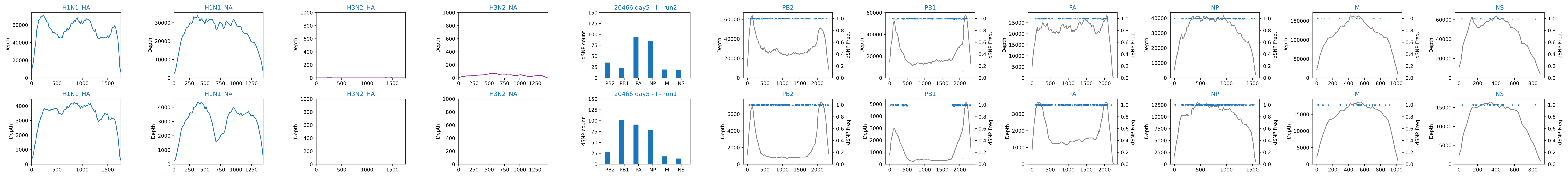
H1N1_HA
H1N1_NA
H3N2_HA
H3N2_NA
20466 day5 - I - run2
PB2
PB1
PA
NP
M
NS
H1N1_HA
H1N1_NA
H3N2_HA
H3N2_NA
20466 day5 - I - run1
PB2
PB1
PA
NP
M
NS
Depth
dSNP count
dSNP Freq.

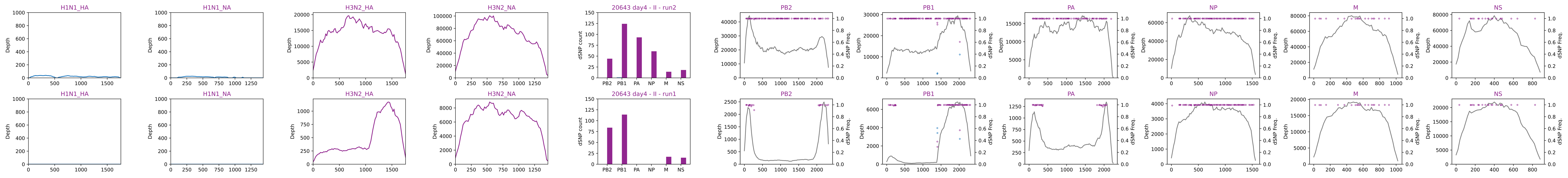
H1N1_HA
H1N1_NA
H3N2_HA
H3N2_NA
20643 day4 - II - run2
PB2
PB1
PA
NP
M
NS
H1N1_HA
H1N1_NA
H3N2_HA
H3N2_NA
20643 day4 - II - run1
PB2
PB1
PA
NP
M
NS
Depth
dSNP count
dSNP Freq.

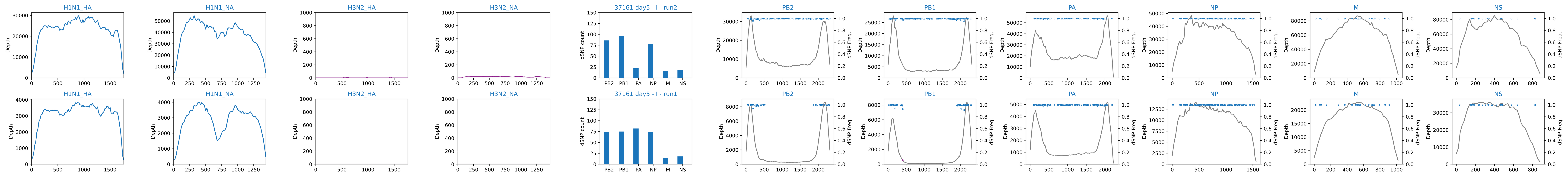
H1N1_HA
H1N1_NA
H3N2_HA
H3N2_NA
37161 day5 - I - run2
PB2
PB1
PA
NP
M
NS
H1N1_HA
H1N1_NA
H3N2_HA
H3N2_NA
37161 day5 - I - run1
PB2
PB1
PA
NP
M
NS
Depth
dSNP count
dSNP Freq.

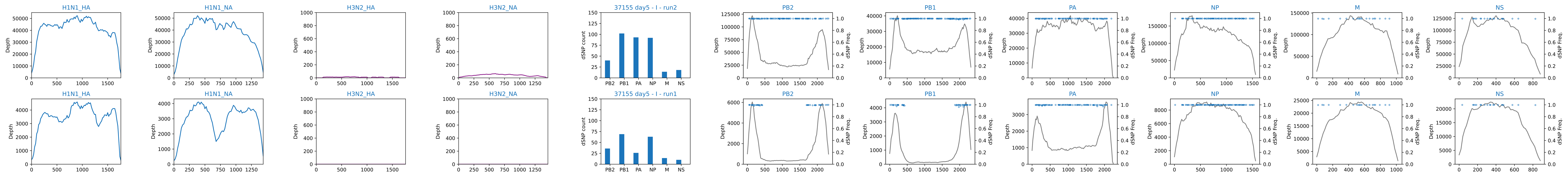

H1N1_HA
H1N1_NA
H3N2_HA
H3N2_NA
37155 day5 - I - run2
PB2
PB1
PA
NP
M
NS
H1N1_HA
H1N1_NA
H3N2_HA
H3N2_NA
37155 day5 - I - run1
PB2
PB1
PA
NP
M
NS
Depth
dSNP count
dSNP Freq.

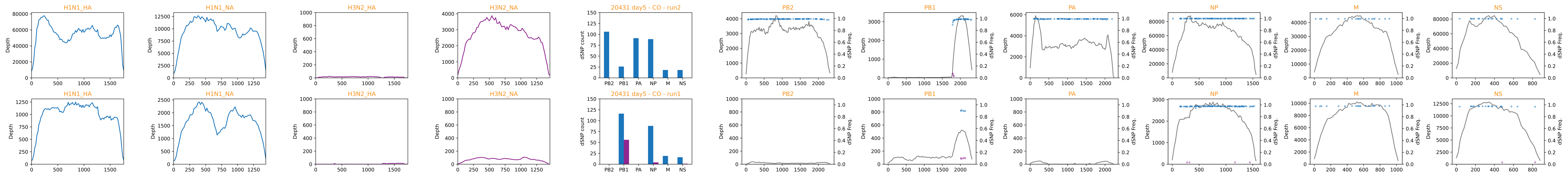

H1N1_HA
H1N1_NA
H3N2_HA
H3N2_NA
20431 day5 - CO - run2
PB2
PB1
PA
NP
M
NS
H1N1_HA
H1N1_NA
H3N2_HA
H3N2_NA
20431 day5 - CO - run1
PB2
PB1
PA
NP
M
NS
Depth
dSNP count
dSNP Freq.

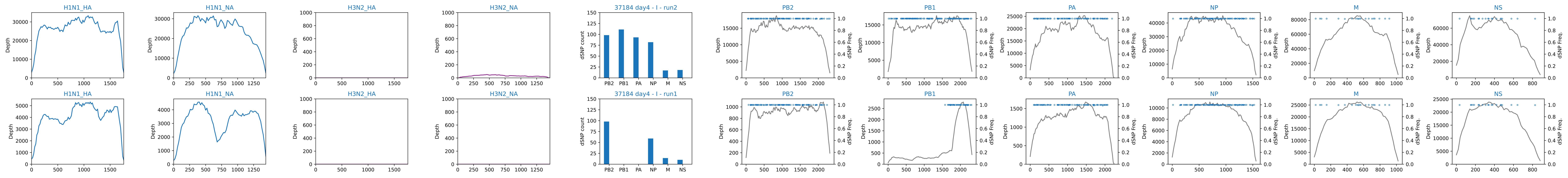

H1N1_HA
H1N1_NA
H3N2_HA
H3N2_NA
37184 day4 - I - run2
PB2
PB1
PA
NP
M
NS
H1N1_HA
H1N1_NA
H3N2_HA
H3N2_NA
37184 day4 - I - run1
PB2
PB1
PA
NP
M
NS
Depth
dSNP count
dSNP Freq.

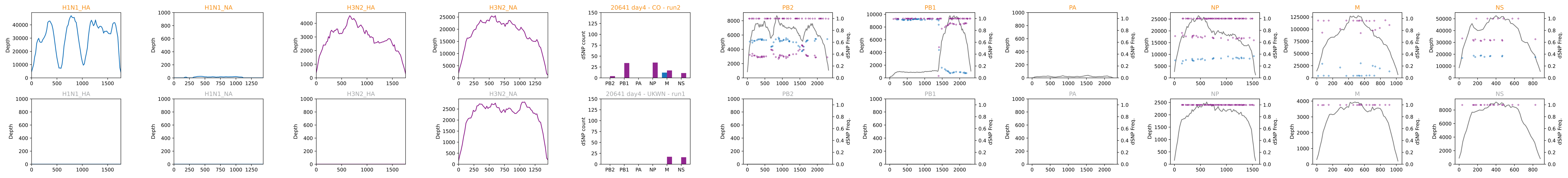
H1N1_HA
H1N1_NA
H3N2_HA
H3N2_NA
20641 day4 - CO - run2
PB2
PB1
PA
NP
M
NS
H1N1_HA
H1N1_NA
H3N2_HA
H3N2_NA
20641 day4 - UKWN - run1
PB2
PB1
PA
NP
M
NS
Depth
dSNP count
dSNP Freq.

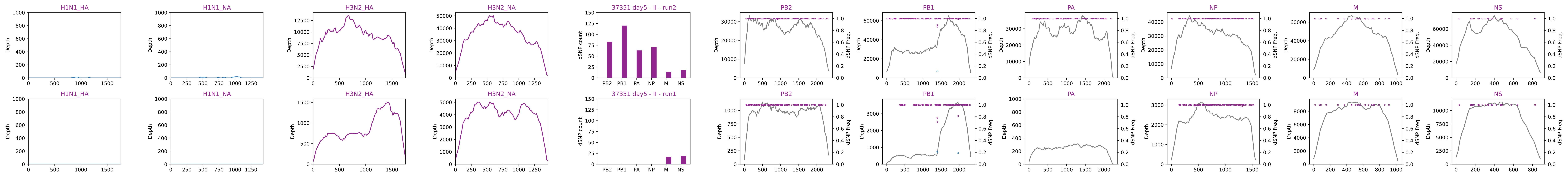
H1N1_HA
H1N1_NA
H3N2_HA
H3N2_NA
37351 day5 - II - run2
PB2
PB1
PA
NP
M
NS
37351 day5 - II - run1
Depth
dSNP count
dSNP Freq.

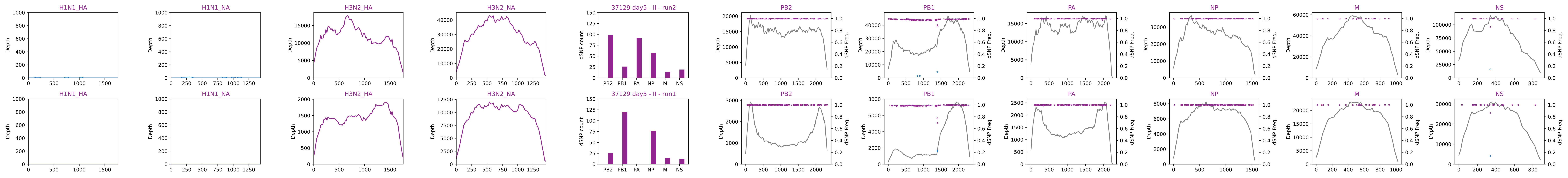
H1N1_HA
H1N1_NA
H3N2_HA
H3N2_NA
37129 day5 - II - run2
PB2
PB1
PA
NP
M
NS
H1N1_HA
H1N1_NA
H3N2_HA
H3N2_NA
37129 day5 - II - run1
PB2
PB1
PA
NP
M
NS
Depth
dSNP count
dSNP Freq.

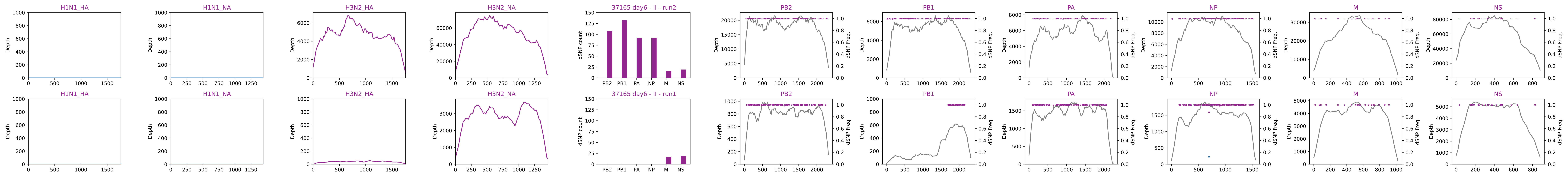
H1N1_HA
H1N1_NA
H3N2_HA
H3N2_NA
37165 day6 - II - run2
PB2
PB1
PA
NP
M
NS
H1N1_HA
H1N1_NA
H3N2_HA
H3N2_NA
37165 day6 - II - run1
PB2
PB1
PA
NP
M
NS
Depth
dSNP count
dSNP Freq.

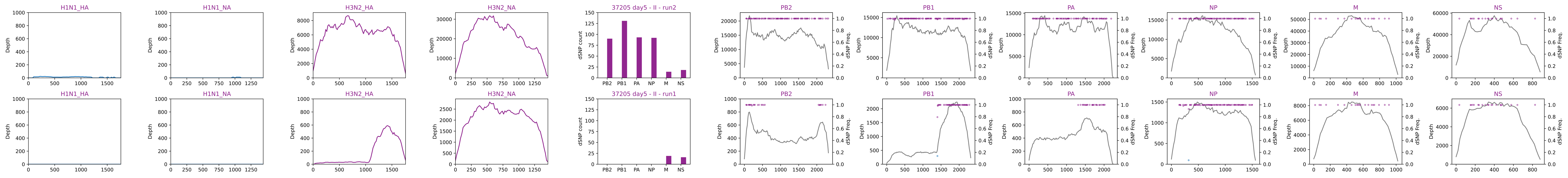
H1N1_HA
H1N1_NA
H3N2_HA
H3N2_NA
37205 day5 - II - run2
PB2
PB1
PA
NP
M
NS
H1N1_HA
H1N1_NA
H3N2_HA
H3N2_NA
37205 day5 - II - run1
PB2
PB1
PA
NP
M
NS
Depth
dSNP count
dSNP Freq.

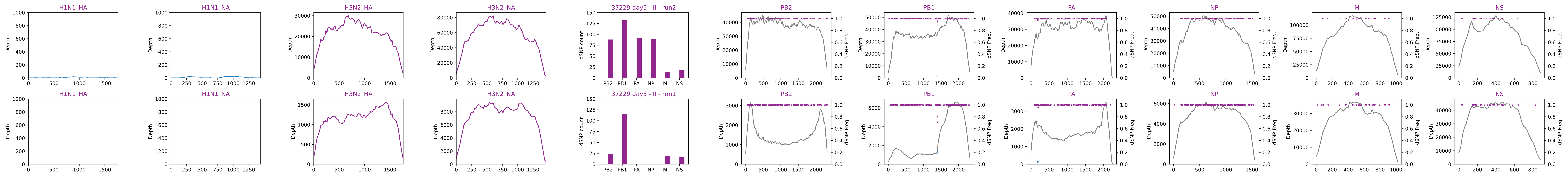
H1N1_HA
H1N1_NA
H3N2_HA
H3N2_NA
37229 day5 - II - run2
PB2
PB1
PA
NP
M
NS
H1N1_HA
H1N1_NA
H3N2_HA
H3N2_NA
37229 day5 - II - run1
PB2
PB1
PA
NP
M
NS
Depth
dSNP count
dSNP Freq.

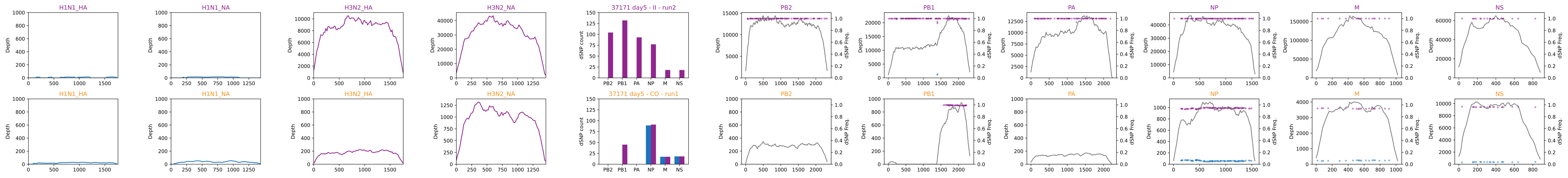
H1N1_HA
H1N1_NA
H3N2_HA
H3N2_NA
37171 day5 - II - run2
PB2
PB1
PA
NP
M
NS
H1N1_HA
H1N1_NA
H3N2_HA
H3N2_NA
37171 day5 - CO - run1
PB2
PB1
PA
NP
M
NS
Depth
dSNP count
dSNP Freq.

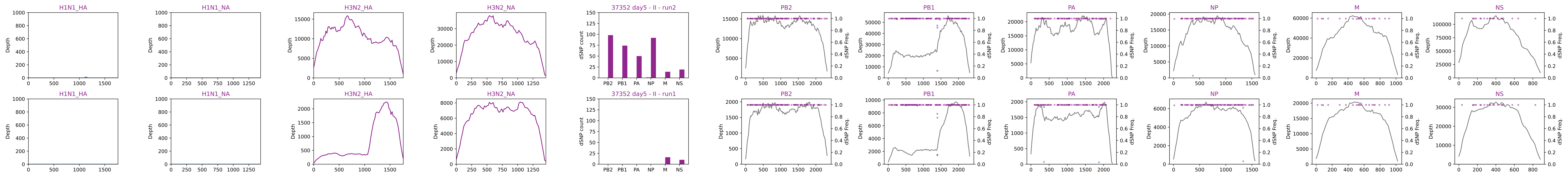

H1N1_HA
H1N1_NA
H3N2_HA
H3N2_NA
37352 day5 - II - run2
PB2
PB1
PA
NP
M
NS
37352 day5 - II - run1
Depth
dSNP count
dSNP Freq.

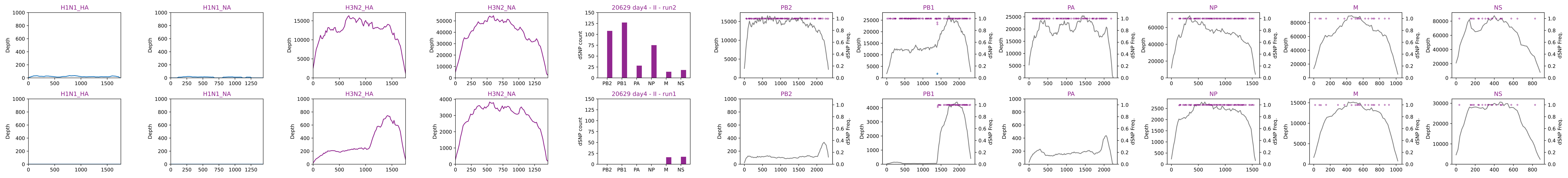
H1N1_HA
H1N1_NA
H3N2_HA
H3N2_NA
20629 day4 - II - run2
PB2
PB1
PA
NP
M
NS
H1N1_HA
H1N1_NA
H3N2_HA
H3N2_NA
20629 day4 - II - run1
PB2
PB1
PA
NP
M
NS
Depth
dSNP count
dSNP Freq.

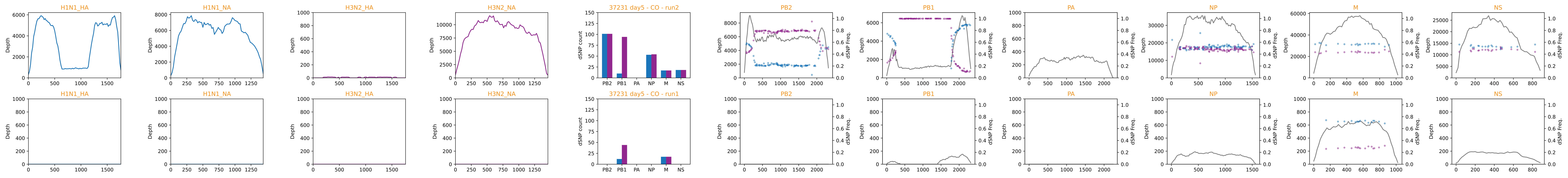
H1N1_HA
H1N1_NA
H3N2_HA
H3N2_NA
37231 day5 - CO - run2
PB2
PB1
PA
NP
M
NS
H1N1_HA
H1N1_NA
H3N2_HA
H3N2_NA
37231 day5 - CO - run1
PB2
PB1
PA
NP
M
NS
Depth
dSNP count
dSNP Freq.
PB2 PB1 PA NP M NS

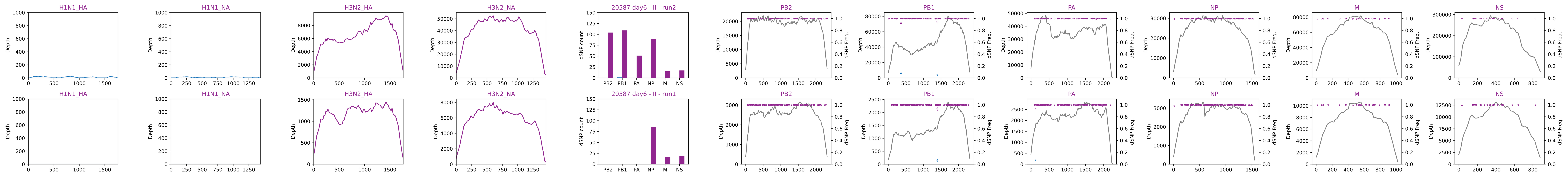
H1N1_HA
H1N1_NA
H3N2_HA
H3N2_NA
20587 day6 - II - run2
PB2
PB1
PA
NP
M
NS
H1N1_HA
H1N1_NA
H3N2_HA
H3N2_NA
20587 day6 - II - run1
PB2
PB1
PA
NP
M
NS
Depth
dSNP count
dSNP Freq.

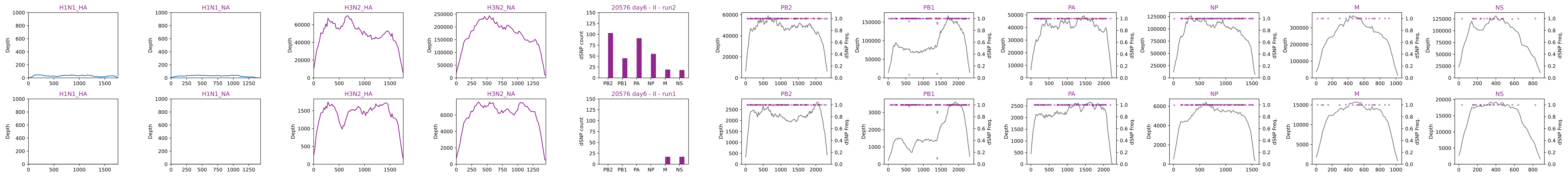
H1N1_HA
H1N1_NA
H3N2_HA
H3N2_NA
20576 day6 - II - run2
PB2
PB1
PA
NP
M
NS
H1N1_HA
H1N1_NA
H3N2_HA
H3N2_NA
20576 day6 - II - run1
PB2
PB1
PA
NP
M
NS
Depth
dSNP count
dSNP Freq.

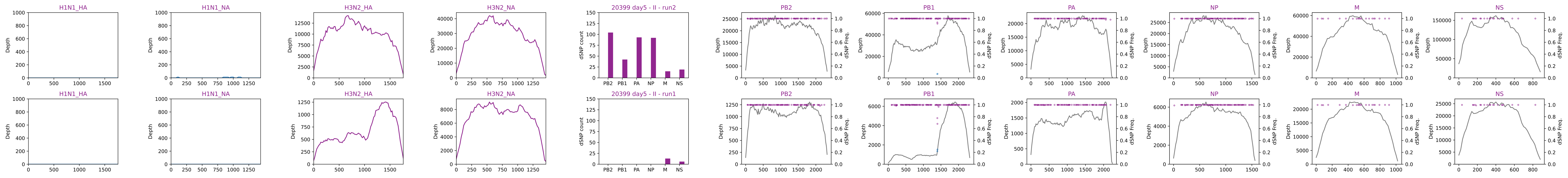
H1N1_HA
H1N1_NA
H3N2_HA
H3N2_NA
20399 day5 - II - run2
PB2
PB1
PA
NP
M
NS
20399 day5 - II - run1
Depth
dSNP count
dSNP Freq.

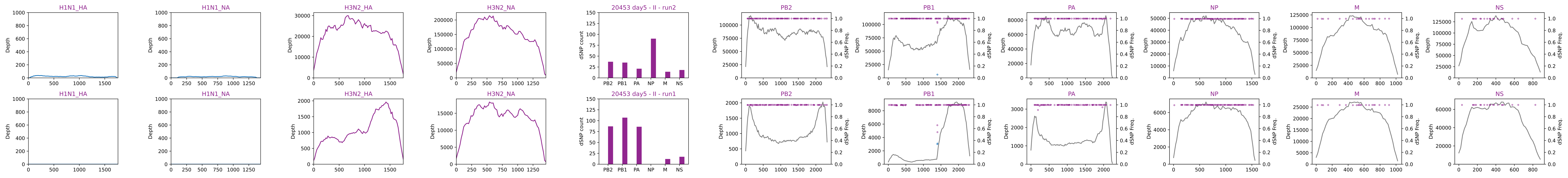

H1N1_HA
H1N1_NA
H3N2_HA
H3N2_NA
20453 day5 - II - run2
PB2
PB1
PA
NP
M
NS
20453 day5 - II - run1

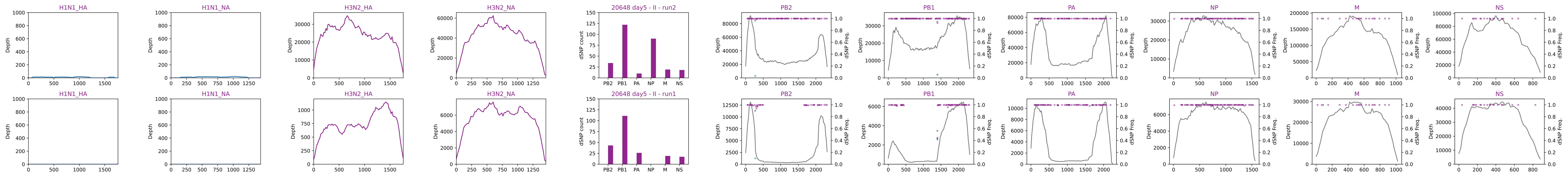
H1N1_HA
H1N1_NA
H3N2_HA
H3N2_NA
20648 day5 - II - run2
PB2
PB1
PA
NP
M
NS
20648 day5 - II - run1
Depth
dSNP count
dSNP Freq.

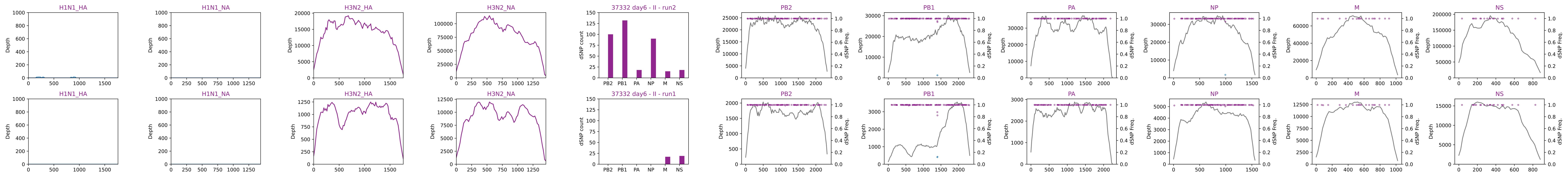
H1N1_HA
H1N1_NA
H3N2_HA
H3N2_NA
37332 day6 - II - run2
PB2
PB1
PA
NP
M
NS
H1N1_HA
H1N1_NA
H3N2_HA
H3N2_NA
37332 day6 - II - run1
PB2
PB1
PA
NP
M
NS
Depth
dSNP count
dSNP Freq.

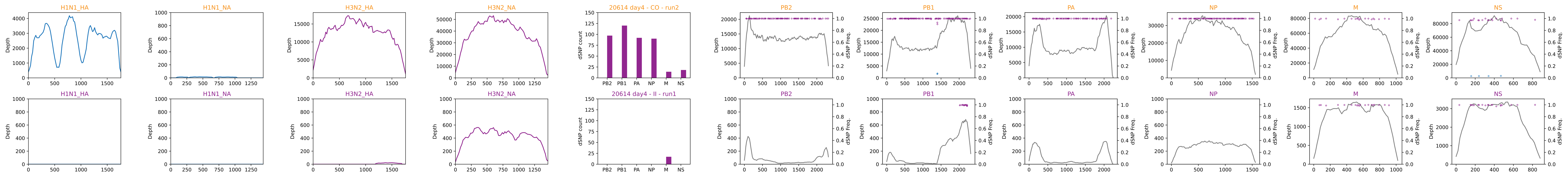
H1N1_HA
H1N1_NA
H3N2_HA
H3N2_NA
20614 day4 - CO - run2
PB2
PB1
PA
NP
M
NS
H1N1_HA
H1N1_NA
H3N2_HA
H3N2_NA
20614 day4 - II - run1
PB2
PB1
PA
NP
M
NS
Depth
dSNP count
dSNP Freq.

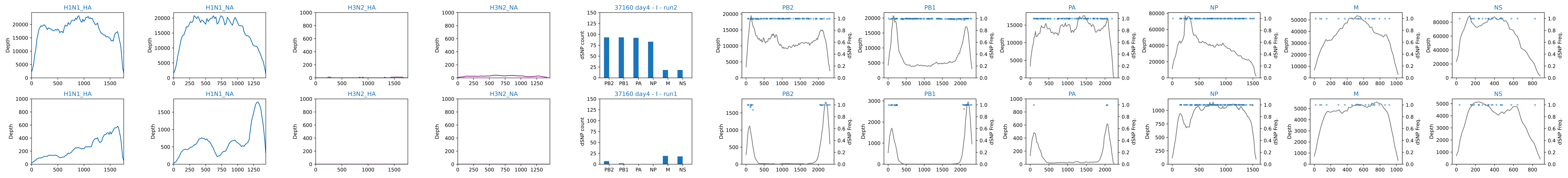
H1N1_HA
H1N1_NA
H3N2_HA
H3N2_NA
37160 day4 - I - run2
PB2
PB1
PA
NP
M
NS
H1N1_HA
H1N1_NA
H3N2_HA
H3N2_NA
37160 day4 - I - run1
PB2
PB1
PA
NP
M
NS
Depth
dSNP count
dSNP Freq.

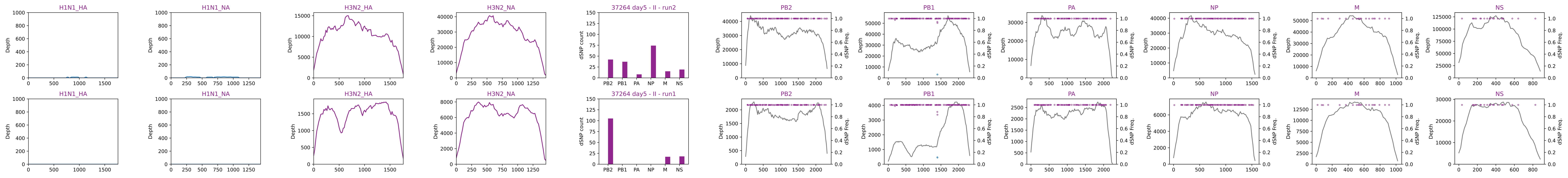
H1N1_HA
H1N1_NA
H3N2_HA
H3N2_NA
37264 day5 - II - run2
PB2
PB1
PA
NP
M
NS
H1N1_HA
H1N1_NA
H3N2_HA
H3N2_NA
37264 day5 - II - run1
PB2
PB1
PA
NP
M
NS
Depth
dSNP count
dSNP Freq.

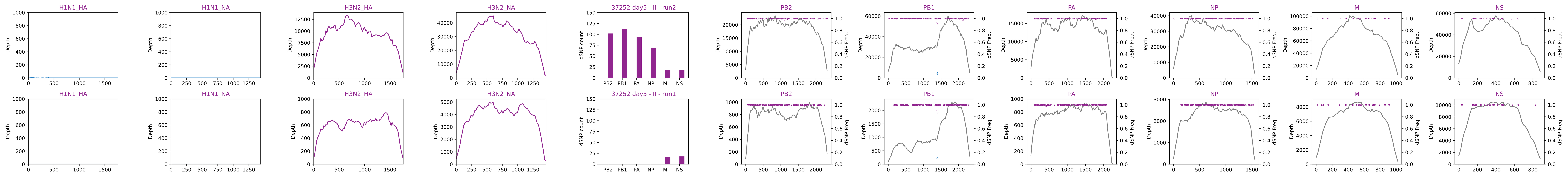
H1N1_HA
H1N1_NA
H3N2_HA
H3N2_NA
37252 day5 - II - run2
PB2
PB1
PA
NP
M
NS
H1N1_HA
H1N1_NA
H3N2_HA
H3N2_NA
37252 day5 - II - run1
PB2
PB1
PA
NP
M
NS
Depth
dSNP count
dSNP Freq.

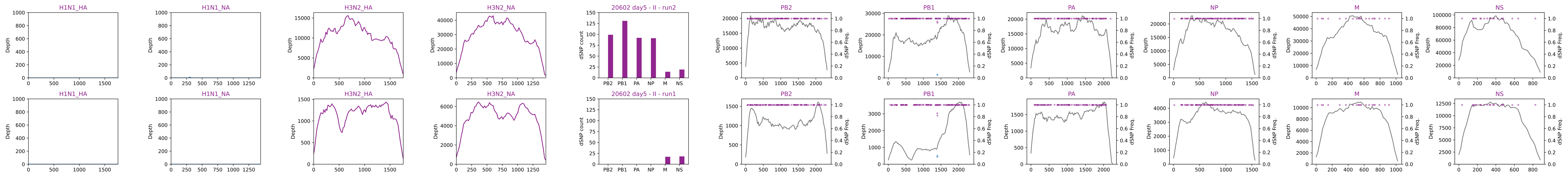

H1N1_HA
H1N1_NA
H3N2_HA
H3N2_NA
20602 day5 - II - run2
PB2
PB1
PA
NP
M
NS
20602 day5 - II - run1
Depth
dSNP count
dSNP Freq.

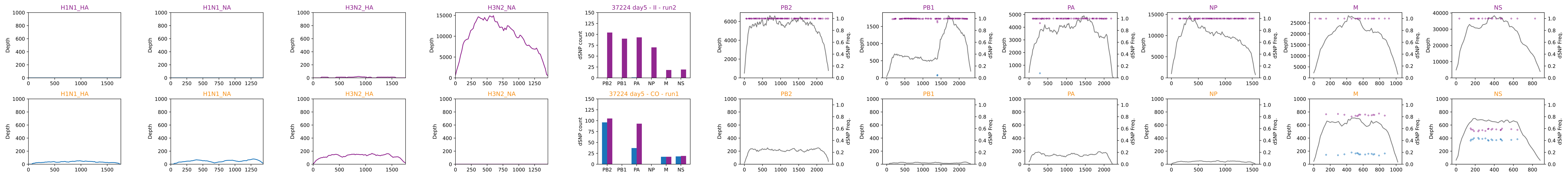
H1N1_HA
H1N1_NA
H3N2_HA
H3N2_NA
37224 day5 - II - run2
PB2
PB1
PA
NP
M
NS
37224 day5 - CO - run1
Depth
dSNP count
dSNP Freq.

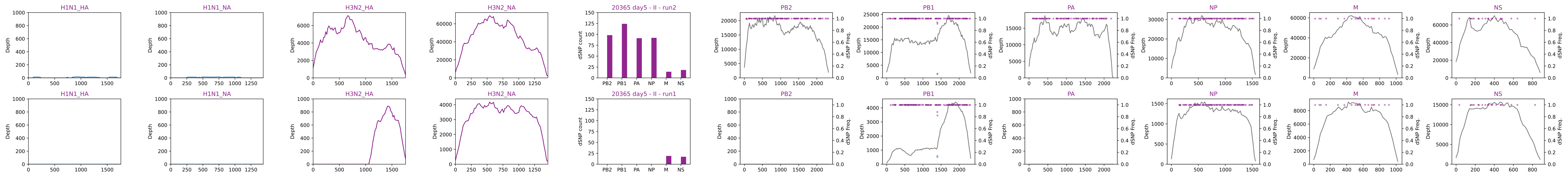
H1N1_HA
H1N1_NA
H3N2_HA
H3N2_NA
20365 day5 - II - run2
PB2
PB1
PA
NP
M
NS
H1N1_HA
H1N1_NA
H3N2_HA
H3N2_NA
20365 day5 - II - run1
PB2
PB1
PA
NP
M
NS
Depth
dSNP count
dSNP Freq.

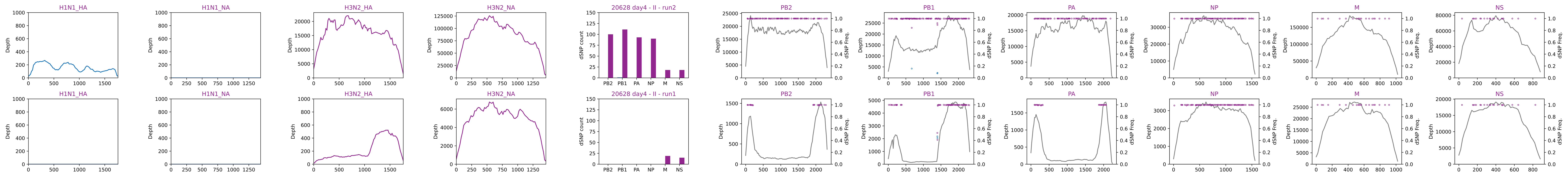

H1N1_HA
H1N1_NA
H3N2_HA
H3N2_NA
20628 day4 - II - run2
PB2
PB1
PA
NP
M
NS
H1N1_HA
H1N1_NA
H3N2_HA
H3N2_NA
20628 day4 - II - run1
PB2
PB1
PA
NP
M
NS
Depth
dSNP count
dSNP Freq.

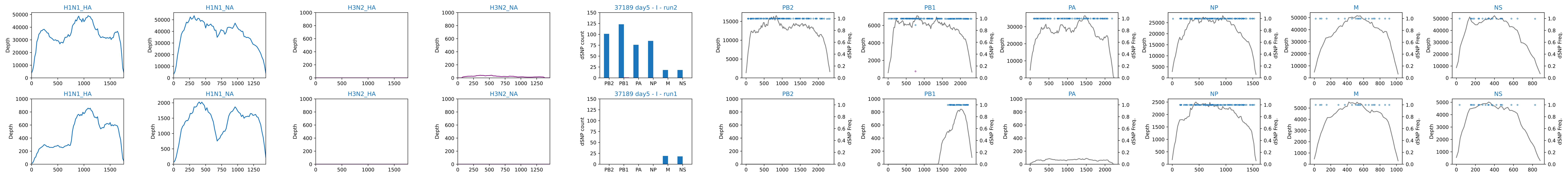

H1N1_HA
H1N1_NA
H3N2_HA
H3N2_NA
37189 day5 - I - run2
PB2
PB1
PA
NP
M
NS
H1N1_HA
H1N1_NA
H3N2_HA
H3N2_NA
37189 day5 - I - run1
PB2
PB1
PA
NP
M
NS
Depth
dSNP count
dSNP Freq.

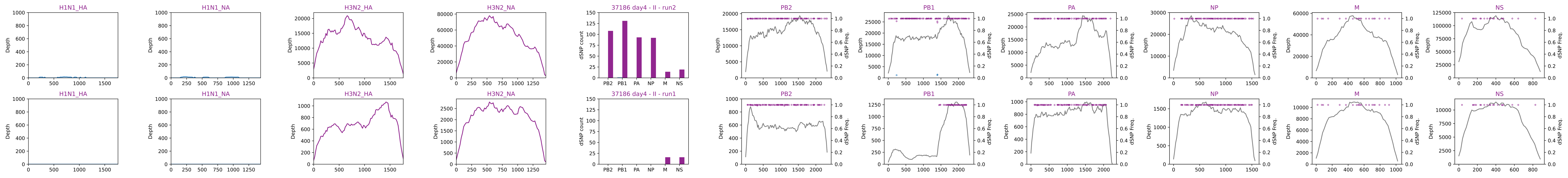
H1N1_HA
H1N1_NA
H3N2_HA
H3N2_NA
37186 day4 - II - run2
PB2
PB1
PA
NP
M
NS
H1N1_HA
H1N1_NA
H3N2_HA
H3N2_NA
37186 day4 - II - run1
PB2
PB1
PA
NP
M
NS
Depth
dSNP count
dSNP Freq.

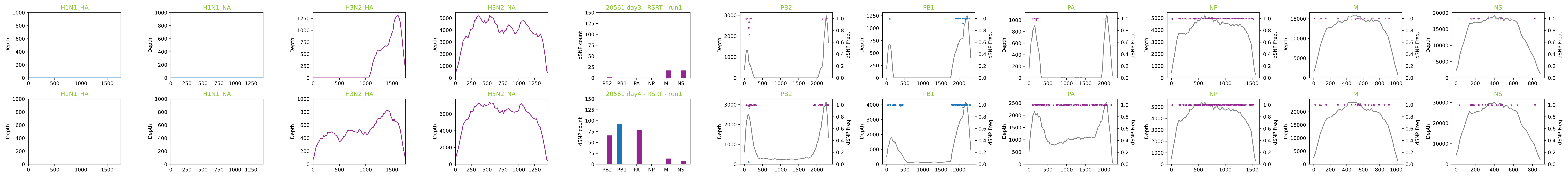
H1N1_HA
H1N1_NA
H3N2_HA
H3N2_NA
20561 day3 - RSRT - run1
PB2
PB1
PA
NP
M
NS
H1N1_HA
H1N1_NA
H3N2_HA
H3N2_NA
20561 day4 - RSRT - run1
PB2
PB1
PA
NP
M
NS
Depth
dSNP count
dSNP Freq.
PB2 PB1 PA NP M NS

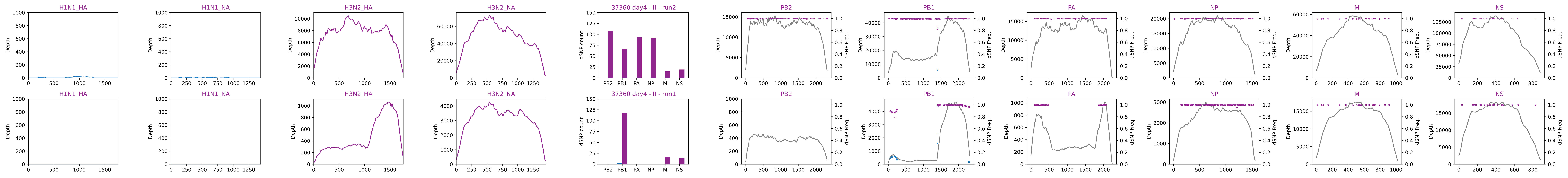

H1N1_HA
H1N1_NA
H3N2_HA
H3N2_NA
37360 day4 - II - run2
PB2
PB1
PA
NP
M
NS
37360 day4 - II - run1
Depth
dSNP count
dSNP Freq.

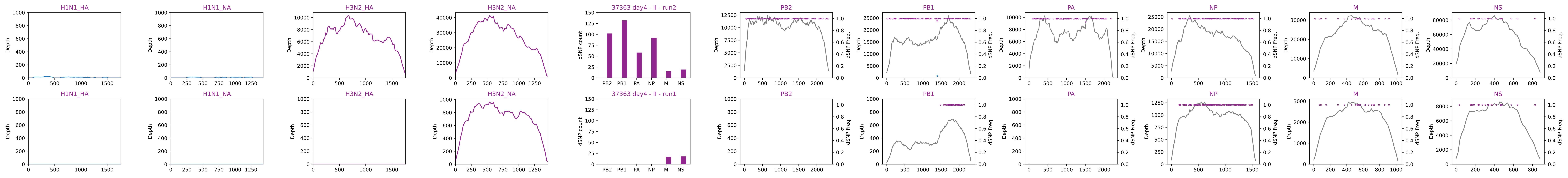
H1N1_HA
H1N1_NA
H3N2_HA
H3N2_NA
37363 day4 - II - run2
PB2
PB1
PA
NP
M
NS
H1N1_HA
H1N1_NA
H3N2_HA
H3N2_NA
37363 day4 - II - run1
PB2
PB1
PA
NP
M
NS
Depth
dSNP count
dSNP Freq.

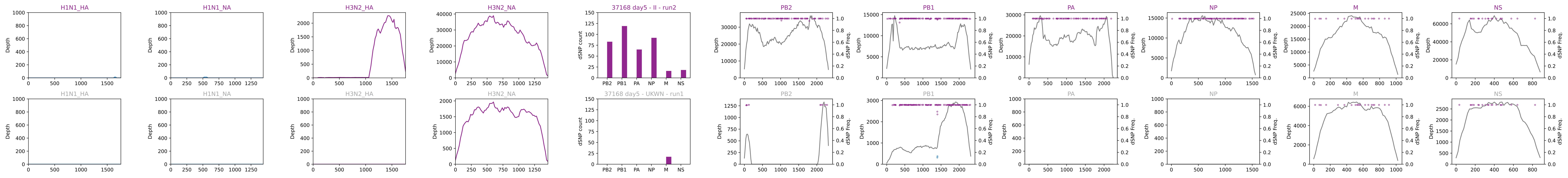
H1N1_HA
H1N1_NA
H3N2_HA
H3N2_NA
37168 day5 - II - run2
PB2
PB1
PA
NP
M
NS
H1N1_HA
H1N1_NA
H3N2_HA
H3N2_NA
37168 day5 - UKWN - run1
PB2
PB1
PA
NP
M
NS
Depth
dSNP count
dSNP Freq.

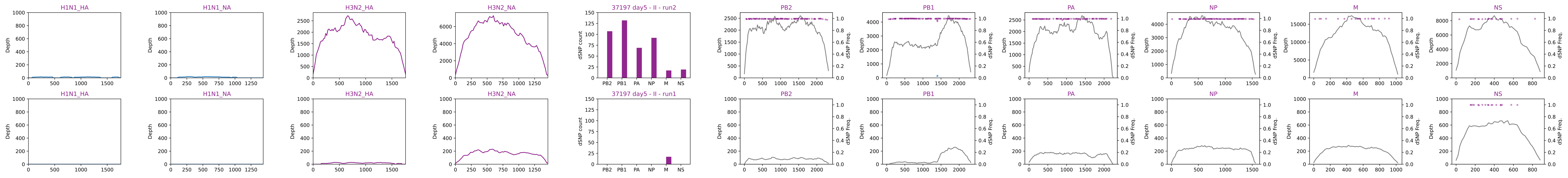

H1N1_HA
H1N1_NA
H3N2_HA
H3N2_NA
37197 day5 - II - run2
PB2
PB1
PA
NP
M
NS
H1N1_HA
H1N1_NA
H3N2_HA
H3N2_NA
37197 day5 - II - run1
PB2
PB1
PA
NP
M
NS

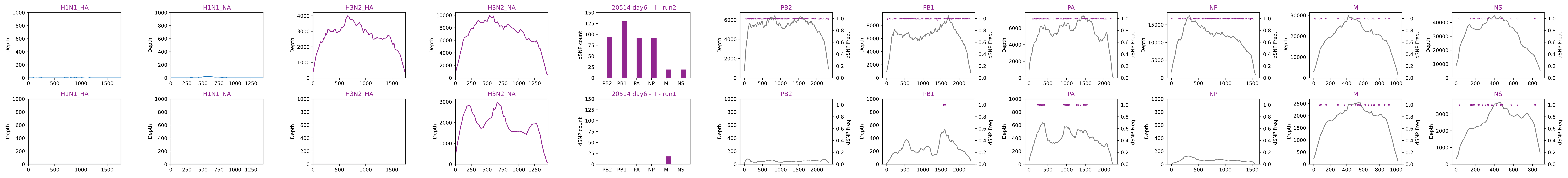
H1N1_HA
H1N1_NA
H3N2_HA
H3N2_NA
20514 day6 - II - run2
PB2
PB1
PA
NP
M
NS
H1N1_HA
H1N1_NA
H3N2_HA
H3N2_NA
20514 day6 - II - run1
PB2
PB1
PA
NP
M
NS
Depth
dSNP count
dSNP Freq.

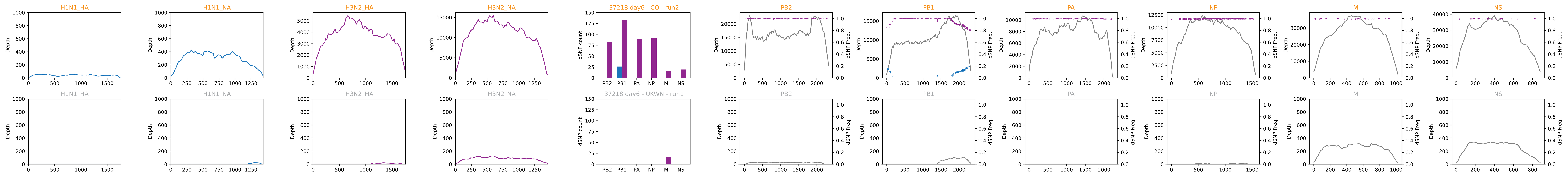
H1N1_HA
H1N1_NA
H3N2_HA
H3N2_NA
37218 day6 - CO - run2
PB2
PB1
PA
NP
M
NS
Depth
dSNP count
dSNP Freq.
37218 day6 - UKWN - run1

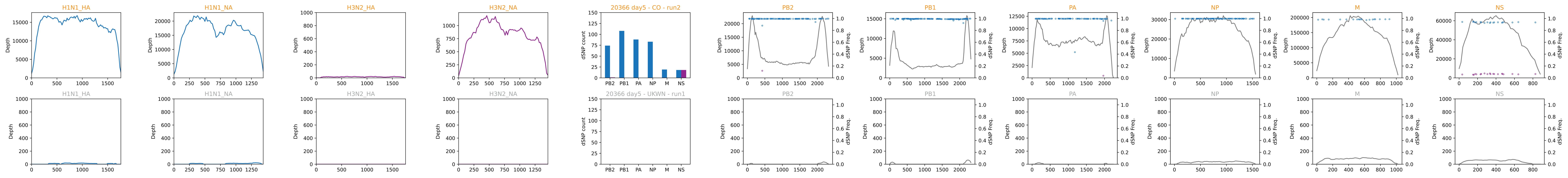
H1N1_HA
H1N1_NA
H3N2_HA
H3N2_NA
20366 day5 - CO - run2
PB2
PB1
PA
NP
M
NS
H1N1_HA
H1N1_NA
H3N2_HA
H3N2_NA
20366 day5 - UKWN - run1
PB2
PB1
PA
NP
M
NS
Depth
dSNP count
dSNP Freq.

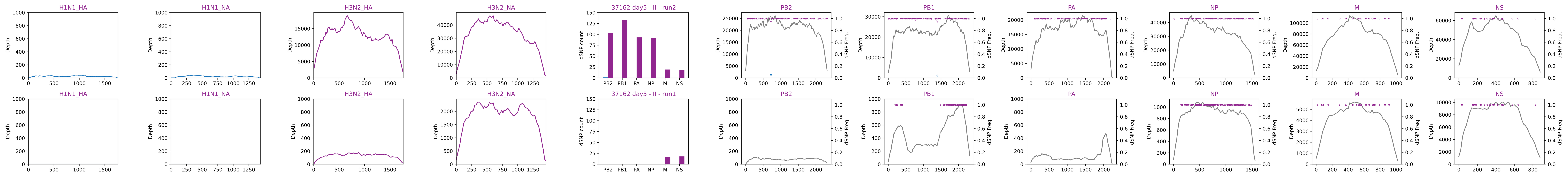
H1N1_HA
H1N1_NA
H3N2_HA
H3N2_NA
37162 day5 - II - run2
PB2
PB1
PA
NP
M
NS
37162 day5 - II - run1
Depth
dSNP count
dSNP Freq.

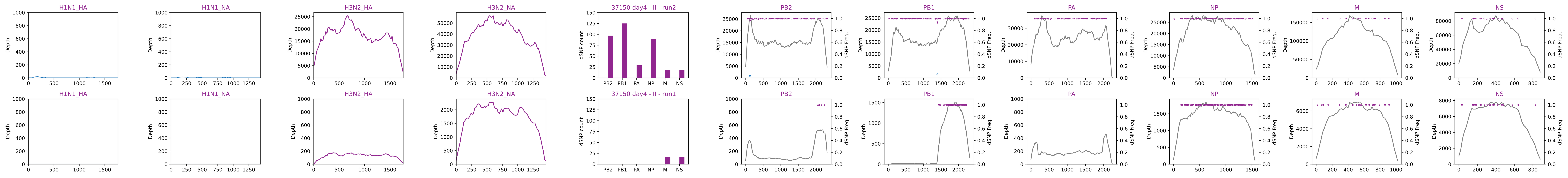

H1N1_HA
H1N1_NA
H3N2_HA
H3N2_NA
37150 day4 - II - run2
PB2
PB1
PA
NP
M
NS
H1N1_HA
H1N1_NA
H3N2_HA
H3N2_NA
37150 day4 - II - run1
PB2
PB1
PA
NP
M
NS
Depth
dSNP count
dSNP Freq.

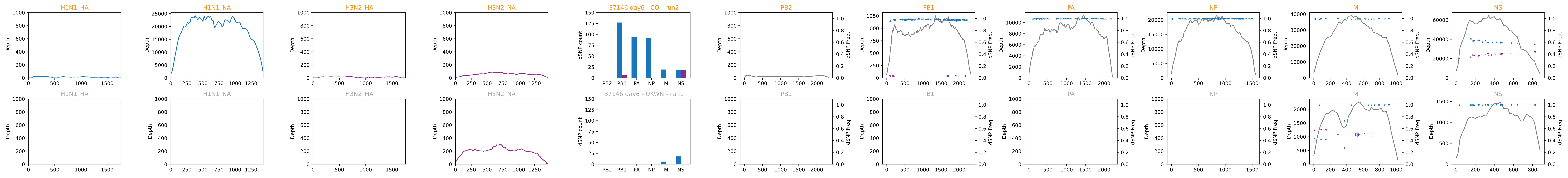
H1N1_HA
H1N1_NA
H3N2_HA
H3N2_NA
37146 day6 - CO - run2
PB2
PB1
PA
NP
M
NS
37146 day6 - UKWN - run1
Depth
dSNP count
dSNP Freq.

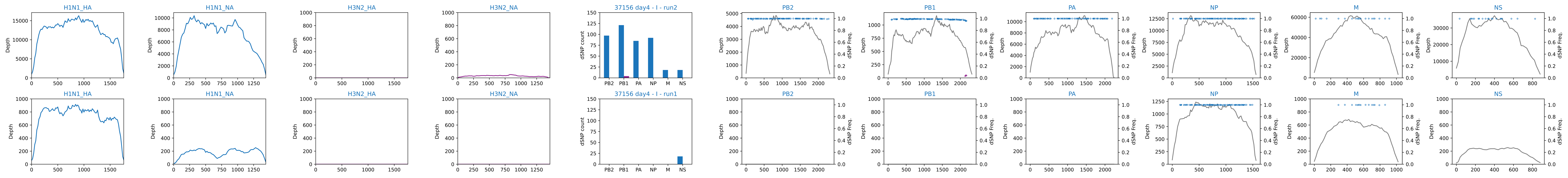
H1N1_HA
H1N1_NA
H3N2_HA
H3N2_NA
37156 day4 - I - run2
PB2
PB1
PA
NP
M
NS
H1N1_HA
H1N1_NA
H3N2_HA
H3N2_NA
37156 day4 - I - run1
PB2
PB1
PA
NP
M
NS
Depth
dSNP count
dSNP Freq.

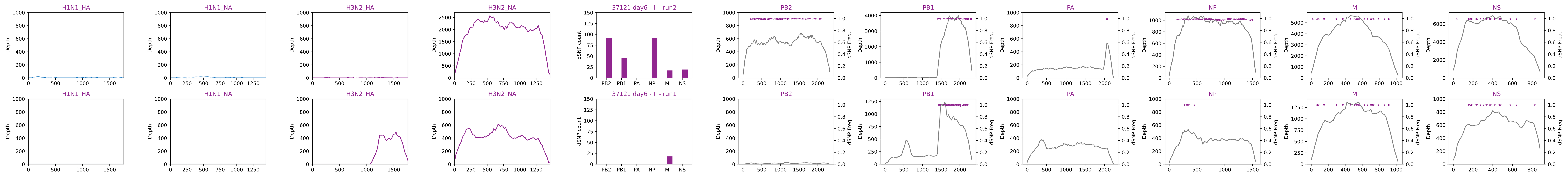

H1N1_HA
H1N1_NA
H3N2_HA
H3N2_NA
37121 day6 - II - run2
PB2
PB1
PA
NP
M
NS
H1N1_HA
H1N1_NA
H3N2_HA
H3N2_NA
37121 day6 - II - run1
PB2
PB1
PA
NP
M
NS
Depth
dSNP count
dSNP Freq.
PB2 PB1 PA NP M NS

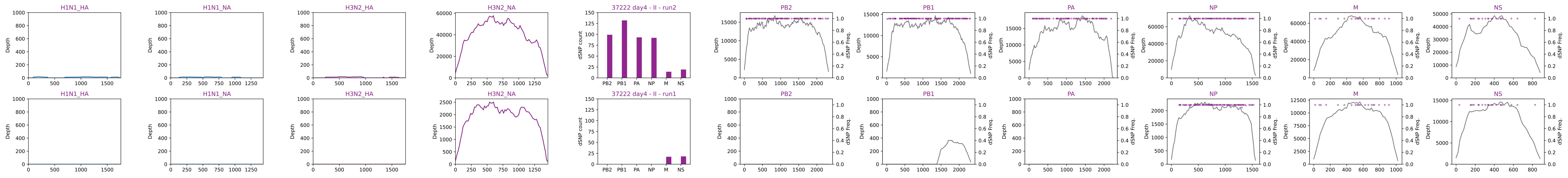
H1N1_HA
H1N1_NA
H3N2_HA
H3N2_NA
37222 day4 - II - run2
PB2
PB1
PA
NP
M
NS
H1N1_HA
H1N1_NA
H3N2_HA
H3N2_NA
37222 day4 - II - run1
PB2
PB1
PA
NP
M
NS
Depth
dSNP count
dSNP Freq.

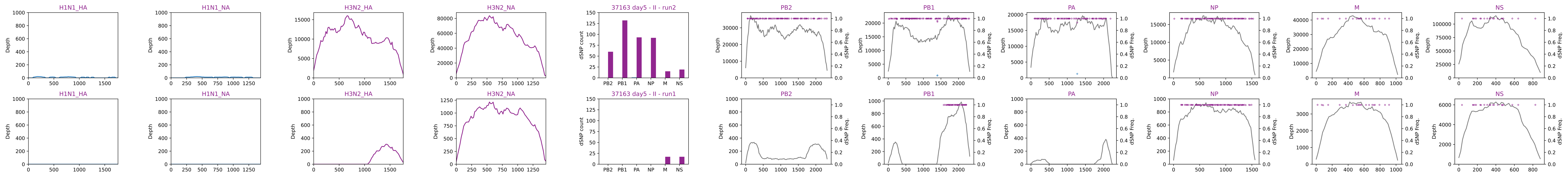
H1N1_HA
H1N1_NA
H3N2_HA
H3N2_NA
37163 day5 - II - run2
PB2
PB1
PA
NP
M
NS
H1N1_HA
H1N1_NA
H3N2_HA
H3N2_NA
37163 day5 - II - run1
PB2
PB1
PA
NP
M
NS
Depth
dSNP count
dSNP Freq.

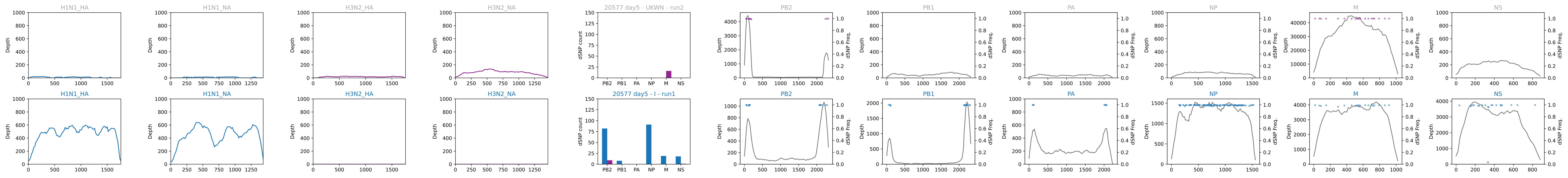
H1N1_HA
H1N1_NA
H3N2_HA
H3N2_NA
20577 day5 - UKWN - run2
PB2
PB1
PA
NP
M
NS
H1N1_HA
H1N1_NA
H3N2_HA
H3N2_NA
20577 day5 - I - run1
PB2
PB1
PA
NP
M
NS
Depth
dSNP count
dSNP Freq.

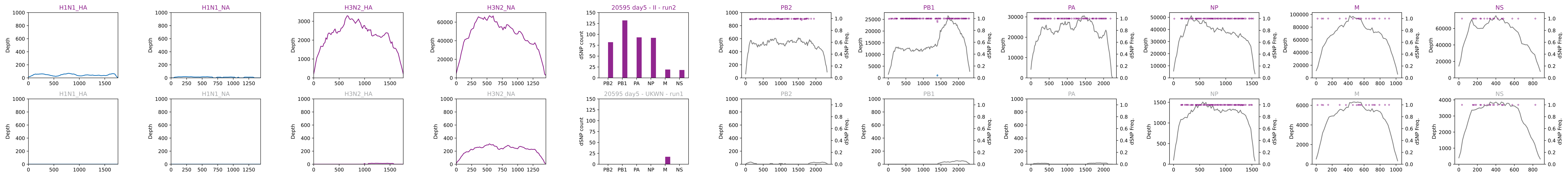
H1N1_HA
H1N1_NA
H3N2_HA
H3N2_NA
20595 day5 - II - run2
PB2
PB1
PA
NP
M
NS
H1N1_HA
H1N1_NA
H3N2_HA
H3N2_NA
20595 day5 - UKWN - run1
PB2
PB1
PA
NP
M
NS
Depth
dSNP count
dSNP Freq.

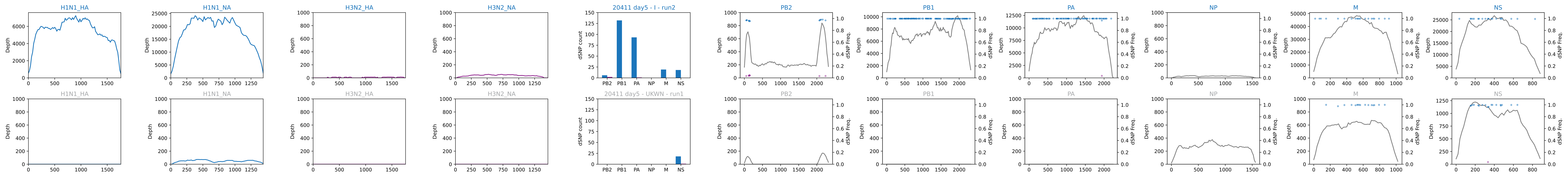
H1N1_HA
H1N1_NA
H3N2_HA
H3N2_NA
20411 day5 - I - run2
PB2
PB1
PA
NP
M
NS
H1N1_HA
H1N1_NA
H3N2_HA
H3N2_NA
20411 day5 - UKWN - run1
PB2
PB1
PA
NP
M
NS

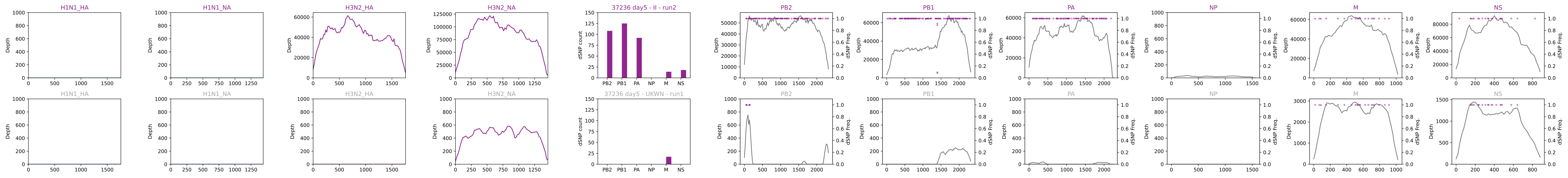
H1N1_HA
H1N1_NA
H3N2_HA
H3N2_NA
37236 day5 - II - run2
PB2
PB1
PA
NP
M
NS
37236 day5 - UKWN - run1
Depth
dSNP count
dSNP Freq.

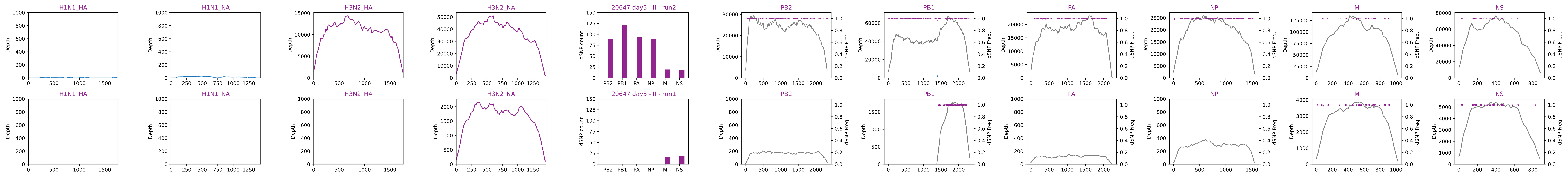
H1N1_HA
H1N1_NA
H3N2_HA
H3N2_NA
20647 day5 - II - run2
PB2
PB1
PA
NP
M
NS
H1N1_HA
H1N1_NA
H3N2_HA
H3N2_NA
20647 day5 - II - run1
PB2
PB1
PA
NP
M
NS
Depth
dSNP count
dSNP Freq.

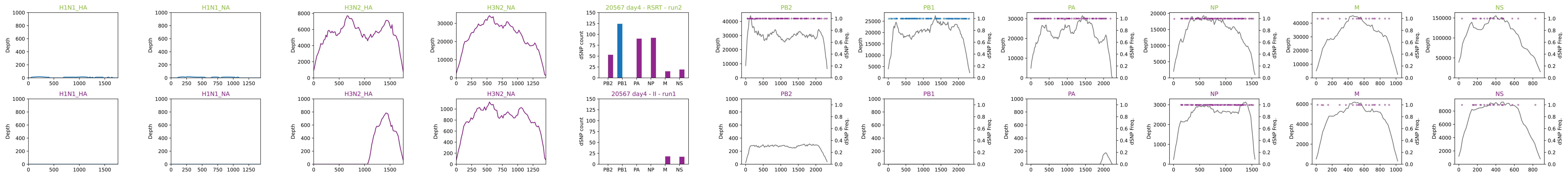
H1N1_HA
H1N1_NA
H3N2_HA
H3N2_NA
20567 day4 - RSRT - run2
PB2
PB1
PA
NP
M
NS
H1N1_HA
H1N1_NA
H3N2_HA
H3N2_NA
20567 day4 - II - run1
PB2
PB1
PA
NP
M
NS
Depth
dSNP count
dSNP Freq.
PB2 PB1 PA NP M NS

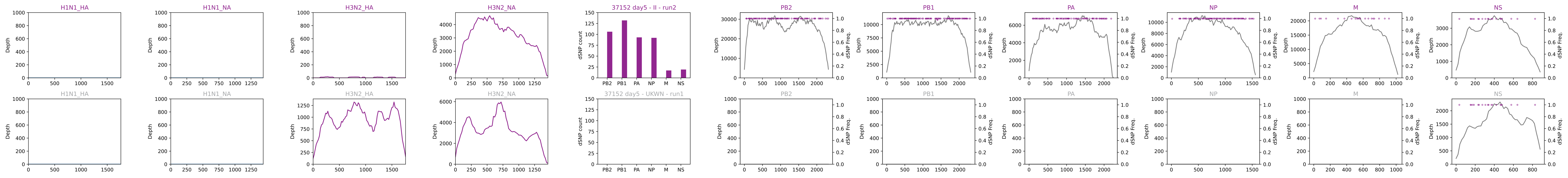
H1N1_HA
H1N1_NA
H3N2_HA
H3N2_NA
37152 day5 - II - run2
PB2
PB1
PA
NP
M
NS
H1N1_HA
H1N1_NA
H3N2_HA
H3N2_NA
37152 day5 - UKWN - run1
PB2
PB1
PA
NP
M
NS
Depth
dSNP count
dSNP Freq.

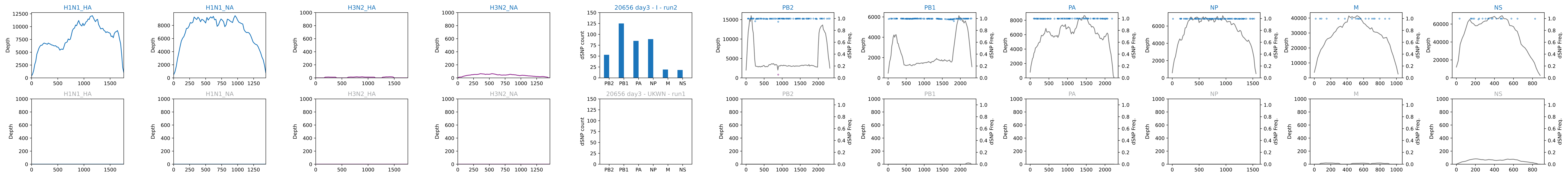
H1N1_HA
H1N1_NA
H3N2_HA
H3N2_NA
20656 day3 - I - run2
PB2
PB1
PA
NP
M
NS
20656 day3 - UKWN - run1
Depth
dSNP count
dSNP Freq.

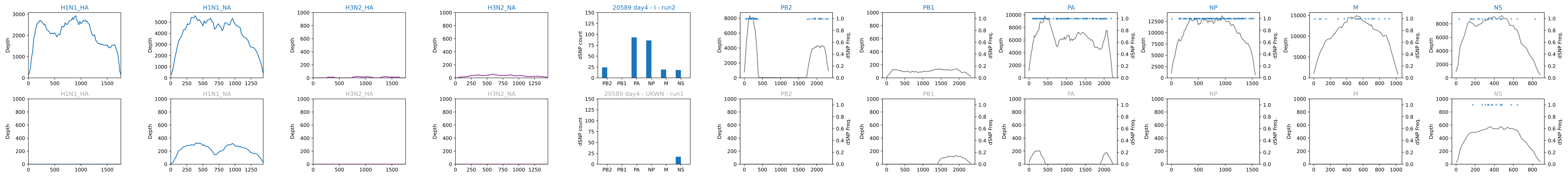

H1N1_HA
H1N1_NA
H3N2_HA
H3N2_NA
20589 day4 - I - run2
PB2
PB1
PA
NP
M
NS
20589 day4 - UKWN - run1
Depth
dSNP count
dSNP Freq.

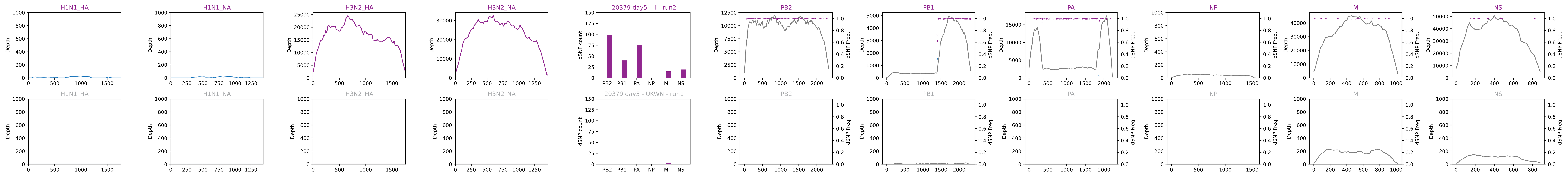
H1N1_HA
H1N1_NA
H3N2_HA
H3N2_NA
20379 day5 - II - run2
PB2
PB1
PA
NP
M
NS
20379 day5 - UKWN - run1
Depth
dSNP count
dSNP Freq.

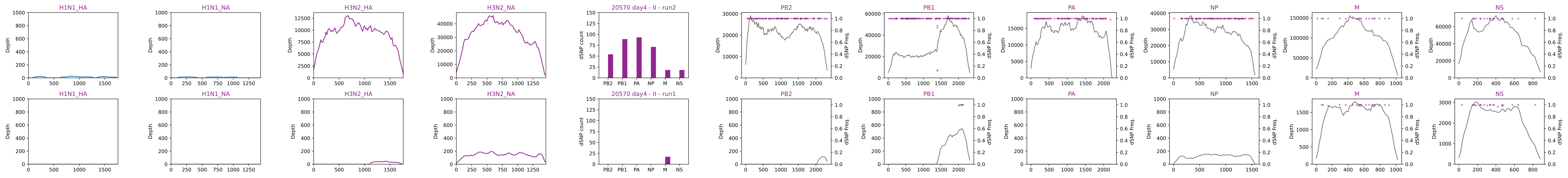
H1N1_HA
H1N1_NA
H3N2_HA
H3N2_NA
20570 day4 - II - run2
PB2
PB1
PA
NP
M
NS
H1N1_HA
H1N1_NA
H3N2_HA
H3N2_NA
20570 day4 - II - run1
PB2
PB1
PA
NP
M
NS
Depth
dSNP count
dSNP Freq.

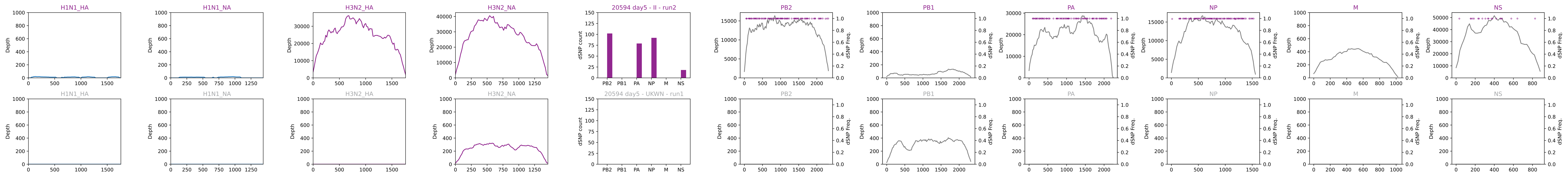
H1N1_HA
H1N1_NA
H3N2_HA
H3N2_NA
20594 day5 - II - run2
PB2
PB1
PA
NP
M
NS
20594 day5 - UKWN - run1
Depth
dSNP count
dSNP Freq.

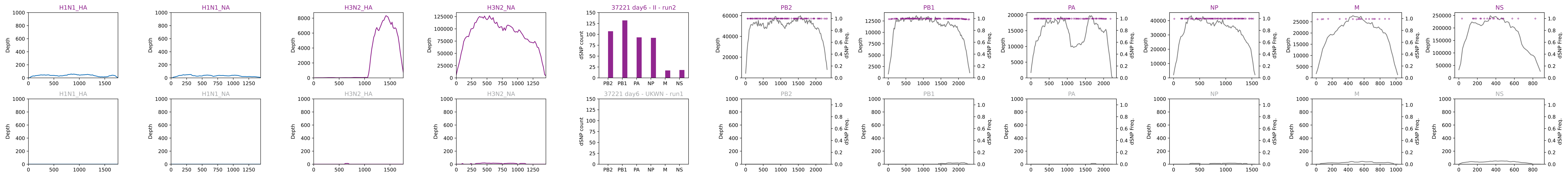
H1N1_HA
H1N1_NA
H3N2_HA
H3N2_NA
37221 day6 - II - run2
PB2
PB1
PA
NP
M
NS
H1N1_HA
H1N1_NA
H3N2_HA
H3N2_NA
37221 day6 - UKWN - run1
PB2
PB1
PA
NP
M
NS
Depth
dSNP count
dSNP Freq.

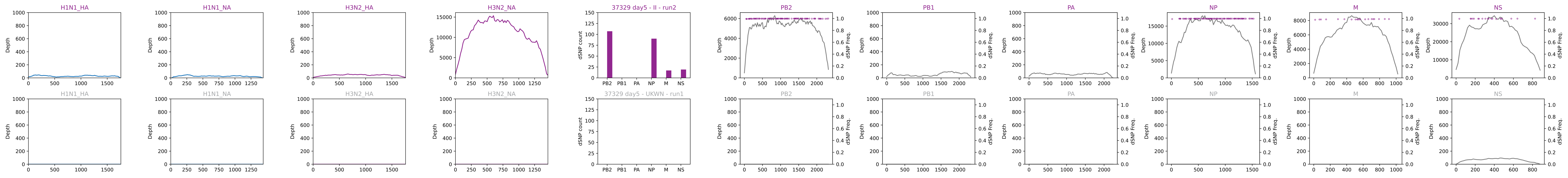

H1N1_HA
H1N1_NA
H3N2_HA
H3N2_NA
37329 day5 - II - run2
PB2
PB1
PA
NP
M
NS
37329 day5 - UKWN - run1
Depth
dSNP count
dSNP Freq.

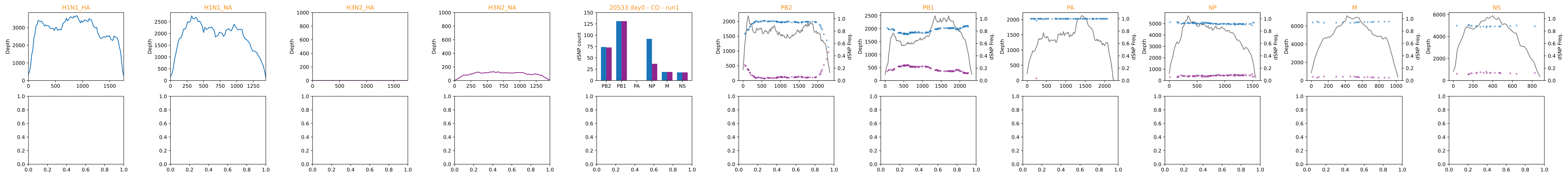
H1N1_HA
H1N1_NA
H3N2_HA
H3N2_NA
20533 day0 - CO - run1
PB2
PB1
PA
NP
M
NS
Depth
dSNP count
dSNP Freq.

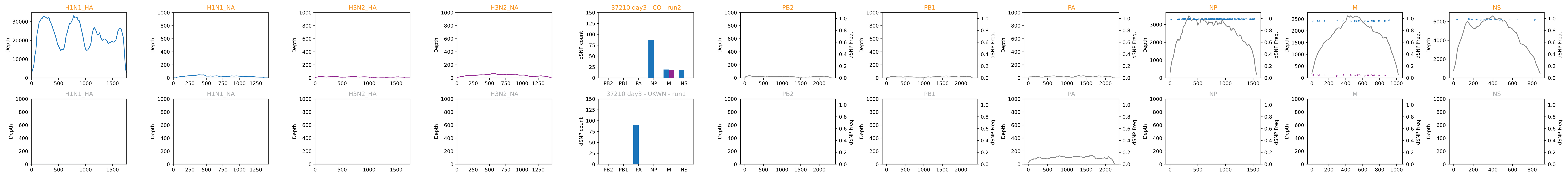
H1N1_HA
H1N1_NA
H3N2_HA
H3N2_NA
37210 day3 - CO - run2
PB2
PB1
PA
NP
M
NS
H1N1_HA
H1N1_NA
H3N2_HA
H3N2_NA
37210 day3 - UKWN - run1
PB2
PB1
PA
NP
M
NS
Depth
dSNP count
dSNP Freq.

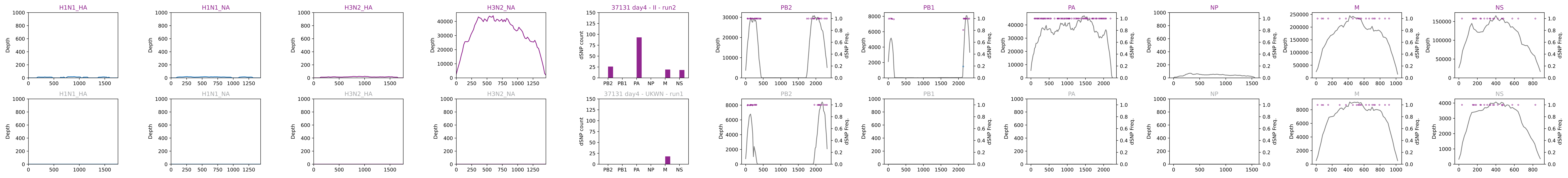
H1N1_HA
H1N1_NA
H3N2_HA
H3N2_NA
37131 day4 - II - run2
PB2
PB1
PA
NP
M
NS
H1N1_HA
H1N1_NA
H3N2_HA
H3N2_NA
37131 day4 - UKWN - run1
PB2
PB1
PA
NP
M
NS
Depth
dSNP count
dSNP Freq.

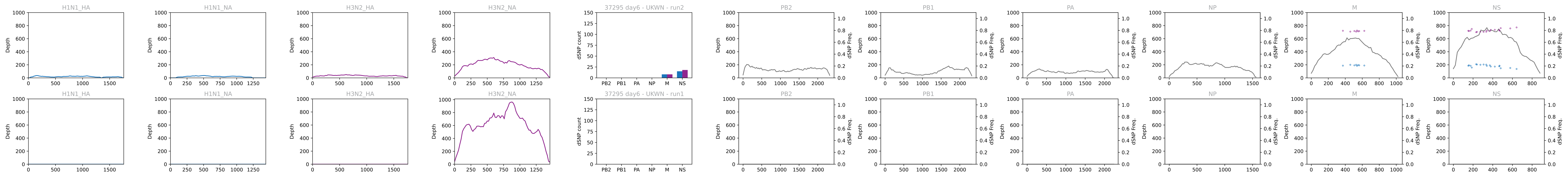
H1N1_HA
H1N1_NA
H3N2_HA
H3N2_NA
37295 day6 - UKWN - run2
PB2
PB1
PA
NP
M
NS
37295 day6 - UKWN - run1
Depth
dSNP count
dSNP Freq.

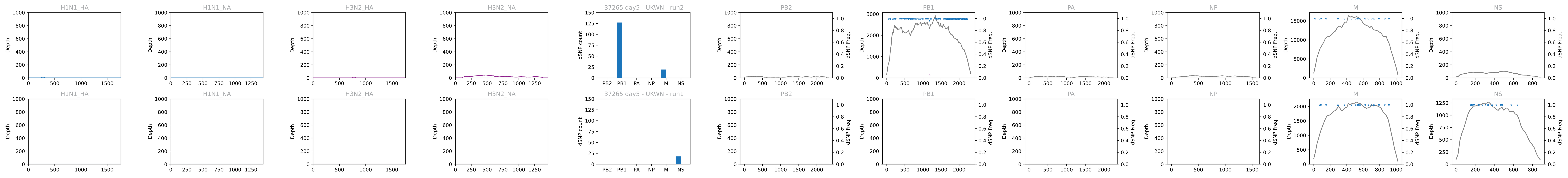
H1N1_HA
H1N1_NA
H3N2_HA
H3N2_NA
37265 day5 - UKWN - run2
PB2
PB1
PA
NP
M
NS
37265 day5 - UKWN - run1
Depth
dSNP count
dSNP Freq.

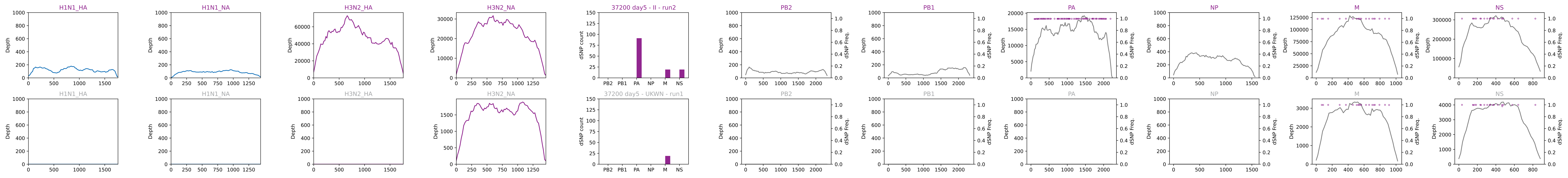
H1N1_HA
H1N1_NA
H3N2_HA
H3N2_NA
37200 day5 - II - run2
PB2
PB1
PA
NP
M
NS
H1N1_HA
H1N1_NA
H3N2_HA
H3N2_NA
37200 day5 - UKWN - run1
PB2
PB1
PA
NP
M
NS
Depth
dSNP count
dSNP Freq.
PB2 PB1 PA NP M NS

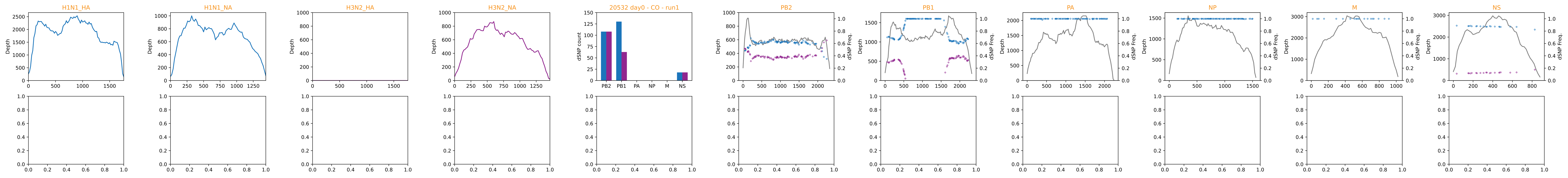

H1N1_HA
H1N1_NA
H3N2_HA
H3N2_NA
20532 day0 - CO - run1
PB2
PB1
PA
NP
M
NS
Depth
dSNP count
dSNP Freq.

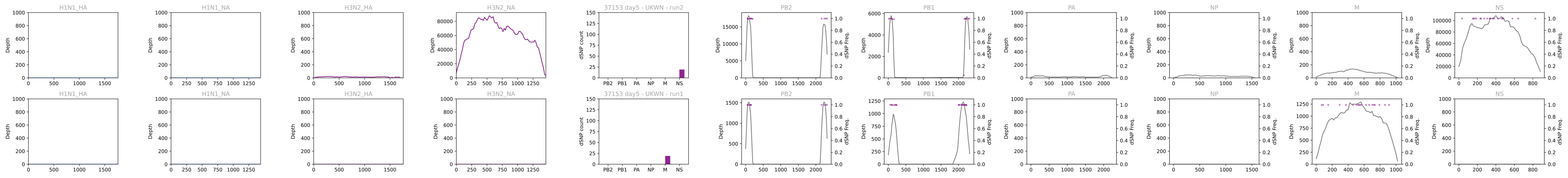
H1N1_HA
H1N1_NA
H3N2_HA
H3N2_NA
37153 day5 - UKWN - run2
PB2
PB1
PA
NP
M
NS
37153 day5 - UKWN - run1
Depth
dSNP count
dSNP Freq.

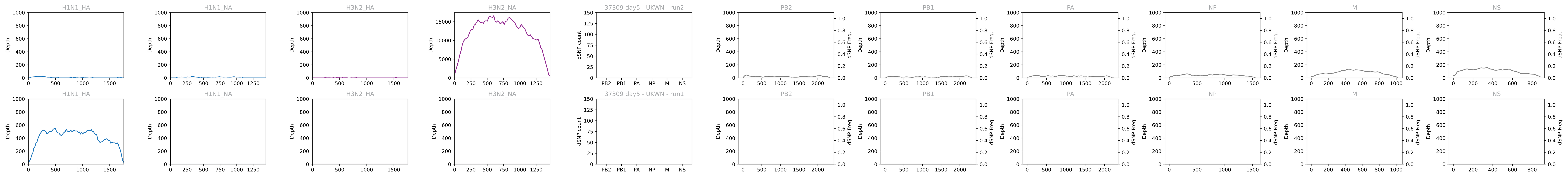
H1N1_HA
H1N1_NA
H3N2_HA
H3N2_NA
37309 day5 - UKWN - run2
PB2
PB1
PA
NP
M
NS
H1N1_HA
H1N1_NA
H3N2_HA
H3N2_NA
37309 day5 - UKWN - run1
PB2
PB1
PA
NP
M
NS
Depth
dSNP count
dSNP Freq.

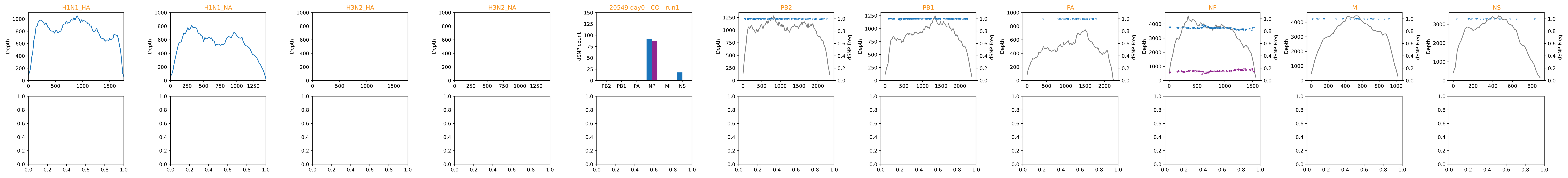
H1N1_HA
H1N1_NA
H3N2_HA
H3N2_NA
20549 day0 - CO - run1
PB2
PB1
PA
NP
M
NS
Depth
dSNP count
dSNP Freq.

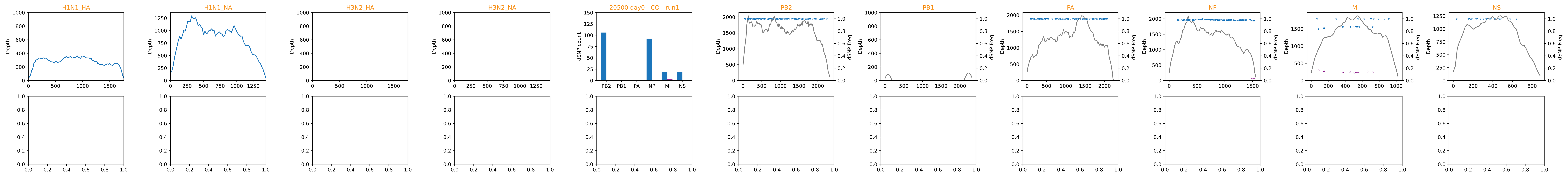

20500 day0 - CO - run1
H1N1_HA
H1N1_NA
H3N2_HA
H3N2_NA
PB2
PB1
PA
NP
M
NS
Depth
dSNP count
dSNP Freq.

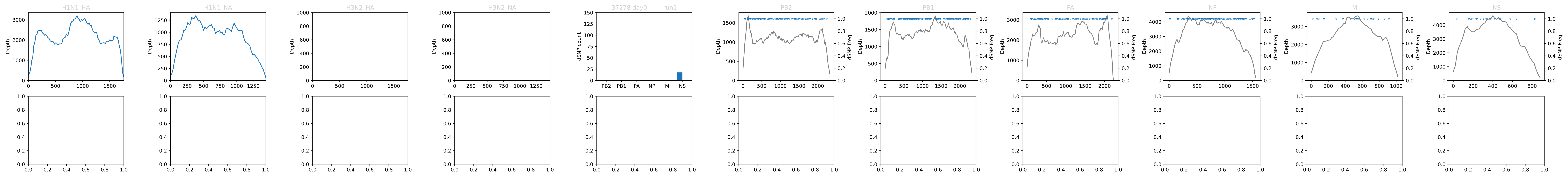
H1N1_HA
H1N1_NA
H3N2_HA
H3N2_NA
37278 day0 - -- - run1
PB2
PB1
PA
NP
M
NS
Depth
dSNP count
dSNP Freq.

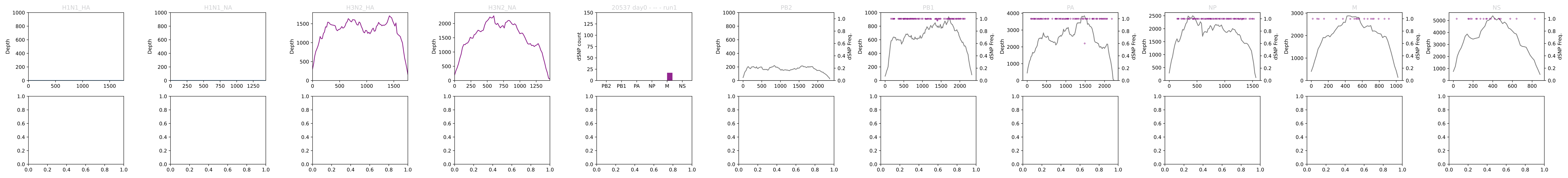

H1N1_HA
H1N1_NA
H3N2_HA
H3N2_NA
20537 day0 - -- - run1
PB2
PB1
PA
NP
M
NS
Depth
dSNP count
dSNP Freq.
PB2 PB1 PA NP M NS

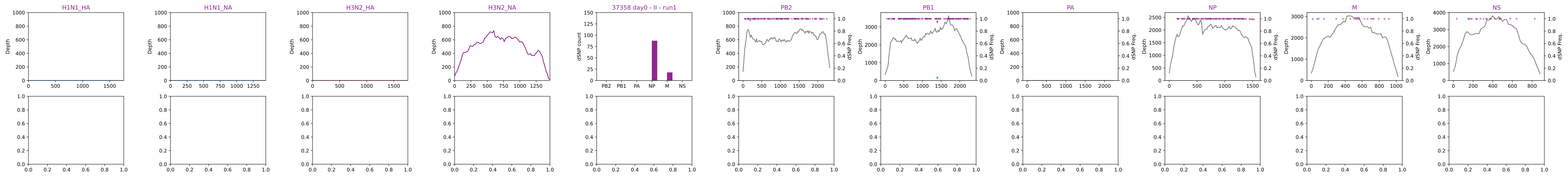
H1N1_HA
H1N1_NA
H3N2_HA
H3N2_NA
37358 day0 - II - run1
PB2
PB1
PA
NP
M
NS
Depth
dSNP count
dSNP Freq.

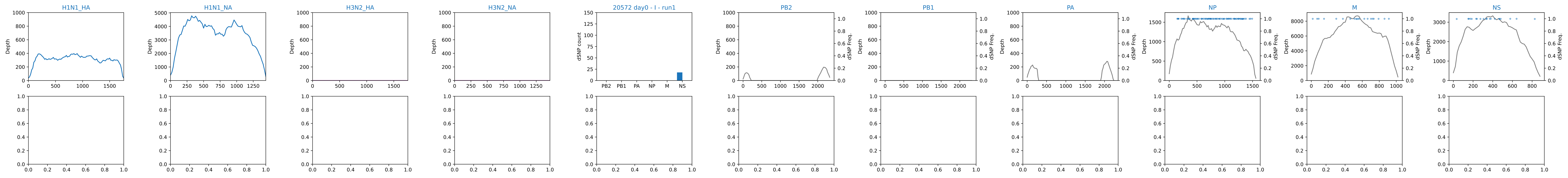
H1N1_HA
H1N1_NA
H3N2_HA
H3N2_NA
20572 day0 - I - run1
PB2
PB1
PA
NP
M
NS
Depth
dSNP count
dSNP Freq.

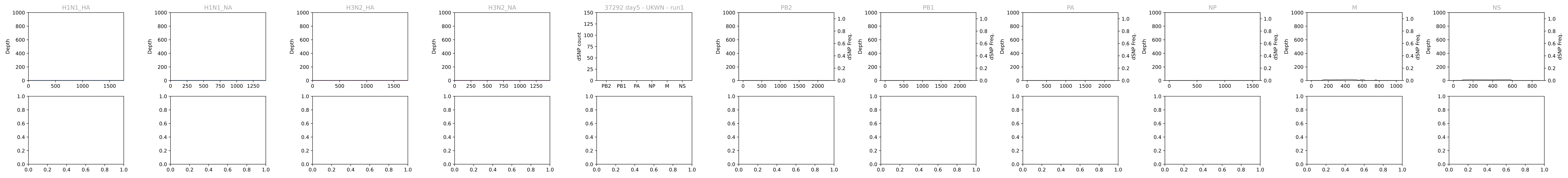
H1N1_HA
H1N1_NA
H3N2_HA
H3N2_NA
37292 day5 - UKWN - run1
PB2
PB1
PA
NP
M
NS
Depth
dSNP count
dSNP Freq.

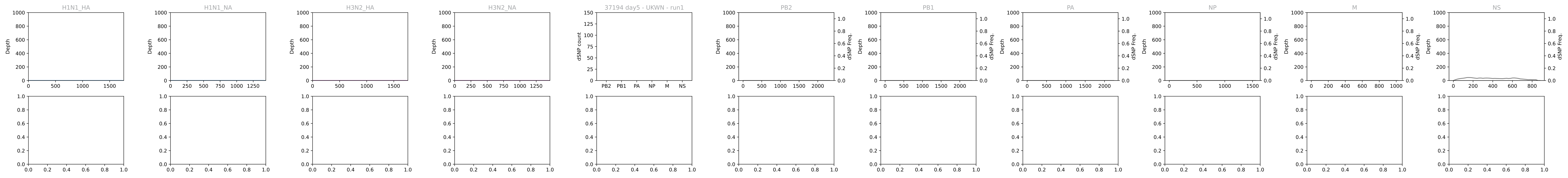

H1N1_HA
H1N1_NA
H3N2_HA
H3N2_NA
37194 day5 - UKWN - run1
PB2
PB1
PA
NP
M
NS
Depth
dSNP count
dSNP Freq.

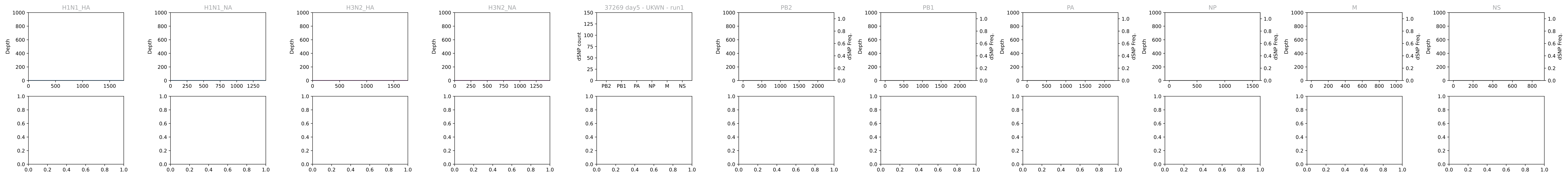
H1N1_HA
H1N1_NA
H3N2_HA
H3N2_NA
37269 day5 - UKWN - run1
PB2
PB1
PA
NP
M
NS
Depth
dSNP count
dSNP Freq.

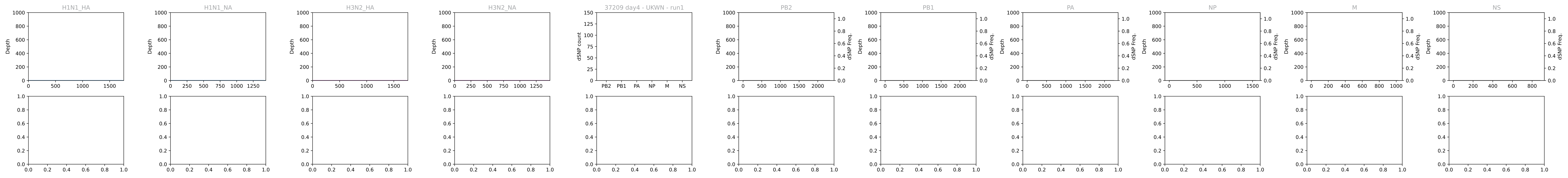
H1N1_HA
H1N1_NA
H3N2_HA
H3N2_NA
37209 day4 - UKWN - run1
PB2
PB1
PA
NP
M
NS
Depth
dSNP count
dSNP Freq.

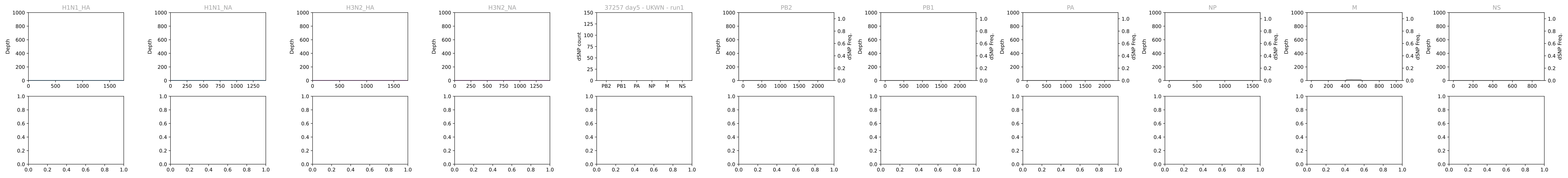
H1N1_HA
H1N1_NA
H3N2_HA
H3N2_NA
37257 day5 - UKWN - run1
PB2
PB1
PA
NP
M
NS
Depth
dSNP count
dSNP Freq.

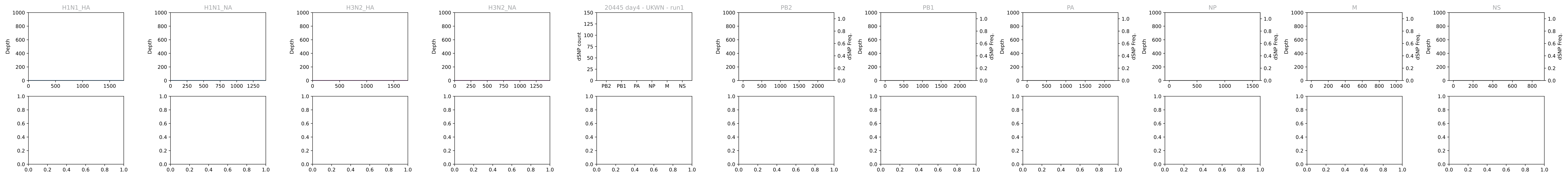
H1N1_HA
H1N1_NA
H3N2_HA
H3N2_NA
20445 day4 - UKWN - run1
PB2
PB1
PA
NP
M
NS
Depth
dSNP count
dSNP Freq.

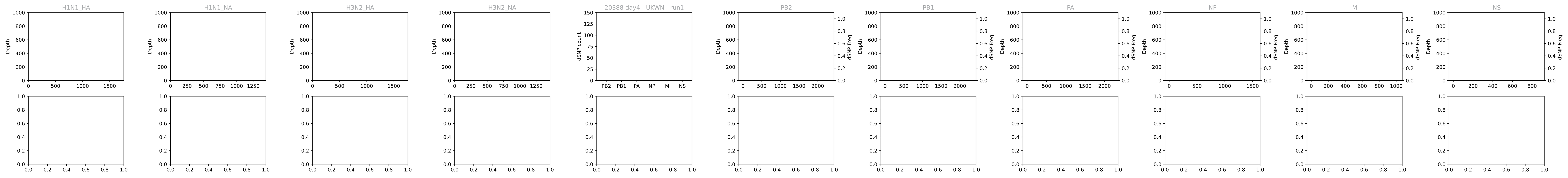
H1N1_HA
H1N1_NA
H3N2_HA
H3N2_NA
20388 day4 - UKWN - run1
PB2
PB1
PA
NP
M
NS
Depth
dSNP count
dSNP Freq.
PB2 PB1 PA NP M NS

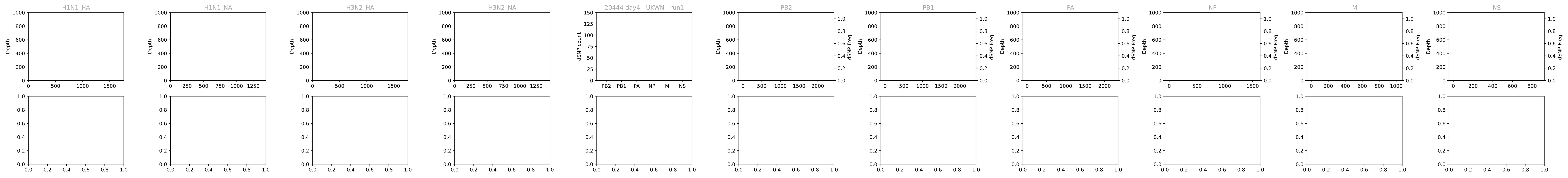
H1N1_HA
H1N1_NA
H3N2_HA
H3N2_NA
20444 day4 - UKWN - run1
PB2
PB1
PA
NP
M
NS
Depth
dSNP count
dSNP Freq.

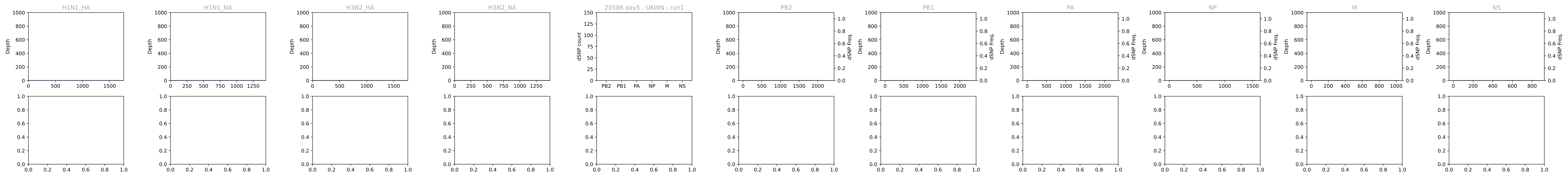
H1N1_HA
H1N1_NA
H3N2_HA
H3N2_NA
20586 day5 - UKWN - run1
PB2
PB1
PA
NP
M
NS
Depth
dSNP count
dSNP Freq.

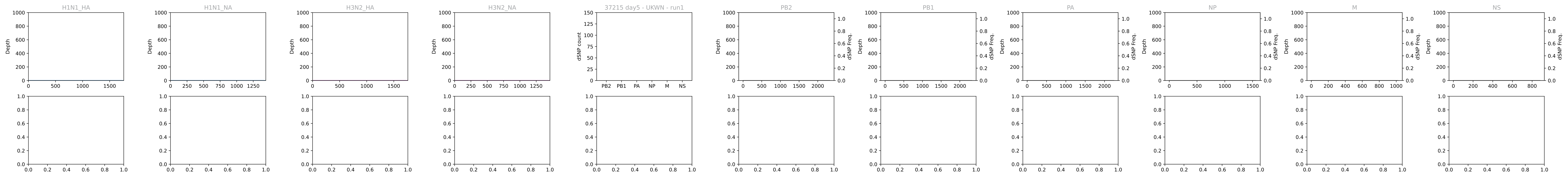

H1N1_HA
H1N1_NA
H3N2_HA
H3N2_NA
37215 day5 - UKWN - run1
PB2
PB1
PA
NP
M
NS
Depth
dSNP count
dSNP Freq.

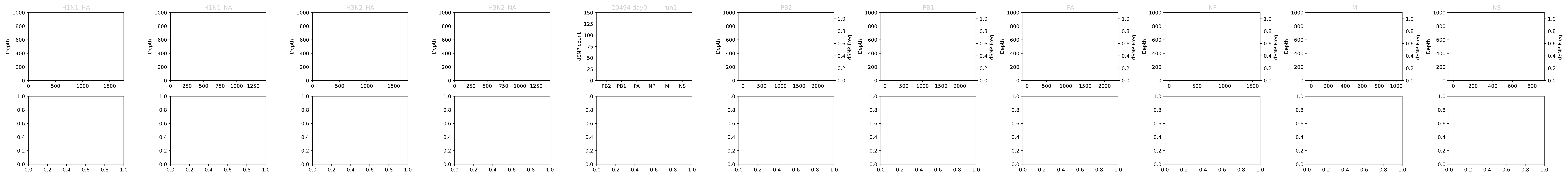

H1N1_HA
H1N1_NA
H3N2_HA
H3N2_NA
20494 day0 - -- - run1
PB2
PB1
PA
NP
M
NS
Depth
dSNP count
dSNP Freq.

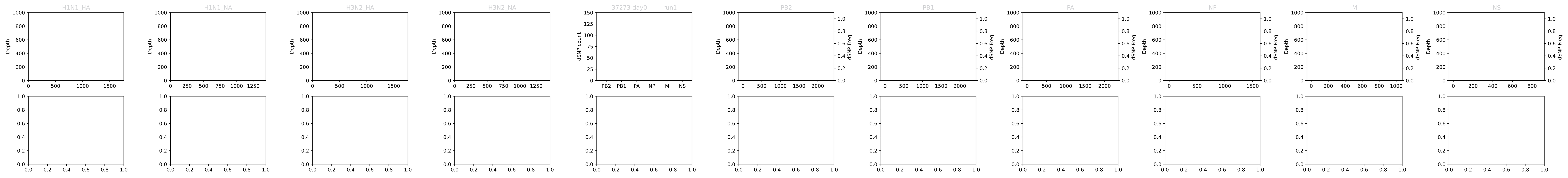

H1N1_HA
H1N1_NA
H3N2_HA
H3N2_NA
37273 day0 - -- - run1
PB2
PB1
PA
NP
M
NS
Depth
dSNP count
dSNP Freq.
